# Far-red-emitting Nile red with boosted solvatochromism enables multidimensional imaging of membrane heterogeneity and dynamics

**DOI:** 10.1016/j.xinn.2026.101319

**Published:** 2026-02-24

**Authors:** Mingqiao Liu, Yunzhe Fu, Xiaopeng Li, Haolin Zhang, Yumiao Ma, Meiqi Li, Chunyan Shan, Hongxi Zheng, Xiaoyi Lu, Yan Zhang, Li Yu, Peng Xi, Zhixing Chen

**Affiliations:** 1 College of Future Technology, Institute of Molecular Medicine, National Biomedical Imaging Center, Beijing Key Laboratory of Cardiometabolic Molecular Medicine, Peking University, Beijing 100871, China; 2 Academy for Advanced Interdisciplinary Studies, Peking University, Beijing 100871, China; 3 Department of Biomedical Engineering, National Biomedical Imaging Center, College of Future Technology, Peking University, Beijing 100871, China; 4 State Key Laboratory of Membrane Biology, Tsinghua University-Peking University Joint Centre for Life Sciences, Beijing Frontier Research Center for Biological Structure, School of Life Sciences, Tsinghua University, Beijing 100084, China; 5 BSJ Institute, Beijing 100084, China; 6 School of Life Sciences, Peking University, Beijing 100871, China; 7 Faculty of Natural, Mathematical & Engineering Sciences, King’s College London, London WC2R2ls, UK; 8 Peking-Tsinghua Center for Life Sciences, Academy for Advanced Interdisciplinary Studies Peking University, Beijing 100871, China

**Keywords:** fluorescence imaging, environment-sensitive probe, spectrum and polarization optical tomography, membrane heterogeneity, migrasome

## Abstract

The heterogeneity of lipid membranes plays an essential role in regulating the biological functions of organelles. As a solvatochromic probe, Nile red exhibits optical responses to varying membrane environments; however, its limited solvatochromism restricts its ability to detect subtle changes in membrane polarity and lipid order. In this work, we rationally design the auxochrome of Nile red by incorporating an oxygenated julolidine moiety and develop oxygenated julolidine-based Nile red (JONR), which exhibits significantly enhanced sensitivity to environmental polarity, along with strong fluorogenicity from protic to aprotic solvents, large Stokes shifts, and far-red emission in polar environments. Compared to Nile red, JONR demonstrates 3-fold greater accuracy in identifying various organelles in live cells and enables real-time tracking of their dynamic interactions through ratiometric imaging. Furthermore, JONR can distinguish lipid environments through polarization modulation depth and fluorescence lifetime variations. By integrating these properties with its superior spectral response, we present the first comprehensive mapping of subtle lipid heterogeneity within migration-associated structures in L929 cells. The high sensitivity to subtle differences in lipid environments of JONR enables fine resolution of subcellular membrane structures in live cells and provides a new approach for high-throughput analysis of intracellular microenvironments.

## Public summary

• JONR has a far-red emission spectrum and superior sensitivity to environmental changes in lipid membranes.
• JONR enables accurate mapping of organelles and monitoring of organelle interaction networks in live cells.
• JONR provides multidimensional optical responses, revealing differences in subcellular membrane heterogeneity.

## Graphical abstract

**Figure:**
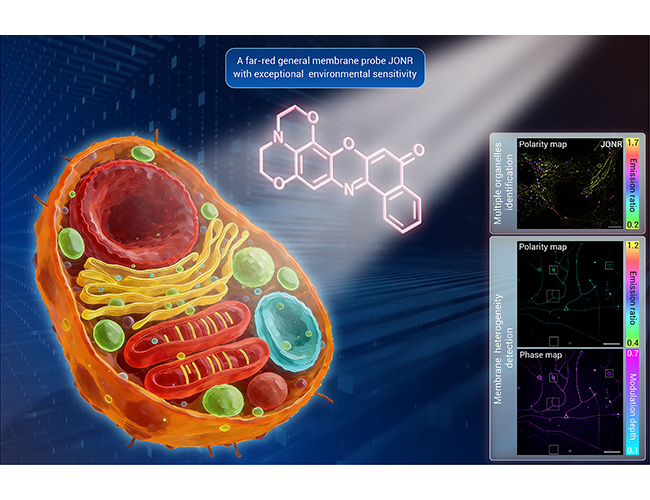

## Introduction

Lipids serve as essential structural components of cellular membranes, with mammalian systems harboring thousands of distinct lipid species that collectively establish intricate internal mechanisms of cells.^1^^,^^2^ The biophysical properties of membranes are primarily determined by their lipid composition. Cholesterol (CL) and sphingomyelin (SM) form a highly packed state, called the liquid-ordered (L_o_) phase, while unsaturated lipids mainly form a loosely packed liquid-disordered (L_d_) phase.^3^^,^^4^^,^^5^ Remarkably, the spatial distribution and relative abundance of these phases exhibit pronounced heterogeneity not only across cell types but also at subcellular levels.^6^ Therefore, the biological relevance and regulatory mechanisms of membrane phase heterogeneity in health and disease remain central topics in membrane biology.^1^ Environment-sensitive probe-based imaging is a powerful approach to visualize fine variations in membrane composition and to quantify lipid packing density, phase order, and membrane tension with subcellular precision.^7^^,^^8^^,^^9^

Nile red (NR) is a lipophilic solvatochromic fluorophore whose solvatochromism arises from a strong excited-state dipole moment. In highly polar solvents, the Franck-Condon excited state is stabilized by the dipoles of surrounding solvent molecules, resulting in a redshifted fluorescence emission.^10^^,^^11^ Compared to other solvatochromic dyes (e.g., Prodan and di-4-ANEPPDHQ), NR exhibits red fluorescence, high quantum yield (QY), and good photostability.^3^^,^^12^^,^^13^ In addition, NR binds preferentially to hydrophobic membrane regions and shows low fluorescence in aqueous environments but strong fluorescence in apolar lipid environments.^13^^,^^14^^,^^15^ These properties enable NR to function as a “wash-free” probe.^16^^,^^17^^,^^18^

**Figure 1:**
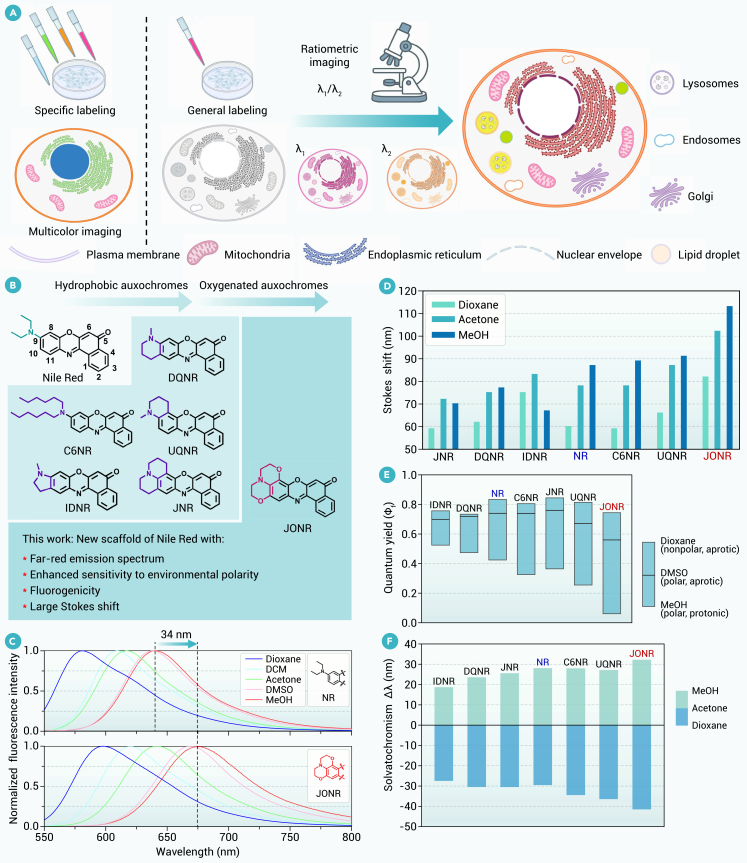
Principles of imaging multiple subcellular structures along with the design and optical properties of new NR derivatives (A) Workflow of the general labeling strategy for imaging and profiling subcellular structures and its advantages over specific labeling. Under the specific labeling strategy (left), conventional multicolor imaging requires distinct markers for each organelle, with limited multiplexing capacity (typically up to four fluorophores in the visible range) and reduced labeling efficiency as the number of targets increases. In contrast, the general labeling strategy (right) employs a single lipophilic probe to stain nearly all lipid-containing structures in live cells. Ratiometric imaging or spectral unmixing is then used to resolve emission shifts among lipid structures at different subcellular locations, enabling analysis of lipid composition or segmentation of subcellular compartments based on their spectral properties. (B) Design strategy and chemical structures of the new NR derivatives. (C) Emission spectra of NR and JONR. (D) Comparison of the Stokes shifts of the NRs. The derivatives are listed in order of their maximum Stokes shift across various solvents. (E) Comparison of the QYs. The derivatives are listed in order of their brightness response to different solvents. (F) Solvatochromism in the emission band of the NRs. The derivatives are listed in order of their solvatochromism. Concentrations of the derivative solutions: 2 μM.

Building on the unique advantages of NR for imaging lipid structures, solvatochromic probes have been developed to detect the lipid order of the plasma membrane,^19^ achieve super-resolution mapping of membrane organization,^20^ and monitor the lipid order in organelles under stress conditions.^21^ In addition, various optical modulation and detection strategies have been introduced to improve imaging resolution and throughput. By recording emission spectra at the detection side, differences in NR fluorescence can be analyzed to resolve lipid composition at the single-pixel level.^22^ Furthermore, the integration of ratiometric imaging with polarized excitation, termed spectrum and polarization optical tomography (SPOT), enables simultaneous detection of spectral shifts and fluorescence dipole orientations.^23^^,^^24^^,^^25^^,^^26^ By correlating lipid polarity with phase behavior, SPOT allows identification of lipid heterogeneity across multiple organelles, offering a scalable and high-throughput alternative to conventional specific labeling approaches, which are limited by spectral crosstalk and suffer from decreased labeling efficiency as the number of targets increases (Figure 1A). A recent study also achieved simultaneous imaging and identification of multiple structures by utilizing fluorescent proteins with distinct fluorescence lifetimes in combination with fluorescence lifetime microscopy (FLIM).^27^

These methods have provided valuable tools for membrane biology research, but the analytical precision remains substantially limited. This limitation hampers high-throughput organelle identification and accurate detection of subtle lipid variations at sub-organellar resolution. The fundamental barrier lies in the limited sensitivity of the fluorescent probe to small differences in the microenvironment. The response of NR to environmental polarity remains less sensitive than that of other solvatochromic fluorophores.^8^ Current modifications of the NR molecular structure are limited, with most efforts focused on functionalizing its core scaffold.^10^^,^^28^ Previous studies have introduced electron withdrawing-donating groups into the benzo[a]phenoxazone moiety or extended its conjugated structure via Suzuki coupling.^29^^,^^30^^,^^31^ However, these modifications have had minimal impact on solvatochromism. Only a few studies have modified the diethylamino group but have had negligible effects on the spectral properties of NR.^32^^,^^33^^,^^34^

Here, we design and synthesize a series of NR derivatives through rational auxochrome engineering. Beyond auxochromes, which may induce spectral red-shifts, we introduce electronegative atoms into the conjugated system of NR.^35^ Among these derivatives, oxygenated julolidine-based NR (JONR), incorporating 2,3,4,5-tetrahydro-1,6-dioxa-3a-azaphenalene (TDAP) as an auxochrome, offers the following advantages: bathochromically shifted emission spectra extending into the far-red region, a large Stokes shift, strong fluorogenicity from protic solvents to aprotic solvents, and, most importantly, superior sensitivity to environmental polarity (Figure 1). Benefitting from these advantages, simultaneous resolution of lipid membrane heterogeneity across distinct subcellular structures in COS-7 cells was achieved through ratiometric imaging. JONR provides 3-fold more accurate identification of morphologically similar organelles than conventional NR probes. Without the need for multicolor labeling, the dynamic extension of endoplasmic reticulum (ER) tubules and “hitchhiking” behavior on rapidly moving late endosomes (LEs) were visualized. Furthermore, JONR exhibits responsiveness to distinct lipid microenvironments through polarization modulation depth and fluorescence lifetime variations. In combination with the superior spectral responses of JONR, we capture subtle compositional and structural heterogeneity within migration-associated structures, including mitochondria, migrasomes, retractosomes, and retraction fibers in L929 cells.

## Materials and methods

### UV-visible and fluorescence spectroscopy

The absorption and emission spectra of the new NR dyes in spectrum-grade solvents including dioxane, acetone, dichloromethane (DCM), dimethyl sulfoxide (DMSO), methanol (MeOH), methanol-*d4* (CD_3_OD), ethanol (EtOH), isopropanol (IPA), n-butanol (n-BuOH), and ethylene glycol (EG) were measured using a Duetta fluorescence and absorbance spectrometer (Horiba, Kyoto, Japan) in a 1 cm square quartz cuvette. All measurements were taken at ambient temperature (23°C ± 2°C). Fluorescent molecules were prepared as stock solutions in DMSO and diluted to keep the DMSO concentration below 1% (v/v).

### QY determination

Fluorescence QYs of the new NR dyes in spectrum-grade solvents (dioxane, acetone, DCM, DMSO, MeOH, CD_3_OD, EtOH, IPA, n-BuOH, and EG) were determined via the relative determination method. They were determined with rhodamine B (Φ_f_ = 0.31 in H_2_O) as a reference. The absorbance of the sample and reference at their respective excitation wavelengths was controlled to be lower than 0.1. Reported values are averages (*n* = 4). Fluorescence QYs, Φ_f_ (sample), were calculated by the following equation:

$$\frac{\Phi_{f,sample}}{\Phi_{f,ref}}=\frac{{OD}_{ref}I_{sample}d_{\text{sample}}^{2}}{{OD}_{\text{sample}}I_{\text{ref}}d_{\text{ref}}^{2}}\text{,}$$

where Φ_f_ is the QY of fluorescence; I is the integrated emission intensity; OD is the optical density at the excitation wavelength; d is the refractive index of solvents, d_water_ = 1.33; d_dioxane_ = 1.418; d_acetone_ = 1.424; d_DCM_ = 1.36; d_DMSO_ = 1.48; d_MeOH_ = 1.329; d_EtOH_ = 1.361; d_IPA_ = 1.377; d_n-BuOH_ = 1.399; and d_EG_ = 1.432

### Extinction coefficient

Fluorescent dyes were quantified by quantitative nuclear magnetic resonance (qNMR) using a toluene standard (2.0 μL). A dilution series of 0.5, 1.5, and 2.5 μM of fluorophore was prepared in spectrum-grade solvents for *ε* (extinction coefficient). Absorbance spectra were recorded on a Duetta fluorescence and absorbance spectrometer (Horiba), and the absorbance maxima were plotted against the concentration. A linear function was fitted to the data using Origin 2023, and extinction coefficients were calculated from the slope following Lambert-Beer’s law. Reported values for *ε* are averages (*n* = 3).

### Bulk bleaching measurement

Dyes were immobilized within polymethylmethacrylate (PMMA) films prepared using an SW-4A spin coater (Setcas, Beijing, China) and round glass coverslips (diameter = 25 mm) as substrates. 100 μL 5% PMMA in chloroform containing 4 μM dye was applied to the coverslip, followed by spin coating (80,000 rpm, 10 s; 800,000 rpm, 45 s). These polymer films were then irradiated using an LSM880 confocal microscope (Carl Zeiss, Oberkochen, Germany) equipped with a 20× Air Plan Apochromat 20×/0.8 NA (numerical aperture) objective. Three groups of time-lapse images (200 frames, 8.2 × 8.2 μm, 1.0 fps (frames per second), 100% laser power) were acquired for each dye, and quantification of fluorescence intensity was achieved using Fiji software from three parallel experiments.

### Measurement of singlet oxygen QY

Singlet oxygen QYs were determined using 1,3-diphenylisobenzofuran (DPBF; Q105708, Dibai, Shanghai, China) as a chemical indicator. Dyes and 80 μM DPBF were dissolved in air-saturated MeOH, followed by irradiation with an light emitting diode (LED) lamp (520–530 nm, 50 mW/cm^2^). For JONR, NR, and tetramethyl rhodamine ethyl ester (TMRE), the dyes were corrected for concentration so that their absorbance values at 525 nm were equal. DPBF absorption was monitored at 415 nm. The linear decay slope of 415 nm is positively correlated with singlet oxygen QY. TMRE in acetonitrile (*ϕ* = 0.023) was used as a reference.

### GUV preparation and imaging

1,2-Dioleoyl-*sn*-glycero-3-phosphocholine (DOPC), CL, and SM were used in a giant unilamellar vesicle (GUV) preparation. Three kinds of lipids—pure DOPC, DOPC:CL 7:3, and SM:CL 7:3—were dissolved in CHCl_3_ to a final concentration of 10 mM. GUVs were prepared using the electroformation technique by Vesicle Prep Pro (Nanion Technologies). To immobilize lipid vesicles for confocal imaging, the GUV solution supplemented with 10 mM of NR or JONR was mixed with 0.5 wt % low-gelling-temperature agarose (Sigma) solution at a 1:1 ratio. The mixture was dropped onto a glass slide and was imaged with the SPOT system and FLIM.

### Cell culture

COS-7 cells (CRL-1651) were cultured in high-glucose DMEM (Gibco, 21063029) supplemented with 10% fetal bovine serum (FBS; Gibco) and 1% 100 mM sodium pyruvate solution in a 37°C incubator with 5% CO_2_. L929 cells were cultured in DMEM (Gibco) supplemented with 10% FBS (Excell), 2 mM GlutaMAX, and 100 U/mL penicillin-streptomycin in 5% CO_2_ at 37°C. When the cells reached 80% confluence, they were detached using trypsin-EDTA and subsequently seeded onto 8-chambered cover glasses (Cellvis, C8SB-1.5H) for imaging experiments.

### Cell staining process

Stock solutions of the new NR analogs were added to the standard culture medium, with a recommended final concentration of 500 nM. Cells were incubated with the dyes for 20 min, after which they were ready for fluorescence imaging without requiring any additional processing steps. JONR staining is insensitive to the presence or absence of serum in the culture medium. Furthermore, since JONR is non-fluorescent in aqueous solution, no washing steps are necessary post staining, allowing immediate imaging.

## Results and discussion

### Design and synthesis of the new NR family

We aim to improve the solvatochromism of NR by enhancing intramolecular charge transfer in the excited state. The design strategy first modified the diethylamino group of the NR fluorophore into hydrophobic auxochromes to improve electron-donating effects. Furthermore, polar substituents were introduced to extend π conjugation, where multiple polar centers may strengthen dipole-dipole interactions or hydrogen bonds between the dye and solvent molecules, thereby enhancing solvatochromism. We introduced the oxygen atom as both a polar substituent and a conjugated electron-donating group into the NR scaffold. The NR with the diethylamino group replaced by a six-carbon chain is referred to as C6NR. Three conformationally restricted auxochromes were selected: julolidine, tetrahydroquinoline, and indoline. The corresponding NR derivatives are named as follows: JNR (julolidine), DQNR (Nile Red with the quinoline ring in the down position), and UQNR (Nile Red with the quinoline ring in the up position) (1-methyl-1,2,3,4-tetrahydroquinoline, with UQNR having the ring at the 8 position), and IDNR (1-methylindoline). Building upon the JNR scaffold, TDAP was designed as an advanced auxochrome featuring two oxygen atoms integrated into the conjugated system. This optimized derivative was subsequently employed to synthesize the JONR (oxygenated JNR) fluorophore (Figure 1B). The new NR fluorophores were synthesized following a published protocol involving two key reaction steps: a nitroso substitution reaction and a condensation-cyclization sequence, with reaction conditions optimized to achieve a final yield of over 60% for all products.^30^

### Impact of new auxochromes on NR optical properties

To systematically investigate the solvatochromism of the new analogues, *E*_T_(30) was selected as the metric for solvent polarity.^11^ Dioxane was chosen as the solvent with the lowest polarity, while DCM and acetone were selected as solvents with medium polarity. For high-polarity solvents, we opted for the aprotic solvent DMSO and the protic solvent MeOH. The solvatochromism was defined as the difference between the longest and shortest maximum emission wavelength (λ^em^_max_) observed across these solvents.

The spectral data indicate that all modification strategies resulted in a redshift in the emission wavelengths of the new NR derivatives across various solvents, which is consistent with our hypothesis (Table 1). Notably, the emission spectra of JONR in DMSO and MeOH extend into the far-red region (>650 nm), exhibiting a 34 nm redshift in the maximum emission wavelength compared to NR (Figure 1C). The maximum absorption wavelength of JONR is close to that of NR, resulting in a significantly larger Stokes shift among all analogs (Figures 1D and S7).

**Table 1:** Spectra and photophysical properties of the NR analogs in selected solvents

Dye	Solvent	λ^abs^_max_ (nm)	λ^em^_max_ (nm)	Solvatochromism (nm)	Stokes shift (nm)	Φ_f_
NR	dioxane	520	581	59	61	0.82
	acetone	532	611		79	0.82
	MeOH	552	640		88	0.42
C6NR	dioxane	520	580	64	60	0.81
	acetone	536	615		79	0.76
	MeOH	554	644		90	0.32
DQNR	dioxane	528	591	55	63	0.74
	acetone	546	622		76	0.71
	MeOH	568	646		78	0.47
UQNR	dioxane	522	589	65	67	0.82
	acetone	538	626		88	0.75
	MeOH	562	654		92	0.25
JNR	dioxane	544	604	57	60	0.85
	acetone	562	635		73	0.84
	MeOH	590	661		71	0.36
IDNR	dioxane	524	600	42	76	0.76
	acetone	544	628		84	0.77
	MeOH	574	642		68	0.52
JONR	dioxane	516	599	75	83	0.75
	acetone	538	641		103	0.69
	MeOH	560	674		114	0.055

The effect of auxochromes on the solvatochromism of NR is a key focus. In the case of NR, changing the solvent from acetone to dioxane and methanol causes shifts in the peak emission of −30 and +29 nm, respectively, giving a total solvatochromism of 59 nm. Among these new analogs, C6NR, UQNR, and JONR exhibit enhanced solvatochromism (Figure 1F; Table 1). JONR demonstrates a solvatochromism of 75 nm across different solvents, representing an improvement of over 25% compared to NR. Additionally, we found that the fluorescence of JONR is significantly quenched by protic solvents such as MeOH and water. The QY of JONR in MeOH is 5.5%, while it exhibits almost no fluorescence in water. Compared to its performance in the polar aprotic solvent DMSO, the QY of JONR exhibits nearly a 10-fold increase (Figure 1E). Although it has been previously observed that the QY of NR decreases in polar protic solvents, JONR demonstrates the most significant fluorogenicity. This would help minimize background fluorescence in membrane imaging and enable wash-free labeling.

We also evaluated four aspects of JONR and the reference compounds NR and TMRE: (1) photostability, (2) *in vitro* singlet oxygen generation, (3) phototoxicity based on cell apoptosis, and (4) cytotoxicity. Both JONR and NR demonstrate similar phototoxicity, photostability, and singlet oxygen generation while showing no significant cytotoxicity (Figure S8).

### Computational results explain the optical properties exhibited by JONR

The redshifted spectra and improved solvatochromism exhibited by JONR align with our rational design. Through theoretical analysis, we compared the excited states of the structurally similar JONR and JNR to explain these properties. First, the computed S_1_ emission wavelength for NR in DMSO was tested using various popular functionals in combination with the TZVP (triple zeta valence plus polarization) basis set, and according to the benchmark result, TPSSh (Tao, Perdew, Staroverov, and Scuseria hybrid functional) was selected for further computation (Table S1).

**Figure 2:**
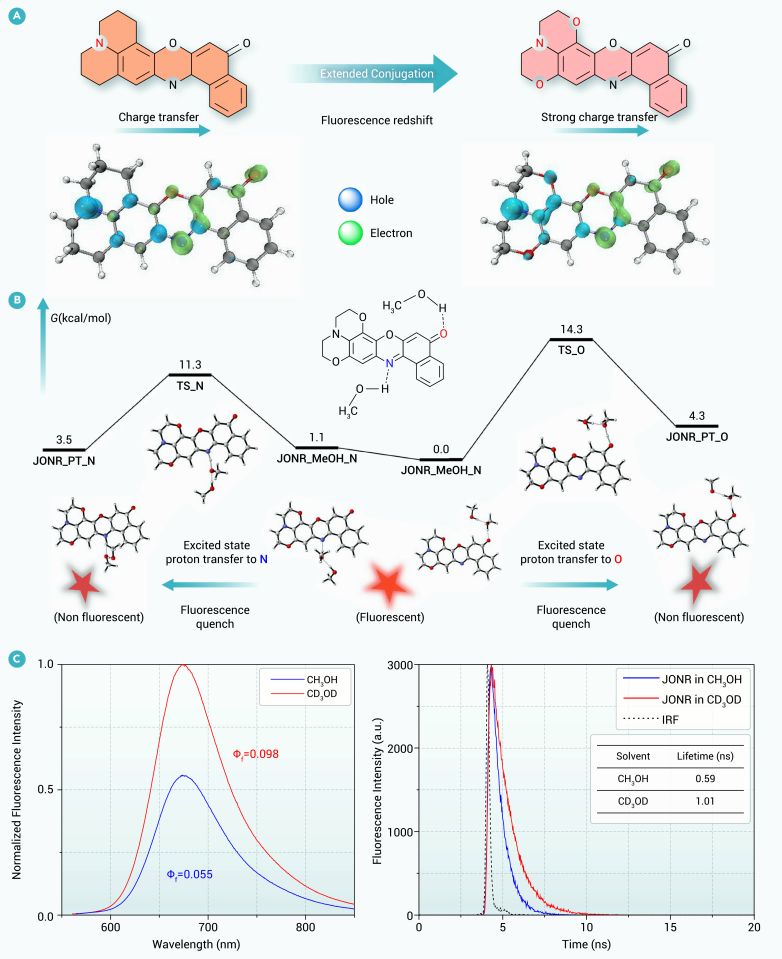
Theoretical analysis and experimental verification of the photophysical properties of JONR (A) S_1_ state of the hole (blue) and electron (green) isosurface for the S_1_ state of JNR and JONR. The stronger charge transfer accounts for the redshifted spectra of JONR and its improved solvatochromism. The isovalue is taken to be 0.005. (B) Gibbs free energy for the EPT between the S_1_ state of JONR and MeOH. In protic solvents, EPT to either nitrogen or oxygen atoms leads to fluorescence quenching of JONR. (C) Normalized fluorescence spectra (left) and fluorescence lifetime decay curves (right) of JONR (2 μM) in MeOH (blue) and CD_3_OD (red). The instrument response function (IRF) is presented as a dashed line (black).

The computational results at the TPSSh-SMD (solvent model density)/TZVP level show that both JNR and JONR exhibit a significant redshift in polar solvents and that the response for JONR is greater (Table 2). The origin of the redshift is revealed by excited-state hole-electron analysis.^36^ In this analysis, the spatial distribution of electron transfer upon excitation is visualized by the hole and electron isosurface. The S_1_ states of both JNR and JONR exhibit typical charge transfer excitation, in which the electron from the arylamine motif is transferred to the electron-deficient phenoxazin-5(5H)-one fragment (Figure 2A). The hole-electron mass center distance (D index) was employed to quantify the charge transfer character. JONR exhibits a larger D index (2.758 Å) than JNR (2.203 Å), indicating greater spatial separation between holes and electrons and a stronger charge transfer character in JONR. For JONR, the alkoxyl substitute further contributes to the charge transfer, revealed by the extended hole distribution on the oxygen atoms (Figure 2A), which leads to its redshift over JNR. The improved charge transfer nature is also responsible for the stronger solvent response. In the polar solvent, the charge separation resulting from the introduced oxygen atoms in the S_1_ state is further stabilized, leading to the observed redshift and a stronger solvent response (Table 2).

**Table 2:** Computed S_1_ emission wavelength and oscillator strength for JNR and JONR in selected solvents

Solvent	S_1_ emission wavelength (nm)	*f* (a.u.)
**JNR**		

DCM	642	1.0852
DMSO	648	1.1600
MeOH	665	1.1736

**JONR**		

DCM	637	0.9773
DMSO	664	1.0400
MeOH	691	0.9793

Two methanol molecules are included to calculate the emission wavelength and oscillator strength data in methanol.

Additionally, we elucidated the fluorescence quenching phenomenon of JONR in protic solvents through the mechanism of excited-state proton transfer (EPT). The S_1_ state oscillator is minimally affected by the solvent polarity (Table 2), which prompted us to investigate the S_1_ states in the presence of explicit MeOH molecules. The two possible hydrogen bonding sites—namely the nitrogen and the carbonyl oxygen atoms—were separately investigated. Similarly, the explicit MeOH binding makes a negligible change to the emission wavelength and oscillator strength (JONR_MeOH_N and JONR_MeOH_O). However, the presence of MeOH in this complex enables rapid EPT processes through two transition states, TS_O or TS_N. The resulting protonated JONR-methoxide complex (JONR_PT_O or JONR_PT_N) exhibits a dark state with negligible oscillator strength and no emission (Figure 2B). Although JONR_PT_O and JONR_PT_N possess higher free energy than JONR, the EPT process may open additional decay pathways and therefore be responsible for the quenching in protic solvents.

To verify the existence of the EPT mechanism, we studied the photophysical properties of JONR in various protic solvents. The results showed that JONR exhibits fluorescence quenching in all tested protic solvents, with the quenching efficiency increasing as the solvent pKa decreases and polarity increases (Figures S9A and S9B). This quenching behavior was found to be independent of solvent viscosity (Figures S9C and S9D). Notably, despite the similar polarity of the protic solvent IPA (*E*_T_(30) = 48.4) and the aprotic solvent DMSO (*E*_T_(30) = 45.0), JONR exhibits significant fluorescence quenching in IPA (Figure S9E). Further evidence was obtained by comparing the fluorescence intensity of JONR in MeOHand CD_3_OD. Owing to the significantly reduced proton transfer efficiency in CD_3_OD compared to MeOH, JONR exhibited a 1.8-fold increase in QY and an approximate doubling of fluorescence lifetime compared to MeOH (Figure 2C). These results provide direct experimental support for the existence of the EPT mechanism.

### JONR enables higher-sensitivity detection of membrane heterogeneity at the subcellular level

**Figure 3:**
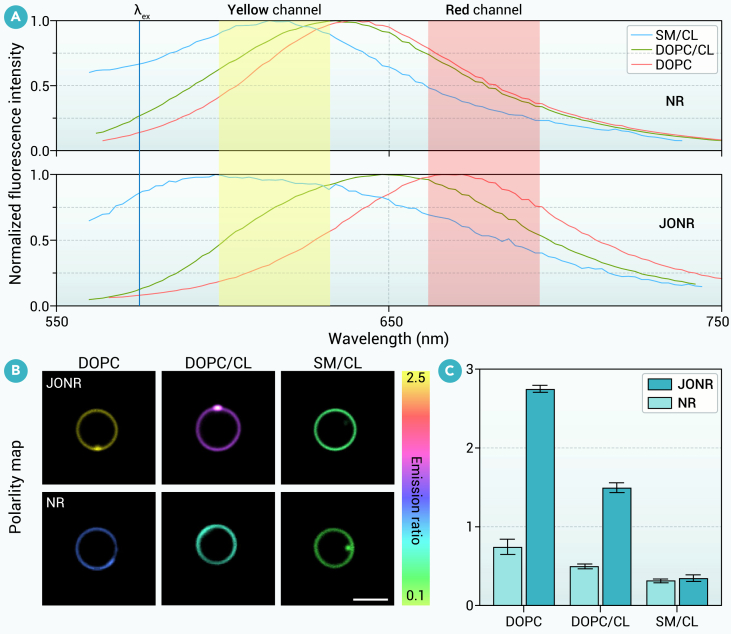
Ratiometric imaging of L_o_ and L_d_ phases in GUVs using NR and JONR (A) Normalized fluorescence spectra of NR and JONR from L_d_ (DOPC) to L_o_ (SM/CL) phases of GUVs. The blue vertical line represents the excitation wavelength (λ_ex_), while the yellow and red regions represent the detection range for the yellow and red channels of the microscope. (B) Polarity maps of GUVs with varying lipid compositions stained with JONR or NR. The color bar indicates the maximum and minimum emission ratio values, with different colors representing different levels of membrane polarity. (C) Quantification of emission ratios from GUVs stained with JONR or NR is shown in (B). Each condition was independently repeated at least five times. Data are shown as mean ± SEM. Scale bars: 1 μm.

NR is capable of responding to membrane polarity variations across different organelles; however, its limited environmental sensitivity prevents accurate identification of lipid compositional changes based on spectral shifts. It not only reduces the ability to resolve subcellular heterogeneity but also constrains the throughput of organelle-specific imaging and the capacity to monitor multiple subcellular interactions simultaneously. Conventional multicolor imaging, which relies on filter switching, often fails to meet these demands. To validate the application potential of JONR in membrane imaging, we assessed its environmental sensitivity compared to NR in dual-channel ratiometric imaging. Using 561 nm excitation (blue vertical line in Figure 3A), fluorescence signals from both the yellow and red emission channels (the yellow and red spectral windows in Figure 3A) were collected (Figure S10). The ratio of red to yellow fluorescence intensity was defined as the emission ratio. In polar environments, JONR shows a redshift in its emission spectrum and the emission ratio increases. In non-polar environments, the emission shifts toward blue, and the emission ratio decreases.

We used GUV models to quantitatively investigate the range of emission ratio values of JONR and compare its environmental sensitivity with NR in ratiometric imaging. Three types of GUV models were prepared: pure DOPC, DOPC:CL 7:3, and SM:CL 7:3. Pure DOPC vesicles are generally considered to represent the L_d_ phase, characterized by relatively high polarity, whereas SM:CL 7:3 vesicles are regarded as representing the L_o_ phase, which exhibits relatively low polarity. Spectral measurements revealed that JONR exhibited greater solvatochromism than NR in membrane environments, with a more pronounced redshift upon insertion into lipid bilayers (Figure 3A). Polarity maps obtained from ratiometric imaging further visualized the lipid composition heterogeneity among these vesicles (Figure 3B), showing that the environmental sensitivity of JONR was nearly 3-fold higher than that of NR (Figure 3C).

**Figure 4:**
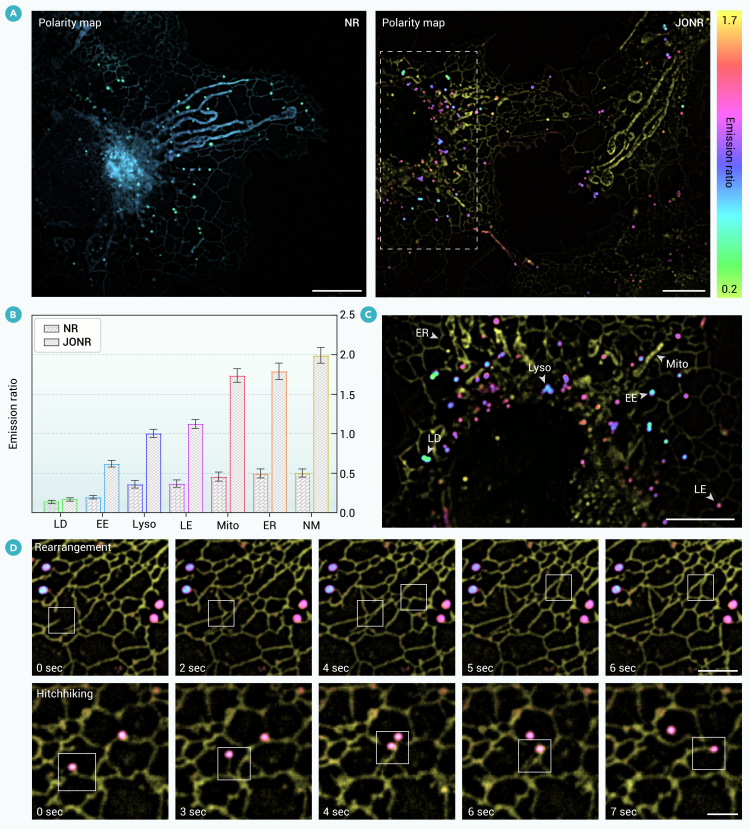
JONR exhibits superior responsiveness to lipid heterogeneity in live-cell ratiometric imaging (A) Polarity map of COS-7 cells showing the spatial distribution of membrane polarity based on NR labeling (left) and JONR labeling (right). (B) Comparison of emission ratios in different subcellular organelles labeled by JONR or NR. Each experiment was independently repeated at least five times. (C) High-magnification view of (B), demonstrating the identification of multiple organelles based on lipid morphology and emission ratio differences. (D) Dynamic interactions between organelles captured in live COS-7 cells. Top: rapid remodeling dynamics of the ER. Bottom: hitchhiking behavior of an LE (pink) along extending ER tubules (yellow). The color scale denotes the emission ratio range. Data are shown as mean ± SEM. Scale bars: 500 nm (D, bottom), 3 μm (D, top), and 10 μm (A and C).

To examine the ability of JONR to label lipid-rich subcellular compartments in live cells, we transfected COS-7 cells with GFP-fused plasmids targeting different organelles. After 24 h of expression, cells were labeled with either NR or JONR. The GFP channel served as a localization marker to identify specific subcellular structures. Co-localization analysis confirmed that JONR successfully labeled at least 14 distinct compartments (Figure S11). Under identical staining and imaging conditions, NR shows limited spectral response to different subcellular membrane structures, whereas JONR exhibits a much stronger ability to distinguish various lipid membranes (Figure 4A). We also evaluated other NR derivatives for live-cell ratiometric imaging (Figures S12A–S12G). Among all tested probes, JONR achieved high signal-to-noise ratios (SNR) in both the yellow and red channels while maintaining moderate brightness, supporting reliable ratiometric quantification (Figures S12H and S12I). Next, to quantitatively evaluate the responsiveness of JONR versus NR to lipid composition differences across subcellular compartments, we analyzed the emission ratio of JONR- and NR-labeled structures identified by GFP labeling. From lipid droplets (LDs) with low polarity to nuclear membranes (NMs) with high polarity, both JONR and NR showed increasing emission ratios, confirming their sensitivity to membrane polarity. However, JONR exhibited a markedly enhanced redshift in the most polar compartments, with an emission ratio exceeding 3-fold that of NR (Figure 4B). Across the tested subcellular membranes, JONR also exhibited a substantially larger emission ratio dynamic range than other NR derivatives, resulting in improved separation of distinct membrane compartments (Figure S12J). Even between adjacent compartments with comparable membrane polarity, such as lysosomes (Lyso) and LEs, JONR produced greater and more discernible ratio variations than NR. This highlights the superior sensitivity of JONR to subtle differences in membrane polarity, enabling fine identification between morphologically similar organelles. By combining emission ratio mapping and morphological features, we were able to simultaneously identify multiple organelles within live cells, including the ER, mitochondria, lysosomes, early endosomes (EEs), LEs, and LDs (Figure 4C).

Notably, JONR also enabled the capture of dynamic organelle interactions that previously required dual- or triple-labeling strategies. We focused on the ER, the largest intracellular organelle, which undergoes rapid remodeling and interacts extensively with other organelles. In 2018, Guo et al. uncovered new ER remodeling mechanisms, including hitchhiking of the ER on motile organelles and budding.^37^ Using SPOT imaging, we can not only visualize the dynamic extension events of ER tubules in real time but also observe the hitchhiking behavior of ER tubules attached to fast-moving LEs (Figure 4D), highlighting the significant advantages of general labeling and ratiometric imaging in studying organelle interactomes.

### Multidimensional imaging of membrane orientation and lipid order with JONR

To further expand the imaging dimension and reveal in-depth structural insights into subcellular membrane structures, we characterized the polarization properties of JONR on GUVs using SPOT imaging. Polarization modulation was applied at the excitation side, and the fluorescence intensity under varying excitation polarization angles was recorded (Figure S10). Fluorophores embedded in ordered membranes exhibited stronger modulation depth and more precisely resolved dipole orientations, suggesting restricted dipole mobility. In contrast, disordered membranes displayed reduced modulation depth (Figure S13A) and weaker polarization responses, with dipole orientations being resolved with lower precision (Figure S13B), consistent with increased dipole flexibility.^23^

We next performed FLIM imaging to collect fluorescence lifetimes of GUVs with distinct lipid compositions. Dipoles in ordered membranes exhibited prolonged relaxation times, indicating restricted rotational freedom, whereas disordered membranes displayed shorter lifetimes, reflecting increased rotational mobility (Figure S13C).^38^ The SPOT and FLIM measurements exhibited consistent trends (Figures S13D and S13E), indicating that JONR effectively reports membrane heterogeneity through both modulation depth and fluorescence lifetime. Together with emission ratio analysis, measurements of modulation depth and fluorescence lifetime enabled a systematic evaluation of how JONR responds to membrane heterogeneity through three distinct optical properties (Figure S13F).

Polarization modulation can further be applied to delineate the relative spatial relationships between membranes and other cellular structures, such as the cytoskeleton. The periodic membrane-associated skeleton on axons serves as a structural framework to organize cytoskeletal and membrane components with nanoscale regularity.^36^^,^^37^ We first labeled filamentous actin (F-actin) in cultured neurons with phalloidin-Alexa 488 (Figure S13G). In previous studies, the emission dipole of phalloidin-Alexa 488 was shown to align parallel to the F-actin axis.^39^ The resulting orientation map showed that the dipole orientations were consistent with the longitudinal extension of axons, suggesting that actin filaments within the membrane-associated skeleton are arranged side by side rather than end to end, in agreement with recent findings using polarization SIM (pSIM).^39^ We further performed SPOT imaging on JONR-labeled neuronal axonal membranes (Figure S13G) and the growth cone structures at the distal end of axons (Figure S13H). The orientation of membrane-bound dipoles was found to be perpendicular to the direction of axonal extension and orthogonal to the protrusion axis of filopodia emerging from the growth cone.^40^^,^^41^ These observations indicate that JONR dipoles align parallel to the phospholipid bilayer within the membrane, providing references for investigating the spatial relationships between membranes and other cellular structures. In parallel, FLIM revealed that the fluorescence lifetime of JONR on the axonal membrane is significantly longer than within intracellular mitochondria, suggesting a more ordered and tightly packed membrane environment at the axonal surface (Figure S14).

### Lipid properties of migration-associated subcellular structures

**Figure 5:**
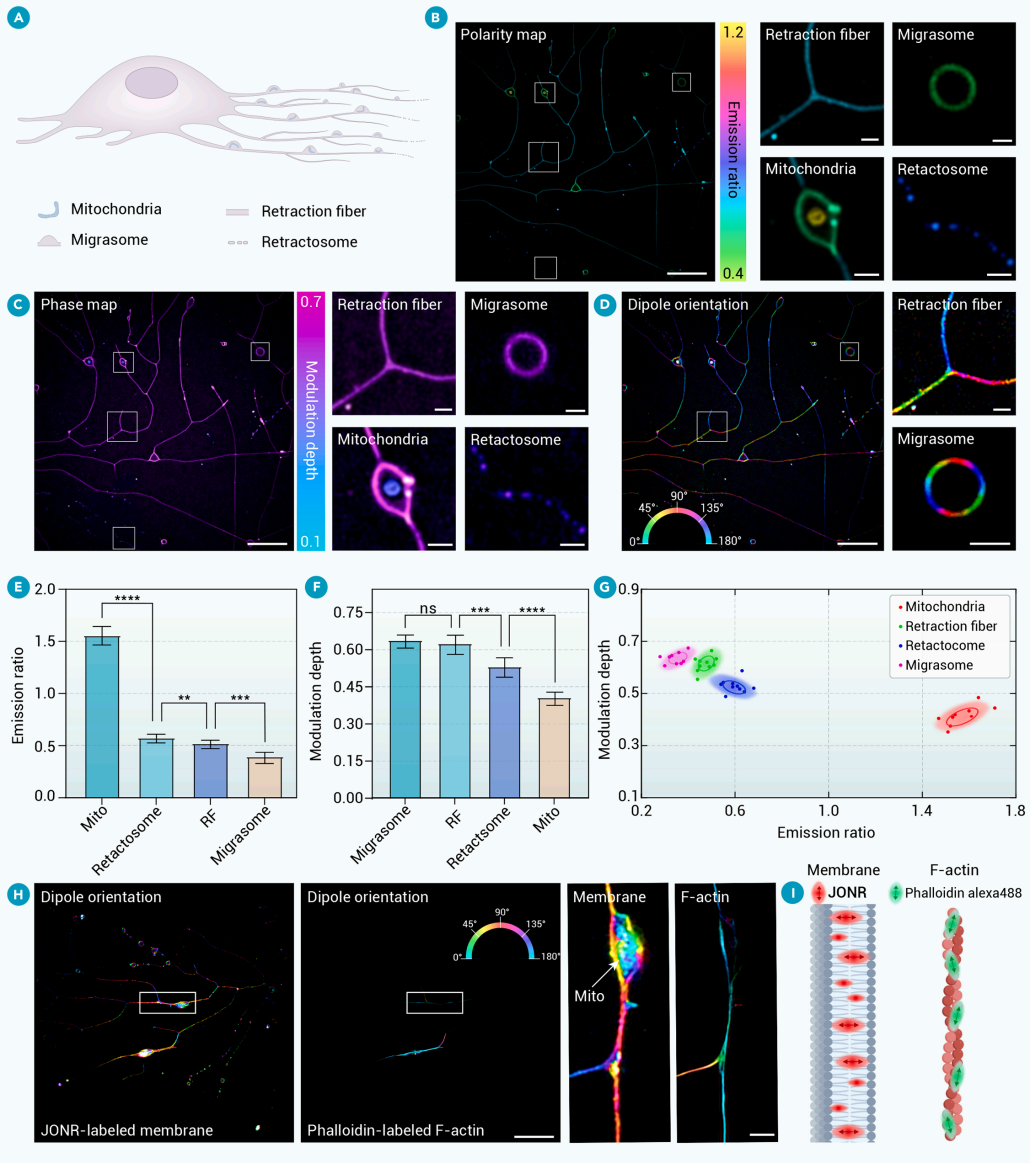
JONR reveals lipid membrane heterogeneity of migration-associated subcellular structures (A) Schematic of subcellular structures closely associated with cell migration, including retraction fibers, migrasomes, mitochondria, and retractosomes. (B) Polarity map of migration-associated structures (left) and magnified view (right). The color scale denotes the emission ratio range, indicating lipid polarity variations across retraction fibers, migrasomes, mitochondria, and retractosomes. (C) Phase map of migration-associated structures (left) and magnified view (right). The color scale reflects the modulation depth range, highlighting local variations in lipid packing. (D) Dipole orientation map of migration-associated structures (left) and magnified view (right). Hue represents the orientation of the fluorescence dipole. JONR-stained retraction fibers and migrasomes exhibit highly ordered dipole alignment. (E) Quantification of emission ratios from migration-associated structures shown in (B). (F) Quantification of modulation depths from structures shown in (C). (G) Optical fingerprint plot classifying migration-associated subcellular structures based on emission ratio and modulation depth. Ellipses denote standard deviation (σ) and 2σ confidence boundaries. (H) Dipole orientation maps of migration-associated membrane structures stained with JONR and actin filaments stained with phalloidin-Alexa 488 (left). An enlarged view shows mitochondria located within migrasomes during mitocytosis (right). (I) Schematic of dipole orientations of JONR and phalloidin-Alexa 488 in the membrane and actin filaments, respectively. Data are shown as mean ± SEM. ∗∗*p* < 0.01, ∗∗∗*p* < 0.001, ∗∗∗∗*p* < 0.0001 (one-way ANOVA). Scale bars: 1 μm (B, right; C, right; D, right; H, right), 5 μm (B, left; C, left; D, left; H, left).

Migrasomes are a newly identified class of vesicular structures that mediate the release of cellular contents and the transfer of signaling molecules, playing essential roles in physiological processes such as tissue development and immune responses.^42^^,^^43^ During cell migration along the extracellular matrix, retraction fibers are pulled out from the trailing edge of the plasma membrane, upon which swollen structures—migrasomes—form. As migration progresses, some retraction fibers break and generate subcellular structures known as retractosomes, while mature migrasomes detach from the cell surface and are released into the extracellular environment (Figure 5A), facilitating intercellular communication.^44^ To investigate the lipid heterogeneity of subcellular structures during migration, we labeled L929 cells with JONR and employed SPOT imaging to quantify the emission ratio and modulation depth of migrasomes, retraction fibers, and retractosomes. Analysis revealed that the emission ratio progressively increased from migrasomes to retraction fibers and then to retractosomes, suggesting a gradual rise in membrane polarity (Figures 5B and 5E). However, the low sensitivity of NR to subtle variations in lipid composition limits its ability to detect these fine differences in membrane structural organization (Figure S15). Recent studies have reported that SM is crucial for migrasome formation, while CL accumulates during their maturation to stabilize membrane structures.^45^^,^^46^^,^^47^ Despite the association of these lipids with enhanced membrane order, we observed no significant difference in modulation depth between migrasomes and retraction fibers (Figures 5C and 5F). Both structures exhibited similarly high modulation depths, with dipole orientations aligned parallel to the direction of membrane extension (Figure 5D), indicating a high degree of lipid order. The decoupling between emission ratio and modulation depth reveals structural and compositional differences among them, and the superior environmental sensitivity of JONR enables effective capture of these variations. Recent cryoelectron tomography studies further revealed that Tspan7 assembles into right-handed helical structures along retraction fibers, forming a transmembrane scaffold that may introduce lipid order independent of lipid composition, thereby influencing membrane physical properties.^48^ We also detected mitochondria within migrasomes, consistent with the recently proposed process of “mitocytosis,” a migration-dependent mitochondrial quality control mechanism that links mitochondrial homeostasis with cellular motility. Quantitative analysis showed that mitochondria exhibited the highest emission ratios but the lowest modulation depths, reflecting membranes of higher polarity and lower lipid order. Based on these multidimensional measurements, we constructed lipid fingerprint profiles for different subcellular structures (Figure 5G), providing a clear visualization of their membrane property differences.

Moreover, to investigate whether the cytoskeleton is spatially associated with migration-related membrane structures, we labeled F-actin with phalloidin-Alexa 488 and performed dipole orientation mapping. We observed the presence of actin filaments adjacent to migrasomes containing mitochondria (Figure 5H). Notably, the orientation rings derived from JONR and F-actin labeling differed by approximately 90° (Figure 5I), indicating that the fluorescence dipoles of the two labels are orthogonal. These findings highlight the capacity of JONR to resolve lipid properties across migration-associated subcellular structures with high spatial and orientational precision. By linking membrane polarity, order, and dipole orientation, this approach offers a powerful framework to dissect the biophysical underpinnings of membrane remodeling during cellular migration.

## Conclusion

Advances in imaging technology and membrane biology have spurred the need to acquire complex biophysical information of membranes, extending beyond spatiotemporal resolution. To achieve precise organelle identification for interaction studies or to resolve membrane heterogeneity at the super-resolution level, the design of fluorescent probes with superior environmental sensitivity is crucial. In this work, we developed JONR, a novel far-red-emitting NR derivative incorporating a rigid TDAP auxochrome, which exhibits significantly enhanced solvatochromism, a large Stokes shift, and improved fluorogenicity while maintaining high QYs in hydrophobic environments. JONR demonstrates pronounced responsiveness to membrane microenvironmental changes through multiple photophysical parameters, including emission spectra, polarization characteristics, and fluorescence lifetime. By implementing SPOT technology, we showcase its exceptional capability to identify subcellular organelles and monitor their dynamic interactions. JONR enables simultaneous mapping of membrane polarity and lipid order, revealing structural heterogeneity across distinct membrane domains of migrasomes. This breakthrough will provide novel mechanistic insights into organelle formation and maturation processes. This work establishes a powerful template for synergistic integration of a novel imaging system with the smart molecular probe in cell biology research. We believe that JONR provides an elegant solution for multidimensional investigation of subcellular membrane properties and enables analysis of the real-time organelle interactome.

## Resource availability

### Materials availability

All stable reagents generated in this study are available from the lead contact.

### Data and code availability

Data presented in this study are provided in supplementary tables. Data are available from the corresponding authors upon request.

## Funding and acknowledgments

This project was supported by funds from the Major Basic Research Project of the Natural Science Foundation of Shandong Province (ZR2024ZD27 to P.X.), the 10.13039/501100012166National Key R&D Program of China (2021YFF0502904 to Z.C. and 2022YFC3401100 to P.X.), the 10.13039/501100009592Beijing Municipal Science & Technology Commission (project Z221100003422013 to Z.C.), the 10.13039/501100021187Beijing National Laboratory for Molecular Sciences (BNLMS202407 to Z.C.), the 10.13039/501100001809National Natural Science Foundation of China (W2412031 to Z.C.). We thank the Metabolic Mass Spectrometry Platform at the IMM, the analytical instrumentation center of Peking University, the NMR facility of the National Center for Protein Sciences at Peking University, and the National Center for Protein Sciences at Peking University. The funders had no role in study design, data collection and analysis, decision to publish, or preparation of the manuscript.

## Author contributions

M. Liu, Y. Fu, M. Li, Z. Chen, and P. Xi conceived the idea. M. Liu conducted molecular synthesis, GUV preparation, and spectroscopic measurements. Y. Fu conducted the setup of microscopy and *in vitro* imaging and image analysis. X. Li conducted L929 cell cultivation and provided suggestions. H. Zhang and X. Lu supported molecular synthesis. Y. Ma conducted the computational data. H. Zheng and Y. Zhang conducted neuron cell isolation and cultivation. Z. Chen and P. Xi supervised the project. M. Liu, Y. Fu, Z. Chen, and P. Xi wrote the manuscript with inputs from all authors.

## Declaration of interests

The authors declare no competing interests.

## Supplemental information

Document S1. Figures S1–S15 and Table S1

Document S2. Article plus supplemental information

## References

[1] Harayama T., Riezman H. (2018). Understanding the diversity of membrane lipid composition. Nat. Rev. Mol. Cell Biol..

[2] Morgan P.K., Pernes G., Huynh K. (2024). A lipid atlas of human and mouse immune cells provides insights into ferroptosis susceptibility. Nat. Cell Biol..

[3] Dietrich C., Bagatolli L.A., Volovyk Z.N. (2001). Lipid rafts reconstituted in model membranes. Biophys. J..

[4] Veatch S.L., Keller S.L. (2002). Organization in Lipid Membranes Containing Cholesterol. Phys. Rev. Lett..

[5] Baumgart T., Hess S.T., Webb W.W. (2003). Imaging coexisting fluid domains in biomembrane models coupling curvature and line tension. Nature.

[6] Basu Ball W., Neff J.K., Gohil V.M. (2018). The role of nonbilayer phospholipids in mitochondrial structure and function. FEBS Lett..

[7] Klymchenko A.S., Kreder R. (2014). Fluorescent probes for lipid rafts: from model membranes to living cells. Chem. Biol..

[8] Klymchenko A.S. (2017). Solvatochromic and Fluorogenic Dyes as Environment-Sensitive Probes: Design and Biological Applications. Acc. Chem. Res..

[9] Klymchenko A.S. (2023). Fluorescent Probes for Lipid Membranes: From the Cell Surface to Organelles. Acc. Chem. Res..

[10] Martinez V., Henary M. (2016). Nile red and nile blue: applications and syntheses of structural analogues. Chem. Eur J..

[11] Reichardt C. (1994). Solvatochromic dyes as solvent polarity indicators. Chem. Rev..

[12] Jin L., Millard A.C., Wuskell J.P. (2006). Characterization and application of a new optical probe for membrane lipid domains. Biophys. J..

[13] Greenspan P., Fowler S.D. (1985). Spectrofluorometric studies of the lipid probe, nile red. J. Lipid Res..

[14] Greenspan P., Mayer E.P., Fowler S.D. (1985). Nile red: a selective fluorescent stain for intracellular lipid droplets. J. Cell Biol..

[15] Mishra R., Sjölander D., Hammarström P. (2011). Spectroscopic characterization of diverse amyloid fibrils in vitro by the fluorescent dye Nile red. Mol. Biosyst..

[16] Prifti E., Reymond L., Umebayashi M. (2014). A fluorogenic probe for SNAP-tagged plasma membrane proteins based on the solvatochromic molecule Nile red. ACS Chem. Biol..

[17] Sundukova M., Prifti E., Bucci A. (2019). A chemogenetic approach for the optical monitoring of voltage in neurons. Angew. Chem. Int. Ed..

[18] Hanser F., Marsol C., Valencia C. (2021). Nile red-based GPCR ligands as ultrasensitive probes of the local lipid microenvironment of the receptor. ACS Chem. Biol..

[19] Kucherak O.A., Oncul S., Darwich Z. (2010). Switchable nile red-based probe for cholesterol and lipid order at the outer leaflet of biomembranes. J. Am. Chem. Soc..

[20] Danylchuk D.I., Moon S., Xu K. (2019). Switchable solvatochromic probes for live-cell super-resolution imaging of plasma membrane organization. Angew. Chem. Int. Ed..

[21] Danylchuk D.I., Jouard P.-H., Klymchenko A.S. (2021). Targeted solvatochromic fluorescent probes for imaging lipid order in organelles under oxidative and mechanical stress. J. Am. Chem. Soc..

[22] Moon S., Yan R., Kenny S.J. (2017). Spectrally Resolved, Functional Super-Resolution Microscopy Reveals Nanoscale Compositional Heterogeneity in Live-Cell Membranes. J. Am. Chem. Soc..

[23] Zhanghao K., Liu W., Li M. (2020). High-dimensional super-resolution imaging reveals heterogeneity and dynamics of subcellular lipid membranes. Nat. Commun..

[24] Zhanghao K., Li M., Chen X. (2025). Fast segmentation and multiplexing imaging of organelles in live cells. Nat. Commun..

[25] Fu Y., Hou Y., Liang Q. (2025). Triangle-beam interference structured illumination microscopy. Nat. Photonics.

[26] Zhong S., Qiao L., Ge X. (2024). Three-dimensional dipole orientation mapping with high temporal-spatial resolution using polarization modulation. PhotoniX.

[27] Tan Z., Hsiung C.-H., Feng J. (2025). Time-resolved fluorescent proteins expand fluorescent microscopy in temporal and spectral domains. Cell.

[28] Jose J., Burgess K. (2006). Benzophenoxazine-based fluorescent dyes for labeling biomolecules. Tetrahedron.

[29] Lauritsen L., Szomek M., Hornum M. (2024). Ratiometric fluorescence nanoscopy and lifetime imaging of novel Nile red analogs for analysis of membrane packing in living cells. Sci. Rep..

[30] Hornum M., Mulberg M.W., Szomek M. (2021). Substituted 9-diethylaminobenzo [a] phenoxazin-5-ones (Nile red Analogues): Synthesis and photophysical properties. J. Org. Chem..

[31] Prioli S., Reinholdt P., Hornum M. (2019). Rational design of Nile red analogs for sensing in membranes. J. Phys. Chem. B.

[32] Long J., Wang Y.M., Matsuura T. (1999). Synthesis and fluorescence properties of novel benzo [a] phenoxazin-5-one derivatives. J. Heterocycl. Chem..

[33] Jose J., Burgess K. (2006). Syntheses and properties of water-soluble Nile red derivatives. J. Org. Chem..

[34] Sherman D.B., Pitner J.B., Ambroise A. (2006). Synthesis of thiol-reactive, long-wavelength fluorescent phenoxazine derivatives for biosensor applications. Bioconjug. Chem..

[35] Dong Y., Zou Y., Jia X. (2023). An acidic medium-compatible deep-near-infrared dye for in vivo imaging. Smart Mol..

[36] Liu Z., Lu T., Chen Q. (2020). An sp-hybridized all-carboatomic ring, cyclo [18] carbon: Electronic structure, electronic spectrum, and optical nonlinearity. Carbon.

[37] Guo Y., Li D., Zhang S. (2018). Visualizing Intracellular Organelle and Cytoskeletal Interactions at Nanoscale Resolution on Millisecond Timescales. Cell.

[38] Sot J., Gartzia-Rivero L., Bañuelos J. (2022). Liquid-crystalline, liquid-ordered, rippled and gel lipid bilayer phases as observed with nile red fluorescence. J. Mol. Liq..

[39] Zhanghao K., Chen X., Liu W. (2019). Super-resolution imaging of fluorescent dipoles via polarized structured illumination microscopy. Nat. Commun..

[40] Igarashi M. (2019). Molecular basis of the functions of the mammalian neuronal growth cone revealed using new methods. Proc. Jpn. Acad. Ser. B Phys. Biol. Sci..

[41] Lowery L.A., Van Vactor D. (2009). The trip of the tip: understanding the growth cone machinery. Nat. Rev. Mol. Cell Biol..

[42] Ma L., Li Y., Peng J. (2015). Discovery of the migrasome, an organelle mediating release of cytoplasmic contents during cell migration. Cell Res..

[43] Jiao H., Jiang D., Hu X. (2021). Mitocytosis, a migrasome-mediated mitochondrial quality-control process. Cell.

[44] Wang Y., Gao S., Liu Y. (2022). Retractosomes: small extracellular vesicles generated from broken-off retraction fibers. Cell Res..

[45] Liang H., Ma X., Zhang Y. (2023). The formation of migrasomes is initiated by the assembly of sphingomyelin synthase 2 foci at the leading edge of migrating cells. Nat. Cell Biol..

[46] Huang Y., Zucker B., Zhang S. (2019). Migrasome formation is mediated by assembly of micron-scale tetraspanin macrodomains. Nat. Cell Biol..

[47] Dharan R., Huang Y., Cheppali S.K. (2023). Tetraspanin 4 stabilizes membrane swellings and facilitates their maturation into migrasomes. Nat. Commun..

[48] Wang D., Jia X., Zhang Q. (2024). Polymerization of Tspan7 into Helical Transmembrane Skeletons for Membrane Tether Stabilization. bioRxiv.

