# Supplementary material for: Far-red-emitting Nile red with boosted solvatochromism enables multidimensional imaging of membrane heterogeneity and dynamics
Source: Innovation (Camb). 2026 Feb 24;7(8):101319. doi: 10.1016/j.xinn.2026.101319 (PMC13457185; doi:10.1016/j.xinn.2026.101319)
Supplement: Supplementary file 1 [file mmc1.pdf]

The Innovation, Volume *7*

Supplemental Information

Far-red-emitting Nile red with boosted solvatochromism enables multi-dimensional imaging of membrane heterogeneity and dynamics

Mingqiao Liu, Yunzhe Fu, Xiaopeng Li, Haolin Zhang, Yumiao Ma, Meiqi Li, Chunyan Shan, Hongxi Zheng, Xiaoyi Lu, Yan Zhang, Li Yu, Peng Xi, and Zhixing Chen

Supporting Information

Table of Contents

Supplementary Figures…………………………………………………………….3

Experimental Details…………………………………………………………...15

Microscopy Setup and Imaging Protocol………………………………………24

Computational Data…………………………………………………………….27

Reference………………………………………………………………………….34

NMR spectra……………………………………………………………………35

Supplementary Figures

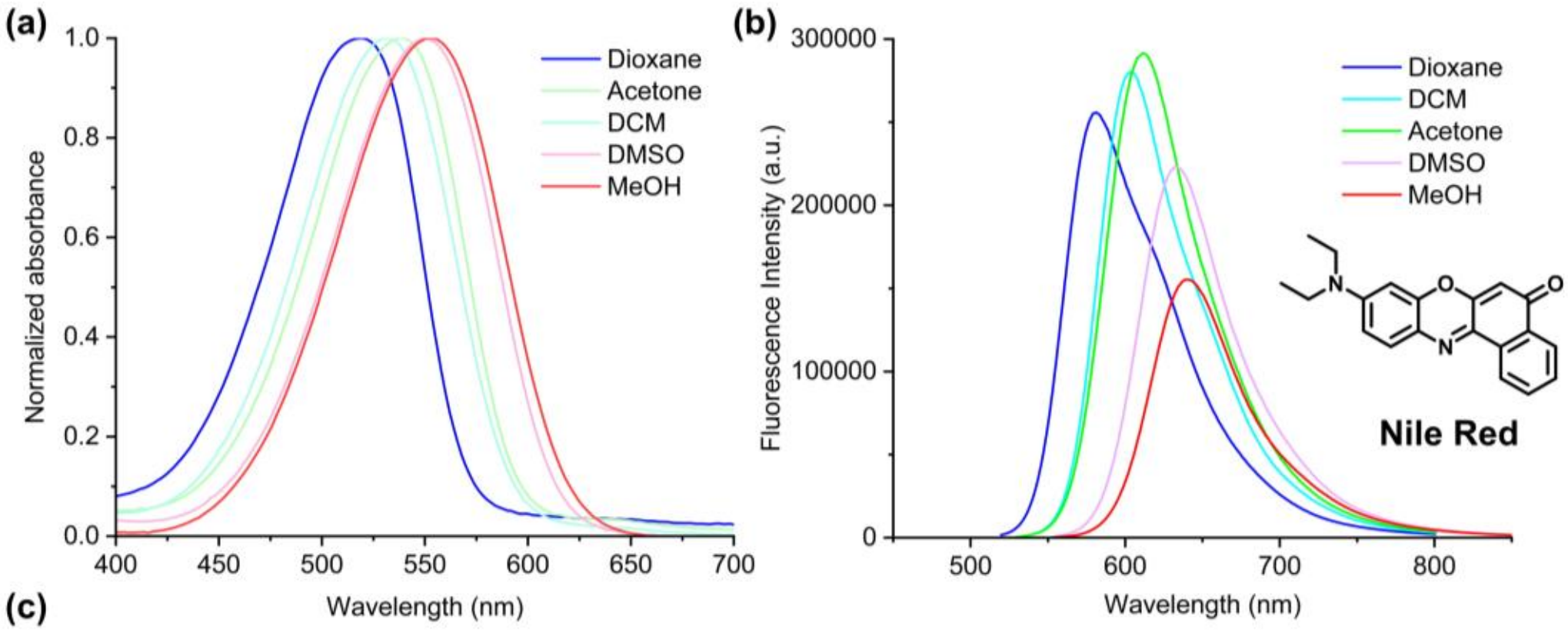

(c)

Solvent	λ^{abs}_{max} (nm)	λ^{em}_{max} (nm)	Solvatochromism (nm)	Stokes shift (nm)	Φ_f	ε [M^{-1} cm^{-1}]
Dioxane	520	581		61	0.82	29500
DCM	538	604		66	0.84	31000
Acetone	532	611	**59**	79	0.82	28500
DMSO	550	632		82	0.74	33000
MeOH	552	640		88	0.42	32600

Figure S1. Characterizations of Nile Red *in vitro*. (a). Normalized absorbance spectra of Nile Red (2 μM); (b). Emission spectra of Nile Red (2 μM); (c). Photophysical properties of Nile Red. Solvatochromism was observed by subtracting the maximum emission wavelength of Nile Red in methanol from that in dioxane.

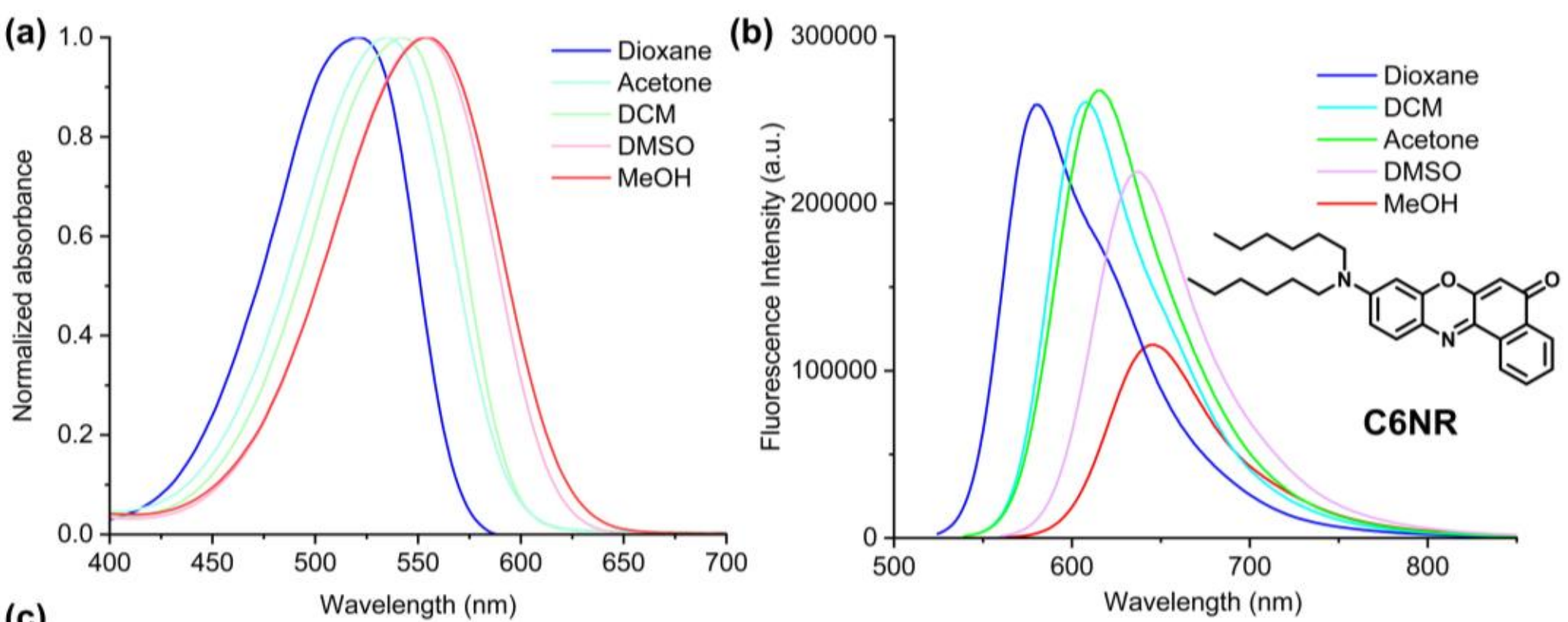

(c)

Solvent	λ^{abs}_{max} (nm)	λ^{em}_{max} (nm)	Solvatochromism (nm)	Stokes shift (nm)	Φ_f	ε [M^{-1} cm^{-1}]
Dioxane	520	580		60	0.81	30000
DCM	542	607		65	0.78	30800
Acetone	536	615	**64**	79	0.76	29000
DMSO	552	638		86	0.74	32000
MeOH	554	644		90	0.32	32800

Figure S2. Characterizations of C6NR *in vitro*. (a). Normalized absorbance spectra of C6NR (2 μM); (b). Emission spectra of C6NR (2 μM); (c). Photophysical properties of C6NR. Solvatochromism was observed by subtracting the maximum emission wavelength of C6NR in methanol from that in dioxane.

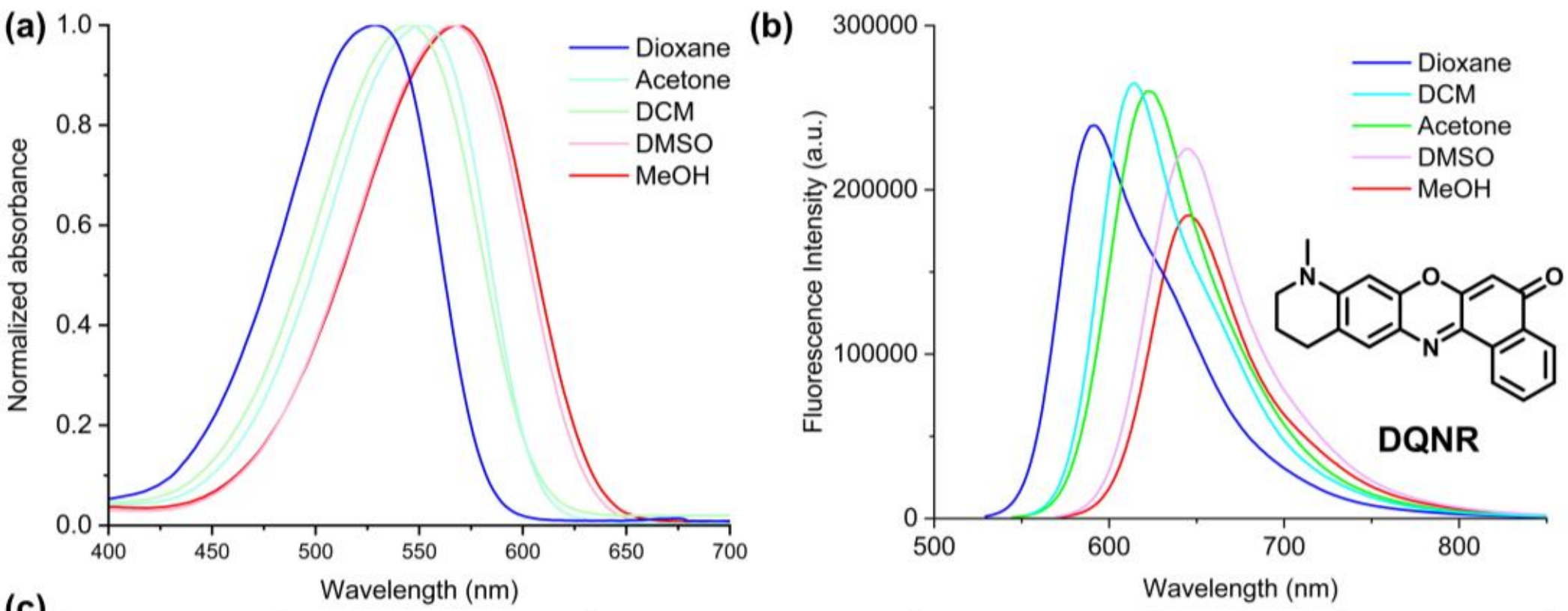

(c)

Solvent	λ^{abs}_{max} (nm)	λ^{em}_{max} (nm)	Solvatochromism (nm)	Stokes shift (nm)	Φ_f	ε [M^{-1} cm^{-1}]
Dioxane	528	591		63	0.74	28400
DCM	552	615		63	0.77	31400
Acetone	546	622	**55**	76	0.71	30500
DMSO	566	644		78	0.72	32000
MeOH	568	646		78	0.47	29000

Figure S3. Characterizations of DQNR *in vitro*. (a). Normalized absorbance spectra of DQNR (2 μM); (b). Emission spectra of DQNR (2 μM); (c). Photophysical properties of DQNR. Solvatochromism was observed by subtracting the maximum emission wavelength of DQNR in methanol from that in dioxane.

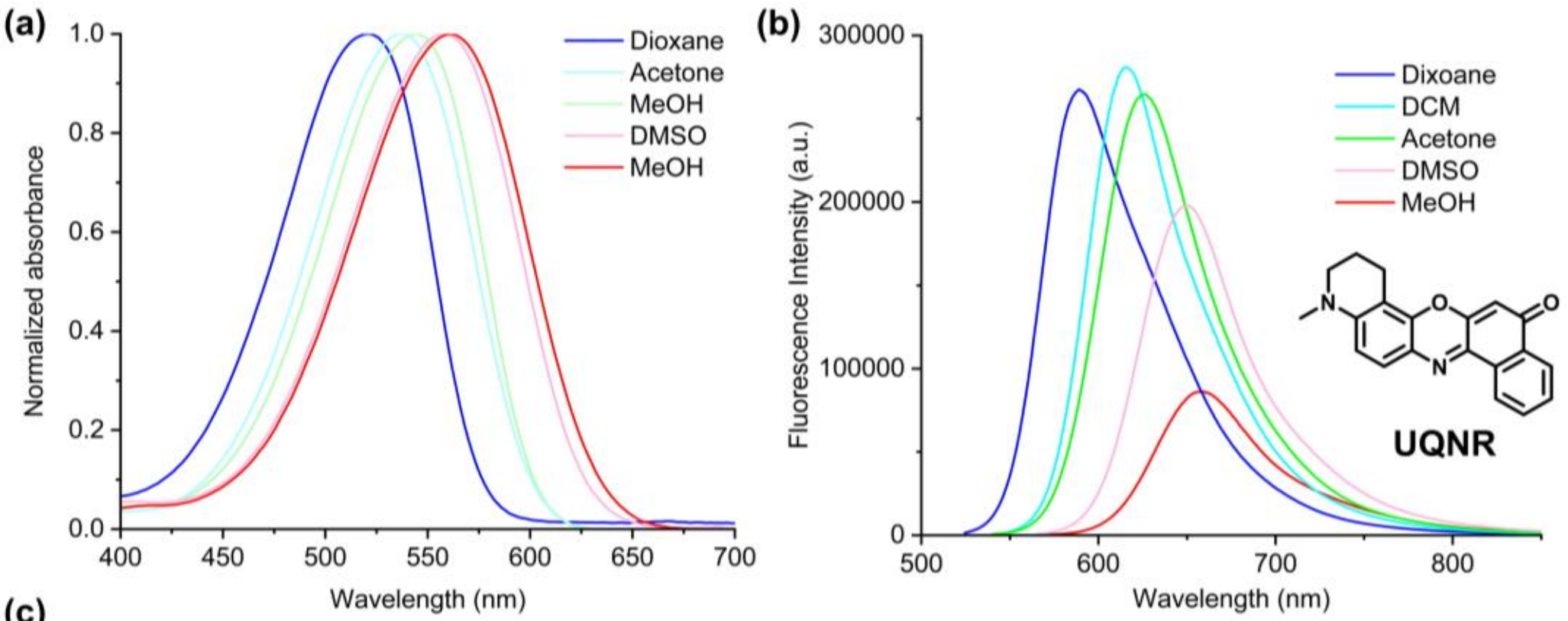

(c)

Solvent	λ^{abs}_{max} (nm)	λ^{em}_{max} (nm)	Solvatochromism (nm)	Stokes shift (nm)	Φ_f	ε [M^{-1} cm^{-1}]
Dioxane	522	589		67	0.82	29600
DCM	544	615		71	0.83	31000
Acetone	538	626	**65**	88	0.75	32400
DMSO	558	650		92	0.67	31700
MeOH	562	654		92	0.25	32300

Figure S4. Characterizations of UQNR *in vitro*. (a). Normalized absorbance spectra of UQNR (2 μM); (b). Emission spectra of UQNR (2 μM); (c). Photophysical properties of UQNR. Solvatochromism was observed by subtracting the maximum emission wavelength of UQNR in methanol from that in dioxane.

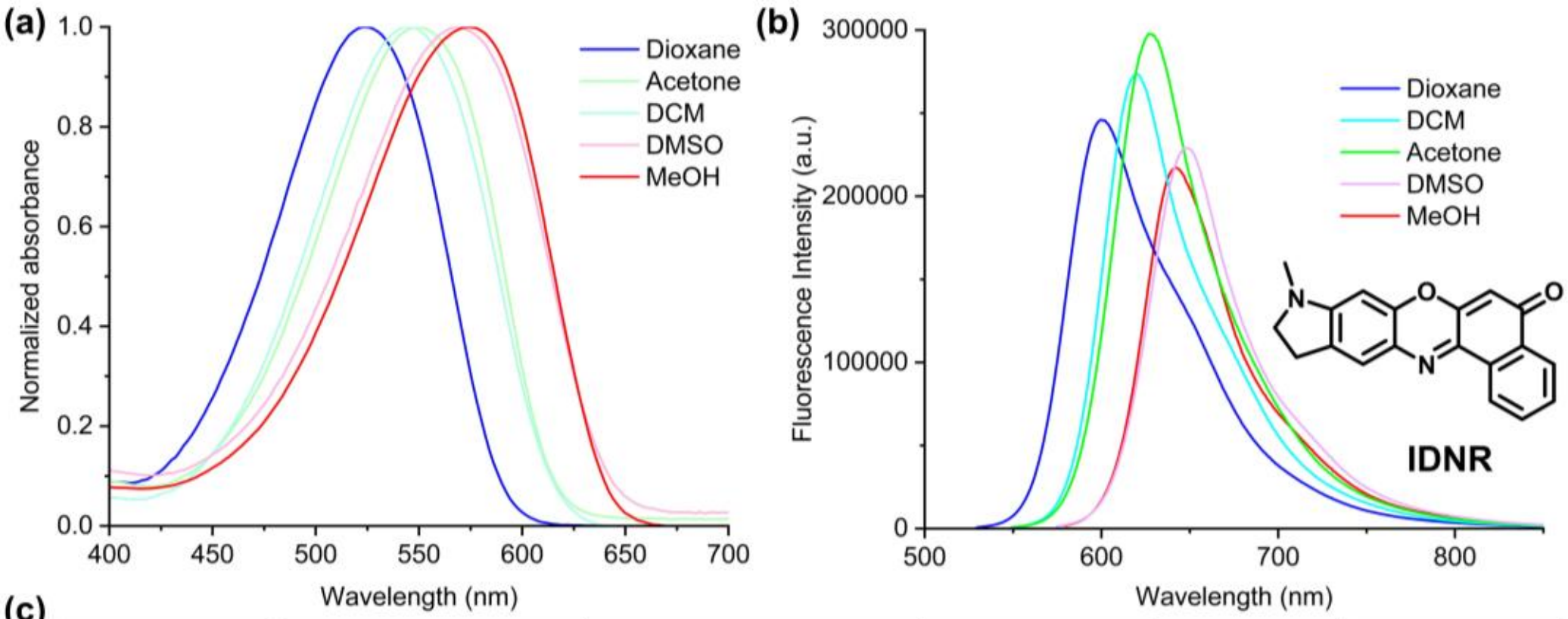

Solvent	λ^{abs}_{max} (nm)	λ^{em}_{max} (nm)	Solvatochromism (nm)	Stokes shift (nm)	Φ_f	ε [M^{-1} cm^{-1}]
Dioxane	524	600		76	0.76	28800
DCM	548	620		72	0.77	30000
Acetone	544	628	**42**	84	0.77	30800
DMSO	570	647		77	0.70	31000
MeOH	574	642		68	0.52	30600

Figure S5. Characterizations of IDNR *in vitro*. (a). Normalized absorbance of IDNR (2 μM); (b). Emission spectra of IDNR (2 μM); (c). Photophysical properties of IDNR. Solvatochromism was observed by subtracting the maximum emission wavelength of IDNR in methanol from that in dioxane.

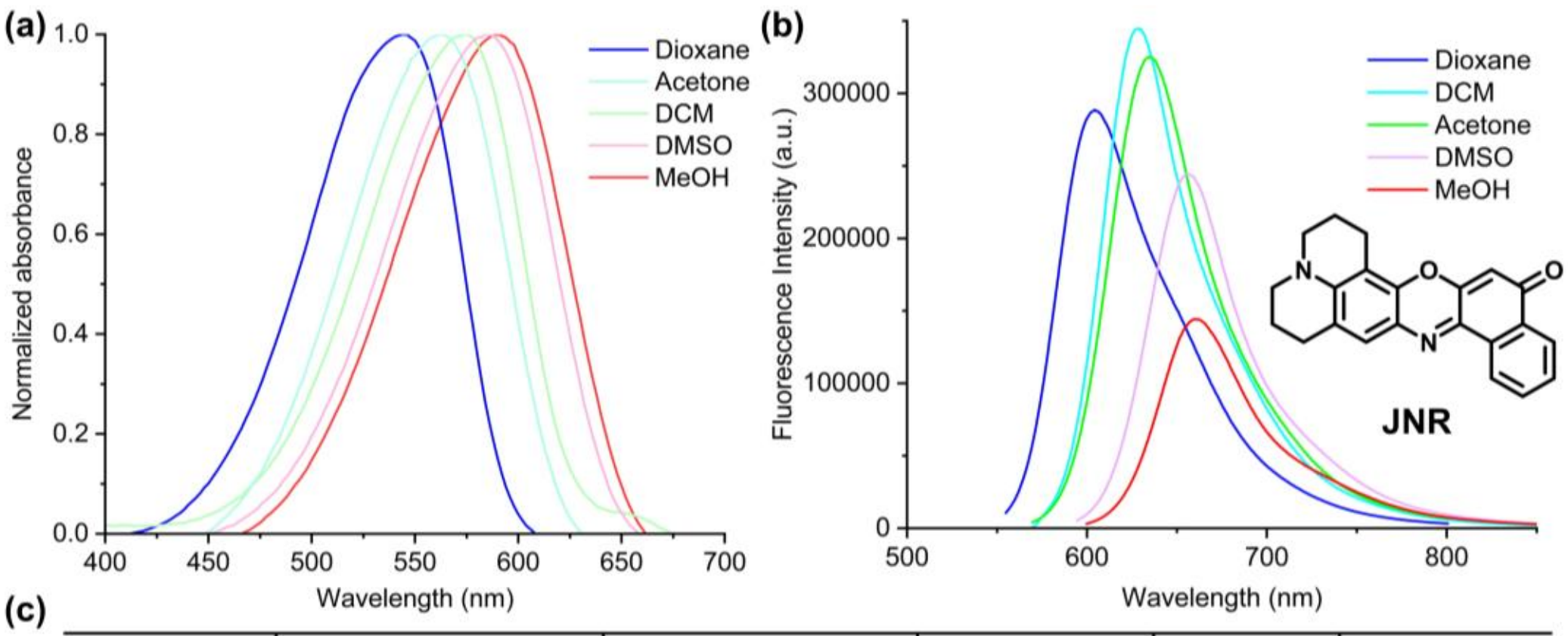

Solvent	λ^{abs}_{max} (nm)	λ^{em}_{max} (nm)	Solvatochromism (nm)	Stokes shift (nm)	Φ_f	ε [M^{-1} cm^{-1}]
Dioxane	544	604		60	0.85	28600
DCM	574	629		55	0.89	30000
Acetone	562	635	**57**	73	0.84	33000
DMSO	584	656		72	0.76	31800
MeOH	590	661		71	0.36	32400

Figure S6. Characterizations of JNR *in vitro.* (a). Normalized absorbance spectra of JNR (2 μM); (b). Emission spectra of JNR (2 μM); (c). Photophysical properties of JNR. Solvatochromism was observed by subtracting the maximum emission wavelength of JNR in methanol from that in dioxane.

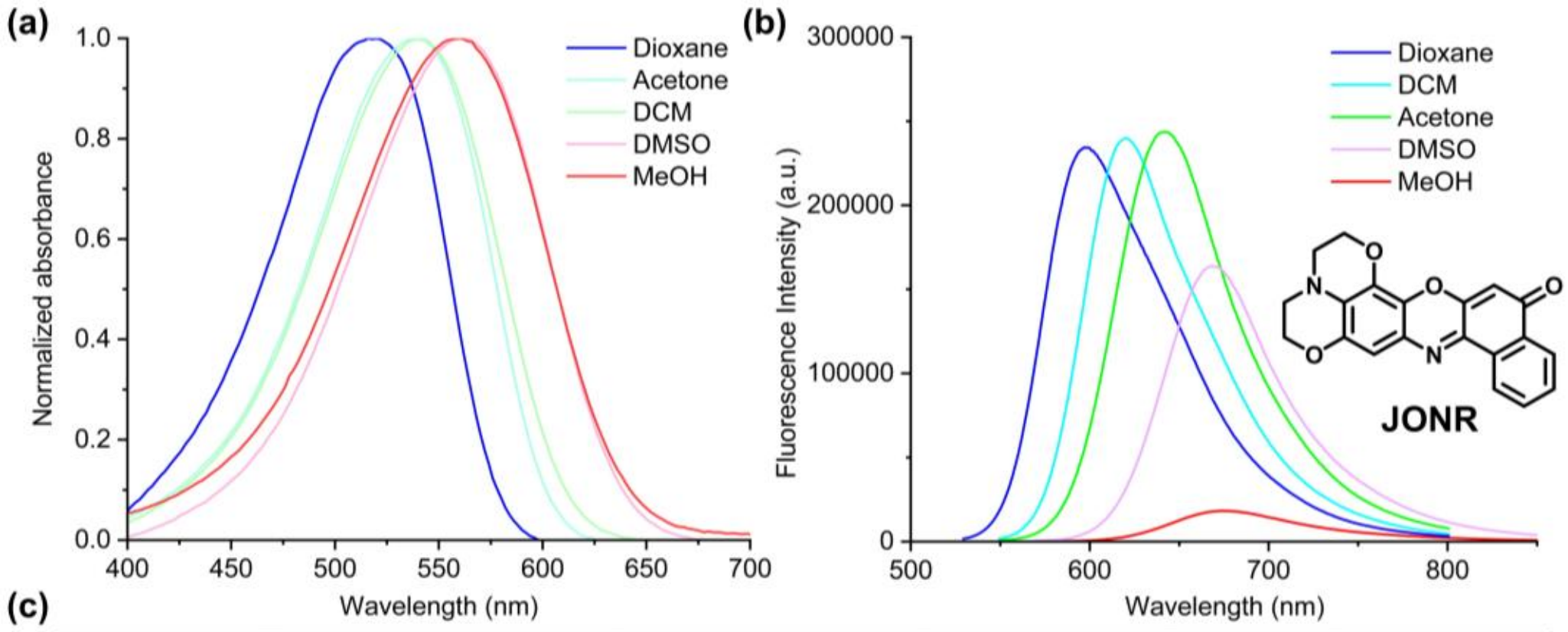

(c)

Solvent	λ^{abs}_{max} (nm)	λ^{em}_{max} (nm)	Solvatochromism (nm)	Stokes shift (nm)	Φ_f	ε [M^{-1} cm^{-1}]
Dioxane	516	599		83	0.75	32800
DCM	538	620		82	0.73	33000
Acetone	538	641	**75**	103	0.69	29600
DMSO	560	667		107	0.56	33200
MeOH	560	674		114	0.055	28400

Figure S7. Characterizations of JONR *in vitro*. (a). Normalized absorbance spectra of JONR (2 μM); (b). Emission spectra of JONR (2 μM); (c). Photophysical properties of JONR. Solvatochromism was observed by subtracting the maximum emission wavelength of JONR in methanol from that in dioxane.

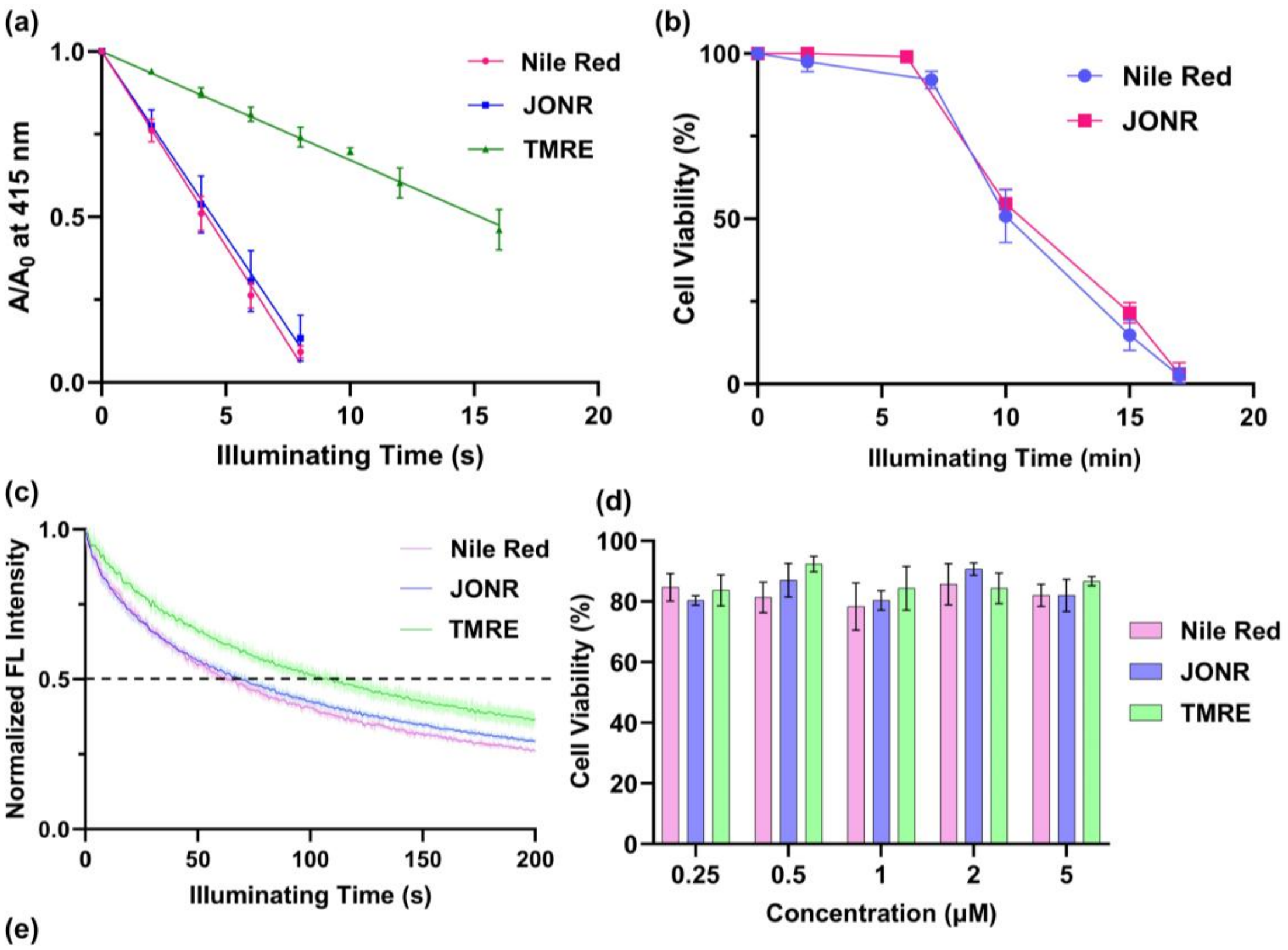

(e)

Compound	Absolute Singlet Oxygen Yield($\Phi_{\Delta}(^1O_2)$) Units: X 10^{-4} (Mean ± SD)	Half-lethal Light Dose (min)	Half-life of Photobleaching (s)
Nile Red	440 ± 6	~10	62
JONR	408 ± 10	~11	71
TMRE	120 ± 7		105

Figure S8. Cytotoxicity, phototoxicity and photostability of JONR. (a) *In vitro* singlet oxygen generation experiments of JONR and Nile Red. The maximum absorption of DPBF at 415 nm was measured under continuous irradiation with a 520-530 nm LED lamp in the presence of each dye (absorbance at 525 nm = 0.1; concentrations: JONR, 3.6 μM; Nile Red, 3.7 μM) in air-saturated MeOH. TMRE in MeOH (Φ_{Δ}= 0.012) in MeOH was selected as a standard. (b) Live-cell phototoxicity measurements of Nile Red and JONR in HeLa cells. Cell apoptosis of > 500 cells after 568 nm LED light illumination (2.6 W/cm^2) was examined at each time point of the three independent experiments. (c) Photobleaching curve of JONR, Nile Red and TMRE in polymethyl methacrylate (PMMA) film recorded under continuous 561 nm laser scanning of a confocal microscope. (d) Cytotoxicity assay of HeLa cells stained with JONR, Nile Red and TMRE. Cytotoxicity of HeLa cells treated with JONR, Nile Red and TMRE in different staining concentrations (0.25, 0.5, 1, 2, 5 μM) for 2 hours. (e) Table of singlet oxygen yield, phototoxicity and photostability of JONR, Nile Red and TMRE. Each experiment was independently repeated at least three times. Data are shown as mean ± SEM.

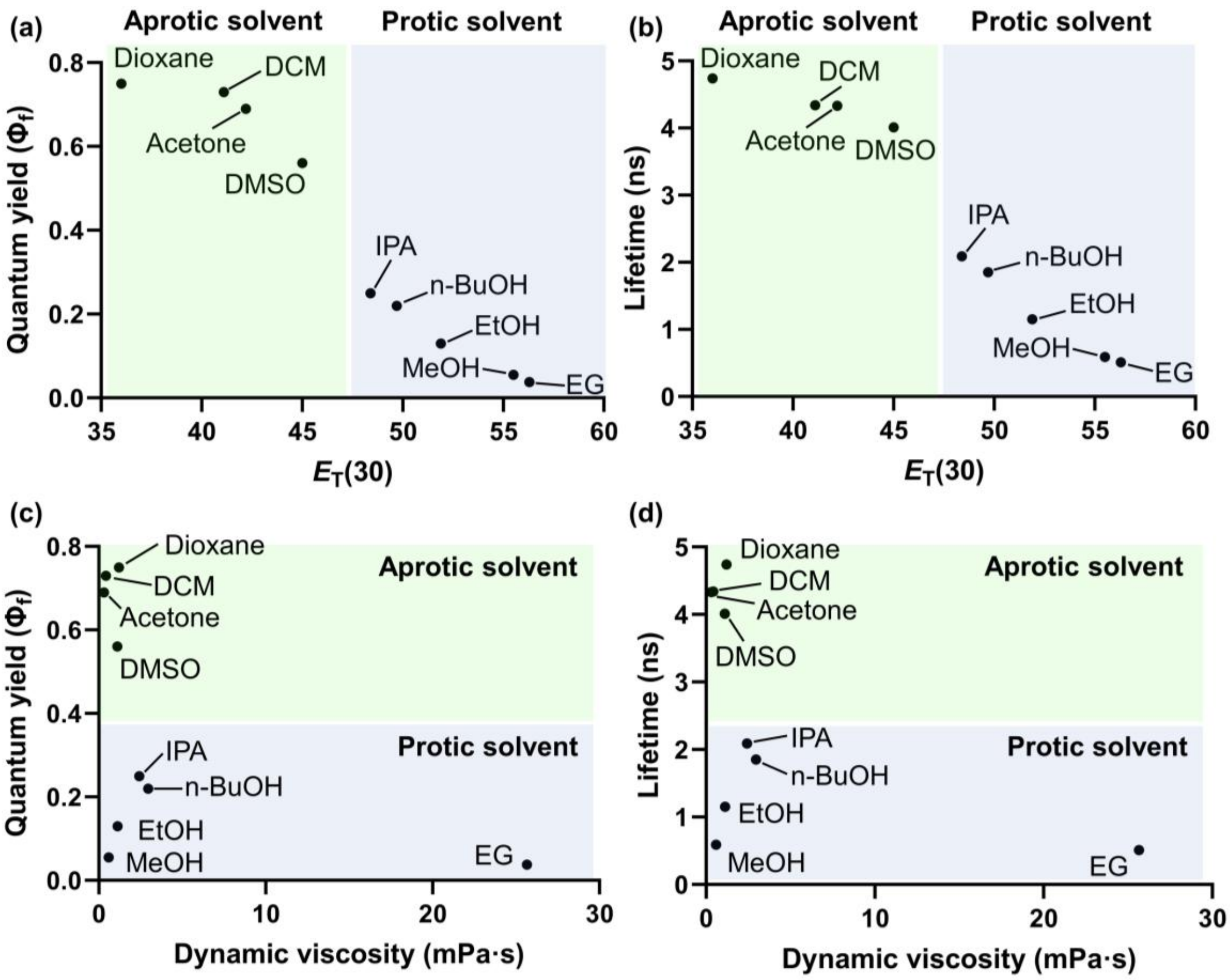

(e)

Solvent	$E_T(30)$	η (mPa·s, 25℃)	pKa (25℃)	λ^{em}_{max} (nm)	Φ_f	Lifetime (ns)
**Dioxane**	36.0	1.2	N.A.	599	0.75	4.74
**DCM**	41.1	0.43	N.A.	620	0.73	4.34
**Acetone**	42.2	0.316	N.A.	641	0.69	4.33
**DMSO**	45	1.1	N.A.	667	0.56	4.01
**IPA**	48.4	0.472	16.5	660	0.25	2.09
**n-BuOH**	49.7	0.594	16.0	663	0.22	1.85
**EtOH**	51.9	2.43	15.9	672	0.13	1.15
**MeOH**	55.5	2.95	15.5	674	0.055	0.59
**EG**	56.3	25.66	14.2	687	0.038	0.51

Figure S9. Relationship between the photophysical properties of JONR and solvent polarity and viscosity. (a) Relationship between the solvent polarity (measured by $E_T(30)$) and the quantum yield of JONR. (b) Relationship between the solvent polarity and the fluorescence lifetime of JONR. (c) Relationship between the solvent viscosity (using dynamic viscosity at 25 °C) and the quantum yield of JONR. (d) Relationship between the solvent viscosity and the fluorescence lifetime of JONR. (e) Table of the polarity, viscosity, pKa of the solvents, and maximum emission wavelength, fluorescence quantum yields, and fluorescence lifetimes of JONR in different solvents.

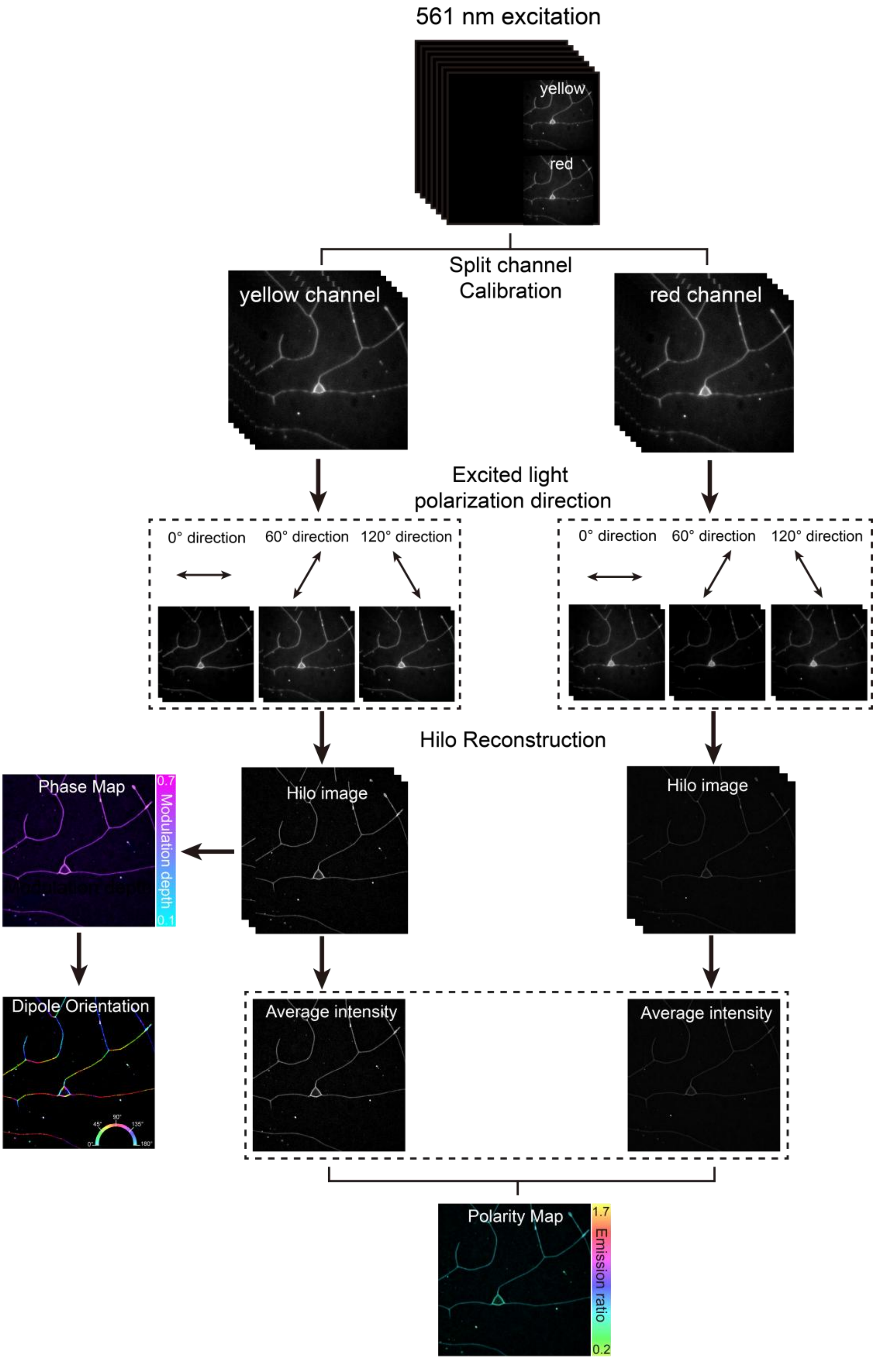

Figure S10. Principle of Spectrum and Polarization Optical Tomography (SPOT). The detailed reconstruction process of SPOT includes the measurement of emission ratio and modulation depth, as well as the mapping of dipole orientation.

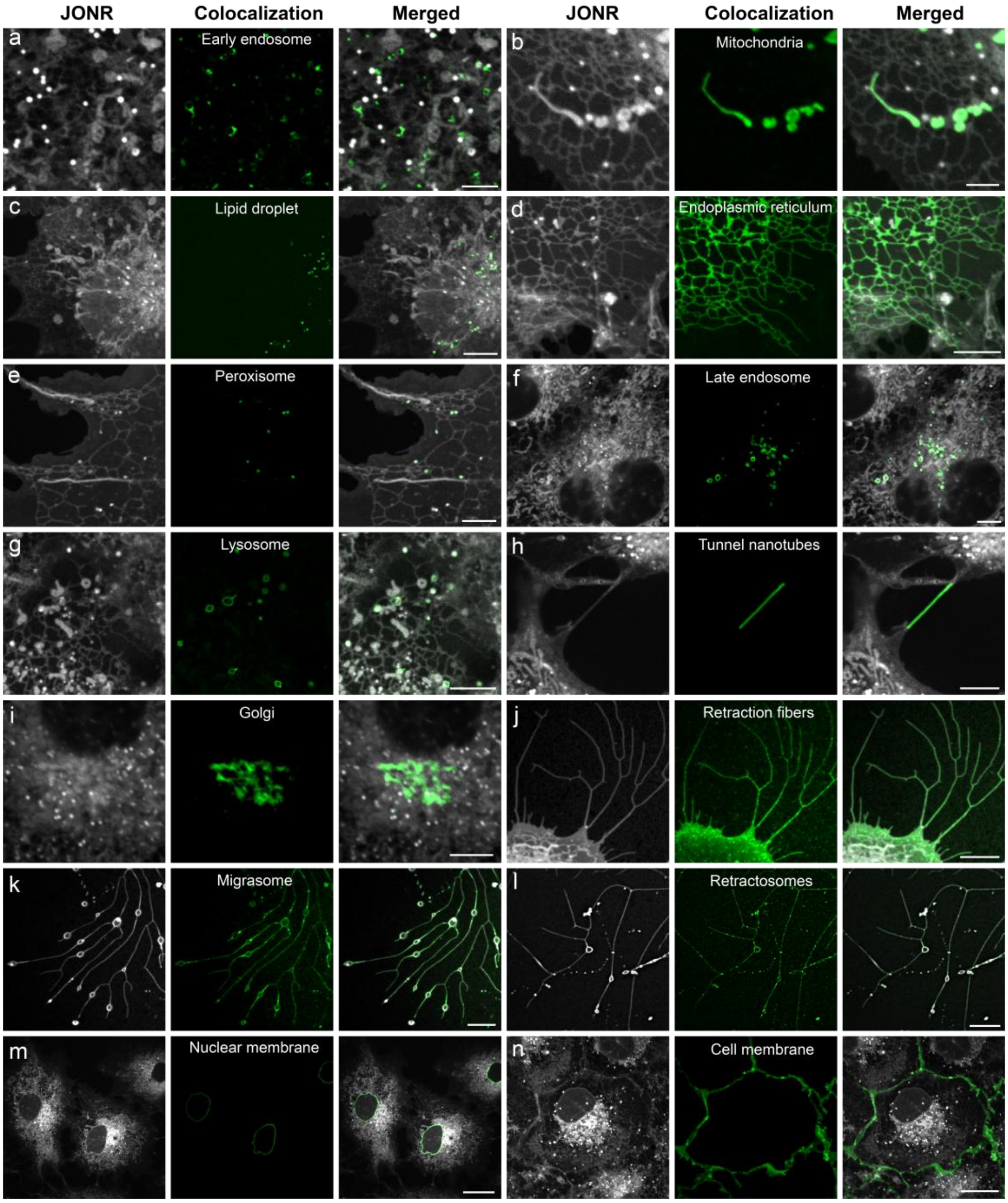

Figure S11. Colocalization of JONR with fluorescent proteins or dyes in live cells. The images in each left column are fluorescent images of JONR in the yellow channel (excitation: 561 nm, emission: 578-614 nm). The images in the middle are the fluorescent images in the green channel (excitation: 488 nm, emission: 505-545 nm). The images on the right are the colocalization of Nile Red signals in both green and yellow emission channels. All the experimental results were repeated at least three times with independently prepared samples. Scale bar: 3 µm (a, b); 5 µm(c-l); 15 µm (m, n).

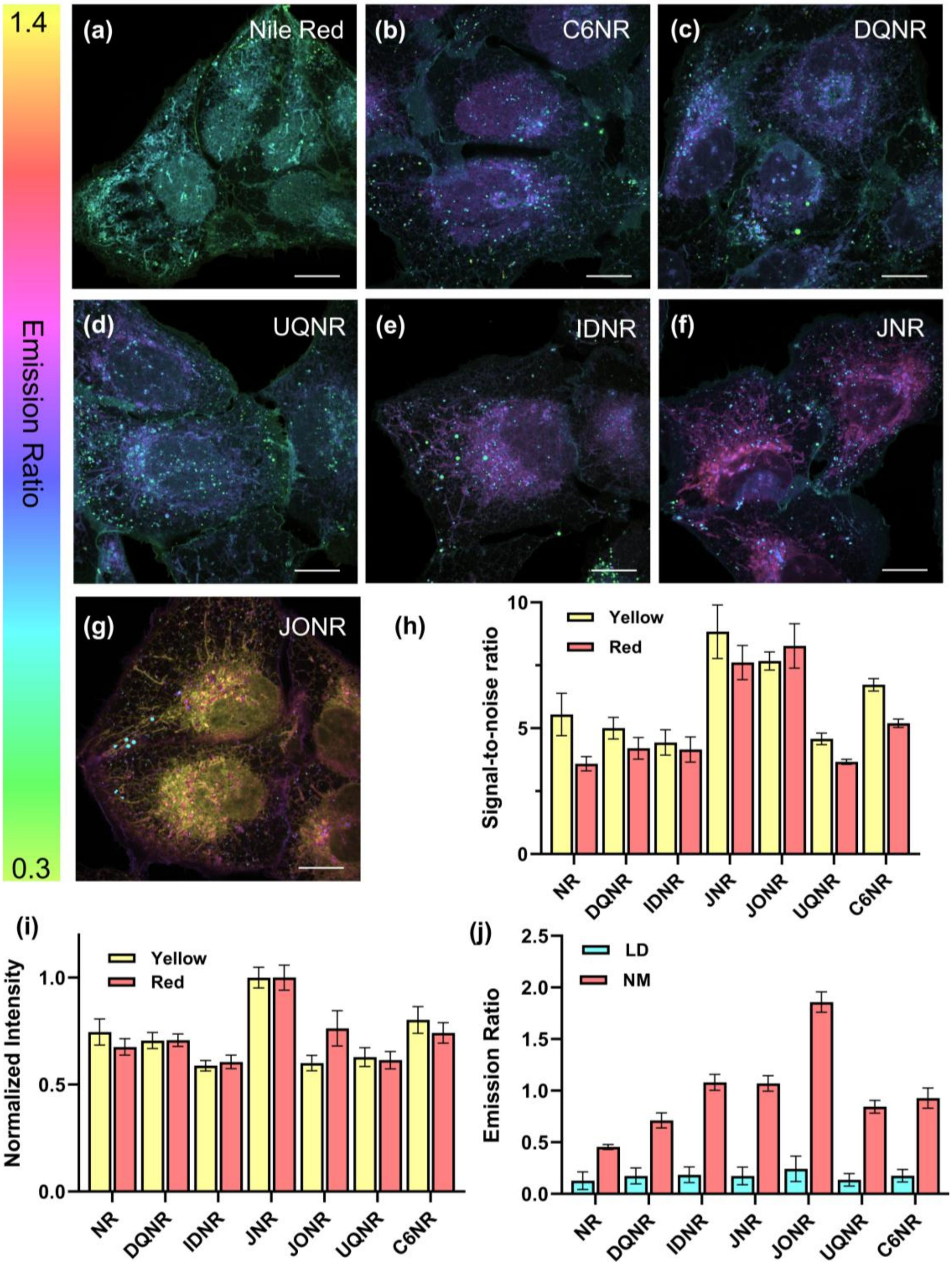

Figure S12. Comparison of imaging performance of Nile Red and derivatives in live cell ratiometric imaging. (a-g) Polarity map of COS-7 cells showing the spatial distribution of membrane polarity based on Nile Red (a), C6NR (b), DQNR (c), UQNR (d), IDNR (e), JNR (f) and JONR (g) staining. (h) Comparison of signal-to-noise ratios (SNR) in mitochondria in two channels stained with different probes. (i) Comparison of brightness in two channels stained with different probes. (j) Comparison of emission ratios in lipid droplets (LD) and nuclear membranes (NM) stained with different probes. Each experiment was independently repeated at least five times. Data are shown as mean ± SEM. Scale bars: 2 μm.

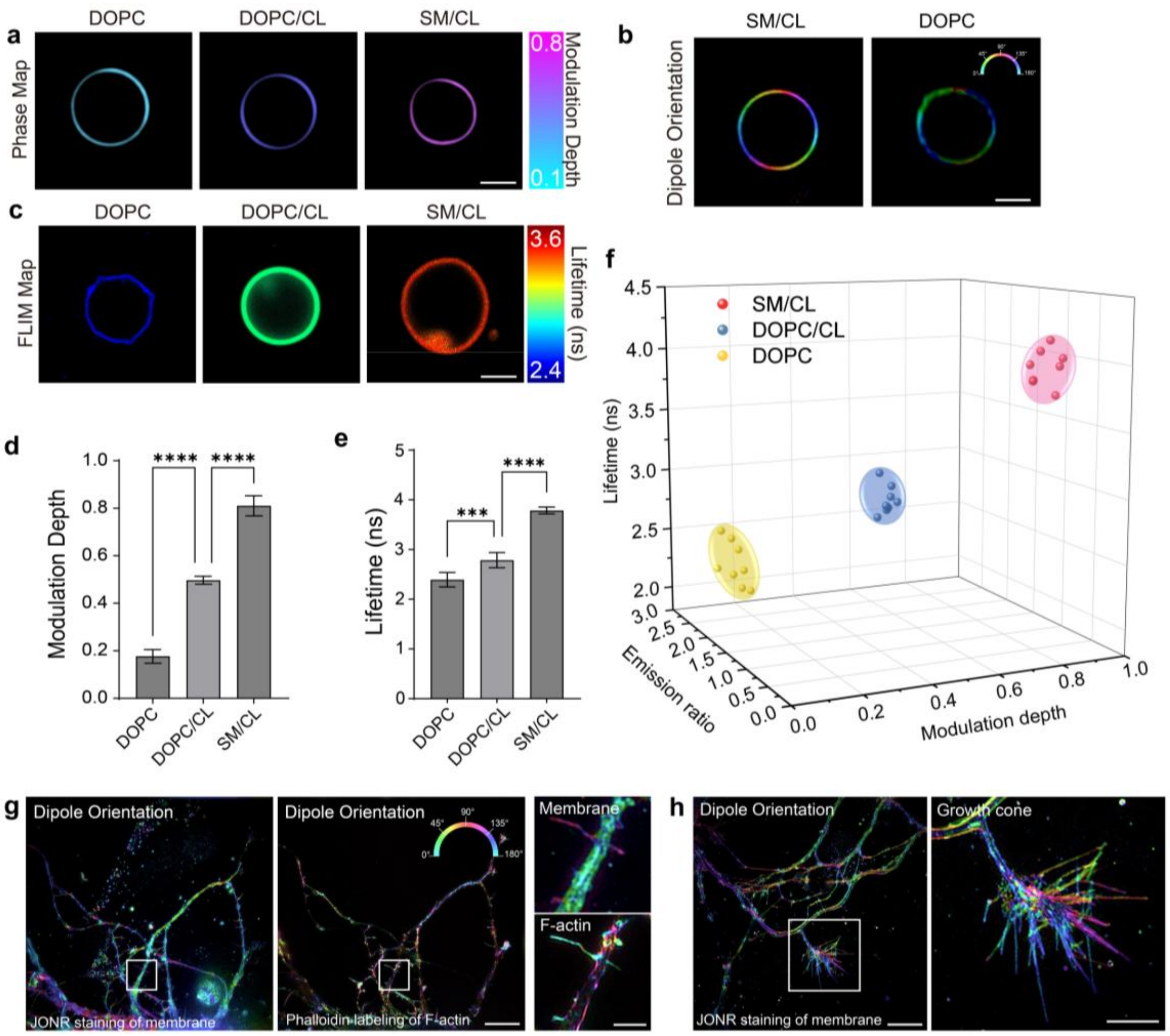

Figure S13. Multidimensional responsiveness of JONR to different lipid compositions. (a) Phase maps of GUVs with different lipid compositions obtained by SPOT imaging, where the color bar indicates the range of modulation depth values. (b) Orientation maps of GUVs composed of different lipid compositions. GUVs composed of SM/CL exhibit more accurately resolved dipole orientations, whereas those composed of pure DOPC show reduced dipole resolution. The hue of the color wheel reflects dipole orientation. (c) FLIM maps of GUVs with different lipid compositions, where the color bar represents the range of fluorescence lifetime values. (d) Quantification of modulation depth values for GUVs with different lipid compositions, corresponding to the phase maps shown in (b). (e) Quantification of fluorescence lifetime values for GUVs with different lipid compositions, corresponding to the FLIM maps shown in (c). (f) Multidimensional mapping of GUVs with different lipid compositions, plotted in three-dimensional coordinates. Solid spheres represent sampling points for different lipid compositions, and the transparent ellipsoids represent the coverage range of all data points. (g) Membrane structures labeled by JONR (left) and F-actin filaments labeled by Phalloidin-Alexa488 (right) in fixed mouse hippocampal neurons. The color coding of the orientation ring corresponds to the angles indicated above. A magnified view of a local region is shown on the right. (h) Dipole orientation mapping of membrane structures labeled by JONR in hippocampal neurons (left panel). A magnified view of the growth cone region is shown (right panel). Each experiment in GUVs was independently repeated more than five times. Data are shown as mean ± SEM. ***$p < 0.001$; ****$p < 0.0001$ (one-way ANOVA). Scale bars: 2 μm (g right), 3 μm (a, b, c),5 μm (h right) and 10 μm (g left, h left).

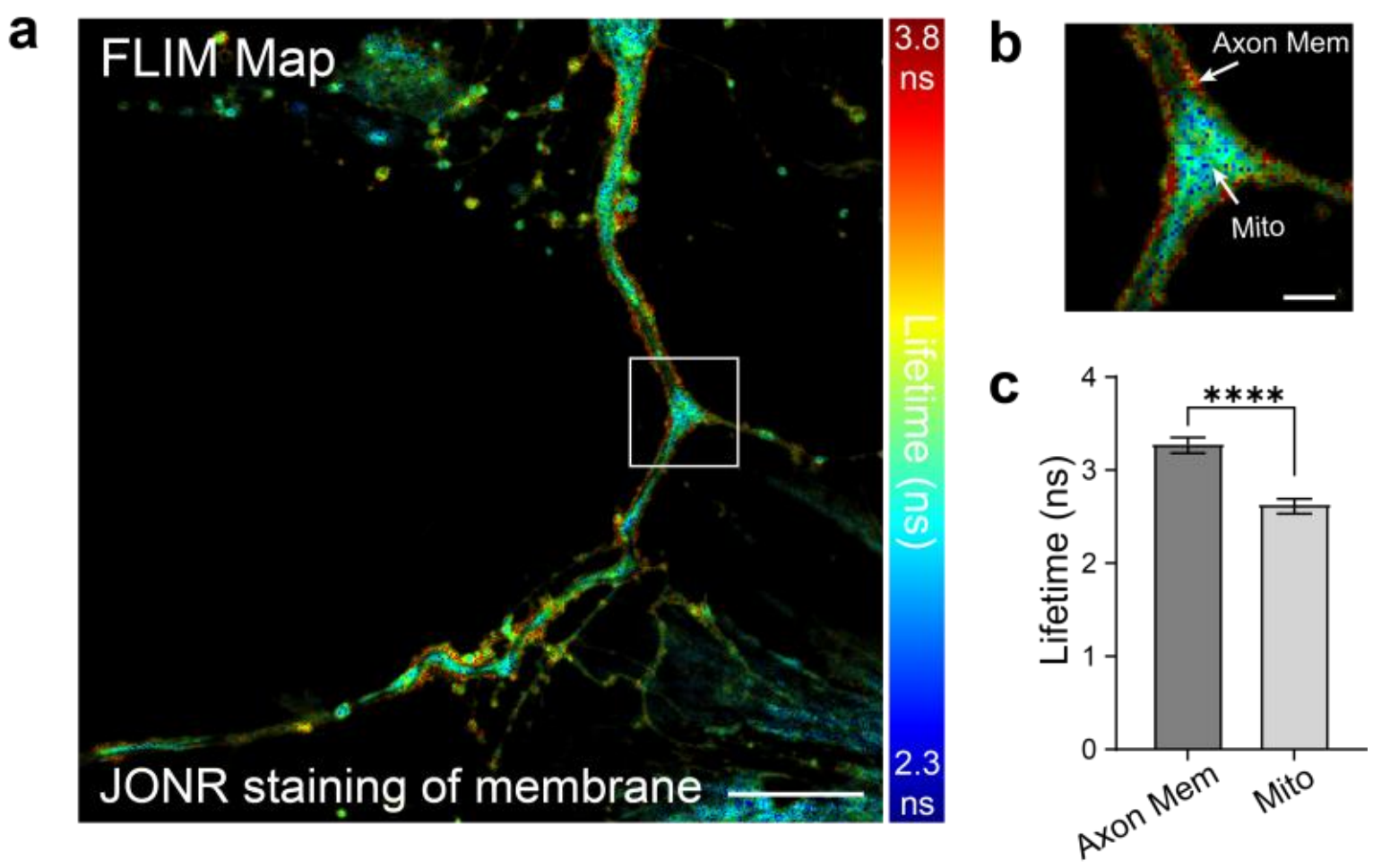

Figure S14. Fluorescence lifetime imaging of neurons labeled with JONR. (a) Representative FLIM image of a neuron membrane stained with JONR. (b) Enlarged views from (a) showing the fluorescence lifetime of the axonal membrane and intra-axonal mitochondria. (c) Statistical comparison reveals a significantly longer fluorescence lifetime in the axonal membrane compared to mitochondria (****$P < 0.0001$, t-test). Data are shown as mean ± SEM. Data are representative of three independent experiments. Scale bars: 10 µm (a), 2 µm (b).

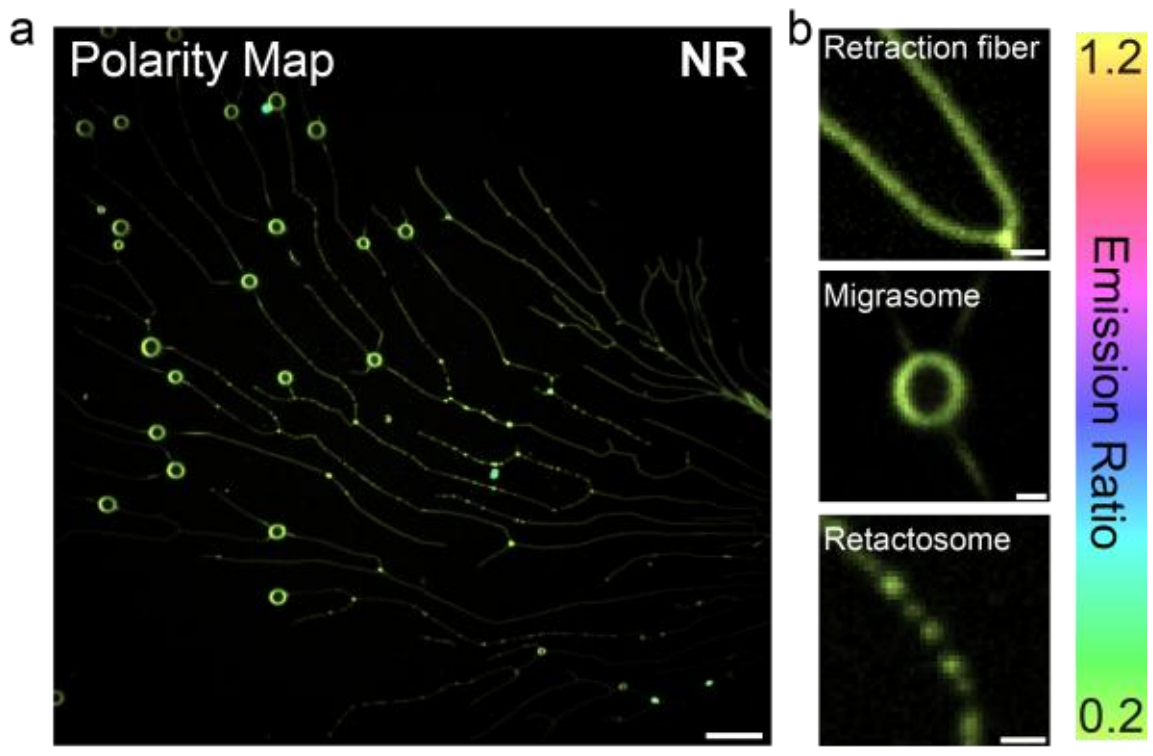

Figure S15. Ratiometric imaging of Nile Red-labeled L929 cells. (a) Overview of migration-related structures in L929 cells. (b) In specific migration-associated structures such as retraction fibers, migrasomes, and retractosomes, the ratio imaging based on Nile Red shows limited ability to distinguish subtle variations in lipid composition. Scale bars: 5 μm (a); 500 nm (b).

Experimental Details

Fluorescence lifetime measurement

JONR was dissolved in spectral grade solvents (dioxane, acetone, DCM, DMSO, MeOH, CD_3OD, EtOH, IPA, n-BuOH, EG) for fluorescence lifetime measurements. A 10 mM stock solution was diluted with different solvents to 2 μM. Fluorescent lifetime measurement was conducted using an FLS980 Spectrometer (Edinburgh Instruments) containing an integrating sphere at room temperature within a 1 cm square quartz cuvette. Fluorescence lifetime data were analyzed using the Fluoracle software (Edinburgh Instruments) by fitting a mono-exponential decay model (n-exponential reconvolution) to the decay. The instrument response function (IRF) used for deconvolution was measured under the same instrument settings. Measurements were carried out at their emission wavelength.

Computational methods

All the DFT calculations were performed by the Gaussian 16 package.[1] Geometry optimization of all structures was performed using the TPSSh functional and the TZVP basis set.[2,3] Frequency calculations were performed to obtain the Gibbs free energy at 298.15 K. The SMD implicit solvation model was used for all calculations, and each S1 state was optimized in the corresponding solvent.[4] The molecular geometry was plotted with CYLView.[5] The isosurface was plotted using VMD.[6] The electron-hole analysis was performed using Multiwfn.[7]

Fixed cell staining process

In general, neuron and L929 cells were fixed with 4% paraformaldehyde (I28800, Thermo Scientific) at room temperature for 10 minutes, followed by PBS buffer (pH =7.4) washes. To label actin filaments, cells were incubated with 1 μM phalloidin–Alexa Fluor 488 (A12379, Invitrogen) for 1 hour at room temperature. After PBS washes, cells were subsequently incubated with 1 μM JONR for 15 minutes to stain lipid membrane structures. Cells were then thoroughly washed with PBS before imaging.

Viability-based phototoxicity assay of HeLa cells

HeLa cells were grown to 80-90 % confluency in a 96-well plate (Costar 3599; Corning, NY, USA) before treatment. To achieve the same brightness after staining, HeLa cells were incubated with 1 μM JONR and 1 μM Nile Red respectively in DMEM (Gibco) for 10 min at 37 °C with 5% CO_2. Then, the cells were washed with PBS three times and maintained in fresh medium for subsequent imaging analysis. 96-well plates were analyzed using a high-content imaging system ImageXpress Micro XLS (Molecular Devices, San Jose, CA, USA), equipped with a 20×/0.4 NA air objective and live-cell imaging device. The average light intensity on cells was measured to be 2.6 W/cm^2 (568 nm). The cells in each well were irradiated with the maximum light intensity at different time points. Time points were selected as 2, 5, 6, 15 and 17 min (each time point with three parallel repeats).

After illumination, the cells were directly used for subsequent imaging analysis. The cells were washed with PBS once and a 100 μL cell viability assay solution containing 1 μM Calcein AM

(Beyotime, Shanghai, China) was added to each sample including controls (without dye incubation). Images of two channels (mitochondria: Cy3 channel, 568 nm; Calcein AM: FITC channel, 488 nm) were recorded on ImageXpress Micro XLS. The cell viability (%) was calculated according to the following equation: cell viability% = B/A *100%, where A = the number of total cells before illumination, and B = the number of Calcein AM-positive cells after illumination. More than 500 cells were counted at each time point.

Measurement of cytotoxicity using Cell Counting Kit-8 (CCK8) reagent

HeLa cells were grown to 80-90 % confluency in a 96-well plate (Costar 3599, Corning) the day before treatment. Cells were either stained with TMRE, JONR, and Nile Red with 5 different concentrations (0.25, 0.5, 1, 2, 5 μM in DMEM) for 12 hours at 37 °C with 5% CO_2. Cells were washed with PBS once after the staining procedure and incubated with cultured medium containing 10% Cell Counting Kit-8 reagent (CCK8; C0038; Beyotime) for 2 hours at 37 °C with 5% CO_2. The absorbance (450 nm) of each well was recorded using a TECAN Infinite M Nano+ microplate reader (TECAN, Männedorf, Switzerland).

Colocalization imaging of live-cell samples

To validate the effectiveness of JONR in labeling membrane-bound cellular structures, we performed spectral ratio imaging using JONR staining while simultaneously labeling individual membrane-bound organelles with fluorescent dyes or fluorescent proteins in live cells. In addition to spectral ratio imaging, a green detection channel (488 nm laser excitation, 525 ± 20 nm emission detection) was introduced to enable colocalization analysis. This approach allowed for the identification and characterization of over ten distinct membrane-bound subcellular structures.

For COS-7 cells, specific membrane-bound organelles were labeled using either plasmids or fluorescent dyes to achieve colocalization imaging. Peroxisomes were labeled with EGFP–SKL, late endosomes with mEGFP–Rab7a, early endosomes with GFP–Rab5, lysosomes with EGFP–LAMP1, Golgi apparatus with GFP–SiT, and the endoplasmic reticulum (ER) with mEmerald–Sec61b. Transfection was performed using Lipofectamine 3000 (Life Technologies) according to the manufacturer's protocol. Briefly, cells were transfected in Opti-MEM (Life Technologies) with Lipofectamine 3000 per well, incubated for 6 hours, and then replaced with standard culture medium. In addition to plasmid-based labeling, mitochondria were labeled with MitoTracker (A66468, Invitrogen) at a concentration of 250 nM for 20 minutes, followed by a medium exchange. Nuclei were labeled with NucBlue™ Live ReadyProbes™ Reagent (Hoechst, 33342) at 200 nM for 10 minutes. Migrasomes, plasma membranes, tunneling nanotubes, retraction fibers, and retractosomes were labeled with 100 nM Alexa Fluor 488-conjugated WGA (W11261, Invitrogen) for 10 minutes and manually annotated.

Data Availability Statement

Data presented in this study are provided in Supplementary Tables. Data are available from the corresponding authors upon request.

Ethics approval and consent to participate

Primary cell isolation procedures were approved by the Peking University Animal Use and Care Committee and complied with the standards of the Association for Assessment and Accreditation of

Laboratory Animal Care (code number IMM-ChenZX-1).

General procedures on synthetic chemistry

Unless otherwise stated, all commercially available materials were purchased at the highest commercial quality and used without further purification. Anhydrous DMF was purchased from MREDA (China), and anhydrous THF was purchased from Innochem (China). All reactions were carried out with dry solvents, unless otherwise mentioned.

Reactions were monitored by Thin Layer Chromatography on plates (GF254) (Yantai Chemicals) using UV light as visualizing agent or by LC/MS (4.6 mm × 150 mm 5 μm C18 column; 5 μL injection; 5-100% CH_3CN/H_2O, linear gradient, with constant 0.1 % v/v formic acid additive; 6 min run; 0.5 mL/min flow; ESI; positive or negative ion mode; detection at 550 nm). If not specially mentioned, flash column chromatography uses silica gel (200-300 mesh) supplied by Tsingtao Haiyang Chemicals (China).

NMR spectra were recorded on Brüker Advance 400 (^{1}H 400 MHz, ^{13}C 101 MHz) and are calibrated using residual undeuterated solvent ($CDCl_3$ at 7.26 ppm ^{1}H NMR, 77.16 ppm ^{13}C NMR; CD_3OD at 3.31 ppm ^{1}H NMR, 49.00 ppm ^{13}C NMR). In order to improve the solubility, several samples were dissolved in a mixture of $CDCl_3$ and CD_3OD and have been marked below. Data for ^{1}H NMR spectra are reported as follows: chemical shift (δ ppm), multiplicity (s = singlet, d = doublet, t = triplet, dd = doublet of doublets, m = multiplet, br = broad), coupling constant (Hz), integration. Data for ^{13}C NMR are reported by chemical shift (δ ppm).

Chemical synthesis

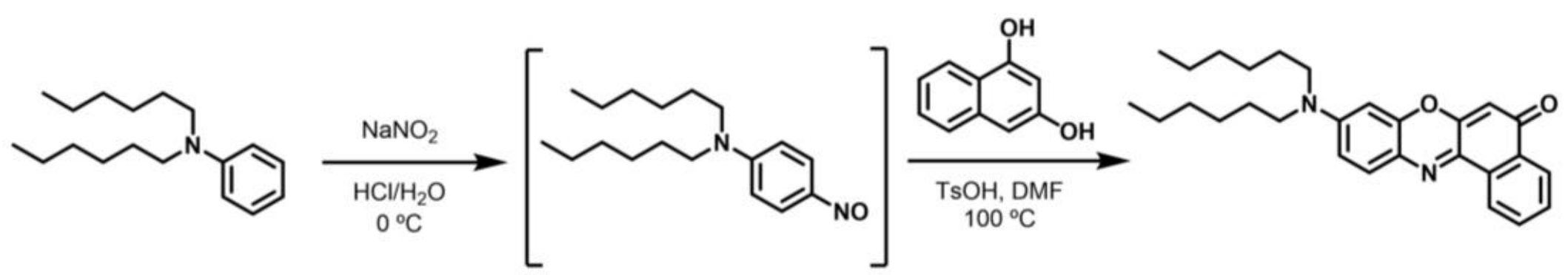

1 C6NR

C6NR: N,N-dihexylaniline (**1**, 1.00 g, 3.83 mmol) was dissolved in a mixture of concentrated HCl (2.00 mL, 23.1 mmol) and H_2O (1.20 mL), and the reaction mixture was cooled to 0 °C. A solution of $NaNO_2$ (317 mg, 4.60 mmol, 1.2 eq) in H_2O (3 mL) was added dropwise over 5 min, and the reaction mixture was stirred for another 8 min at 0 °C (ice bath). The reaction mixture was diluted with ice water, and a saturated solution of $NaHCO_3$ was slowly added to give a neutral solution. The product was extracted with DCM (3 × 10 mL), and the combined organic extracts were concentrated *in vacuo*. A solution of 1,6-naphthalenediol (674 mg, 4.21 mmol, 1.1 eq) and *p*-TsOH (65 mg, 0.380 mmol, 0.1 eq) in anhydrous DMF (10 mL) was added to the crude intermediate. The resulting reaction mixture was stirred at 100 °C for 10 h. After cooling to room temperature, the mixture was extracted with EtOAc (2 × 25 mL). The combined organic layer was washed with H_2O (20 mL), and then dried over anhydrous Na_2SO_4, and filtered. After concentration of the filtrate under reduced pressure, the crude product was purified by silica gel column chromatography (0-2% MeOH in DCM) to afford **C6NR** (1.45 g, 3.37 mmol, 88% yield).

^{1}H NMR (400 MHz, Methanol-*d*$_4$) δ 8.61 (d, *J* = 8.0 Hz, 1H), 8.21 (d, *J* = 7.8 Hz, 1H), 7.73 (t, *J* = 7.0 Hz, 1H), 7.67 – 7.62 (m, 1H), 7.60 – 7.54 (m, 1H), 6.69 (s, 1H), 6.43 (s, 1H), 6.36 – 6.31 (m, 1H), 3.38 (s, 4H), 1.66 (s, 4H), 1.37 (s, 12H), 0.96 – 0.88 (m, 6H).

^{13}C NMR (101 MHz, Methanol-*d*$_4$) δ 184.34, 152.69, 151.69, 146.75, 138.39, 132.05, 131.43, 131.17, 131.12, 129.62, 125.29, 125.20, 123.66, 110.67, 104.54, 96.05, 51.36, 31.52, 27.15, 26.53, 22.48, 13.52.
MS (ESI) calculated for $C_{28}H_{35}N_2O_2$ (MH^+) 431.3, observed 431.0.

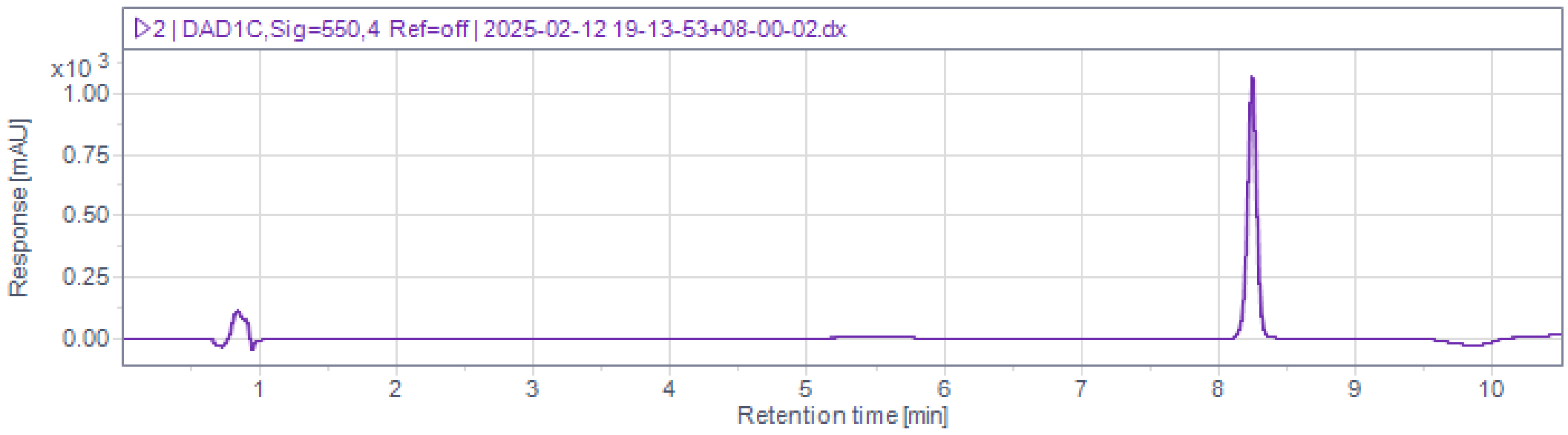

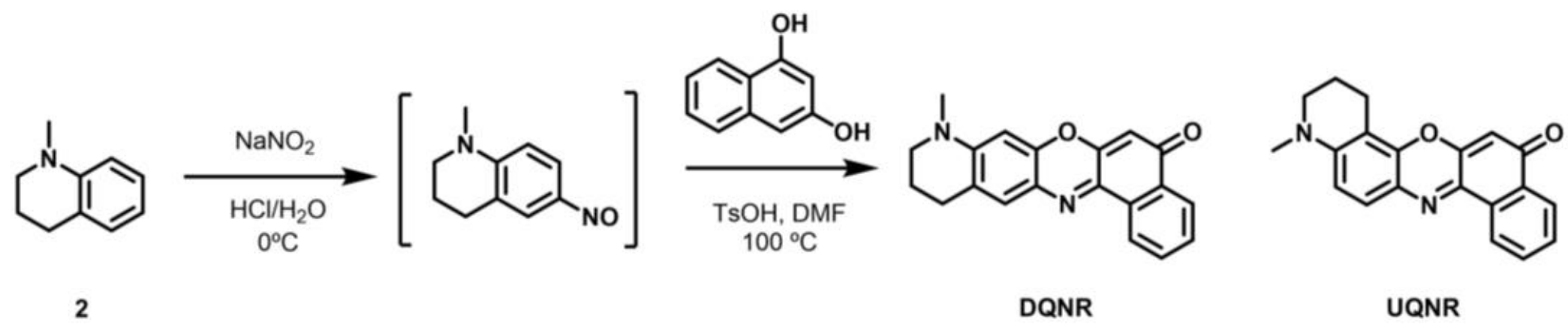

DQNR & UQNR: 1-methyl-1,2,3,4-tetrahydroquinoline (**2**, 1.00 g, 6.80 mmol) was dissolved in a mixture of concentrated HCl (2.00 mL, 23.1 mmol) and H_2O (1.20 mL), and the reaction mixture was cooled to 0 °C. A solution of $NaNO_2$ (563 mg, 8.16 mmol, 1.2 eq) in H_2O (3 mL) was added dropwise over 5 min, and the reaction mixture was stirred for another 8 min at 0 °C (ice bath). The reaction mixture was diluted with ice water, and a saturated solution of $NaHCO_3$ was slowly added to give a neutral solution. The product was extracted with DCM (3 × 10 mL), and the combined organic extracts were concentrated *in vacuo*. A solution of 1,6-naphthalenediol (1.20 g, 7.48 mmol, 1.1 eq) and *p*-TsOH (117 mg, 0.680 mmol, 0.1 eq) in anhydrous DMF (10 mL) was added to the crude intermediate and the resulting reaction mixture was stirred at 100 °C for 10 h. After cooling the reaction mixture to room temperature, the mixture was extracted with EtOAc (2 × 25 mL). The combined organic layer was washed with H_2O (20 mL), and then dried over anhydrous Na_2SO_4, and filtered. After concentration of the filtrate under reduced pressure, the crude product was purified by silica gel column chromatography (0-10% MeOH in DCM) to afford **DQNR** (860 mg, 2.72 mmol, 50% yield) and **UQNR** (709 mg, 2.24 mmol, 23% yield). **UQNR** was characterized by NMR using mixed solvents of $CDCl_3$ and CD_3OD (1:2).
^{1}H NMR (**DQNR**, 400 MHz, Chloroform-*d*) δ 8.64 (d, J = 7.1 Hz, 1H), 8.33 (d, J = 7.7 Hz, 1H), 7.75 – 7.70 (m, 1H), 7.68 – 7.63 (m, 1H), 7.31 (s, 1H), 6.38 (s, 1H), 6.32 (s, 1H), 3.44 – 3.38 (m, 2H), 3.00 (s, 3H), 2.80 (t, J = 6.2 Hz, 2H), 2.00 (p, J = 6.1 Hz, 2H).
^{13}C NMR (**DQNR**, 101 MHz, Chloroform-*d*) δ 183.46, 152.23, 149.60, 145.51, 139.39, 132.06, 131.71, 131.13, 129.70, 129.02, 125.62, 125.08, 123.68, 121.19, 105.23, 95.16, 51.06, 39.20, 27.27, 21.57.
MS (**DQNR,** ESI) calculated for $C_{20}H_{17}N_2O_2$ (MH^+) 317.1, observed 317.1.

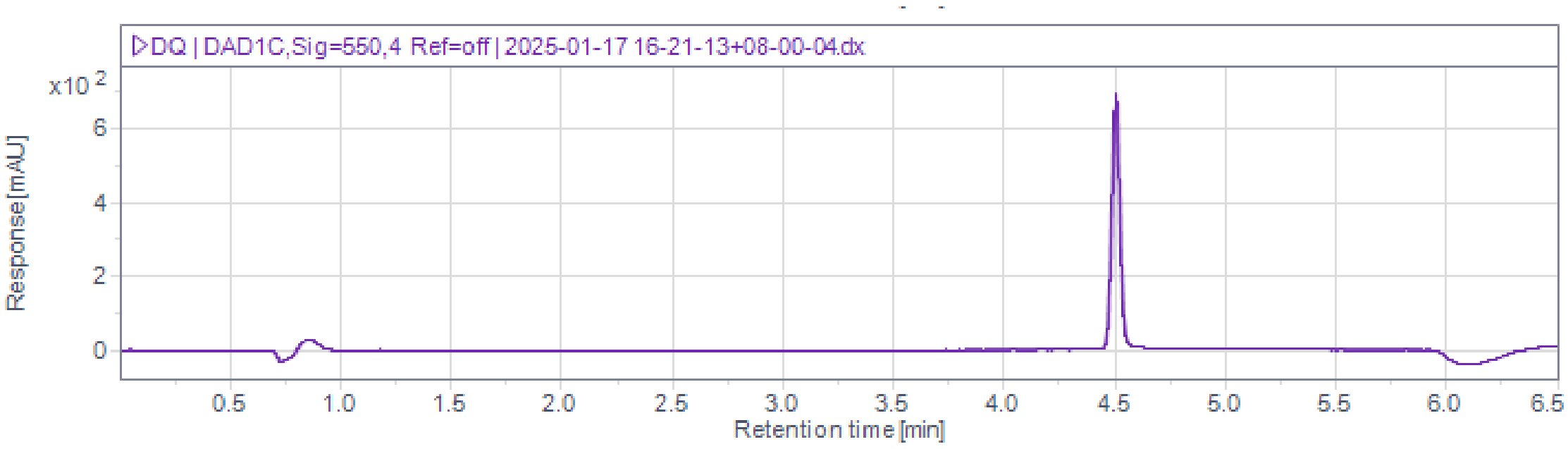

^{1}H NMR (**UQNR**, 400 MHz, Methanol-d_4) δ 8.67 (d, J = 7.3 Hz, 1H), 8.25 (d, J = 7.9 Hz, 1H), 7.79 – 7.74 (m, 1H), 7.69 – 7.64 (m, 1H), 7.56 (d, J = 9.1 Hz, 1H), 6.74 (d, J = 9.1 Hz, 1H), 6.41 (s, 1H), 3.48 – 3.42 (m, 2H), 3.11 (s, 3H), 2.93 – 2.89 (m, 2H), 2.05 (dt, J = 11.8, 6.3 Hz, 2H).

^{13}C NMR (**UQNR**, 101 MHz, Methanol-d_4) δ 183.53, 152.63, 150.37, 137.50, 132.07, 131.52, 130.87, 129.86, 129.61, 129.46, 126.43, 125.27, 123.69, 109.92, 106.89, 104.27, 50.99, 39.19, 20.42, 19.63.

MS (**UQNR,** ESI) calculated for $C_{20}H_{17}N_2O_2$ (MH^+) 317.1, observed 317.1.

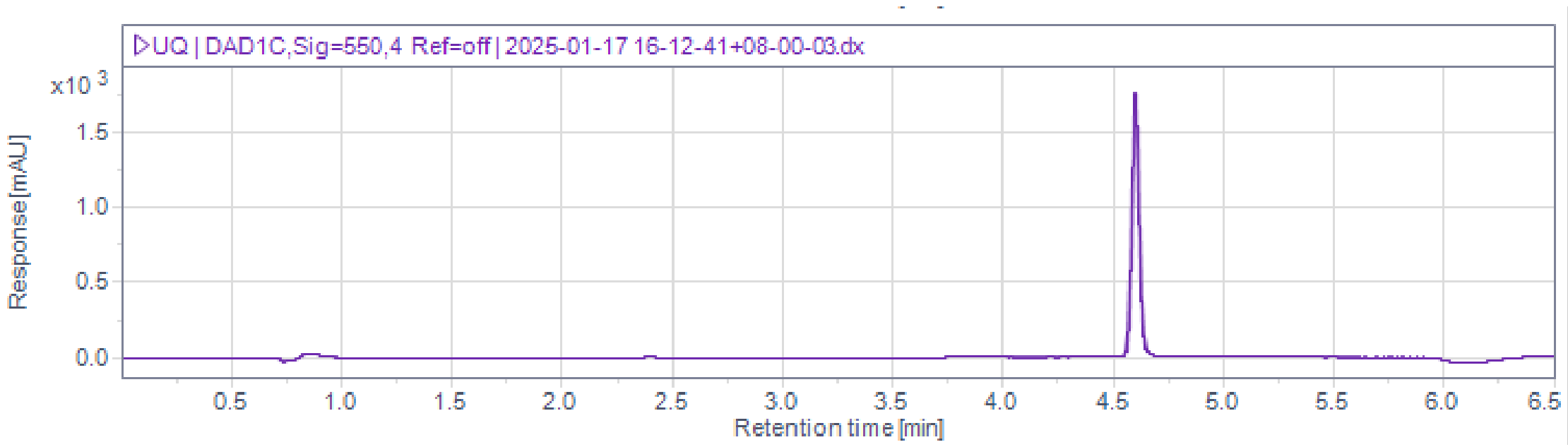

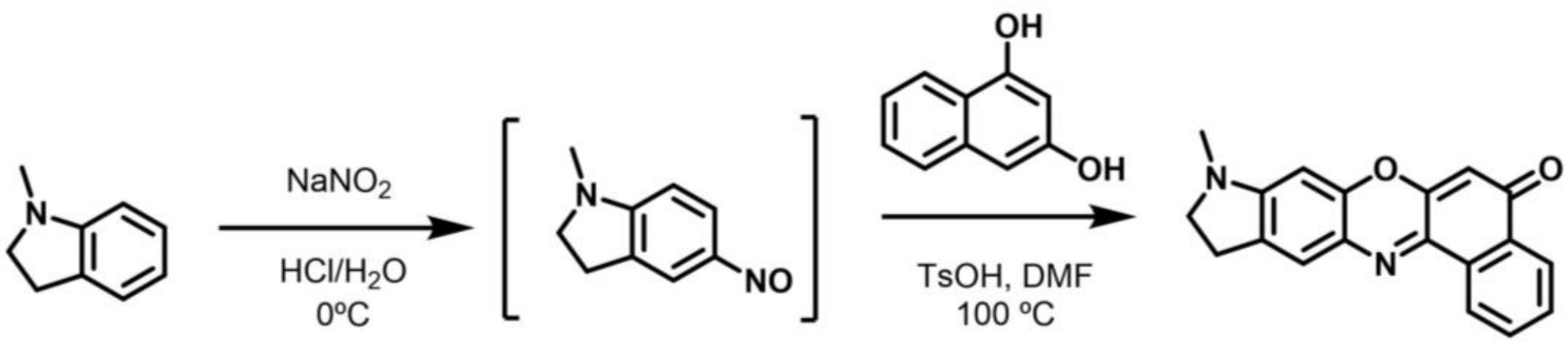

4 **IDNR**

IDNR: 1-methylindoline (**3**, 1.00 g, 7.52 mmol) was dissolved in a mixture of concentrated HCl (3.00 mL, 34.5 mmol) and H_2O (2.40 mL), and the reaction mixture was cooled to 0 °C. A solution of $NaNO_2$ (623 mg, 9.02 mmol, 1.2 eq) in H_2O (4 mL) was added dropwise over 5 min, and the reaction mixture was stirred for another 8 min at 0 °C (ice bath). The reaction mixture was diluted with ice water, and a saturated solution of $NaHCO_3$ was slowly added to give a neutral solution. The product was extracted with DCM (3 × 10 mL), and the combined organic extracts were concentrated *in vacuo*. To the crude intermediate, a solution of 1,6-naphthalenediol (1.32 g, 8.27 mmol, 1.1 eq) and *p*-TsOH (129 mg, 0.752 mmol, 0.1 eq) in anhydrous DMF (10 mL) was added and the resulting reaction mixture was stirred at 100 °C for 10 h. After cooling the reaction mixture to room temperature, the mixture was extracted with EtOAc (2 × 40 mL). The combined organic layer was washed with H_2O (40 mL), and then dried over anhydrous Na_2SO_4, and filtered. After concentration of the filtrate under reduced pressure, the crude product was purified by silica gel column

chromatography (0-10% MeOH in DCM) to afford **IDNR** (1.63 g, 5.41 mmol, 72% yield). **IDNR** was characterized by NMR using mixed solvents of $CDCl_3$ and CD_3OD (2:1).

^{1}H NMR (400 MHz, Methanol-*d*$_4$) δ 8.66 (d, *J* = 7.7 Hz, 1H), 8.26 (d, *J* = 8.0 Hz, 1H), 7.74 (t, *J* = 6.9 Hz, 1H), 7.65 (t, *J* = 8.1 Hz, 1H), 7.37 (s, 1H), 6.38 (s, 1H), 6.22 (s, 1H), 3.69 (t, *J* = 8.1 Hz, 2H), 3.12 (d, *J* = 7.7 Hz, 2H), 2.95 (s, 3H).

MS (ESI) calculated for $C_{19}H_{15}N_2O_2$ (MH^+) 303.1, observed 303.1.

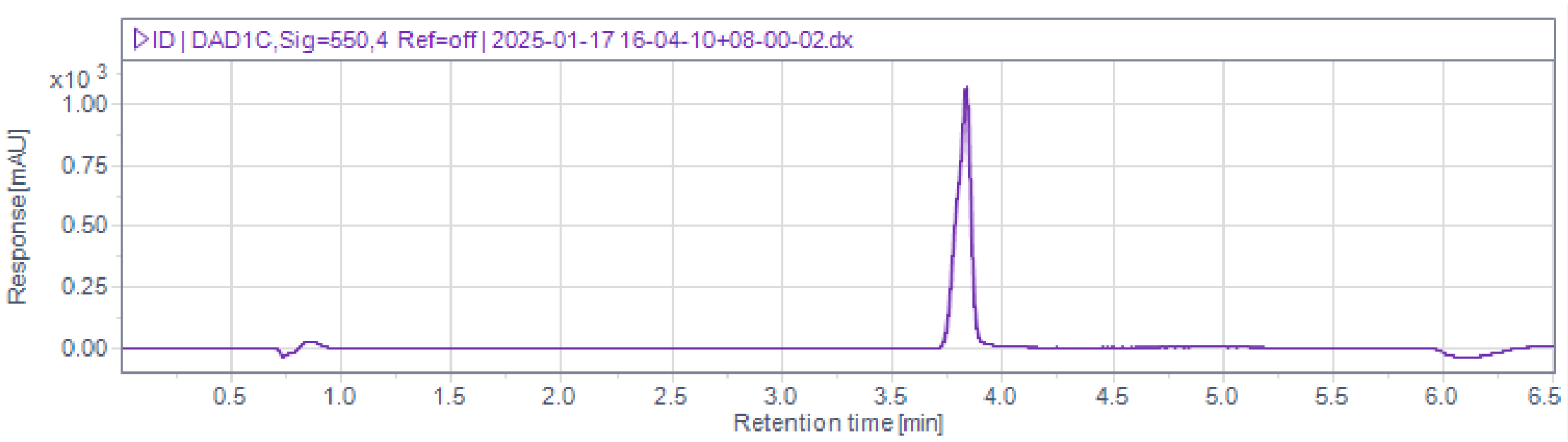

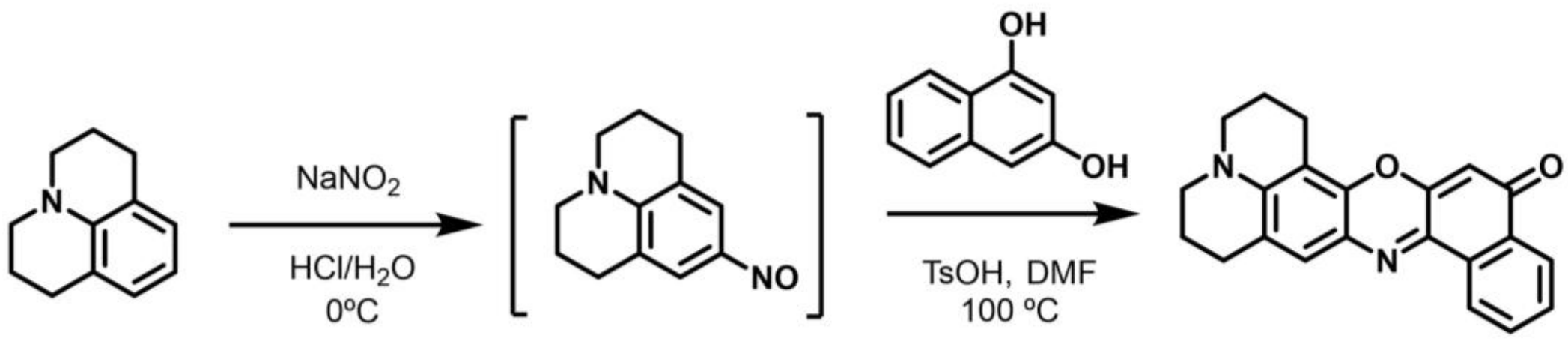

4 JNR

JNR: Julolidine (**4**, 1.00 g, 5.78 mmol) was dissolved in a mixture of concentrated HCl (3.00 mL, 34.5 mmol) and H_2O (2.40 mL), and the reaction mixture was cooled to 0 °C. A solution of $NaNO_2$ (479 mg, 6.94 mmol, 1.2 eq) in H_2O (4 mL) was added dropwise over 5 min, and the reaction mixture was stirred for another 6 min at 0 °C (ice bath). The reaction mixture was diluted with ice water, and a saturated solution of $NaHCO_3$ was slowly added to give a neutral solution. The product was extracted with DCM (3 × 10 mL), and the combined organic extracts were concentrated *in vacuo*. A solution of 1,6-naphthalenediol (1.00 g, 6.36 mmol, 1.1 eq) and *p*-TsOH (99 mg, 0.578 mmol, 0.1 eq) in anhydrous DMF (10 mL) was added to the crude intermediate and the resulting reaction mixture was stirred at 100 °C for 10 h. After cooling to room temperature, the reaction mixture was extracted with EtOAc (2 × 40 mL). The combined organic layer was washed with H_2O (40 mL), and then dried over anhydrous Na_2SO_4, and filtered. After concentration of the filtrate under reduced pressure, the crude product was purified by silica gel column chromatography (0-10% MeOH in DCM) to afford **JNR** (1.58 g, 4.62 mmol, 80% yield). **JNR** was characterized by NMR using mixed solvents of $CDCl_3$ and CD_3OD (1:3).

^{1}H NMR (400 MHz, Methanol-*d*$_4$) δ 8.62 – 8.57 (m, 1H), 8.24 – 8.20 (m, 1H), 7.75 – 7.69 (m, 1H), 7.63 (td, *J* = 7.6, 1.3 Hz, 1H), 7.14 (s, 1H), 6.33 (s, 1H), 3.35 (d, *J* = 5.9 Hz, 2H), 3.33 – 3.31 (m, 2H), 2.80 (t, *J* = 6.4 Hz, 2H), 2.77 – 2.73 (m, 2H), 2.00 (d, *J* = 6.1 Hz, 2H), 1.97 (d, *J* = 6.3 Hz, 2H).

^{13}C NMR (101 MHz, Methanol-*d*$_4$) δ 183.21, 152.63, 147.35, 142.06, 135.96, 132.08, 131.07, 130.83, 129.02, 127.62, 126.16, 125.05, 123.47, 121.43, 105.13, 103.41, 50.24, 49.75, 27.28, 20.85, 19.90, 19.47.

MS (ESI) calculated for $C_{22}H_{19}N_2O_2$ (MH^+) 343.1, observed 343.1.

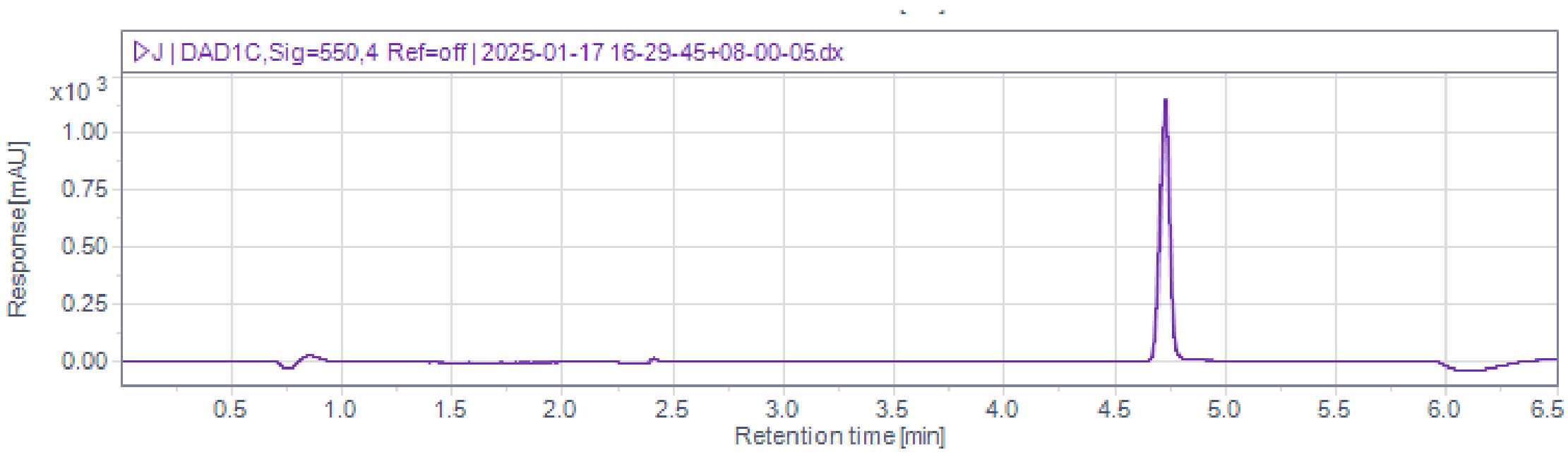

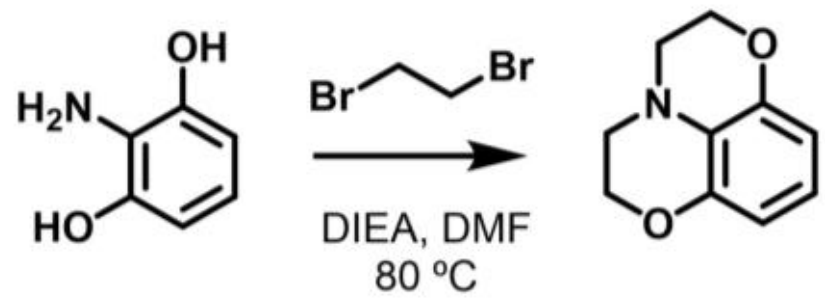

5

Compound **5**: 2-aminobenzene-1,3-diol (1.00 g, 8.00 mmol) was dissolved in a mixture of 1,2-dibromoethane (3.31 g, 17.6 mmol, 2.2 eq), Diisopropylethylamine (DIEA, 4.55 g, 35.2 mmol, 4.4 eq) and DMF (4 mL). The reaction mixture was stirred at 80 °C for 10 h. After cooling to room temperature, the reaction mixture was extracted with EtOAc (3 × 40 mL). The combined organic layer was washed with H_2O (80 mL), and then dried over anhydrous Na_2SO_4, and filtered. After concentration of the filtrate under reduced pressure, the crude product was purified by silica gel column chromatography (10% EtOAc in hexane) to afford compound **5** (1.28 g, 7.22 mmol, 90% yield).

^{1}H NMR (400 MHz, Chloroform-*d*) δ 6.65 – 6.58 (m, 1H), 6.44 (d, J = 8.1 Hz, 2H), 4.44 – 4.38 (m, 4H), 3.20 – 3.14 (m, 4H).

^{13}C NMR (101 MHz, Chloroform-*d*) δ 144.64, 121.96, 118.92, 108.38, 65.29, 46.86.

MS (ESI) calculated for $C_{10}H_{12}NO_2$ (MH^+) 178.1, observed 178.0.

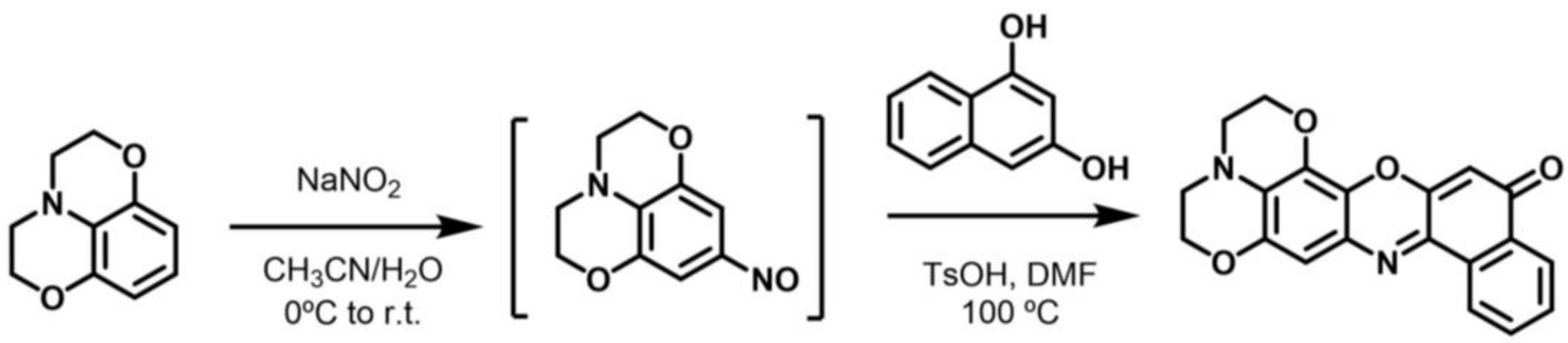

5

JONR

JONR: 2,3,4,5-tetrahydro-1,6-dioxa-3a-azaphenalene (**5**, 500 mg, 2.83 mmol) was dissolved in a mixture of CH_3CN (2.00 mL) and concentrated HCl (1.00 mL, 11.5 mmol), and the reaction mixture was cooled to 0 °C. A solution of $NaNO_2$ (234 mg, 3.39 mmol, 1.2 eq) in H_2O (2 mL) was added dropwise over 3 min, and the reaction mixture was stirred for another 7 min at 0 °C (ice bath). The reaction mixture was diluted with ice water, and a saturated solution of $NaHCO_3$ was slowly added to give a neutral solution. The product was extracted with DCM (2 × 20 mL), and the combined organic extracts were concentrated *in vacuo*. A solution of 1,6-naphthalenediol (500 mg, 31.1 mmol,

1.1 eq) and *p*-TsOH (48 mg, 0.28 mmol, 0.1 eq) in anhydrous DMF (10 mL) was added to the crude intermediate. The resulting reaction mixture was stirred at 100 °C for 10 h. After cooling to room temperature, the reaction mixture was extracted with EtOAc (3 × 30 mL). The combined organic layer was washed with H_2O (50 mL), and then dried over anhydrous Na_2SO_4, and filtered. After concentration of the filtrate under reduced pressure, the crude product was purified by silica gel column chromatography (0-5% MeOH in DCM) to afford **JONR** (663 mg, 1.92 mmol, 68% yield). **JONR** was characterized by NMR using mixed solvents of $CDCl_3$ and CD_3OD (1:1).

^{1}H NMR (400 MHz, Methanol-*d*$_4$) δ 8.69 (d, *J* = 7.1 Hz, 1H), 8.26 (d, *J* = 7.2 Hz, 1H), 7.79 – 7.74 (m, 1H), 7.71 – 7.67 (m, 1H), 6.95 (s, 1H), 6.51 (s, 1H), 4.52 – 4.48 (m, 2H), 4.41 – 4.37 (m, 2H), 3.48 – 3.45 (m, 2H), 3.44 – 3.41 (m, 2H).

^{13}C NMR (101 MHz, Methanol-*d*$_4$) δ 183.58, 151.76, 141.14, 140.93, 131.67, 131.25, 130.73, 130.41, 129.34, 129.02, 127.90, 126.33, 125.40, 124.10, 107.87, 104.96, 64.77, 64.31, 46.19, 29.58.

MS (ESI) calculated for $C_{20}H_{15}N_2O_4$ (MH^+) 347.1, observed 347.1.

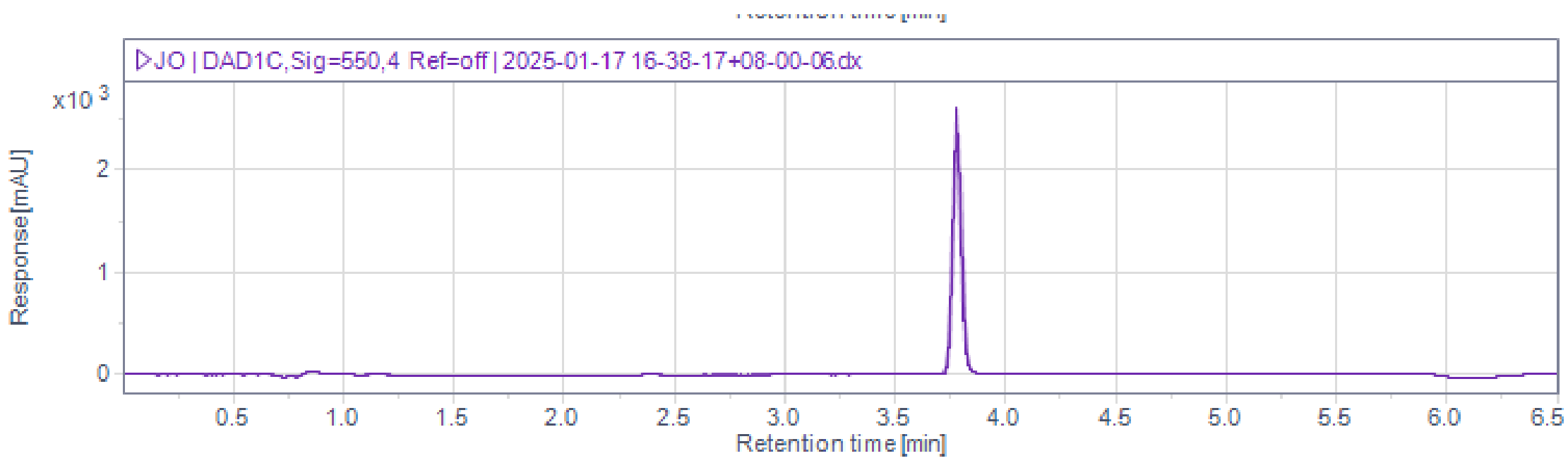

Microscopy Setup and Spectral Ratio Imaging

The SPOT system setup

Our Spectrum and Polarization Optical Tomography (SPOT) system is built upon a commercial inverted fluorescence microscope (Ti2-E, Nikon), equipped with a TIRF objective (100×, 1.49 NA, CFI SR HP Apochromat TIRF 100XC Oil, Nikon). The system employs four lasers (EC30841: MDL-III-405-200mW EF60004, MDL-III-488-180mW EF60009, MGL-FN-561-200mW DE52479, MDL-III-640-200mW EF60007), which are combined using a dichroic mirror set to generate a multicolor excitation source. Laser wavelength selection and intensity modulation are precisely controlled via an acousto-optic tunable filter (AOTF, AA Opto-Electronic, France). To ensure high beam quality, the laser beams are passed through a filtering system composed of focusing lens, collimating lens, and polarization-maintaining single-mode optical fiber (PMC405-PC-2, LBTEK). The collimated beams are then directed into a pure-phase grating module, consisting of polarizing beam splitter (PBS), half-wave plate (AVR1-VIS), and ferroelectric liquid crystal spatial light modulator (FC-SLM, QXGA-3DM, Fourth Dimension Display).

Following phase modulation, the beams are focused onto the pupil plane by an achromatic Fourier lens (AC254-300-A-ML, Thorlabs). To suppress unwanted zeroth-order light and stray diffraction artifacts caused by gaps in the SLM, we implemented a passive spatial filter (Mask) that selectively transmits only the desired ±1st-order diffraction beams. Furthermore, to maximize the contrast of the interference pattern at the sample plane, we designed a customized segmented half-wave plate (Pizza-HWP) that ensures all ±1st-order diffracted beams are modulated into s-polarization. The modulated diffraction beams are relayed to the objective's back focal plane via a relay lens pair, while a polarization-maintaining dichroic mirror (ZT405-415/488/561/640-phase fR-UF3) ensures a high polarization ratio at the objective's back focal plane. Upon passing through the objective, the diffracted beams interfere at the sample plane, generating structured illumination. The fluorescence emitted by the excited sample is transmitted through the dichroic mirror and emission filter (ZET405/488/561/640mv2) and then enters the multispectral imaging splitter (Multisplit V2, Cairn). This splitter spatially separates fluorescence signals of different wavelengths, directing them to distinct regions of the same camera sensor, thereby enabling simultaneous multicolor imaging. A high-quantum-efficiency sCMOS camera (Flash 4.0 V3, Hamamatsu, Japan) is used to capture the fluorescence signals with high sensitivity.

The LCSM and FLIM setup

Fluorescence lifetime imaging microscopy (FLIM) based on laser scanning confocal microscopy (LCSM) was performed using a gated STED microscope (Leica TCS SP8 STED 3X, Leica Microsystems), equipped with a 100×/1.40 NA oil-immersion objective (HCX PL APO, Leica). A white-light laser source provided 80 MHz pulsed excitation over a wavelength range of 470–670 nm. For fluorescence lifetime imaging, the excitation wavelength was set to 561 nm and the emission was collected across the broader range of 590–705 nm.

SPOT image acquisition and processing workflow

SPOT achieves polarization modulation of fluorescent dipoles by altering the polarization state of the excitation light. At the detection end, an integrated image splitter enables simultaneous

acquisition of emission images in the yellow (580-653 nm) and red (665-702 nm) channels, allowing quantification of spectral shift ranges.

The excitation light at 561 nm is used to illuminate the sample. To acquire raw images required for reconstruction, the spatial light modulator (SLM) sequentially loads three different structured illumination directions. For each direction, two phase-shifted patterns (with a phase difference of π) are applied, resulting in a total of six structured illumination images per acquisition cycle.

The raw dual-channel images obtained on the same camera sensor are separated into individual channels, followed by inter-channel registration and single-channel intensity correction.

To generate high-contrast interference fringes, the polarization direction of the excitation beam must be aligned with the fringe orientation on the sample. In SPOT, the polarization orientation of the structured illumination is varied across 0°, 60°, and 120° to match each fringe direction, thereby ensuring effective polarization modulation of fluorescent dipoles.

Each channel’s raw image stack is reconstructed using the HiLo algorithm, which combines the high- and low-spatial frequency components of each pattern direction. This suppresses weakly modulated out-of-focus background signals, thereby enhancing the accuracy of lipid polarity and phase measurements.

By fitting the HiLo-reconstructed images acquired under three polarization directions of structured illumination, the modulation depth of fluorescent dipoles can be extracted to generate the Phase Map. The dipole orientation can also be retrieved through the same fitting process.

To calculate the **modulation depth** and determine the **dipole orientation** β, the emission intensity $I_{HiLo,Pi}$ of a fluorescent dipole under an incident polarization angle θ_i is expressed as:

$$I_{HiLo,Pi} = I_{DC} + I_{AC} \cdot \cos\left(2\theta_i - 2\beta\right), i = 1,2,3 \tag{1-1}$$

In matrix form, the equation can be rewritten as:

$$\begin{bmatrix} l_{HiLo,P1} \\ l_{HiLo,P2} \\ l_{HiLo,P3} \end{bmatrix} = \begin{bmatrix} 1 & \cos 2\theta_1 & \sin 2\theta_1 \\ 1 & \cos 2\theta_2 & \sin 2\theta_2 \\ 1 & \cos 2\theta_3 & \sin 2\theta_3 \end{bmatrix} \begin{bmatrix} I_{DC} \\ I_{AC} \times \cos 2\beta \\ I_{AC} \times \sin 2\beta \end{bmatrix} \tag{1-2}$$

Here, I_{DC} represents the direct current (DC) component, I_{AC} denotes the alternating current (AC) component, θ_i indicates the illumination polarization angle, and β is the orientation angle of the fluorescent dipole.

The values of I_{DC} and I_{AC} can be obtained by solving the inverse of the matrix equation.

The modulation depth is calculated as:

$$ModulationDepth = \frac{2I_{AC}}{I_{AC} + I_{DC}} \tag{1-3}$$

The emission intensities from the yellow and red channels of the HiLo images are averaged and then used to compute the emission ratio, which reflects the emission properties of different lipid compositions. The resulting values are visualized as the **Polarity Map**.

$$Emission ratio = \frac{I_{redchannel}}{I_{yellowchannel}} \tag{1-4}$$

Image registration and light intensity correction

Since spectral ratio imaging involves calculating the ratio between two images acquired at different wavelengths, it is particularly sensitive to chromatic shifts, optical distortions, and misalignments—issues that become more pronounced in super-resolution imaging compared to diffraction-limited multicolor 3D imaging. To ensure accurate quantitative ratio measurements, precise spatial alignment of fine structures at the super-resolution level is essential.

To achieve this, we first acquired calibration reference images using fixed samples to correct for channel misalignment. Specifically, Alexa Fluor™ 555 Phalloidin (A34055, Invitrogen™) and Alexa Fluor™ Plus 647 Phalloidin (A30107, Invitrogen™) were used to label the actin cytoskeleton, enabling the acquisition of images of the same subcellular structures at different emission wavelengths. Subsequently, live-cell imaging of subcellular organelle dynamics was performed using the acquisition approach described above. Finally, Chromagnon software was employed to measure and compute a channel misalignment correction matrix, which was then applied to the captured multichannel live-cell images to generate precisely aligned datasets. The aligned two-channel images were then used to compute ratio images, providing quantitative spectral ratio information.

Computational Data

Table S1. Computed S1 emission wavelength for NR in DMSO using various functionals.

Functional	Computed S1 emission wavelength (nm)
TPSSh	648
PBE0	623
B3LYP	636
cam-B3LYP	623
LC-wPBE	621
wB97xD	628

Cartesian coordinates

JONR_S1_DMSO

C 5.992702 -1.390927 -0.010398

C 5.615557 -0.054381 -0.008606

C 4.265863 0.315793 -0.010196

C 3.268587 -0.698074 -0.003735

C 3.669547 -2.050317 -0.008040

C 5.011348 -2.392292 -0.009573

C 1.862144 -0.352847 -0.015563

C 1.546216 1.046194 0.000271

C 2.490257 2.041824 -0.011303

C -0.730291 0.460154 -0.023618

C -0.382151 -0.902548 -0.012802

H 7.043683 -1.659430 -0.011433

H 6.361801 0.732339 -0.007361

H 2.906195 -2.819006 -0.006540

H 5.301940 -3.437387 -0.010887

H 2.177341 3.080192 -0.006966

N 0.938320 -1.323551 -0.013355

C 3.898903 1.749425 0.003157

O 4.766997 2.652248 0.016946

O 0.228306 1.442843 -0.008196

C -2.061113 0.870116 0.012238

C -1.408308 -1.867486 -0.011482

C -2.734549 -1.487312 0.006583

C -3.093045 -0.106425 -0.013037

H -1.144452 -2.917951 0.000829

N -4.398268 0.276814 -0.100631

C -4.699021 1.680075 -0.340559

H -5.704838 1.889494 0.028161

H -4.668552 1.895014 -1.417344

C -3.693737 2.526021 0.409550

H -3.781449 2.371832 1.488278

H -3.818059 3.580748 0.174524

C -5.411834 -0.743450 -0.323373

H -6.364106 -0.378806 0.066442

H -5.524296 -0.935224 -1.399143

C -5.003785 -2.002083 0.411575

H -5.685022 -2.819725 0.186626

H -4.976977 -1.829459 1.491396

O -2.338976 2.195563 0.008238

O -3.696743 -2.449931 -0.020142

TS_O

C -4.521783 -3.903141 0.170022

C -4.568556 -2.520652 0.086521

C -3.386950 -1.761256 0.049669

C -2.132949 -2.433097 0.086976

C -2.109918 -3.838121 0.175548

C -3.286554 -4.565000 0.214007

C -0.895354 -1.678151 0.061830

C -1.028977 -0.255876 -0.048103

C -2.236549 0.409636 -0.080926

C 1.305649 -0.112537 -0.020440

C 1.407749 -1.494950 0.056963

H -5.443957 -4.473280 0.199225

H -5.522234 -2.008070 0.050765

H -1.151204 -4.340986 0.208051

H -3.252102 -5.646974 0.279071

H -2.248566 1.491480 -0.145582

N 0.277606 -2.305684 0.114434

C -3.440270 -0.310434 -0.049527

O -4.613587 0.264382 -0.089449

O 0.105198 0.519841 -0.077906

C 2.454830 0.705970 -0.116455
C 2.699837 -2.073736 0.088235
C 3.836956 -1.298565 0.028650
C 3.749833 0.111621 -0.049129
H 2.789696 -3.151603 0.158628
N 4.853191 0.904484 -0.028084
C 4.673707 2.338327 0.165463
H 5.556786 2.857317 -0.210434
H 4.561288 2.562110 1.234066
C 3.465029 2.791919 -0.617126
H 3.602764 2.652647 -1.691277
H 3.209935 3.825462 -0.398407
C 6.146312 0.281159 0.216817
H 6.922070 0.900241 -0.238226
H 6.335953 0.216843 1.295943
C 6.149582 -1.100450 -0.408946
H 7.062925 -1.634223 -0.155259
H 6.047998 -1.034010 -1.495685
H -4.589001 1.561175 -0.177329
O -4.697134 2.929847 -0.300386
C -5.024480 3.202205 -1.654054
H -5.120315 4.285979 -1.800231
H -4.256479 2.838387 -2.353984
H -5.981408 2.742496 -1.941283
H -3.385931 3.762914 0.159630
O -2.583380 4.302057 0.448543
C -2.772776 4.670490 1.813848
H -1.914869 5.272480 2.123482
H -3.682962 5.268696 1.950473
H -2.832854 3.792017 2.469626
O 2.280099 2.022042 -0.239553
O 5.067639 -1.903779 0.115655

JONR_S1_DCM

C 5.992423 -1.389576 -0.012090
C 5.614984 -0.052939 -0.009543
C 4.264960 0.315463 -0.010491
C 3.268808 -0.698522 -0.004257
C 3.669497 -2.050451 -0.009415
C 5.011833 -2.391406 -0.011770
C 1.861483 -0.354166 -0.014548
C 1.545298 1.045489 0.001821
C 2.487659 2.041597 -0.009774
C -0.730324 0.458764 -0.020493
C -0.381852 -0.904333 -0.009815
H 7.043601 -1.657353 -0.013590
H 6.359661 0.735301 -0.007968
H 2.904505 -2.817383 -0.008210
H 5.303138 -3.436337 -0.014007
H 2.174593 3.079698 -0.005793
N 0.939596 -1.325687 -0.011197
C 3.896707 1.748298 0.002621
O 4.763919 2.651892 0.014132
O 0.226384 1.442512 -0.005746
C -2.061042 0.868354 0.015346
C -1.407527 -1.868307 -0.009770
C -2.733916 -1.485558 0.009948
C -3.091758 -0.106025 -0.006404
H -1.145466 -2.919080 0.000704
N -4.401865 0.278786 -0.083519
C -4.695443 1.680204 -0.342801
H -5.702946 1.899088 0.016446
H -4.655454 1.885253 -1.421471
C -3.689222 2.531704 0.402828
H -3.780980 2.388210 1.482972
H -3.810623 3.584558 0.157963
C -5.408350 -0.743224 -0.330553
H -6.368903 -0.384862 0.045126
H -5.501772 -0.932443 -1.408872
C -5.006754 -2.006141 0.402772
H -5.683700 -2.824283 0.166060
H -4.992184 -1.839865 1.483924
O -2.337309 2.196327 0.007537
O -3.697040 -2.449174 -0.017415

NR_S1_DMSO

C 6.019732 -1.389292 0.051107
C 5.638558 -0.053607 0.032815
C 4.288298 0.312942 0.007216
C 3.293054 -0.704287 0.009845
C 3.698579 -2.055622 0.025685
C 5.041295 -2.393524 0.047944
C 1.888035 -0.363595 -0.028381
C 1.566175 1.031414 -0.027850
C 2.506432 2.031579 -0.037744
C -0.723176 0.446456 -0.065924
C -0.351559 -0.914836 -0.051345
H 7.071402 -1.654443 0.068925
H 6.382863 0.735009 0.036731
H 2.937550 -2.826615 0.023615

H 5.334836 -3.437701 0.062436
H 2.189697 3.068784 -0.045431
N 0.962015 -1.337772 -0.037579
C 3.916677 1.744800 0.000280
O 4.780226 2.652080 0.019805
O 0.245868 1.424036 -0.059943
C -2.041231 0.878160 -0.055265
C -1.392089 -1.863637 -0.065475
C -2.724286 -1.503636 -0.081371
C -3.068647 -0.112125 -0.079467
H -1.118765 -2.913922 -0.058785
N -4.382341 0.270349 -0.087668
C -4.756057 1.674412 -0.254389
H -5.772102 1.784598 0.125983
H -4.769932 1.915927 -1.327461
C -3.785148 2.585945 0.477966
H -3.841251 2.383891 1.551804
H -4.080361 3.624702 0.315954
C -5.470046 -0.707382 -0.155818
H -6.337267 -0.252700 0.326496
H -5.728469 -0.877329 -1.211706
C -5.088000 -2.019569 0.508527
H -5.908130 -2.729456 0.381908
H -4.944314 -1.858609 1.581491
C -3.806399 -2.549268 -0.123806
H -4.007477 -2.821368 -1.168235
H -3.458762 -3.454590 0.379178
C -2.368029 2.348758 -0.032117
H -1.640651 2.877314 0.586980
H -2.268356 2.755030 -1.046645

NR_S1_MeOH

C 4.397519 -4.068234 -0.095980
C 4.481884 -2.682593 -0.090856
C 3.326731 -1.888400 -0.080131
C 2.051586 -2.521680 -0.085020
C 1.987674 -3.929334 -0.086141
C 3.143309 -4.692951 -0.094474
C 0.837613 -1.733914 -0.058745
C 0.999492 -0.312615 -0.076095
C 2.221140 0.318728 -0.067422
C -1.352364 -0.099851 -0.045393
C -1.456624 -1.504095 -0.041615
H 5.302828 -4.665608 -0.102040
H 5.449341 -2.193533 -0.092844
H 1.014762 -4.405428 -0.084060
H 3.075179 -5.775434 -0.098314
H 2.262664 1.402279 -0.073440
N -0.357939 -2.340560 -0.045977
C 3.435581 -0.425222 -0.087754
O 4.576779 0.145002 -0.102884
O -0.111868 0.497943 -0.070545
C -2.447484 0.750617 -0.022504
C -2.755562 -2.050337 -0.024940
C -3.890357 -1.266177 -0.022611
C -3.748774 0.160592 -0.021354
H -2.849362 -3.131555 -0.019588
N -4.855422 0.962764 -0.015753
C -4.741796 2.414851 -0.157977
H -5.649865 2.851118 0.259882
H -4.711549 2.666702 -1.228454
C -3.500809 2.939909 0.543000
H -3.589140 2.760348 1.618739
H -3.434890 4.018663 0.386916
C -6.206584 0.403779 -0.097683
H -6.879716 1.123594 0.371123
H -6.492398 0.323455 -1.157029
C -6.292723 -0.956059 0.574503
H -7.303230 -1.349141 0.443389
H -6.111447 -0.844831 1.648120
H 4.771357 1.819283 0.008498
O 4.975371 2.789645 0.112361
C 5.688773 2.962713 1.343907
H 5.919813 4.024454 1.444491
H 5.087656 2.646776 2.203849
H 6.625591 2.395497 1.335354
H 3.538485 3.769594 0.030940
O 2.732284 4.342111 -0.011233
C 2.816535 5.132104 -1.200574
H 1.928231 5.765952 -1.238365
H 3.704929 5.775305 -1.195844
H 2.838890 4.507176 -2.101588
C -5.260680 -1.891862 -0.044312
H -5.544365 -2.103281 -1.083578
H -5.231423 -2.851356 0.477612
C -2.261412 2.244278 -0.008476
H -1.378491 2.500612 0.580301
H -2.066841 2.590831 -1.031684

JONR_S1_MeOH

C 4.216395 -4.219900 -0.160662
C 4.348494 -2.839378 -0.114680
C 3.220933 -2.006824 -0.084602
C 1.925971 -2.595534 -0.110885
C 1.812943 -3.999209 -0.153164
C 2.941320 -4.801326 -0.181002
C 0.738398 -1.766257 -0.063477
C 0.955579 -0.348907 -0.038239
C 2.198260 0.237898 -0.010665
C -1.376599 -0.071725 -0.016574
C -1.550795 -1.459003 -0.036574
H 5.100524 -4.847970 -0.181408
H 5.331718 -2.383270 -0.099083
H 0.824057 -4.440941 -0.166647
H 2.836415 -5.880265 -0.216710
H 2.277552 1.318928 0.011252
N -0.471742 -2.331891 -0.065038
C 3.382931 -0.547993 -0.049142
O 4.546720 -0.023786 -0.047451
O -0.129333 0.498100 -0.015571
C -2.465919 0.800649 0.045532
C -2.864295 -1.979145 -0.013592
C -3.955724 -1.141261 0.029215
C -3.787083 0.272211 0.034981
H -3.007356 -3.053047 -0.024178
N -4.855483 1.112763 -0.017271
C -4.617047 2.531429 -0.248644
H -5.467709 3.096644 0.135891
H -4.523739 2.725386 -1.325429
C -3.362617 2.944254 0.486512
H -3.478538 2.829258 1.566776
H -3.083104 3.966951 0.245581
C -6.179748 0.542991 -0.223721
H -6.921449 1.226197 0.194646
H -6.375254 0.428247 -1.298166
C -6.252163 -0.794691 0.481960
H -7.191894 -1.296806 0.262335
H -6.139039 -0.672771 1.562798
H 4.819479 1.630694 0.084268
O 5.063588 2.590759 0.202928
C 5.376066 2.798637 1.586078
H 5.647296 3.848361 1.709509
H 4.517666 2.575396 2.229582
H 6.223361 2.176182 1.893067
H 3.778875 3.624867 -0.348559
O 3.052363 4.228039 -0.645248
C 3.639440 5.486256 -0.986648
H 2.837312 6.141401 -1.332737
H 4.124640 5.955792 -0.122031
H 4.377774 5.382891 -1.791131
O -2.230326 2.129215 0.072045
O -5.213819 -1.681985 0.002609

JONR_PT_N

C 5.865925 -0.070882 -0.454278
C 5.314714 -1.312573 -0.200507
C 3.923744 -1.490474 -0.120442
C 3.066107 -0.365904 -0.295946
C 3.651514 0.894070 -0.559242
C 5.023169 1.037237 -0.633615
C 1.643911 -0.551725 -0.223781
C 1.151485 -1.847484 0.033991
C 1.967201 -2.945567 0.202905
C -1.075050 -1.028773 -0.076362
C -0.610990 0.273569 -0.298256
H 6.942180 0.048462 -0.514674
H 5.950157 -2.179582 -0.060215
H 3.026132 1.767203 -0.700183
H 5.448229 2.014853 -0.834588
H 1.518409 -3.912210 0.399454
N 0.753725 0.478976 -0.367950
C 3.380182 -2.829736 0.157857
O 4.147470 -3.826658 0.339215
O -0.206031 -2.082648 0.112175
C -2.433803 -1.296489 0.018107
C -1.531590 1.310447 -0.483863
C -2.886629 1.044378 -0.398592
C -3.371824 -0.261351 -0.158095
H -1.188641 2.320965 -0.664905
N -4.729320 -0.522749 -0.084806
C -5.101732 -1.776491 0.559573
H -6.139340 -2.004385 0.307012
H -5.013299 -1.707802 1.652139
C -4.203907 -2.874917 0.030043
H -4.329139 -2.993989 -1.049482
H -4.401195 -3.820955 0.530758
C -5.590511 0.636302 0.116139
H -6.615098 0.357974 -0.139056
H -5.568710 0.976529 1.160250

C -5.124958 1.743475 -0.805497
H -5.700506 2.653883 -0.648018
H -5.201065 1.435241 -1.852087
H 1.071550 1.449529 -0.508335
O 1.167560 3.294514 -0.532723
C 1.809405 4.198132 -1.441810
H 1.358110 5.194205 -1.384485
H 2.882548 4.278697 -1.238504
H 1.666359 3.802410 -2.448175
H 1.304094 3.610427 0.389903
O 1.548510 3.954900 2.085184
C 1.220807 2.908374 2.901083
H 0.303542 2.400359 2.578621
H 2.033493 2.169025 2.755586
H 1.202866 3.177367 3.961781
O -3.751652 2.104669 -0.525472
O -2.811424 -2.581465 0.304124

NR_S1_DCM

C 6.020316 -1.387389 0.046999
C 5.638667 -0.051682 0.029533
C 4.287964 0.312916 0.006512
C 3.294384 -0.704701 0.010284
C 3.699683 -2.055607 0.025359
C 5.042983 -2.392384 0.045250
C 1.887935 -0.365088 -0.025880
C 1.565966 1.031192 -0.024265
C 2.504763 2.031633 -0.034755
C -0.722384 0.444704 -0.062558
C -0.351161 -0.915336 -0.048342
H 7.072276 -1.651597 0.062927
H 6.381247 0.738579 0.032297
H 2.937051 -2.824907 0.024175
H 5.337534 -3.436314 0.059026
H 2.188308 3.068739 -0.042017
N 0.964643 -1.339055 -0.034488
C 3.914963 1.744178 0.000053
O 4.778427 2.651801 0.016845
O 0.244362 1.423202 -0.054352
C -2.041879 0.876401 -0.053638
C -1.391004 -1.863560 -0.063306
C -2.724209 -1.503285 -0.080046
C -3.067642 -0.113099 -0.078167
H -1.116306 -2.913393 -0.056730
N -4.384452 0.271274 -0.085217
C -4.754958 1.674676 -0.259578
H -5.772543 1.788686 0.116537
H -4.765276 1.914393 -1.333483
C -3.785414 2.588491 0.473436
H -3.845544 2.389858 1.547666
H -4.079100 3.627240 0.307807
C -5.470425 -0.706674 -0.160460
H -6.341205 -0.253722 0.317878
H -5.725316 -0.877944 -1.217401
C -5.090778 -2.019578 0.505600
H -5.910703 -2.729613 0.377252
H -4.950343 -1.858333 1.578894
C -3.806559 -2.549918 -0.122303
H -4.004688 -2.827323 -1.165968
H -3.460711 -3.453358 0.385376
C -2.366127 2.348059 -0.030528
H -1.639740 2.873821 0.592263
H -2.259086 2.755934 -1.043719

JONR_PT_O

C 4.449439 -4.026122 -0.143400
C 4.525664 -2.650462 -0.105317
C 3.352335 -1.862459 -0.066180
C 2.079503 -2.505606 -0.076352
C 2.034644 -3.916471 -0.112375
C 3.193512 -4.663004 -0.147194
C 0.864326 -1.724721 -0.026214
C 1.014324 -0.330545 -0.006372
C 2.251674 0.316619 0.005698
C -1.328240 -0.121741 0.052381
C -1.437043 -1.530529 -0.002846
H 5.357245 -4.619244 -0.171205
H 5.489778 -2.156496 -0.103126
H 1.066301 -4.401784 -0.115202
H 3.138026 -5.746122 -0.176876
H 2.283463 1.398902 0.013473
N -0.343580 -2.343483 -0.032496
C 3.413725 -0.428804 -0.040068
O 4.642647 0.133966 -0.049565
O -0.091268 0.488564 0.037693
C -2.442200 0.703832 0.064865
C -2.734061 -2.083092 0.016897
C -3.847615 -1.268693 0.036567
C -3.733663 0.142941 0.073449
H -2.853802 -3.159408 -0.015604

N -4.855202 0.954056 0.106813
C -4.644273 2.350712 -0.252839
H -5.503196 2.932837 0.087611
H -4.544278 2.475164 -1.339593
C -3.393625 2.842602 0.445837
H -3.504417 2.776983 1.531778
H -3.160867 3.867031 0.160827
C -6.110182 0.324862 -0.284834
H -6.936674 0.950442 0.058695
H -6.182179 0.215966 -1.375526
C -6.192744 -1.030596 0.383727
H -7.088226 -1.567408 0.075249
H -6.182705 -0.926335 1.472623
H 4.595434 1.138982 -0.016285
O 4.765325 2.762861 0.036883
C 5.223842 3.272372 1.300620
H 5.462295 4.337122 1.221554
H 4.474430 3.123975 2.084631
H 6.128684 2.722521 1.560865
H 3.945617 3.248831 -0.219148
O 2.517213 4.144660 -0.662141
C 2.769112 5.481718 -0.801309
H 2.012292 5.992881 -1.406035
H 2.681314 5.890316 0.225159
H 3.791541 5.694260 -1.134099
O -2.240459 2.062726 0.048372
O -5.084623 -1.870314 -0.014795

TS_N

C -5.904947 -0.012805 -0.327706
C -5.388353 -1.292766 -0.198227
C -4.008352 -1.509680 -0.108959
C -3.124163 -0.393433 -0.148896
C -3.671005 0.902838 -0.283876
C -5.038065 1.087041 -0.377881
C -1.703766 -0.609366 -0.054688
C -1.245334 -1.958878 -0.006059
C -2.082042 -3.045815 0.030469
C 0.979368 -1.175910 -0.013992
C 0.550437 0.141101 -0.056412
H -6.977051 0.136585 -0.395113
H -6.047577 -2.152705 -0.169568
H -3.015256 1.764464 -0.322748
H -5.436965 2.089327 -0.488669
H -1.660257 -4.042626 0.086362
N -0.819834 0.412412 -0.046620
C -3.496205 -2.890029 -0.015776
O -4.283583 -3.879296 0.016654
O 0.108341 -2.223570 0.048392
C 2.351032 -1.484072 0.031898
C 1.499415 1.180741 -0.122582
C 2.849459 0.895472 -0.091862
C 3.312659 -0.439031 -0.039474
H 1.168008 2.209103 -0.191743
N 4.642034 -0.743820 -0.097257
C 5.012419 -2.128697 -0.360745
H 6.041821 -2.284798 -0.033726
H 4.947792 -2.345346 -1.435082
C 4.098955 -3.035655 0.429917
H 4.213768 -2.881963 1.505116
H 4.258349 -4.081511 0.179388
C 5.574742 0.332508 -0.401210
H 6.555660 0.066555 -0.001779
H 5.662499 0.467825 -1.487208
C 5.078832 1.606507 0.253703
H 5.699435 2.453420 -0.031290
H 5.066978 1.505307 1.342386
O 2.700316 -2.777264 0.102658
O 3.744269 1.931700 -0.201020
H -1.078808 1.489727 0.233704
O -1.143516 2.804749 0.689723
C -1.690542 2.917582 1.975771
H -1.245696 3.757029 2.528046
H -2.779513 3.096425 1.931674
H -1.543247 1.997973 2.562140
H -1.276291 4.198391 -0.203925
O -1.376181 5.034452 -0.743466
C -0.216327 5.838144 -0.525389
H -0.111942 6.124532 0.528886
H 0.700628 5.323866 -0.840037
H -0.324204 6.747330 -1.121353

Reference

1. Frisch, M. J.; Trucks, G. W.; Schlegel, H. B.; Scuseria, G. E.; Robb, M. A.; Cheeseman, J. R.; Scalmani, G.; Barone, V.; Petersson, G. A.; Nakatsuji, H.; Li, X.; Caricato, M.; Marenich, A. V.; Bloino, J.; Janesko, B. G.; Gomperts, R.; Mennucci, B.; Hratchian, H. P.; Ortiz, J. V.; Izmaylov, A. F.; Sonnenberg, J. L.; Williams; Ding, F.; Lipparini, F.; Egidi, F.; Goings, J.; Peng, B.; Petrone, A.; Henderson, T.; Ranasinghe, D.; Zakrzewski, V. G.; Gao, J.; Rega, N.; Zheng, G.; Liang, W.; Hada, M.; Ehara, M.; Toyota, K.; Fukuda, R.; Hasegawa, J.; Ishida, M.; Nakajima, T.; Honda, Y.; Kitao, O.; Nakai, H.; Vreven, T.; Throssell, K.; Montgomery Jr., J. A.; Peralta, J. E.; Ogliaro, F.; Bearpark, M. J.; Heyd, J. J.; Brothers, E. N.; Kudin, K. N.; Staroverov, V. N.; Keith, T. A.; Kobayashi, R.; Normand, J.; Raghavachari, K.; Rendell, A. P.; Burant, J. C.; Iyengar, S. S.; Tomasi, J.; Cossi, M.; Millam, J. M.; Klene, M.; Adamo, C.; Cammi, R.; Ochterski, J. W.; Martin, R. L.; Morokuma, K.; Farkas, O.; Foresman, J. B.; Fox, D. J. Gaussian 16 Rev. C.01, Wallingford, CT, 2016.
2. J. M. Tao, J. P. Perdew, V. N. Staroverov, and G. E. Scuseria, “Climbing the density functional ladder: Nonempirical meta-generalized gradient approximation designed for molecules and solids,” *Phys. Rev. Lett.*, 2003, 91, 146401.
3. A. Schaefer, C. Huber, and R. Ahlrichs, “Fully optimized contracted Gaussian-basis sets of triple zeta valence quality for atoms Li to Kr,” *J. Chem. Phys.*, 1994, 100, 5829-35.
4. Marenich, A. V.; Cramer, C. J.; Truhlar, D. G., Universal solvation model based on solute electron density and on a continuum model of the solvent defined by the bulk dielectric constant and atomic surface tensions. *J. Phys. Chem. B,* 2009, 113 (18), 6378-6396.
5. Legault, C., CYLview, 1.0 b, Université de Sherbrooke. Sherbrooke, QC: http://www. cylview. org 2009.
6. Humphrey, W., Dalke, A. and Schulten, K., "VMD - Visual Molecular Dynamics", *J. Molec. Graphics*, 1996, 14, 33-38.
7. Tian Lu, Feiwu Chen, Multiwfn: A Multifunctional Wavefunction Analyzer, *J. Comput. Chem*. 2012, 33, 580-592.

NMR Spectra

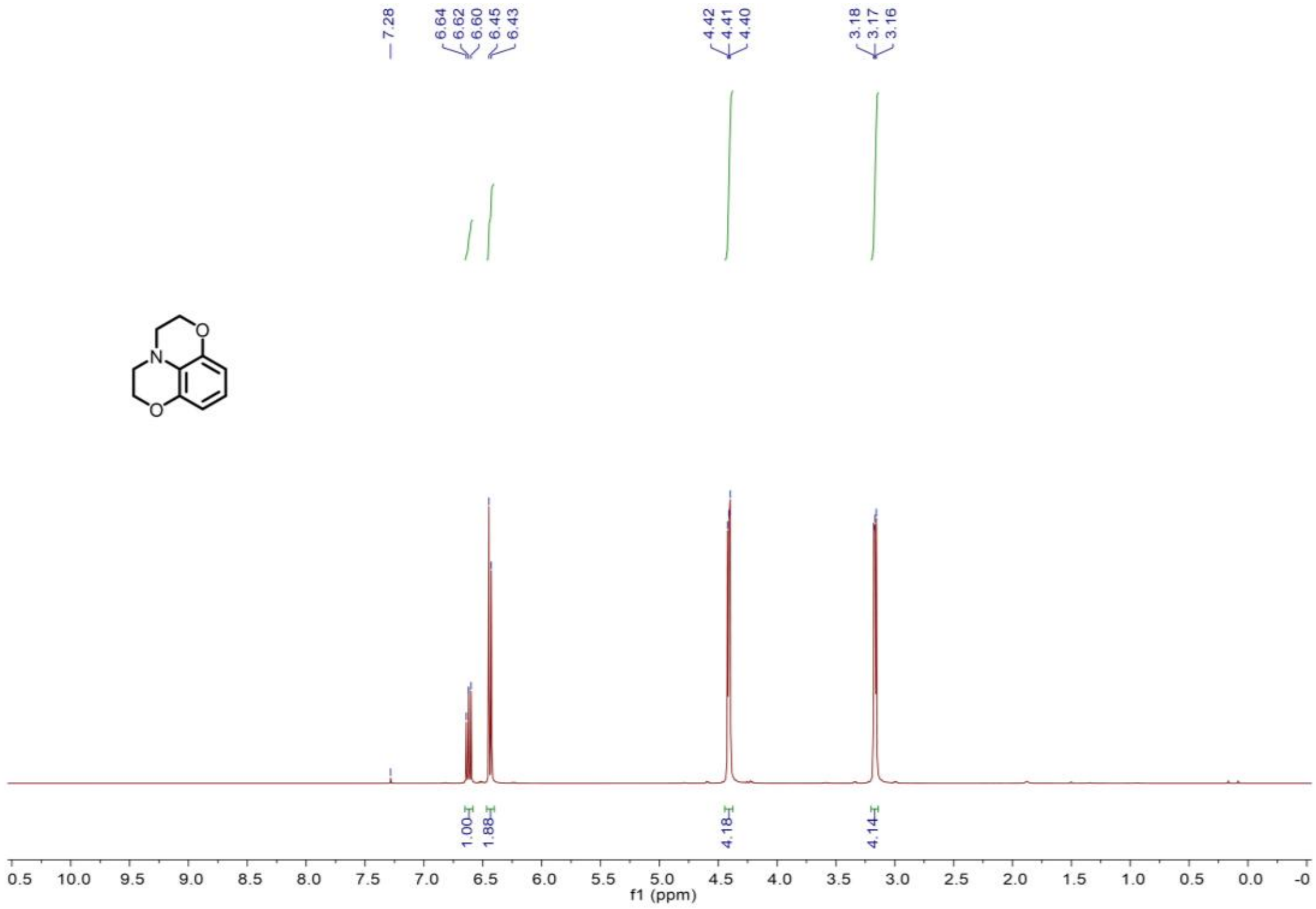
7.28
6.64
6.62
6.60
6.45
6.43
4.42
4.41
4.40
3.18
3.17
3.16
1.00
1.88
4.18
4.14
f1 (ppm)

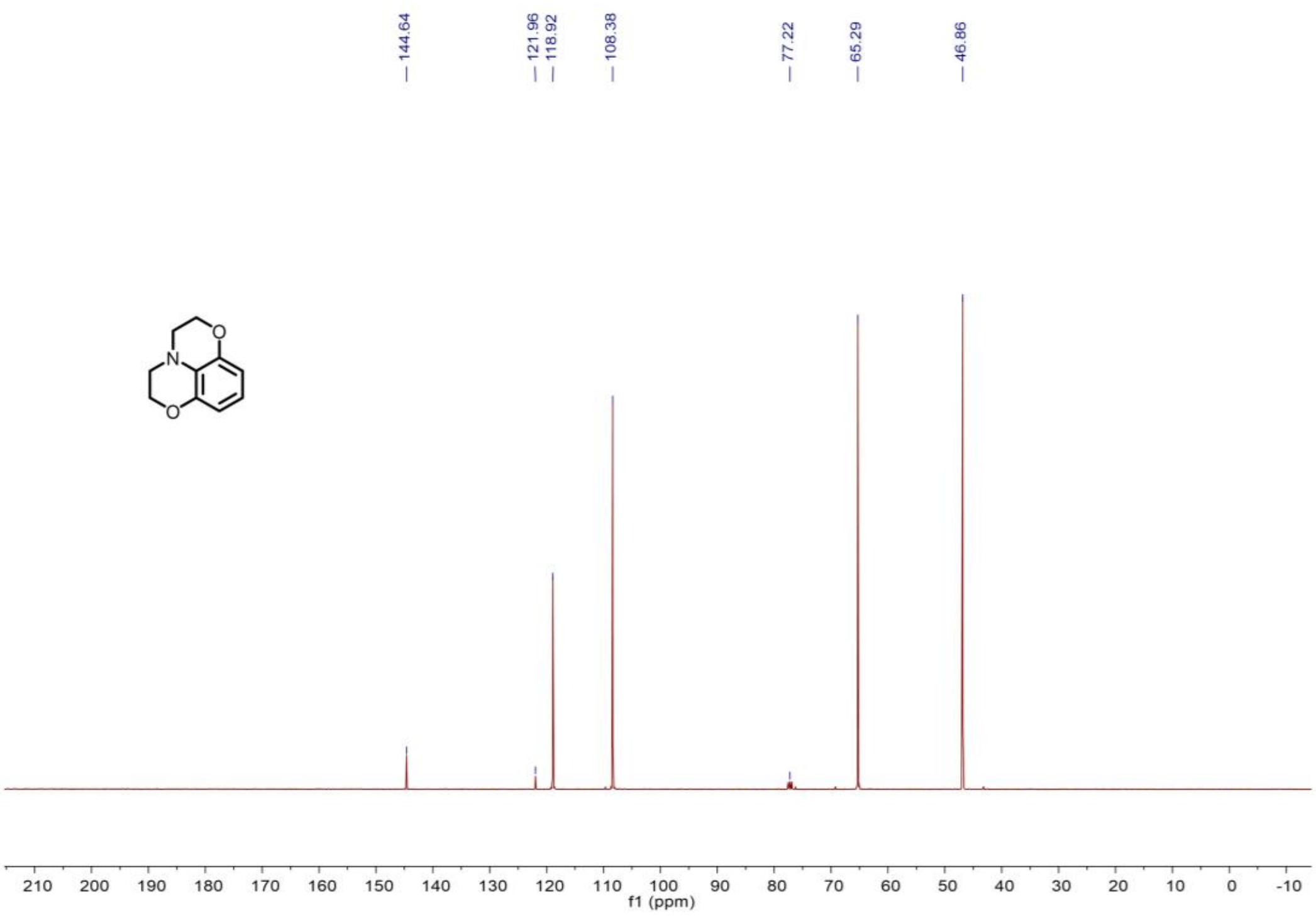
144.64
121.96
118.92
108.38
77.22
65.29
46.86
f1 (ppm)

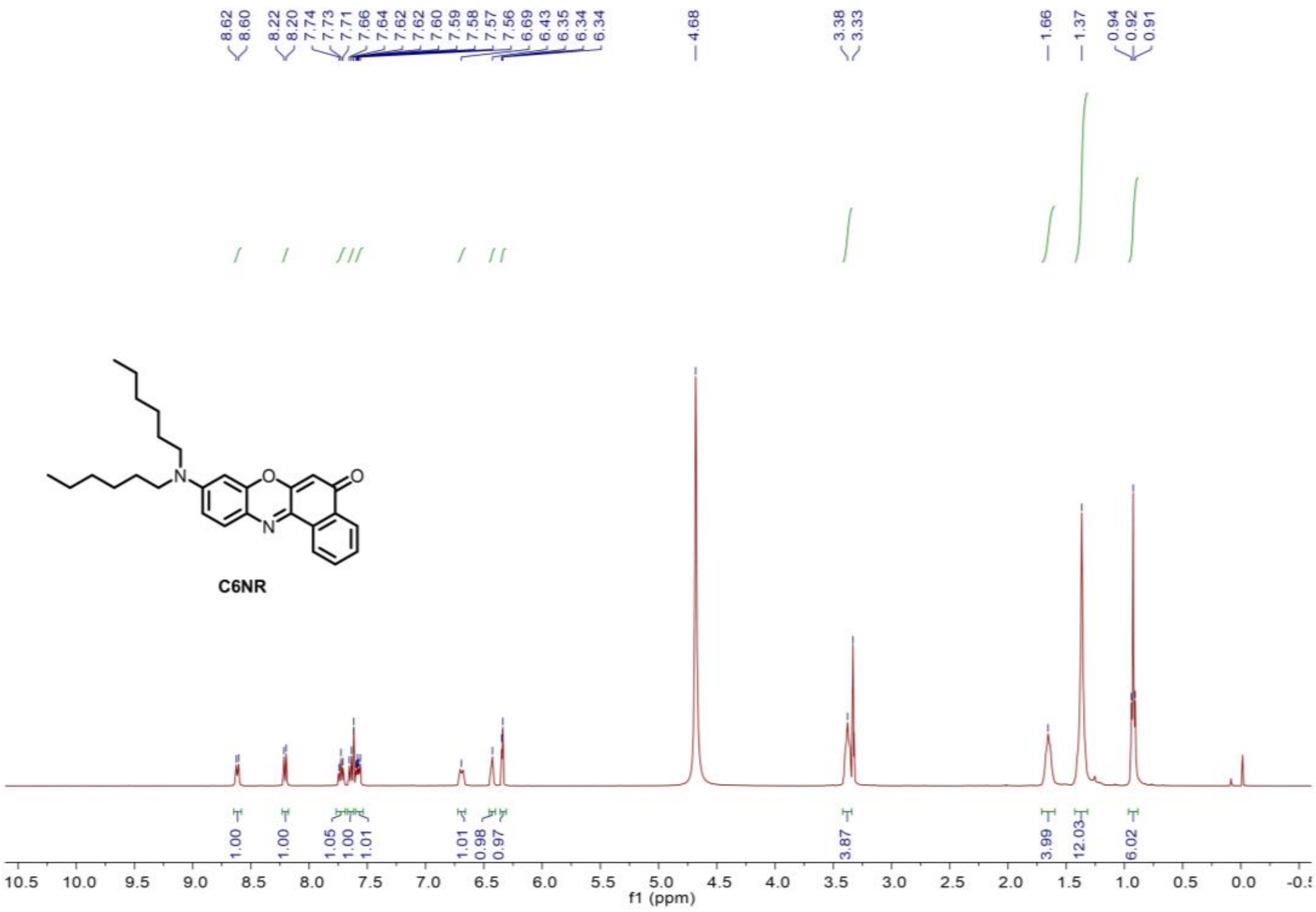
8.62
8.60
8.22
8.20
7.74
7.73
7.71
7.66
7.64
7.62
7.62
7.60
7.59
7.58
7.57
7.56
6.69
6.43
6.35
6.34
6.34
4.68
3.38
3.33
1.66
1.37
0.94
0.92
0.91
C6NR
1.00
1.00
1.05
1.00
1.01
1.01
0.98
0.97
3.87
3.99
12.03
6.02
f1 (ppm)

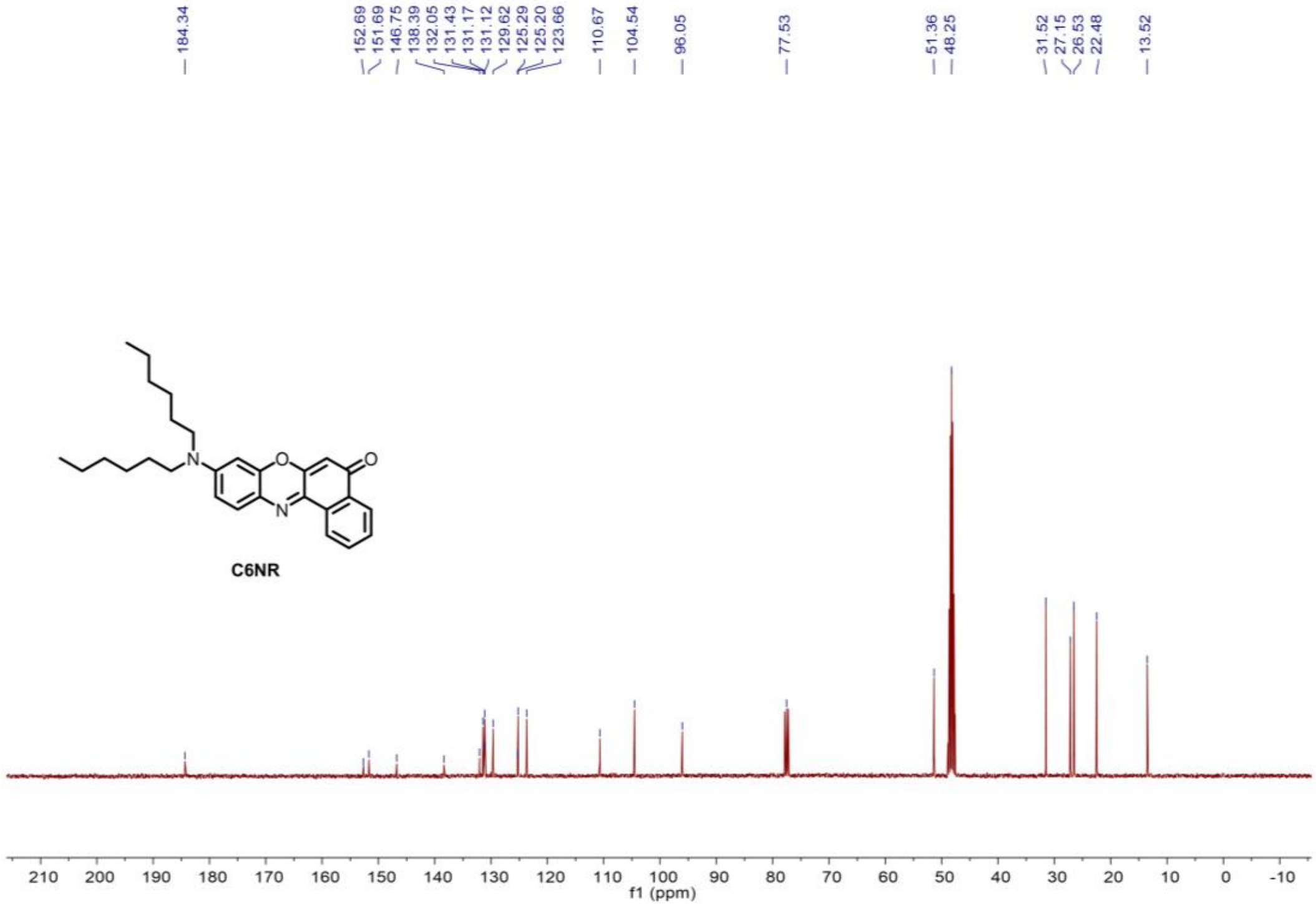
184.34
152.69
151.69
146.75
138.39
132.05
131.43
131.17
131.12
129.62
125.29
125.20
123.66
110.67
104.54
96.05
77.53
51.36
48.25
31.52
27.15
26.53
22.48
13.52
C6NR
f1 (ppm)

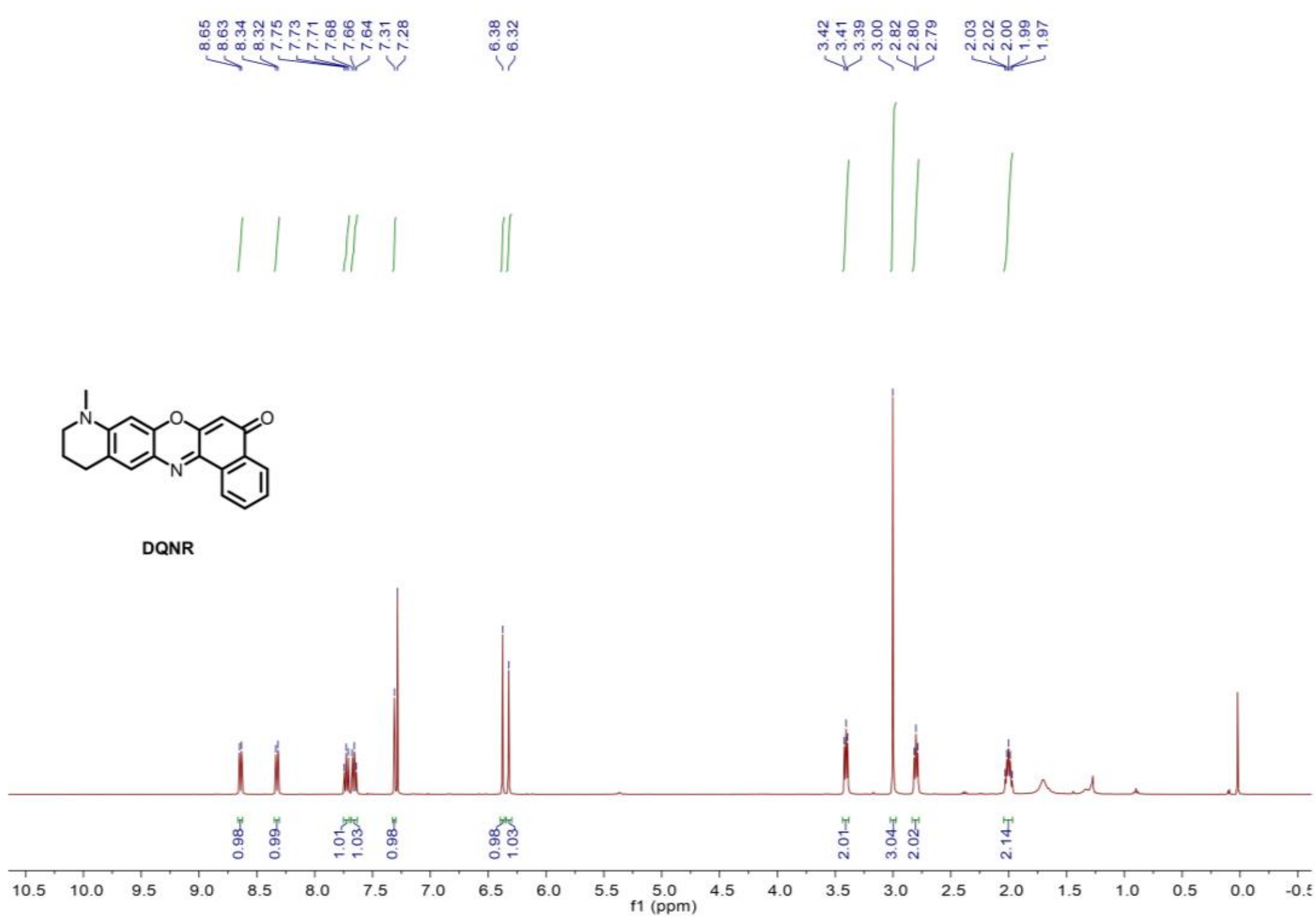
8.65 8.63 8.34 8.32 7.75 7.73 7.71 7.68 7.66 7.64 7.31 7.28
6.38 6.32
3.42 3.41 3.39 3.00 2.82 2.80 2.79
2.03 2.02 2.00 1.99 1.97
DQNR
0.98 0.99 1.01 1.03 0.98 0.98 1.03 2.01 3.04 2.02 2.14
f1 (ppm)

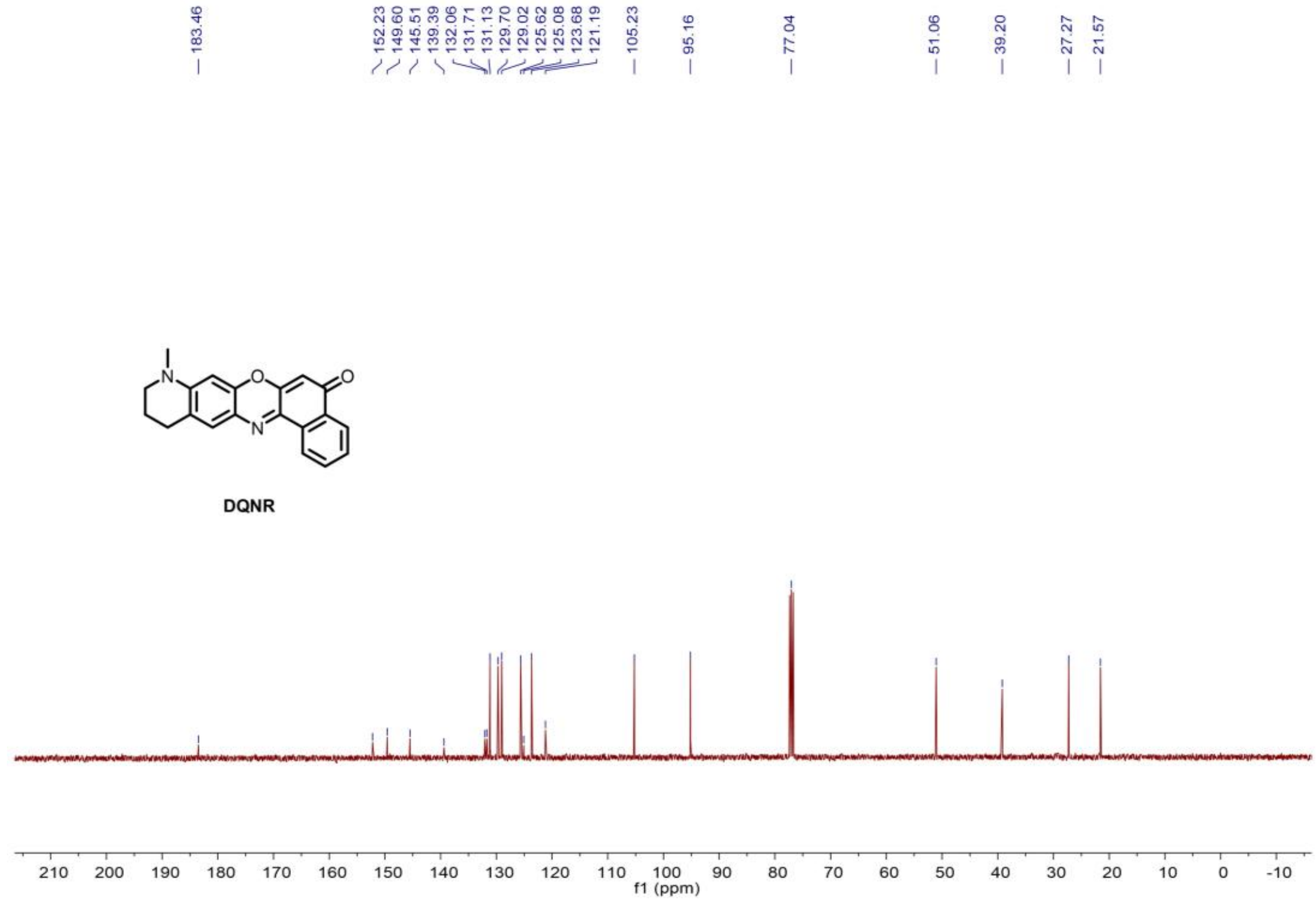
183.46
152.23 149.60 145.51 139.39 132.06 131.71 131.13 129.70 129.02 125.62 125.08 123.68 121.19
105.23
95.16
77.04
51.06
39.20
27.27
21.57
DQNR
f1 (ppm)

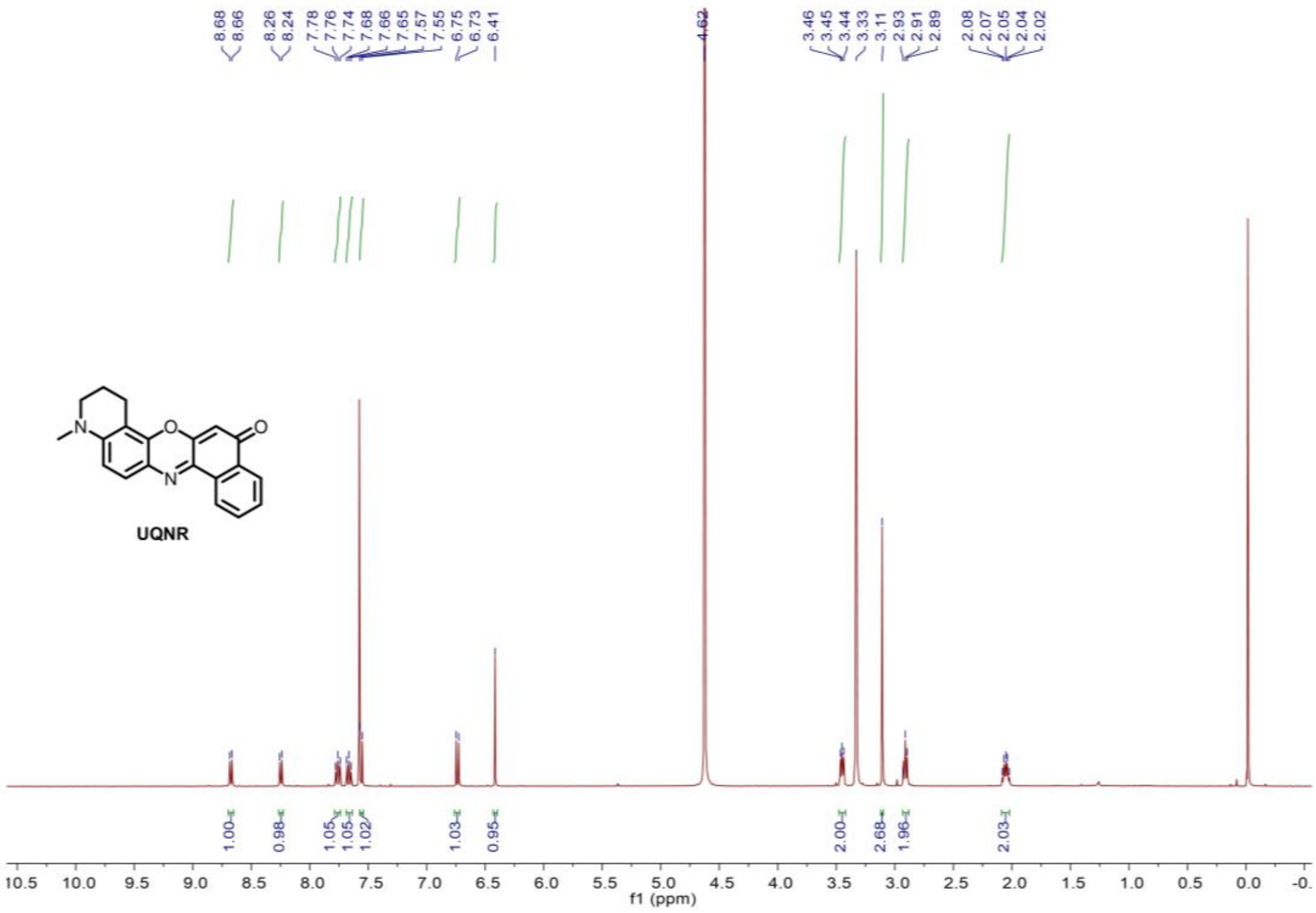
8.68
8.66
8.26
8.24
7.78
7.76
7.74
7.68
7.66
7.65
7.57
7.55
6.75
6.73
6.41
4.62
3.46
3.45
3.44
3.33
3.11
2.93
2.91
2.89
2.08
2.07
2.05
2.04
2.02
UQNR
1.00
0.98
1.05
1.05
1.02
1.03
0.95
2.00
2.68
1.96
2.03
f1 (ppm)

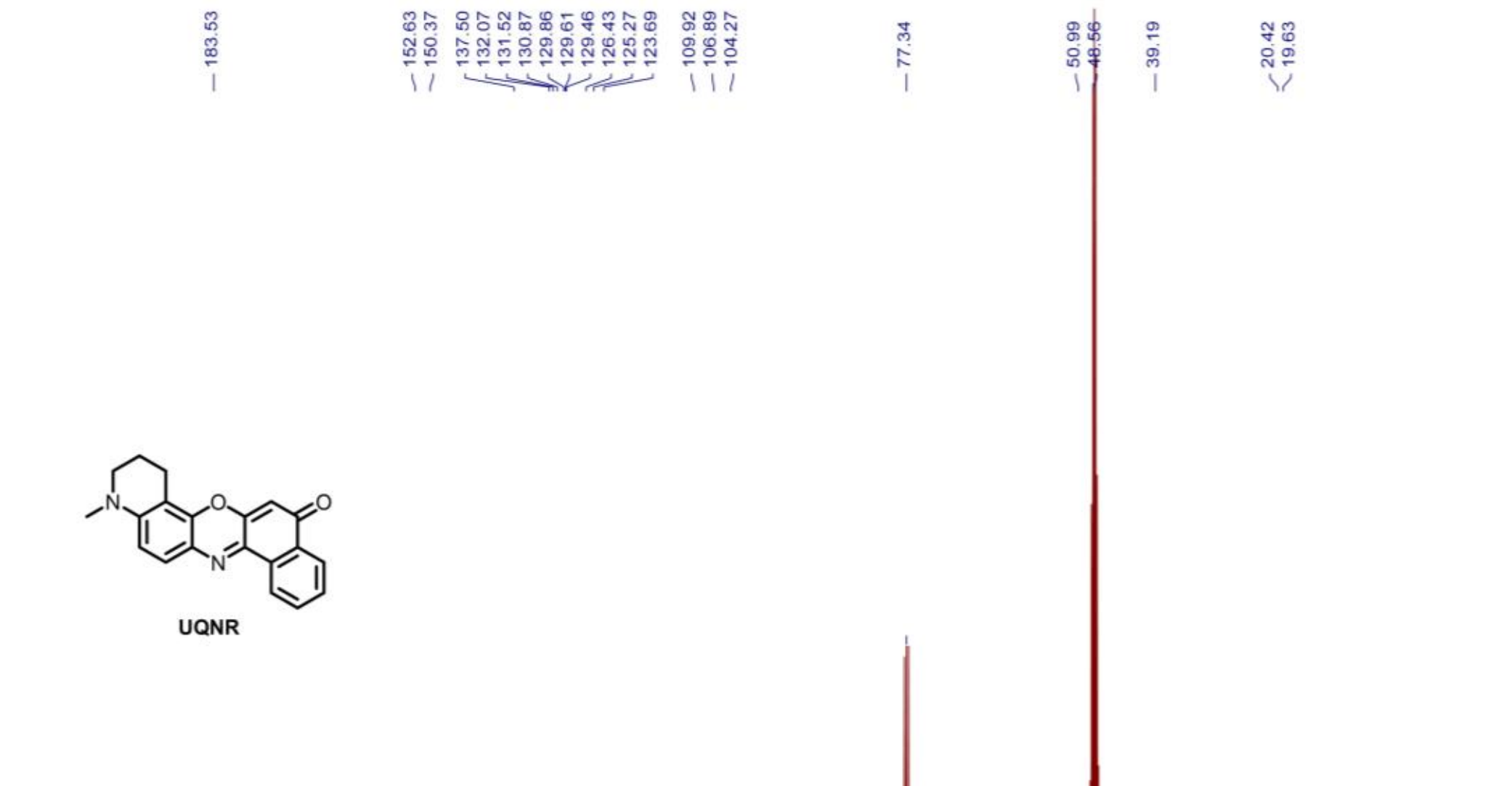
183.53
152.63
150.37
137.50
132.07
131.52
130.87
129.86
129.61
129.46
126.43
125.27
123.69
109.92
106.89
104.27
77.34
50.99
48.56
39.19
20.42
19.63
UQNR
f1 (ppm)

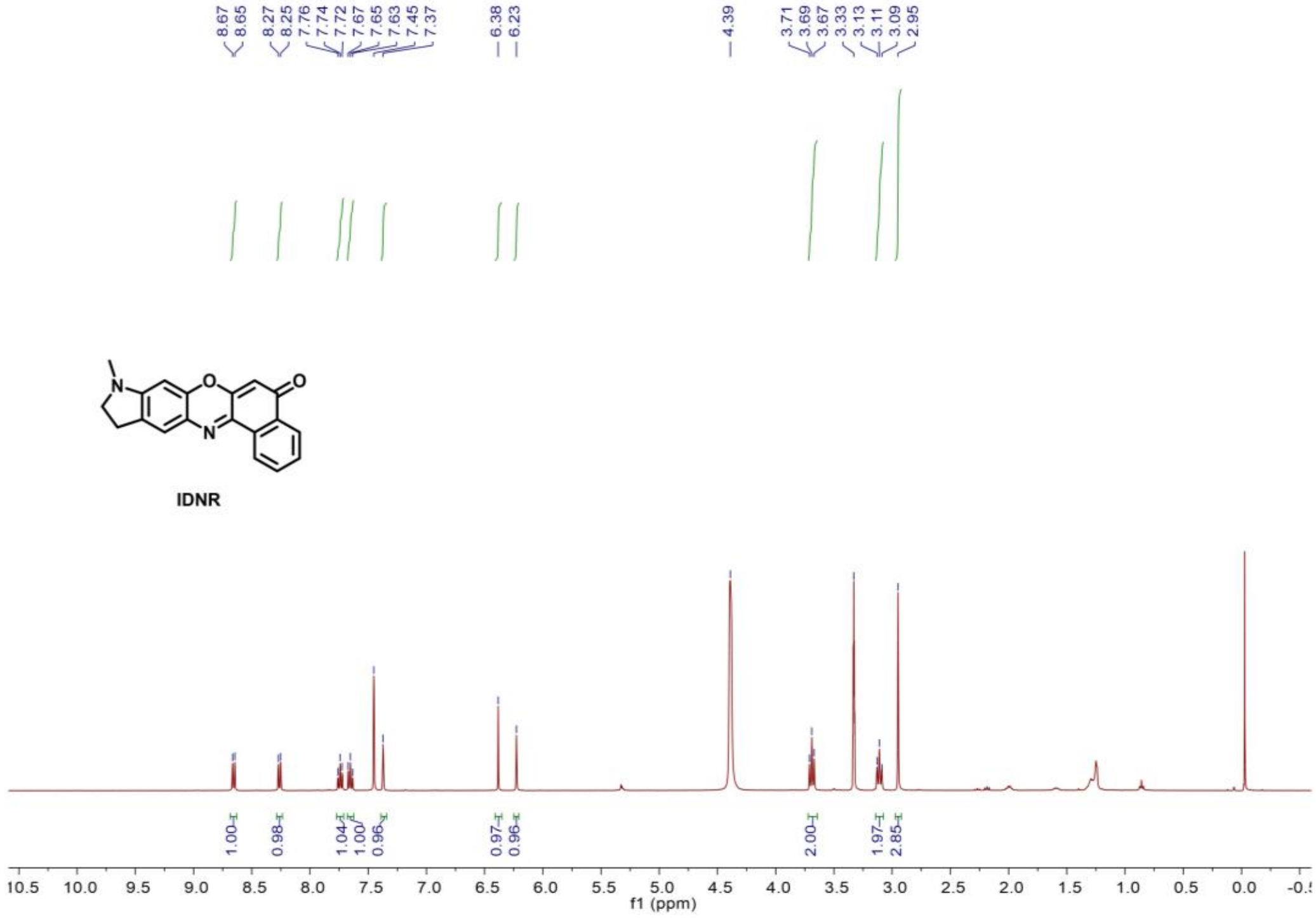
IDNR
f1 (ppm)

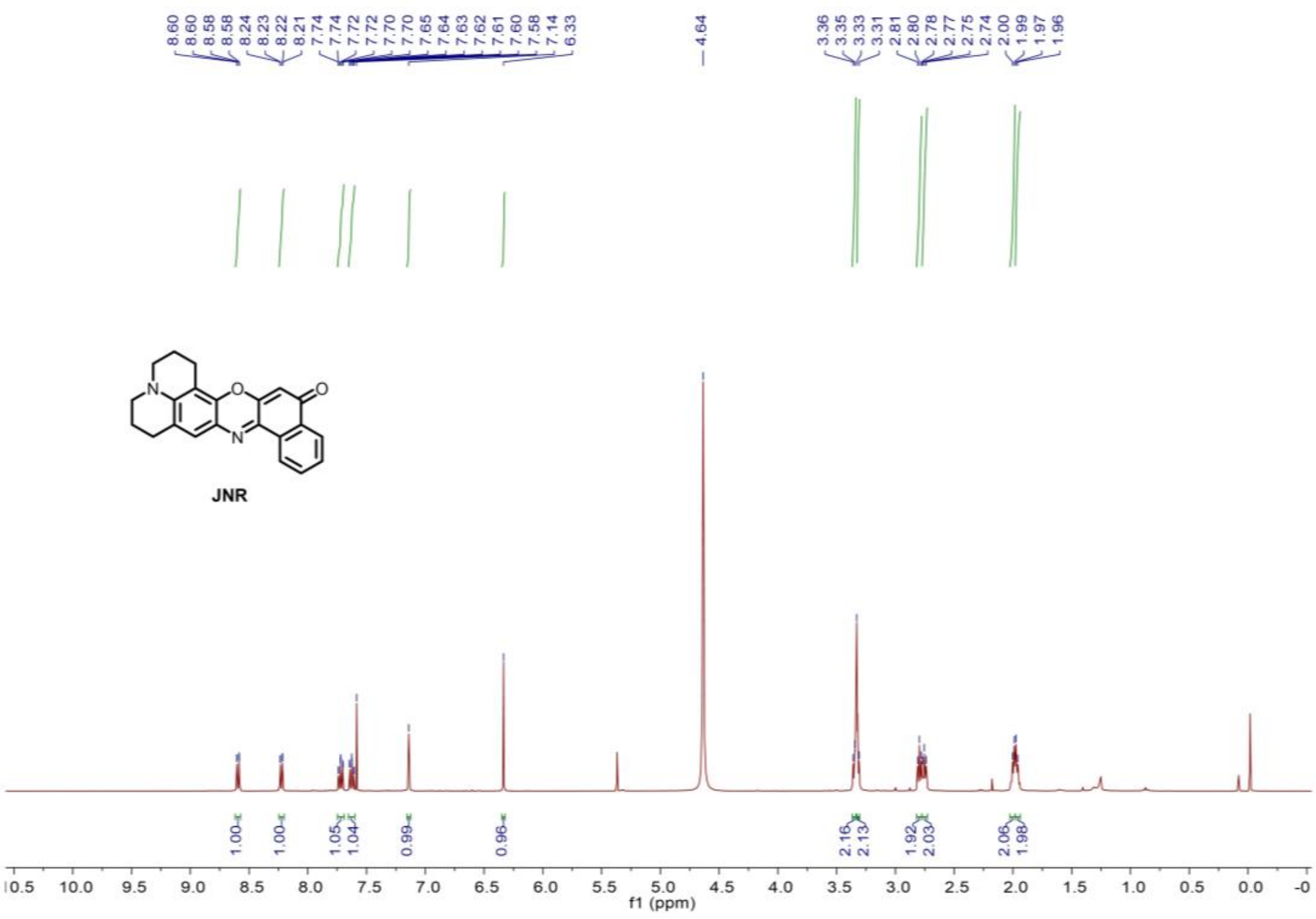
JNR
f1 (ppm)

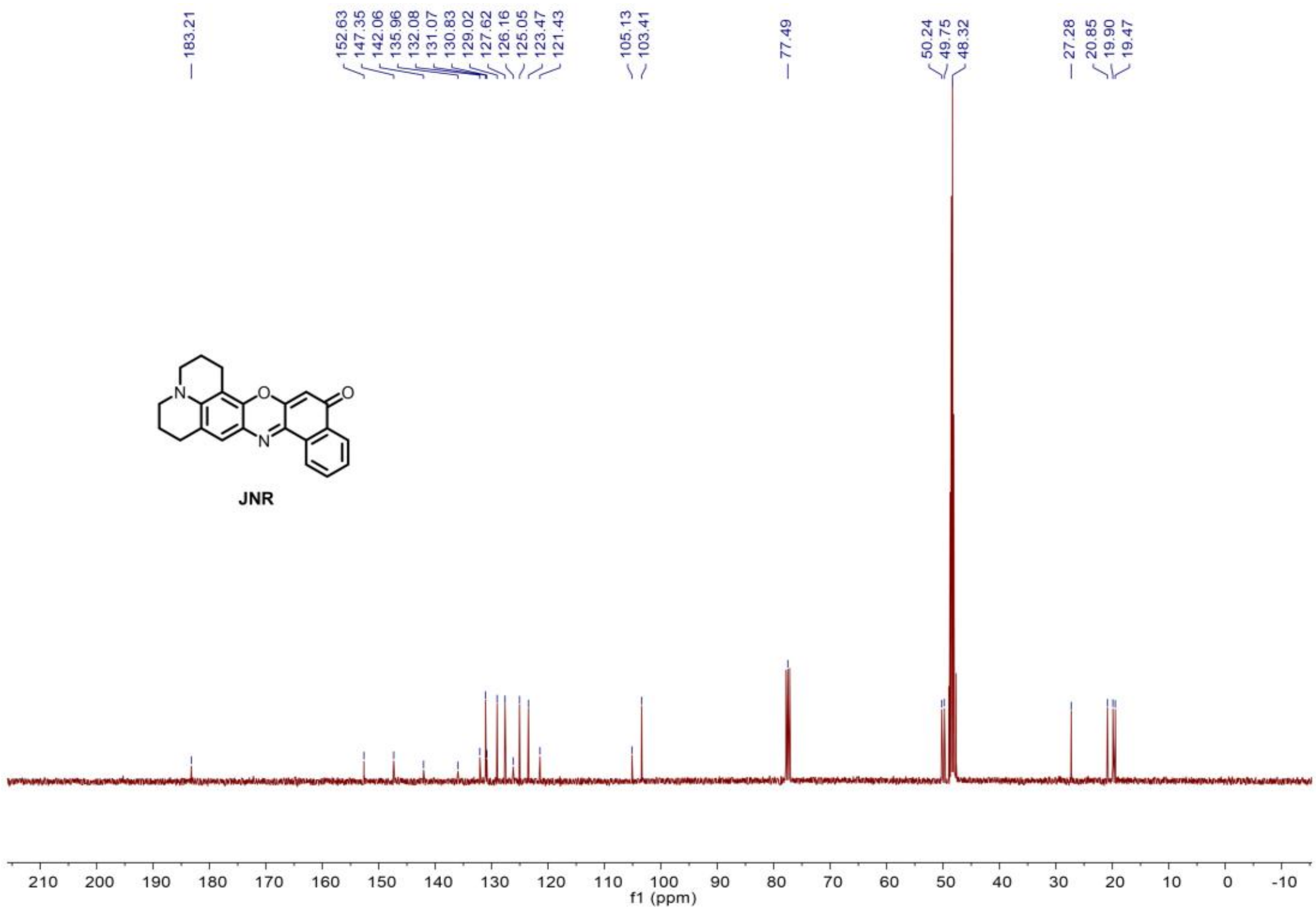
183.21
152.63
147.35
142.06
135.96
132.08
131.07
130.83
129.02
127.62
126.16
125.05
123.47
121.43
105.13
103.41
77.49
50.24
49.75
48.32
27.28
20.85
19.90
19.47
JNR
f1 (ppm)

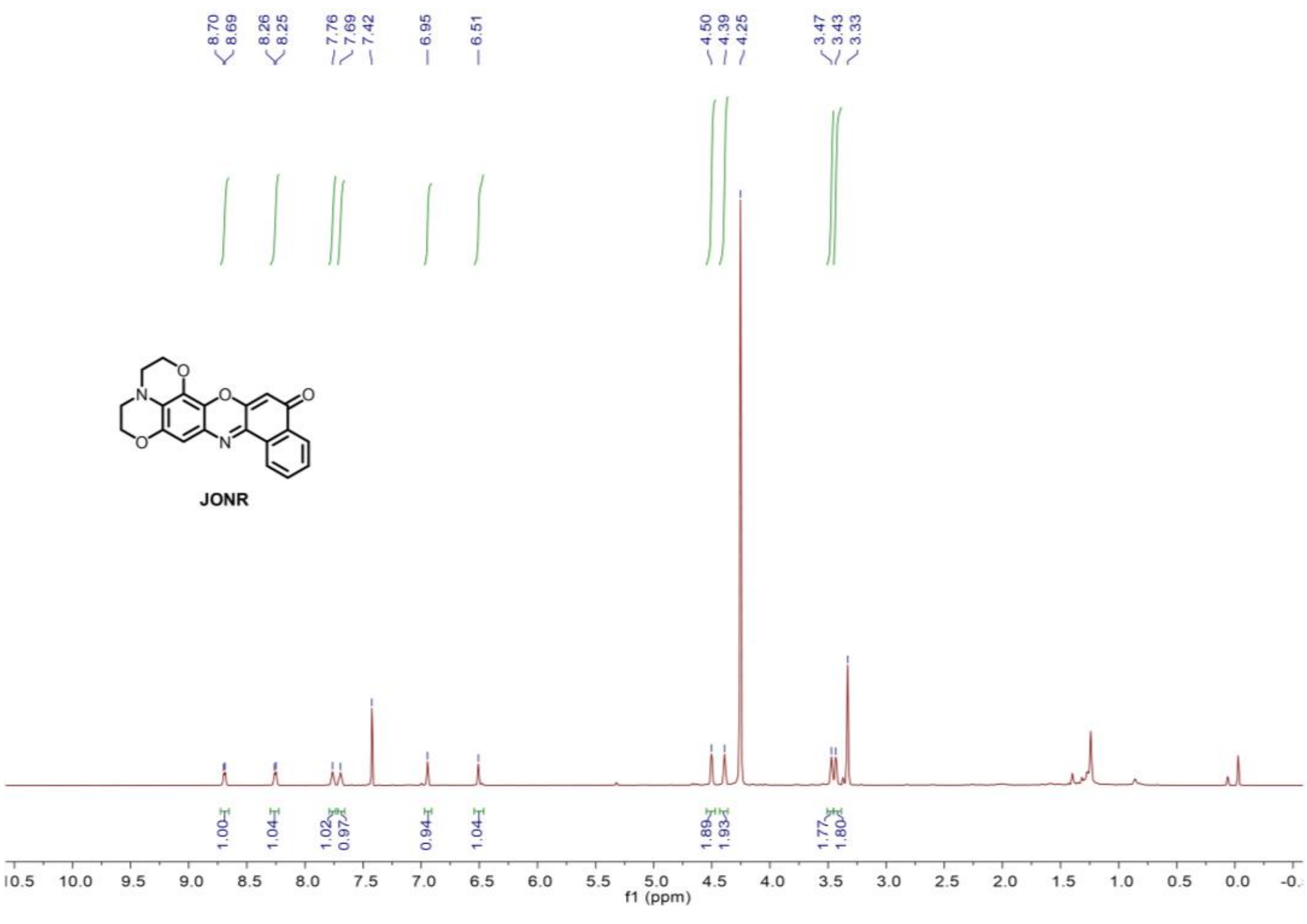
8.70
8.69
8.26
8.25
7.76
7.69
7.42
6.95
6.51
4.50
4.39
4.25
3.47
3.43
3.33
JONR
1.00
1.04
1.02
0.97
0.94
1.04
1.89
1.93
1.77
1.80
f1 (ppm)

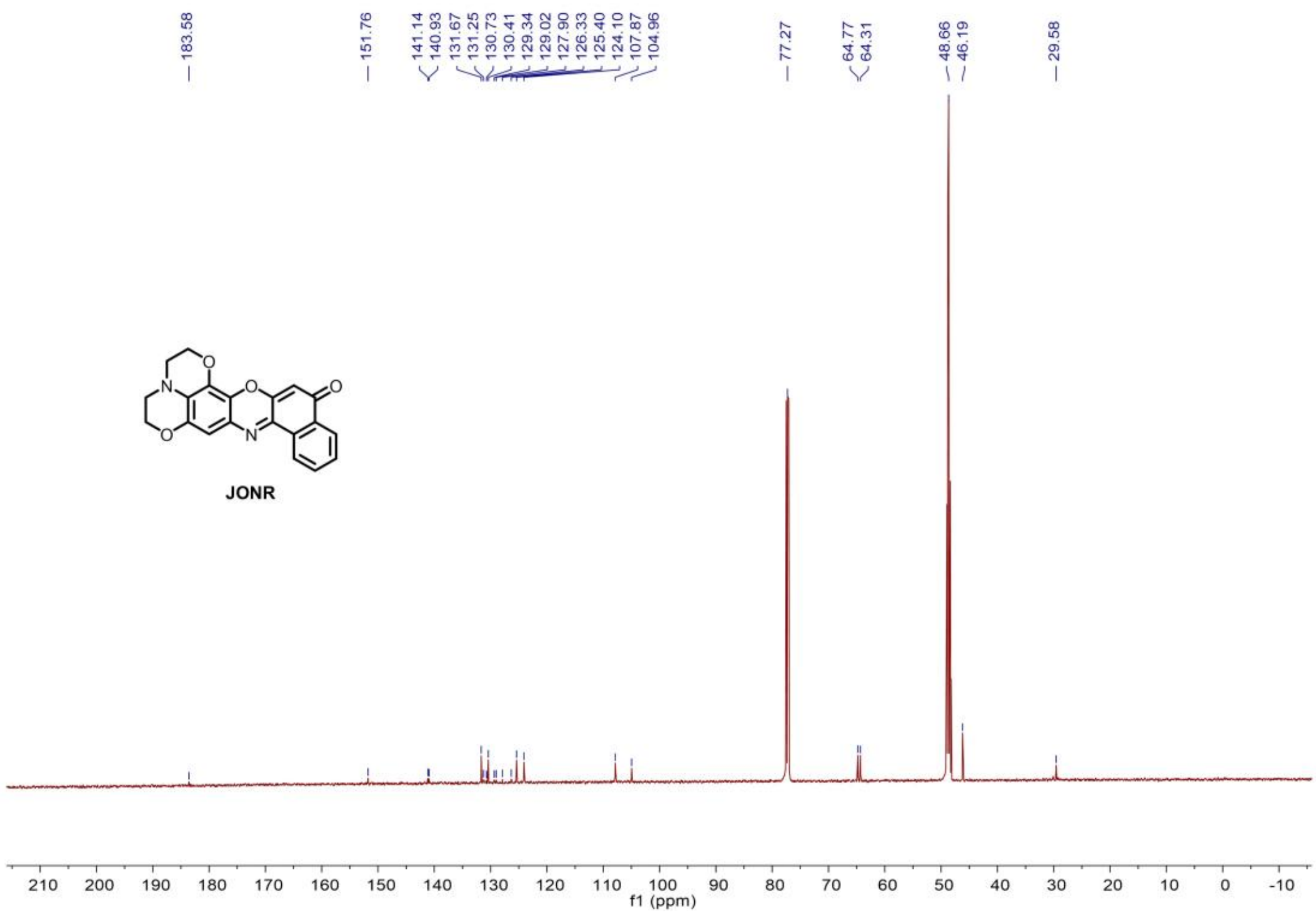
183.58
151.76
141.14
140.93
131.67
131.25
130.73
130.41
129.34
129.02
127.90
126.33
125.40
124.10
107.87
104.96
77.27
64.77
64.31
48.66
46.19
29.58
JONR
210 200 190 180 170 160 150 140 130 120 110 100 90 80 70 60 50 40 30 20 10 0 -10
f1 (ppm)
