# Supplementary material for: Far-red-emitting Nile red with boosted solvatochromism enables multidimensional imaging of membrane heterogeneity and dynamics
Source: Innovation (Camb). 2026 Feb 24;7(8):101319. doi: 10.1016/j.xinn.2026.101319 (PMC13457185; doi:10.1016/j.xinn.2026.101319)
Supplement: Supplementary file 2 [file mmc2.pdf]

OPEN ACCESS

Far-red-emitting Nile red with boosted solvatochromism enables multidimensional imaging of membrane heterogeneity and dynamics

Mingqiao Liu,[1,2,9] Yunzhe Fu,[3,9] Xiaopeng Li,[4] Haolin Zhang,[1] Yumiao Ma,[5] Meiqi Li,[6] Chunyan Shan,[6] Hongxi Zheng,[6] Xiaoyi Lu,[7] Yan Zhang,[6] Li Yu,[4] Peng Xi,[3,*] and Zhixing Chen[1,2,8,*]

*Correspondence: xipeng@pku.edu.cn (P.X.); zhixingchen@pku.edu.cn (Z.C.)

Received: October 24, 2025; Accepted: February 18, 2026; Published Online: February 24, 2026; https://doi.org/10.1016/j.xinn.2026.101319

© 2026 The Authors. Published by Elsevier Inc. on behalf of Youth Innovation Co., Ltd. This is an open access article under the CC BY license (http://creativecommons.org/licenses/by/4.0/).

GRAPHICAL ABSTRACT

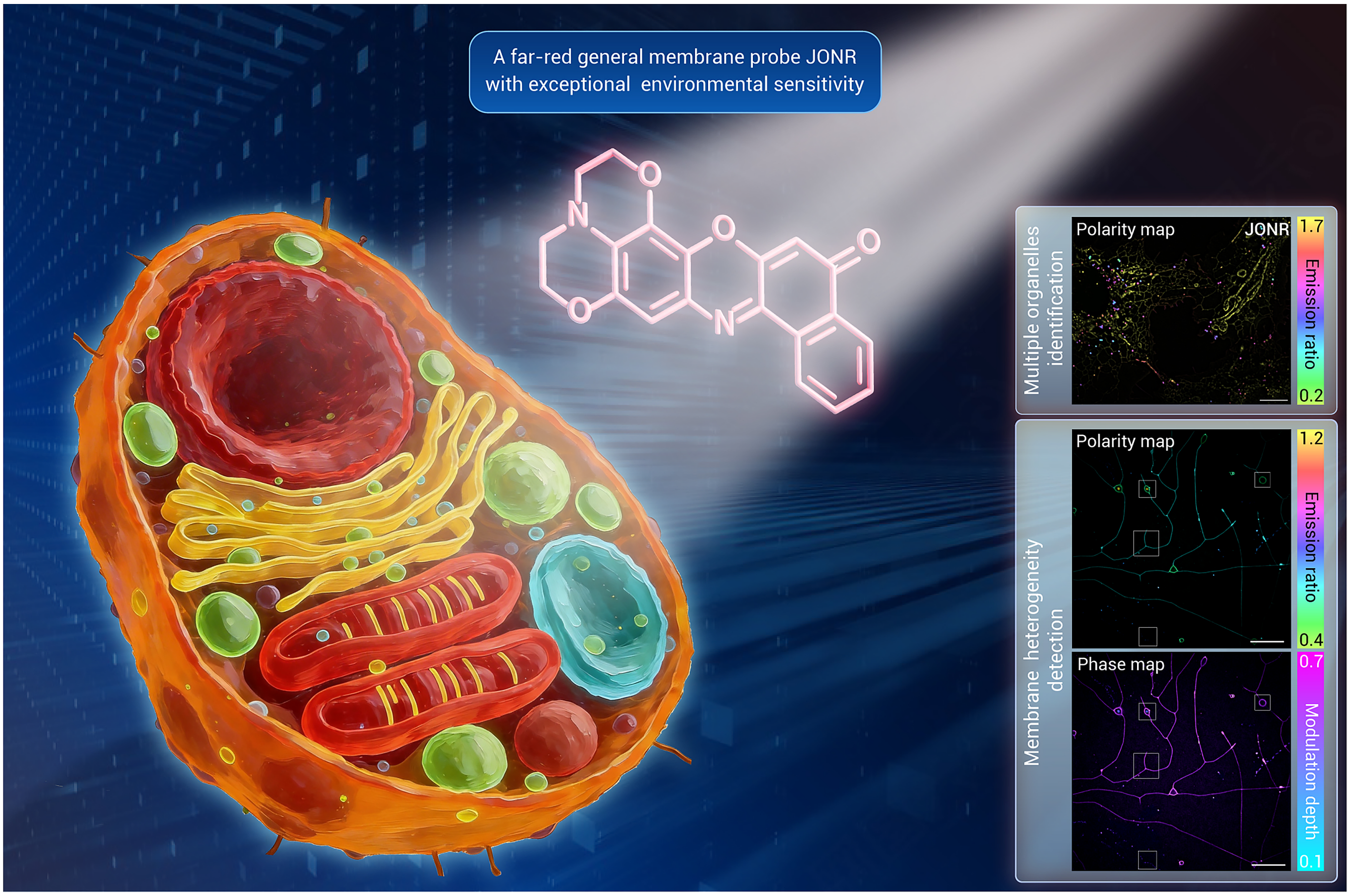

PUBLIC SUMMARY

- JONR has a far-red emission spectrum and superior sensitivity to environmental changes in lipid membranes.
- JONR enables accurate mapping of organelles and monitoring of organelle interaction networks in live cells.
- JONR provides multidimensional optical responses, revealing differences in subcellular membrane heterogeneity.

Far-red-emitting Nile red with boosted solvatochromism enables multidimensional imaging of membrane heterogeneity and dynamics

Mingqiao Liu,[1,2,9] Yunzhe Fu,[3,9] Xiaopeng Li,[4] Haolin Zhang,[1] Yumiao Ma,[5] Meiqi Li,[6] Chunyan Shan,[6] Hongxi Zheng,[6] Xiaoyi Lu,[7] Yan Zhang,[6] Li Yu,[4] Peng Xi,[3,*] and Zhixing Chen[1,2,8,*]

[1]College of Future Technology, Institute of Molecular Medicine, National Biomedical Imaging Center, Beijing Key Laboratory of Cardiometabolic Molecular Medicine, Peking University, Beijing 100871, China

[2]Academy for Advanced Interdisciplinary Studies, Peking University, Beijing 100871, China

[3]Department of Biomedical Engineering, National Biomedical Imaging Center, College of Future Technology, Peking University, Beijing 100871, China

[4]State Key Laboratory of Membrane Biology, Tsinghua University-Peking University Joint Centre for Life Sciences, Beijing Frontier Research Center for Biological Structure, School of Life Sciences, Tsinghua University, Beijing 100084, China

[5]BSJ Institute, Beijing 100084, China

[6]School of Life Sciences, Peking University, Beijing 100871, China

[7]Faculty of Natural, Mathematical & Engineering Sciences, King's College London, London WC2R2ls, UK

[8]Peking-Tsinghua Center for Life Sciences, Academy for Advanced Interdisciplinary Studies Peking University, Beijing 100871, China

[9]These authors contributed equally

*Correspondence: xipeng@pku.edu.cn (P.X.); zhixingchen@pku.edu.cn (Z.C.)

Received: October 24, 2025; Accepted: February 18, 2026; Published Online: February 24, 2026; https://doi.org/10.1016/j.xinn.2026.101319

© 2026 The Authors. Published by Elsevier Inc. on behalf of Youth Innovation Co., Ltd. This is an open access article under the CC BY license (http://creativecommons.org/licenses/by/4.0/).

Citation: Liu M., Fu Y., Li X., et al., (2026). Far-red-emitting Nile red with boosted solvatochromism enables multidimensional imaging of membrane heterogeneity and dynamics. The Innovation **7(8)**, 101319.

The heterogeneity of lipid membranes plays an essential role in regulating the biological functions of organelles. As a solvatochromic probe, Nile red exhibits optical responses to varying membrane environments; however, its limited solvatochromism restricts its ability to detect subtle changes in membrane polarity and lipid order. In this work, we rationally design the auxochrome of Nile red by incorporating an oxygenated julolidine moiety and develop oxygenated julolidine-based Nile red (JONR), which exhibits significantly enhanced sensitivity to environmental polarity, along with strong fluorogenicity from protic to aprotic solvents, large Stokes shifts, and far-red emission in polar environments. Compared to Nile red, JONR demonstrates 3-fold greater accuracy in identifying various organelles in live cells and enables real-time tracking of their dynamic interactions through ratiometric imaging. Furthermore, JONR can distinguish lipid environments through polarization modulation depth and fluorescence lifetime variations. By integrating these properties with its superior spectral response, we present the first comprehensive mapping of subtle lipid heterogeneity within migration-associated structures in L929 cells. The high sensitivity to subtle differences in lipid environments of JONR enables fine resolution of subcellular membrane structures in live cells and provides a new approach for high-throughput analysis of intracellular microenvironments.

INTRODUCTION

Lipids serve as essential structural components of cellular membranes, with mammalian systems harboring thousands of distinct lipid species that collectively establish intricate internal mechanisms of cells.[1,2] The biophysical properties of membranes are primarily determined by their lipid composition. Cholesterol (CL) and sphingomyelin (SM) form a highly packed state, called the liquid-ordered (L_o) phase, while unsaturated lipids mainly form a loosely packed liquid-disordered (L_d) phase.[3–5] Remarkably, the spatial distribution and relative abundance of these phases exhibit pronounced heterogeneity not only across cell types but also at subcellular levels.[6] Therefore, the biological relevance and regulatory mechanisms of membrane phase heterogeneity in health and disease remain central topics in membrane biology.[1] Environment-sensitive probe-based imaging is a powerful approach to visualize fine variations in membrane composition and to quantify lipid packing density, phase order, and membrane tension with subcellular precision.[7–9]

Nile red (NR) is a lipophilic solvatochromic fluorophore whose solvatochromism arises from a strong excited-state dipole moment. In highly polar solvents, the Franck-Condon excited state is stabilized by the dipoles of surrounding solvent molecules, resulting in a redshifted fluorescence emission.[10,11] Compared to other solvatochromic dyes (e.g., Prodan and di-4-ANEPPDHQ), NR exhibits red fluorescence, high quantum yield (QY), and good photostability.[3,12,13] In addition, NR binds preferentially to hydrophobic membrane regions and shows low fluorescence in aqueous environments but strong fluorescence in apolar lipid environments.[13–15] These properties enable NR to function as a "wash-free" probe.[16–18]

Building on the unique advantages of NR for imaging lipid structures, solvatochromic probes have been developed to detect the lipid order of the plasma membrane,[19] achieve super-resolution mapping of membrane organization,[20] and monitor the lipid order in organelles under stress conditions.[21] In addition, various optical modulation and detection strategies have been introduced to improve imaging resolution and throughput. By recording emission spectra at the detection side, differences in NR fluorescence can be analyzed to resolve lipid composition at the single-pixel level.[22] Furthermore, the integration of ratiometric imaging with polarized excitation, termed spectrum and polarization optical tomography (SPOT), enables simultaneous detection of spectral shifts and fluorescence dipole orientations.[23–26] By correlating lipid polarity with phase behavior, SPOT allows identification of lipid heterogeneity across multiple organelles, offering a scalable and high-throughput alternative to conventional specific labeling approaches, which are limited by spectral crosstalk and suffer from decreased labeling efficiency as the number of targets increases (Figure 1A). A recent study also achieved simultaneous imaging and identification of multiple structures by utilizing fluorescent proteins with distinct fluorescence lifetimes in combination with fluorescence lifetime microscopy (FLIM).[27]

These methods have provided valuable tools for membrane biology research, but the analytical precision remains substantially limited. This limitation hampers high-throughput organelle identification and accurate detection of subtle lipid variations at sub-organellar resolution. The fundamental barrier lies in the limited sensitivity of the fluorescent probe to small differences in the microenvironment. The response of NR to environmental polarity remains less sensitive than that of other solvatochromic fluorophores.[8] Current modifications of the NR molecular structure are limited, with most efforts focused on functionalizing its core scaffold.[10,28] Previous studies have introduced electron withdrawing-donating groups into the benzo[a]phenoxazone moiety or extended its conjugated structure via Suzuki coupling.[29–31] However, these modifications have had minimal impact on solvatochromism. Only a few studies have modified the diethylamino group but have had negligible effects on the spectral properties of NR.[32–34]

Here, we design and synthesize a series of NR derivatives through rational auxochrome engineering. Beyond auxochromes, which may induce spectral red-shifts, we introduce electronegative atoms into the conjugated system of NR.[35] Among these derivatives, oxygenated julolidine-based NR (JONR), incorporating 2,3,4,5-tetrahydro-1,6-dioxa-3a-azaphenalene (TDAP) as an auxochrome, offers the following advantages: bathochromically shifted emission spectra extending into the far-red region, a large Stokes shift, strong fluorogenicity from protic solvents to aprotic solvents, and, most importantly, superior sensitivity to environmental polarity (Figure 1). Benefitting from these advantages, simultaneous resolution of lipid membrane heterogeneity across distinct subcellular structures in COS-7 cells was achieved through ratiometric imaging.

www.the-innovation.org

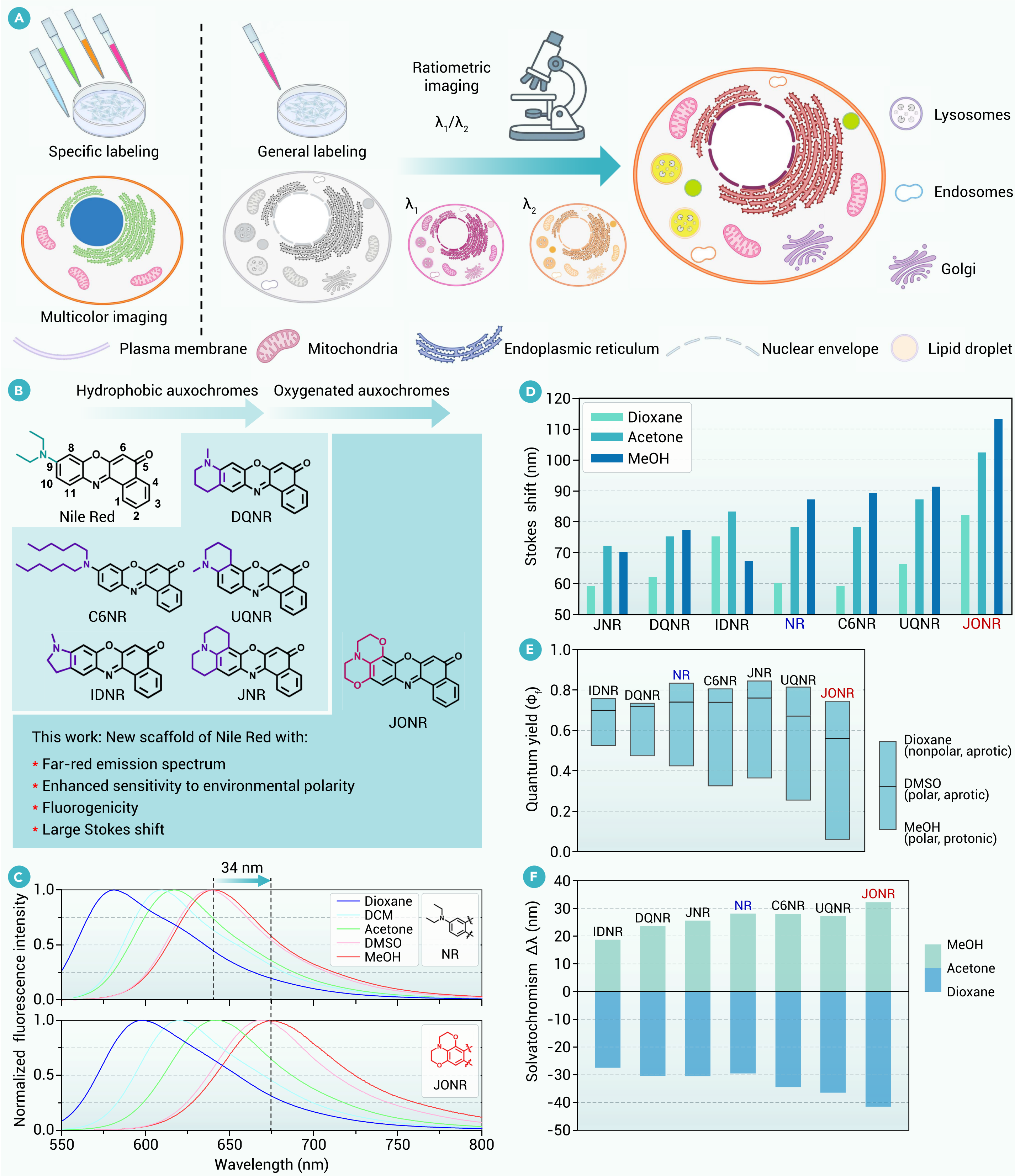

Figure 1. Principles of imaging multiple subcellular structures along with the design and optical properties of new NR derivatives (A) Workflow of the general labeling strategy for imaging and profiling subcellular structures and its advantages over specific labeling. Under the specific labeling strategy (left), conventional multicolor imaging requires distinct markers for each organelle, with limited multiplexing capacity (typically up to four fluorophores in the visible range) and reduced labeling efficiency as the number of targets increases. In contrast, the general labeling strategy (right) employs a single lipophilic probe to stain nearly all lipid-containing structures in live cells. Ratiometric imaging or spectral unmixing is then used to resolve emission shifts among lipid structures at different subcellular locations, enabling analysis of lipid composition or segmentation of subcellular compartments based on their spectral properties. (B) Design strategy and chemical structures of the new NR derivatives. (C) Emission spectra of NR and JONR. (D) Comparison of the Stokes shifts of the NRs. The derivatives are listed in order of their maximum Stokes shift across various solvents. (E) Comparison of the QYs. The derivatives are listed in order of their brightness response to different solvents. (F) Solvatochromism in the emission band of the NRs. The derivatives are listed in order of their solvatochromism. Concentrations of the derivative solutions: 2 μM.

JONR provides 3-fold more accurate identification of morphologically similar organelles than conventional NR probes. Without the need for multicolor labeling, the dynamic extension of endoplasmic reticulum (ER) tubules and "hitchhiking" behavior on rapidly moving late endosomes (LEs) were visualized. Furthermore, JONR exhibits responsiveness to distinct lipid microenvironments through polarization modulation depth and fluorescence lifetime variations. In combination with the superior spectral responses of JONR, we capture subtle compositional and structural heterogeneity within migration-associated structures, including mitochondria, migrasomes, retractosomes, and retraction fibers in L929 cells.

MATERIALS AND METHODS

UV-visible and fluorescence spectroscopy

The absorption and emission spectra of the new NR dyes in spectrum-grade solvents including dioxane, acetone, dichloromethane (DCM), dimethyl sulfoxide (DMSO), methanol (MeOH), methanol-*d4* (CD_3OD), ethanol (EtOH), isopropanol (IPA), n-butanol (n-BuOH), and ethylene glycol (EG) were measured using a Duetta fluorescence and absorbance spectrometer (Horiba, Kyoto, Japan) in a 1 cm square quartz cuvette. All measurements were taken at ambient temperature (23°C ± 2°C). Fluorescent molecules were prepared as stock solutions in DMSO and diluted to keep the DMSO concentration below 1% (v/v).

QY determination

Fluorescence QYs of the new NR dyes in spectrum-grade solvents (dioxane, acetone, DCM, DMSO, MeOH, CD_3OD, EtOH, IPA, n-BuOH, and EG) were determined via the relative determination method. They were determined with rhodamine B (Φ_f = 0.31 in H_2O) as a reference. The absorbance of the sample and reference at their respective excitation wavelengths was controlled to be lower than 0.1. Reported values are averages (n = 4). Fluorescence QYs, Φ_f (sample), were calculated by the following equation:

$$\frac{\Phi_{f,sample}}{\Phi_{f,ref}} = \frac{OD_{ref} I_{sample} d^2_{sample}}{OD_{sample} I_{ref} d^2_{ref}},$$

where Φ_f is the QY of fluorescence; I is the integrated emission intensity; OD is the optical density at the excitation wavelength; d is the refractive index of solvents, d_{water} = 1.33; $d_{dioxane}$ = 1.418; $d_{acetone}$ = 1.424; d_{DCM} = 1.36; d_{DMSO} = 1.48; d_{MeOH} = 1.329; d_{EtOH} = 1.361; d_{IPA} = 1.377; $d_{n\text{-}BuOH}$ = 1.399; and d_{EG} = 1.432

Extinction coefficient

Fluorescent dyes were quantified by quantitative nuclear magnetic resonance (qNMR) using a toluene standard (2.0 μL). A dilution series of 0.5, 1.5, and 2.5 μM of fluorophore was prepared in spectrum-grade solvents for ε (extinction coefficient). Absorbance spectra were recorded on a Duetta fluorescence and absorbance spectrometer (Horiba), and the absorbance maxima were plotted against the concentration. A linear function was fitted to the data using Origin 2023, and extinction coefficients were calculated from the slope following Lambert-Beer's law. Reported values for ε are averages (n = 3).

Bulk bleaching measurement

Dyes were immobilized within polymethylmethacrylate (PMMA) films prepared using an SW-4A spin coater (Setcas, Beijing, China) and round glass coverslips (diameter = 25 mm) as substrates. 100 μL 5% PMMA in chloroform containing 4 μM dye was applied to the coverslip, followed by spin coating (80,000 rpm, 10 s; 800,000 rpm, 45 s). These polymer films were then irradiated using an LSM880 confocal microscope (Carl Zeiss, Oberkochen, Germany) equipped with a 20× Air Plan Apochromat 20×/0.8 NA (numerical aperture) objective. Three groups of time-lapse images (200 frames, 8.2 × 8.2 μm, 1.0 fps (frames per second), 100% laser power) were acquired for each dye, and quantification of fluorescence intensity was achieved using Fiji software from three parallel experiments.

Measurement of singlet oxygen QY

Singlet oxygen QYs were determined using 1,3-diphenylisobenzofuran (DPBF; Q105708, Dibai, Shanghai, China) as a chemical indicator. Dyes and 80 μM DPBF were dissolved in air-saturated MeOH, followed by irradiation with an light emitting diode (LED) lamp (520–530 nm, 50 mW/cm^2). For JONR, NR, and tetramethyl rhodamine ethyl ester (TMRE), the dyes were corrected for concentration so that their absorbance values at 525 nm were equal. DPBF absorption was monitored at 415 nm. The linear decay slope of 415 nm is positively correlated with singlet oxygen QY. TMRE in acetonitrile (ϕ = 0.023) was used as a reference.

GUV preparation and imaging

1,2-Dioleoyl-*sn*-glycero-3-phosphocholine (DOPC), CL, and SM were used in a giant unilamellar vesicle (GUV) preparation. Three kinds of lipids—pure DOPC, DOPC:CL 7:3, and SM:CL 7:3—were dissolved in $CHCl_3$ to a final concentration of 10 mM. GUVs were prepared using the electroformation technique by Vesicle Prep Pro (Nanion Technologies). To immobilize lipid vesicles for confocal imaging, the GUV solution supplemented with 10 mM of NR or JONR was mixed with 0.5 wt % low-gelling-temperature agarose (Sigma) solution at a 1:1 ratio. The mixture was dropped onto a glass slide and was imaged with the SPOT system and FLIM.

Cell culture

COS-7 cells (CRL-1651) were cultured in high-glucose DMEM (Gibco, 21063029) supplemented with 10% fetal bovine serum (FBS; Gibco) and 1% 100 mM sodium pyruvate solution in a 37°C incubator with 5% CO_2. L929 cells were cultured in DMEM (Gibco) supplemented with 10% FBS (Excell), 2 mM GlutaMAX, and 100 U/mL penicillin-streptomycin in 5% CO_2 at 37°C. When the cells reached 80% confluence, they were detached using trypsin-EDTA and subsequently seeded onto 8-chambered cover glasses (Cellvis, C8SB-1.5H) for imaging experiments.

Cell staining process

Stock solutions of the new NR analogs were added to the standard culture medium, with a recommended final concentration of 500 nM. Cells were incubated with the dyes for 20 min, after which they were ready for fluorescence imaging without requiring any additional processing steps. JONR staining is insensitive to the presence or absence of serum in the culture medium. Furthermore, since JONR is non-fluorescent in aqueous solution, no washing steps are necessary post staining, allowing immediate imaging.

RESULTS AND DISCUSSION

Design and synthesis of the new NR family

We aim to improve the solvatochromism of NR by enhancing intramolecular charge transfer in the excited state. The design strategy first modified the diethylamino group of the NR fluorophore into hydrophobic auxochromes to improve electron-donating effects. Furthermore, polar substituents were introduced to extend π conjugation, where multiple polar centers may strengthen dipole-dipole interactions or hydrogen bonds between the dye and solvent molecules, thereby enhancing solvatochromism. We introduced the oxygen atom as both a polar substituent and a conjugated electron-donating group into the NR scaffold. The NR with the diethylamino group replaced by a six-carbon chain is referred to as C6NR. Three conformationally restricted auxochromes were selected: julolidine, tetrahydroquinoline, and indoline. The corresponding NR derivatives are named as follows: JNR (julolidine), DQNR (Nile Red with the quinoline ring in the down position), and UQNR (Nile Red with the quinoline ring in the up position) (1-methyl-1,2,3,4-tetrahydroquinoline, with UQNR having the ring at the 8 position), and IDNR (1-methylindoline). Building upon the JNR scaffold, TDAP was designed as an advanced auxochrome featuring two oxygen atoms integrated into the conjugated system. This optimized derivative was subsequently employed to synthesize the JONR (oxygenated JNR) fluorophore (Figure 1B). The new NR fluorophores were synthesized following a published protocol involving two key reaction steps: a nitroso substitution reaction and a condensation-cyclization sequence, with reaction conditions optimized to achieve a final yield of over 60% for all products.[30]

Impact of new auxochromes on NR optical properties

To systematically investigate the solvatochromism of the new analogues, $E_T(30)$ was selected as the metric for solvent polarity.[11] Dioxane was chosen as the solvent with the lowest polarity, while DCM and acetone were selected as solvents with medium polarity. For high-polarity solvents, we opted for the aprotic solvent DMSO and the protic solvent MeOH. The solvatochromism was defined as the difference between the longest and shortest maximum emission wavelength (λ^{em}_{max}) observed across these solvents.

The spectral data indicate that all modification strategies resulted in a redshift in the emission wavelengths of the new NR derivatives across various solvents, which is consistent with our hypothesis (Table 1). Notably, the emission spectra of JONR in DMSO and MeOH extend into the far-red region (>650 nm), exhibiting a 34 nm redshift in the maximum emission wavelength compared to NR (Figure 1C). The maximum absorption wavelength of JONR is close to that of NR, resulting in a significantly larger Stokes shift among all analogs (Figures 1D and S7).

The effect of auxochromes on the solvatochromism of NR is a key focus. In the case of NR, changing the solvent from acetone to dioxane and methanol

www.the-innovation.org

Table 1. Spectra and photophysical properties of the NR analogs in selected solvents

Dye	Solvent	λ^{abs}_{max} (nm)	λ^{em}_{max} (nm)	Solvatochromism (nm)	Stokes shift (nm)	Φ_f
NR	dioxane	520	581	59	61	0.82
	acetone	532	611		79	0.82
	MeOH	552	640		88	0.42
C6NR	dioxane	520	580	64	60	0.81
	acetone	536	615		79	0.76
	MeOH	554	644		90	0.32
DQNR	dioxane	528	591	55	63	0.74
	acetone	546	622		76	0.71
	MeOH	568	646		78	0.47
UQNR	dioxane	522	589	65	67	0.82
	acetone	538	626		88	0.75
	MeOH	562	654		92	0.25
JNR	dioxane	544	604	57	60	0.85
	acetone	562	635		73	0.84
	MeOH	590	661		71	0.36
IDNR	dioxane	524	600	42	76	0.76
	acetone	544	628		84	0.77
	MeOH	574	642		68	0.52
JONR	dioxane	516	599	75	83	0.75
	acetone	538	641		103	0.69
	MeOH	560	674		114	0.055

causes shifts in the peak emission of −30 and +29 nm, respectively, giving a total solvatochromism of 59 nm. Among these new analogs, C6NR, UQNR, and JONR exhibit enhanced solvatochromism (Figure 1F; Table 1). JONR demonstrates a solvatochromism of 75 nm across different solvents, representing an improvement of over 25% compared to NR. Additionally, we found that the fluorescence of JONR is significantly quenched by protic solvents such as MeOH and water. The QY of JONR in MeOH is 5.5%, while it exhibits almost no fluorescence in water. Compared to its performance in the polar aprotic solvent DMSO, the QY of JONR exhibits nearly a 10-fold increase (Figure 1E). Although it has been previously observed that the QY of NR decreases in polar protic solvents, JONR demonstrates the most significant fluorogenicity. This would help minimize background fluorescence in membrane imaging and enable wash-free labeling.

We also evaluated four aspects of JONR and the reference compounds NR and TMRE: (1) photostability, (2) *in vitro* singlet oxygen generation, (3) phototoxicity based on cell apoptosis, and (4) cytotoxicity. Both JONR and NR demonstrate similar phototoxicity, photostability, and singlet oxygen generation while showing no significant cytotoxicity (Figure S8).

Computational results explain the optical properties exhibited by JONR

The redshifted spectra and improved solvatochromism exhibited by JONR align with our rational design. Through theoretical analysis, we compared the excited states of the structurally similar JONR and JNR to explain these properties. First, the computed S_1 emission wavelength for NR in DMSO was tested using various popular functionals in combination with the TZVP (triple zeta valence plus polarization) basis set, and according to the benchmark result, TPSSh (Tao, Perdew, Staroverov, and Scuseria hybrid functional) was selected for further computation (Table S1).

The computational results at the TPSSh-SMD (solvent model density)/TZVP level show that both JNR and JONR exhibit a significant redshift in polar solvents and that the response for JONR is greater (Table 2). The origin of the redshift is revealed by excited-state hole-electron analysis.[36] In this analysis, the spatial distribution of electron transfer upon excitation is visualized by the hole and electron isosurface. The S_1 states of both JNR and JONR exhibit typical charge transfer excitation, in which the electron from the arylamine motif is transferred to the electron-deficient phenoxazin-5(5H)-one fragment (Figure 2A). The hole-electron mass center distance (D index) was employed to quantify the charge transfer character. JONR exhibits a larger D index (2.758 Å) than JNR (2.203 Å), indicating greater spatial separation between holes and electrons and a stronger charge transfer character in JONR. For JONR, the alkoxyl substitute further contributes to the charge transfer, revealed by the extended hole distribution on the oxygen atoms (Figure 2A), which leads to its redshift over JNR. The improved charge transfer nature is also responsible for the stronger solvent response. In the polar solvent, the charge separation resulting from the introduced oxygen atoms in the S_1 state is further stabilized, leading to the observed redshift and a stronger solvent response (Table 2).

Table 2. Computed S_1 emission wavelength and oscillator strength for JNR and JONR in selected solvents

Solvent	S_1 emission wavelength (nm)	*f* (a.u.)
**JNR**		
DCM	642	1.0852
DMSO	648	1.1600
MeOH	665	1.1736
**JONR**		
DCM	637	0.9773
DMSO	664	1.0400
MeOH	691	0.9793

Two methanol molecules are included to calculate the emission wavelength and oscillator strength data in methanol.

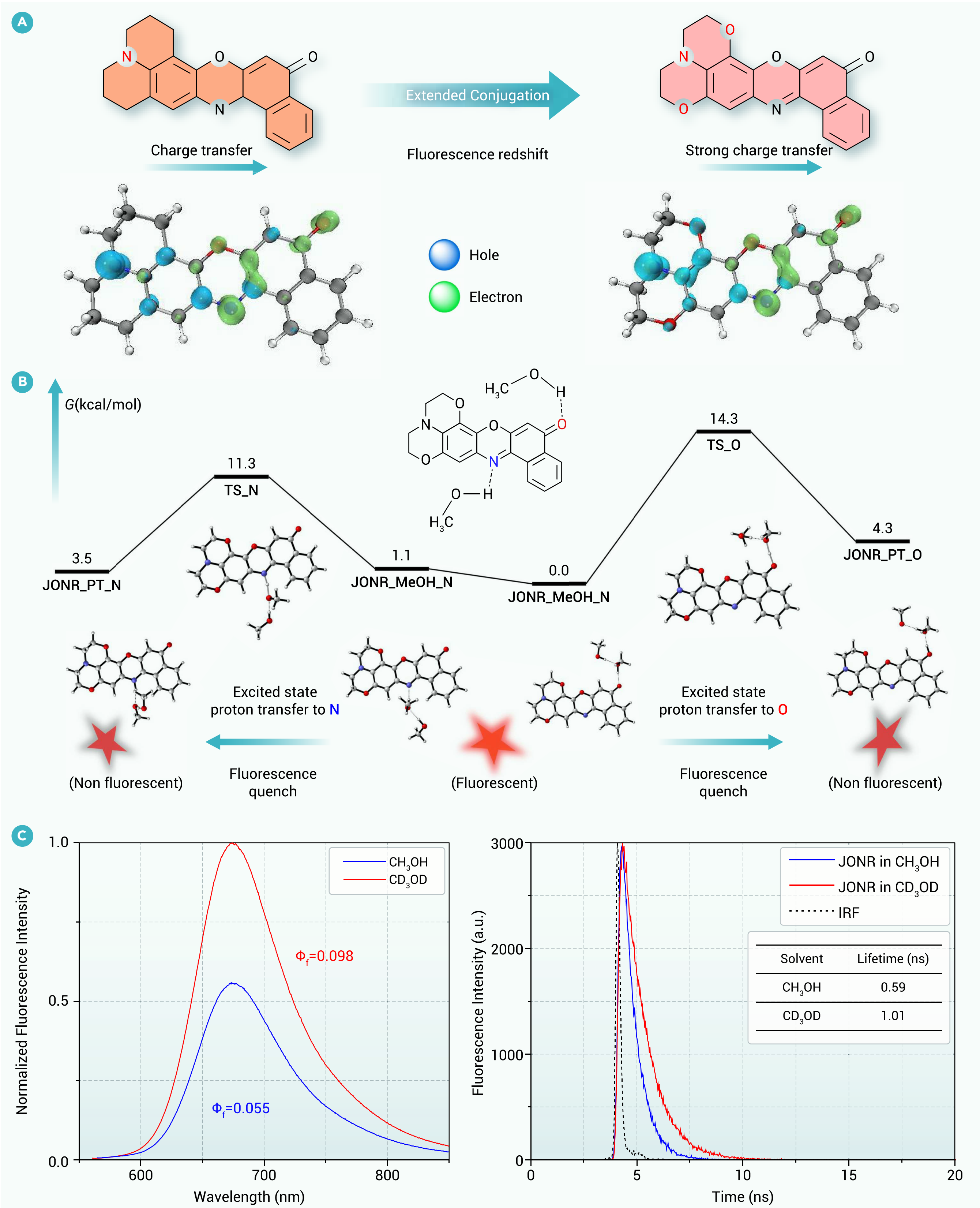

Figure 2. Theoretical analysis and experimental verification of the photophysical properties of JONR (A) S_1 state of the hole (blue) and electron (green) isosurface for the S_1 state of JNR and JONR. The stronger charge transfer accounts for the redshifted spectra of JONR and its improved solvatochromism. The isovalue is taken to be 0.005. (B) Gibbs free energy for the EPT between the S_1 state of JONR and MeOH. In protic solvents, EPT to either nitrogen or oxygen atoms leads to fluorescence quenching of JONR. (C) Normalized fluorescence spectra (left) and fluorescence lifetime decay curves (right) of JONR (2 μM) in MeOH (blue) and CD_3OD (red). The instrument response function (IRF) is presented as a dashed line (black).

www.the-innovation.org

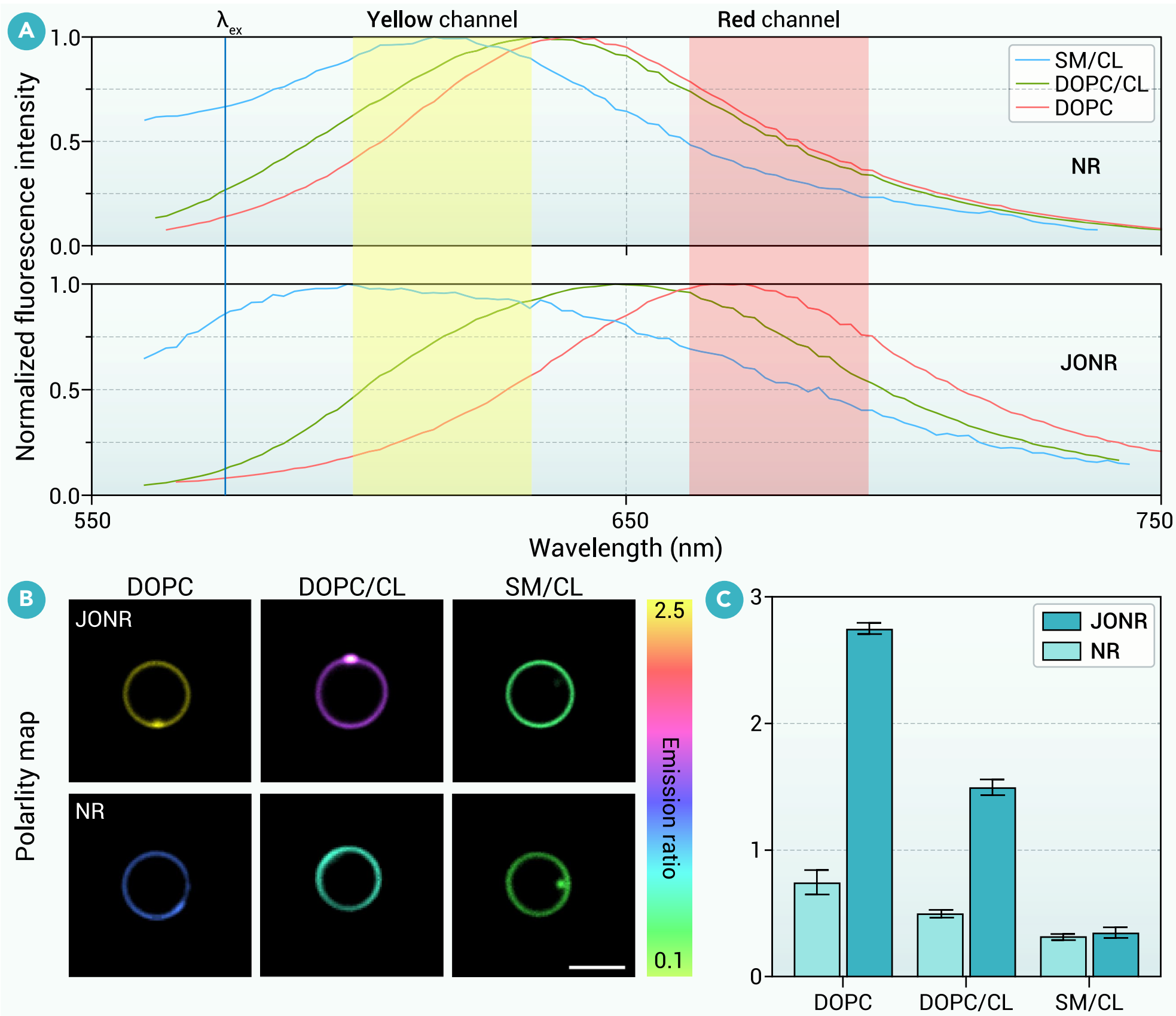

Figure 3. Ratiometric imaging of L_o and L_d phases in GUVs using NR and JONR (A) Normalized fluorescence spectra of NR and JONR from L_d (DOPC) to L_o (SM/CL) phases of GUVs. The blue vertical line represents the excitation wavelength (λ_{ex}), while the yellow and red regions represent the detection range for the yellow and red channels of the microscope. (B) Polarity maps of GUVs with varying lipid compositions stained with JONR or NR. The color bar indicates the maximum and minimum emission ratio values, with different colors representing different levels of membrane polarity. (C) Quantification of emission ratios from GUVs stained with JONR or NR is shown in (B). Each condition was independently repeated at least five times. Data are shown as mean ± SEM. Scale bars: 1 μm.

Additionally, we elucidated the fluorescence quenching phenomenon of JONR in protic solvents through the mechanism of excited-state proton transfer (EPT). The S_1 state oscillator is minimally affected by the solvent polarity (Table 2), which prompted us to investigate the S_1 states in the presence of explicit MeOH molecules. The two possible hydrogen bonding sites—namely the nitrogen and the carbonyl oxygen atoms—were separately investigated. Similarly, the explicit MeOH binding makes a negligible change to the emission wavelength and oscillator strength (JONR_MeOH_N and JONR_MeOH_O). However, the presence of MeOH in this complex enables rapid EPT processes through two transition states, TS_O or TS_N. The resulting protonated JONR-methoxide complex (JONR_PT_O or JONR_PT_N) exhibits a dark state with negligible oscillator strength and no emission (Figure 2B). Although JONR_PT_O and JONR_PT_N possess higher free energy than JONR, the EPT process may open additional decay pathways and therefore be responsible for the quenching in protic solvents.

To verify the existence of the EPT mechanism, we studied the photophysical properties of JONR in various protic solvents. The results showed that JONR exhibits fluorescence quenching in all tested protic solvents, with the quenching efficiency increasing as the solvent pKa decreases and polarity increases (Figures S9A and S9B). This quenching behavior was found to be independent of solvent viscosity (Figures S9C and S9D). Notably, despite the similar polarity of the protic solvent IPA ($E_T(30)$ = 48.4) and the aprotic solvent DMSO ($E_T(30)$ = 45.0), JONR exhibits significant fluorescence quenching in IPA (Figure S9E). Further evidence was obtained by comparing the fluorescence intensity of JONR in MeOHand CD_3OD. Owing to the significantly reduced proton transfer efficiency in CD_3OD compared to MeOH, JONR exhibited a 1.8-fold increase in QY and an approximate doubling of fluorescence lifetime compared to MeOH (Figure 2C). These results provide direct experimental support for the existence of the EPT mechanism.

JONR enables higher-sensitivity detection of membrane heterogeneity at the subcellular level

NR is capable of responding to membrane polarity variations across different organelles; however, its limited environmental sensitivity prevents accurate identification of lipid compositional changes based on spectral shifts. It not only reduces the ability to resolve subcellular heterogeneity but also constrains the throughput of organelle-specific imaging and the capacity to monitor multiple subcellular interactions simultaneously. Conventional multicolor imaging, which relies on filter switching, often fails to meet these demands. To validate the application potential of JONR in membrane imaging, we assessed its environmental sensitivity compared to NR in dual-channel ratiometric imaging. Using 561 nm excitation (blue vertical line in Figure 3A), fluorescence signals from both the yellow and red emission channels (the yellow and red spectral windows in Figure 3A) were collected (Figure S10). The ratio of red to yellow fluorescence intensity was defined as the emission ratio. In polar environments, JONR shows a redshift in its emission spectrum and the emission ratio increases. In non-polar environments, the emission shifts toward blue, and the emission ratio decreases.

We used GUV models to quantitatively investigate the range of emission ratio values of JONR and compare its environmental sensitivity with NR in ratiometric imaging. Three types of GUV models were prepared: pure DOPC, DOPC:CL 7:3, and SM:CL 7:3. Pure DOPC vesicles are generally considered to represent the L_d phase, characterized by relatively high polarity, whereas SM:CL 7:3 vesicles are regarded as representing the L_o phase, which exhibits relatively low polarity. Spectral measurements revealed that JONR exhibited greater solvatochromism than NR in membrane environments, with a more pronounced redshift upon insertion into lipid bilayers (Figure 3A). Polarity maps obtained from ratiometric imaging further visualized the lipid composition heterogeneity among these vesicles (Figure 3B), showing that the environmental sensitivity of JONR was nearly 3-fold higher than that of NR (Figure 3C).

To examine the ability of JONR to label lipid-rich subcellular compartments in live cells, we transfected COS-7 cells with GFP-fused plasmids targeting different organelles. After 24 h of expression, cells were labeled with either NR or JONR. The GFP channel served as a localization marker to identify specific subcellular structures. Co-localization analysis confirmed that JONR successfully labeled at least 14 distinct compartments (Figure S11). Under identical staining and imaging conditions, NR shows limited spectral response to different subcellular membrane structures, whereas JONR exhibits a much stronger ability to distinguish various lipid membranes (Figure 4A). We also evaluated other NR derivatives for live-cell ratiometric imaging (Figures S12A–S12G). Among all tested probes, JONR achieved high signal-to-noise ratios (SNR) in both the yellow and red channels while maintaining moderate brightness, supporting reliable ratiometric quantification (Figures S12H and S12I). Next, to quantitatively evaluate the responsiveness of JONR versus NR to lipid composition differences across subcellular compartments, we analyzed the emission ratio of JONR- and NR-labeled structures identified by GFP labeling. From lipid droplets (LDs) with low polarity to nuclear membranes (NMs) with high polarity, both JONR and NR showed increasing emission ratios, confirming their sensitivity to membrane polarity. However, JONR exhibited a markedly enhanced redshift in the most polar compartments, with an emission ratio

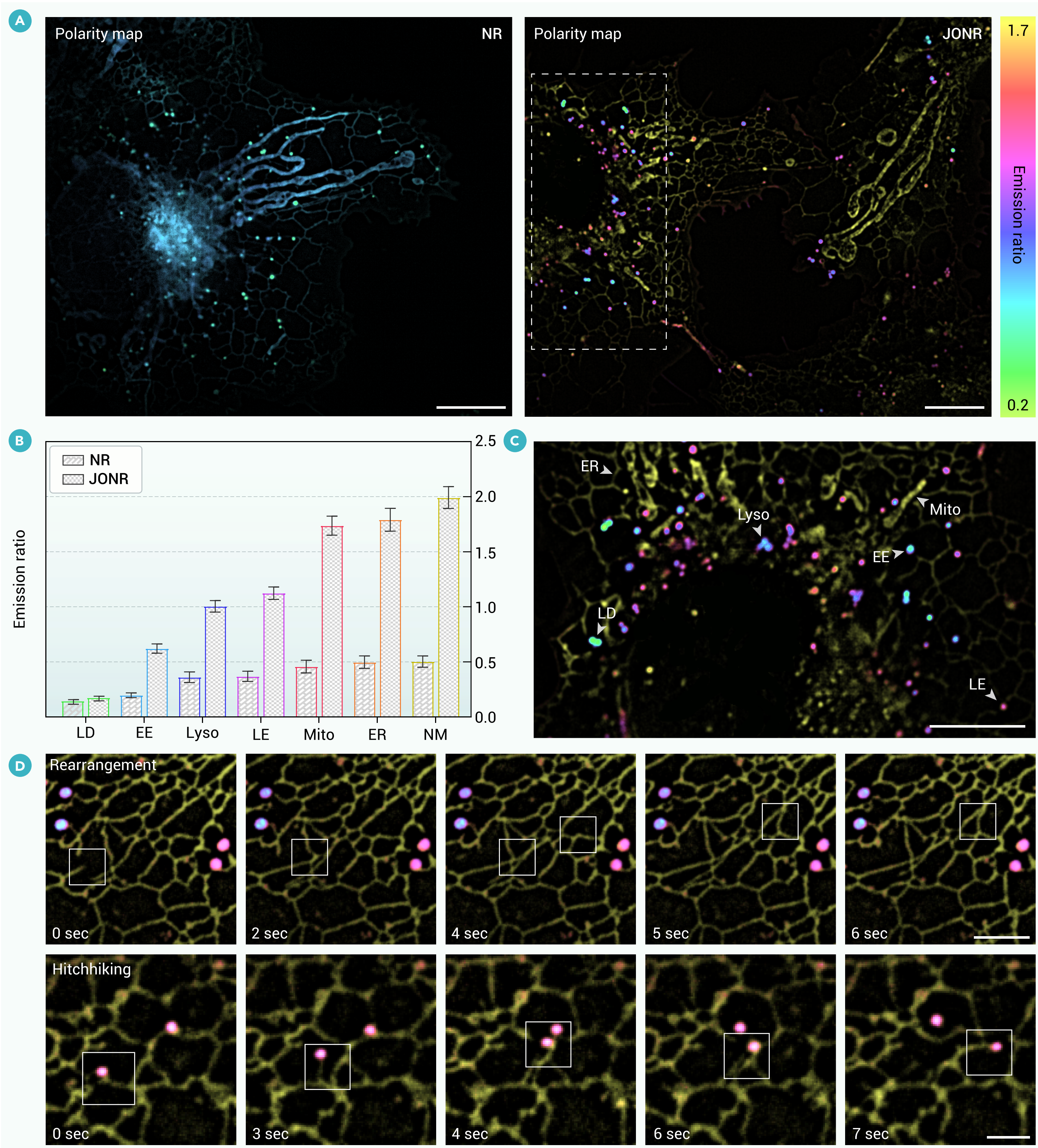

Figure 4. JONR exhibits superior responsiveness to lipid heterogeneity in live-cell ratiometric imaging (A) Polarity map of COS-7 cells showing the spatial distribution of membrane polarity based on NR labeling (left) and JONR labeling (right). (B) Comparison of emission ratios in different subcellular organelles labeled by JONR or NR. Each experiment was independently repeated at least five times. (C) High-magnification view of (B), demonstrating the identification of multiple organelles based on lipid morphology and emission ratio differences. (D) Dynamic interactions between organelles captured in live COS-7 cells. Top: rapid remodeling dynamics of the ER. Bottom: hitchhiking behavior of an LE (pink) along extending ER tubules (yellow). The color scale denotes the emission ratio range. Data are shown as mean ± SEM. Scale bars: 500 nm (D, bottom), 3 μm (D, top), and 10 μm (A and C).

exceeding 3-fold that of NR (Figure 4B). Across the tested subcellular membranes, JONR also exhibited a substantially larger emission ratio dynamic range than other NR derivatives, resulting in improved separation of distinct membrane compartments (Figure S12J). Even between adjacent compartments with comparable membrane polarity, such as lysosomes (Lyso) and LEs, JONR produced greater and more discernible ratio variations than NR. This highlights the superior sensitivity of JONR to subtle differences in membrane polarity, enabling fine identification between morphologically similar organelles.

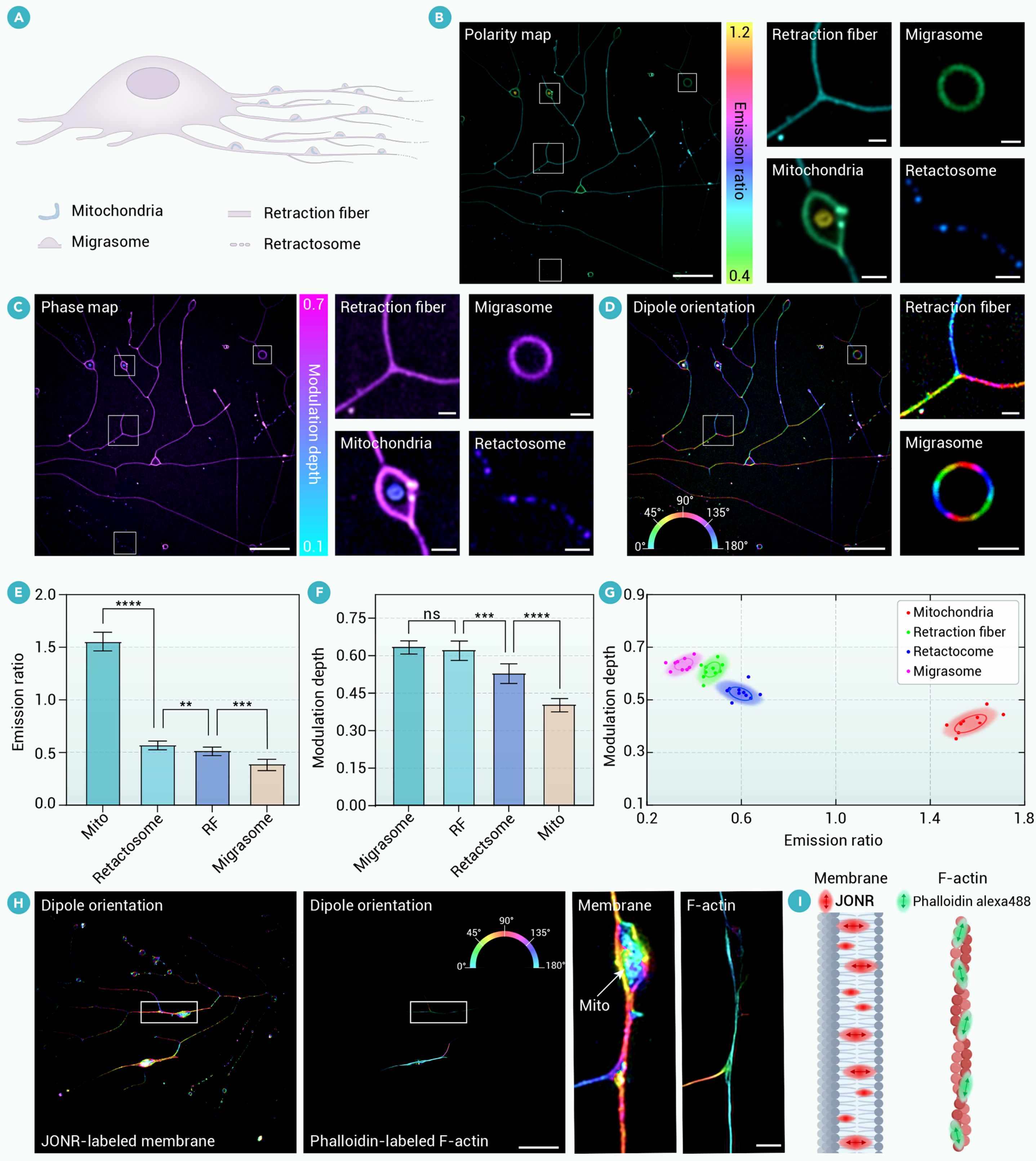

Figure 5. JONR reveals lipid membrane heterogeneity of migration-associated subcellular structures (A) Schematic of subcellular structures closely associated with cell migration, including retraction fibers, migrasomes, mitochondria, and retractosomes. (B) Polarity map of migration-associated structures (left) and magnified view (right). The color scale denotes the emission ratio range, indicating lipid polarity variations across retraction fibers, migrasomes, mitochondria, and retractosomes. (C) Phase map of migration-associated structures (left) and magnified view (right). The color scale reflects the modulation depth range, highlighting local variations in lipid packing. (D) Dipole orientation map of migration-associated structures (left) and magnified view (right). Hue represents the orientation of the fluorescence dipole. JONR-stained retraction fibers and migrasomes exhibit highly ordered dipole alignment. (E) Quantification of emission ratios from migration-associated structures shown in (B). (F) Quantification of modulation depths from structures shown in (C). (G) Optical fingerprint plot classifying migration-associated subcellular structures based on emission ratio and modulation depth. Ellipses denote standard deviation

(legend continued on next page)

By combining emission ratio mapping and morphological features, we were able to simultaneously identify multiple organelles within live cells, including the ER, mitochondria, lysosomes, early endosomes (EEs), LEs, and LDs (Figure 4C).

Notably, JONR also enabled the capture of dynamic organelle interactions that previously required dual- or triple-labeling strategies. We focused on the ER, the largest intracellular organelle, which undergoes rapid remodeling and interacts extensively with other organelles. In 2018, Guo et al. uncovered new ER remodeling mechanisms, including hitchhiking of the ER on motile organelles and budding.[37] Using SPOT imaging, we can not only visualize the dynamic extension events of ER tubules in real time but also observe the hitchhiking behavior of ER tubules attached to fast-moving LEs (Figure 4D), highlighting the significant advantages of general labeling and ratiometric imaging in studying organelle interactomes.

Multidimensional imaging of membrane orientation and lipid order with JONR

To further expand the imaging dimension and reveal in-depth structural insights into subcellular membrane structures, we characterized the polarization properties of JONR on GUVs using SPOT imaging. Polarization modulation was applied at the excitation side, and the fluorescence intensity under varying excitation polarization angles was recorded (Figure S10). Fluorophores embedded in ordered membranes exhibited stronger modulation depth and more precisely resolved dipole orientations, suggesting restricted dipole mobility. In contrast, disordered membranes displayed reduced modulation depth (Figure S13A) and weaker polarization responses, with dipole orientations being resolved with lower precision (Figure S13B), consistent with increased dipole flexibility.[23]

We next performed FLIM imaging to collect fluorescence lifetimes of GUVs with distinct lipid compositions. Dipoles in ordered membranes exhibited prolonged relaxation times, indicating restricted rotational freedom, whereas disordered membranes displayed shorter lifetimes, reflecting increased rotational mobility (Figure S13C).[38] The SPOT and FLIM measurements exhibited consistent trends (Figures S13D and S13E), indicating that JONR effectively reports membrane heterogeneity through both modulation depth and fluorescence lifetime. Together with emission ratio analysis, measurements of modulation depth and fluorescence lifetime enabled a systematic evaluation of how JONR responds to membrane heterogeneity through three distinct optical properties (Figure S13F).

Polarization modulation can further be applied to delineate the relative spatial relationships between membranes and other cellular structures, such as the cytoskeleton. The periodic membrane-associated skeleton on axons serves as a structural framework to organize cytoskeletal and membrane components with nanoscale regularity.[36,37] We first labeled filamentous actin (F-actin) in cultured neurons with phalloidin-Alexa 488 (Figure S13G). In previous studies, the emission dipole of phalloidin-Alexa 488 was shown to align parallel to the F-actin axis.[39] The resulting orientation map showed that the dipole orientations were consistent with the longitudinal extension of axons, suggesting that actin filaments within the membrane-associated skeleton are arranged side by side rather than end to end, in agreement with recent findings using polarization SIM (pSIM).[39] We further performed SPOT imaging on JONR-labeled neuronal axonal membranes (Figure S13G) and the growth cone structures at the distal end of axons (Figure S13H). The orientation of membrane-bound dipoles was found to be perpendicular to the direction of axonal extension and orthogonal to the protrusion axis of filopodia emerging from the growth cone.[40,41] These observations indicate that JONR dipoles align parallel to the phospholipid bilayer within the membrane, providing references for investigating the spatial relationships between membranes and other cellular structures. In parallel, FLIM revealed that the fluorescence lifetime of JONR on the axonal membrane is significantly longer than within intracellular mitochondria, suggesting a more ordered and tightly packed membrane environment at the axonal surface (Figure S14).

Lipid properties of migration-associated subcellular structures

Migrasomes are a newly identified class of vesicular structures that mediate the release of cellular contents and the transfer of signaling molecules, playing essential roles in physiological processes such as tissue development and immune responses.[42,43] During cell migration along the extracellular matrix, retraction fibers are pulled out from the trailing edge of the plasma membrane, upon which swollen structures—migrasomes—form. As migration progresses, some retraction fibers break and generate subcellular structures known as retractosomes, while mature migrasomes detach from the cell surface and are released into the extracellular environment (Figure 5A), facilitating intercellular communication.[44] To investigate the lipid heterogeneity of subcellular structures during migration, we labeled L929 cells with JONR and employed SPOT imaging to quantify the emission ratio and modulation depth of migrasomes, retraction fibers, and retractosomes. Analysis revealed that the emission ratio progressively increased from migrasomes to retraction fibers and then to retractosomes, suggesting a gradual rise in membrane polarity (Figures 5B and 5E). However, the low sensitivity of NR to subtle variations in lipid composition limits its ability to detect these fine differences in membrane structural organization (Figure S15). Recent studies have reported that SM is crucial for migrasome formation, while CL accumulates during their maturation to stabilize membrane structures.[45–47] Despite the association of these lipids with enhanced membrane order, we observed no significant difference in modulation depth between migrasomes and retraction fibers (Figures 5C and 5F). Both structures exhibited similarly high modulation depths, with dipole orientations aligned parallel to the direction of membrane extension (Figure 5D), indicating a high degree of lipid order. The decoupling between emission ratio and modulation depth reveals structural and compositional differences among them, and the superior environmental sensitivity of JONR enables effective capture of these variations. Recent cryoelectron tomography studies further revealed that Tspan7 assembles into right-handed helical structures along retraction fibers, forming a transmembrane scaffold that may introduce lipid order independent of lipid composition, thereby influencing membrane physical properties.[48] We also detected mitochondria within migrasomes, consistent with the recently proposed process of "mitocytosis," a migration-dependent mitochondrial quality control mechanism that links mitochondrial homeostasis with cellular motility. Quantitative analysis showed that mitochondria exhibited the highest emission ratios but the lowest modulation depths, reflecting membranes of higher polarity and lower lipid order. Based on these multidimensional measurements, we constructed lipid fingerprint profiles for different subcellular structures (Figure 5G), providing a clear visualization of their membrane property differences.

Moreover, to investigate whether the cytoskeleton is spatially associated with migration-related membrane structures, we labeled F-actin with phalloidin-Alexa 488 and performed dipole orientation mapping. We observed the presence of actin filaments adjacent to migrasomes containing mitochondria (Figure 5H). Notably, the orientation rings derived from JONR and F-actin labeling differed by approximately 90° (Figure 5I), indicating that the fluorescence dipoles of the two labels are orthogonal. These findings highlight the capacity of JONR to resolve lipid properties across migration-associated subcellular structures with high spatial and orientational precision. By linking membrane polarity, order, and dipole orientation, this approach offers a powerful framework to dissect the biophysical underpinnings of membrane remodeling during cellular migration.

CONCLUSION

Advances in imaging technology and membrane biology have spurred the need to acquire complex biophysical information of membranes, extending beyond spatiotemporal resolution. To achieve precise organelle identification for interaction studies or to resolve membrane heterogeneity at the super-resolution level, the design of fluorescent probes with superior environmental sensitivity is crucial. In this work, we developed JONR, a novel far-red-emitting NR derivative incorporating a rigid TDAP

(σ) and 2σ confidence boundaries. (H) Dipole orientation maps of migration-associated membrane structures stained with JONR and actin filaments stained with phalloidin-Alexa 488 (left). An enlarged view shows mitochondria located within migrasomes during mitocytosis (right). (I) Schematic of dipole orientations of JONR and phalloidin-Alexa 488 in the membrane and actin filaments, respectively. Data are shown as mean ± SEM. **$p < 0.01$, ***$p < 0.001$, ****$p < 0.0001$ (one-way ANOVA). Scale bars: 1 μm (B, right; C, right; D, right; H, right), 5 μm (B, left; C, left; D, left; H, left).

auxochrome, which exhibits significantly enhanced solvatochromism, a large Stokes shift, and improved fluorogenicity while maintaining high QYs in hydrophobic environments. JONR demonstrates pronounced responsiveness to membrane microenvironmental changes through multiple photophysical parameters, including emission spectra, polarization characteristics, and fluorescence lifetime. By implementing SPOT technology, we showcase its exceptional capability to identify subcellular organelles and monitor their dynamic interactions. JONR enables simultaneous mapping of membrane polarity and lipid order, revealing structural heterogeneity across distinct membrane domains of migrasomes. This breakthrough will provide novel mechanistic insights into organelle formation and maturation processes. This work establishes a powerful template for synergistic integration of a novel imaging system with the smart molecular probe in cell biology research. We believe that JONR provides an elegant solution for multidimensional investigation of subcellular membrane properties and enables analysis of the real-time organelle interactome.

RESOURCE AVAILABILITY

Materials availability

All stable reagents generated in this study are available from the lead contact.

Data and code availability

Data presented in this study are provided in supplementary tables. Data are available from the corresponding authors upon request.

FUNDING AND ACKNOWLEDGMENTS

This project was supported by funds from the Major Basic Research Project of the Natural Science Foundation of Shandong Province (ZR2024ZD27 to P.X.), the National Key R&D Program of China (2021YFF0502904 to Z.C. and 2022YFC3401100 to P.X.), the Beijing Municipal Science & Technology Commission (project Z221100003422013 to Z.C.), the Beijing National Laboratory for Molecular Sciences (BNLMS202407 to Z.C.), the National Natural Science Foundation of China (W2412031 to Z.C.). We thank the Metabolic Mass Spectrometry Platform at the IMM, the analytical instrumentation center of Peking University, the NMR facility of the National Center for Protein Sciences at Peking University, and the National Center for Protein Sciences at Peking University. The funders had no role in study design, data collection and analysis, decision to publish, or preparation of the manuscript.

AUTHOR CONTRIBUTIONS

M. Liu, Y. Fu, M. Li, Z. Chen, and P. Xi conceived the idea. M. Liu conducted molecular synthesis, GUV preparation, and spectroscopic measurements. Y. Fu conducted the setup of microscopy and *in vitro* imaging and image analysis. X. Li conducted L929 cell cultivation and provided suggestions. H. Zhang and X. Lu supported molecular synthesis. Y. Ma conducted the computational data. H. Zheng and Y. Zhang conducted neuron cell isolation and cultivation. Z. Chen and P. Xi supervised the project. M. Liu, Y. Fu, Z. Chen, and P. Xi wrote the manuscript with inputs from all authors.

DECLARATION OF INTERESTS

The authors declare no competing interests.

SUPPLEMENTAL INFORMATION

It can be found online at https://doi.org/10.1016/j.xinn.2026.101319.

REFERENCES

1. Harayama, T. and Riezman, H. (2018). Understanding the diversity of membrane lipid composition. *Nat. Rev. Mol. Cell Biol.* **19**:281–296. DOI:10.1038/nrm.2017.138
2. Morgan, P.K., Pernes, G., Huynh, K. et al. (2024). A lipid atlas of human and mouse immune cells provides insights into ferroptosis susceptibility. *Nat. Cell Biol.* **26**:645–659. DOI:10.1038/s41556-024-01377-z
3. Dietrich, C., Bagatolli, L.A., Volovyk, Z.N. et al. (2001). Lipid rafts reconstituted in model membranes. *Biophys. J.* **80**:1417–1428. DOI:10.1016/s0006-3495(01)76114-0
4. Veatch, S.L. and Keller, S.L. (2002). Organization in Lipid Membranes Containing Cholesterol. *Phys. Rev. Lett.* **89**:268101. DOI:10.1103/PhysRevLett.89.268101
5. Baumgart, T., Hess, S.T. and Webb, W.W. (2003). Imaging coexisting fluid domains in biomembrane models coupling curvature and line tension. *Nature* **425**:821–824. DOI:10.1038/nature02013
6. Basu Ball, W., Neff, J.K. and Gohil, V.M. (2018). The role of nonbilayer phospholipids in mitochondrial structure and function. *FEBS Lett.* **592**:1273–1290. DOI:10.1002/1873-3468.12887
7. Klymchenko, A.S. and Kreder, R. (2014). Fluorescent probes for lipid rafts: from model membranes to living cells. *Chem. Biol.* **21**:97–113. DOI:10.1016/j.chembiol.2013.11.009
8. Klymchenko, A.S. (2017). Solvatochromic and Fluorogenic Dyes as Environment-Sensitive Probes: Design and Biological Applications. *Acc. Chem. Res.* **50**:366–375. DOI:10.1021/acs.accounts.6b00517
9. Klymchenko, A.S. (2023). Fluorescent Probes for Lipid Membranes: From the Cell Surface to Organelles. *Acc. Chem. Res.* **56**:1–12. DOI:10.1021/acs.accounts.2c00586
10. Martinez, V. and Henary, M. (2016). Nile red and nile blue: applications and syntheses of structural analogues. *Chem. Eur J.* **22**:13764–13782. DOI:10.1002/chem.201601570
11. Reichardt, C. (1994). Solvatochromic dyes as solvent polarity indicators. *Chem. Rev.* **94**:2319–2358. DOI:10.1021/cr00032a005
12. Jin, L., Millard, A.C., Wuskell, J.P. et al. (2006). Characterization and application of a new optical probe for membrane lipid domains. *Biophys. J.* **90**:2563–2575. DOI:10.1529/biophysj.105.072884
13. Greenspan, P. and Fowler, S.D. (1985). Spectrofluorometric studies of the lipid probe, nile red. *J. Lipid Res.* **26**:781–789. DOI:10.1016/S0022-2275(20)34307-8
14. Greenspan, P., Mayer, E.P. and Fowler, S.D. (1985). Nile red: a selective fluorescent stain for intracellular lipid droplets. *J. Cell Biol.* **100**:965–973. DOI:10.1083/jcb.100.3.965
15. Mishra, R., Sjölander, D. and Hammarström, P. (2011). Spectroscopic characterization of diverse amyloid fibrils in vitro by the fluorescent dye Nile red. *Mol. Biosyst.* **7**:1232–1240. DOI:10.1039/C0MB00236D
16. Prifti, E., Reymond, L., Umebayashi, M. et al. (2014). A fluorogenic probe for SNAP-tagged plasma membrane proteins based on the solvatochromic molecule Nile red. *ACS Chem. Biol.* **9**:606–612. DOI:10.1021/cb400819c
17. Sundukova, M., Prifti, E., Bucci, A. et al. (2019). A chemogenetic approach for the optical monitoring of voltage in neurons. *Angew. Chem. Int. Ed.* **58**:2341–2344. DOI:10.1002/anie.201812967
18. Hanser, F., Marsol, C., Valencia, C. et al. (2021). Nile red-based GPCR ligands as ultrasensitive probes of the local lipid microenvironment of the receptor. *ACS Chem. Biol.* **16**:651–660. DOI:10.1021/cb400819c
19. Kucherak, O.A., Oncul, S., Darwich, Z. et al. (2010). Switchable nile red-based probe for cholesterol and lipid order at the outer leaflet of biomembranes. *J. Am. Chem. Soc.* **132**:4907–4916. DOI:10.1021/ja100351w
20. Danylchuk, D.I., Moon, S., Xu, K. et al. (2019). Switchable solvatochromic probes for live-cell super-resolution imaging of plasma membrane organization. *Angew. Chem. Int. Ed.* **58**:14920–14924. DOI:10.1002/anie.201907690
21. Danylchuk, D.I., Jouard, P.-H. and Klymchenko, A.S. (2021). Targeted solvatochromic fluorescent probes for imaging lipid order in organelles under oxidative and mechanical stress. *J. Am. Chem. Soc.* **143**:912–924. DOI:10.1021/jacs.0c10972
22. Moon, S., Yan, R., Kenny, S.J. et al. (2017). Spectrally Resolved, Functional Super-Resolution Microscopy Reveals Nanoscale Compositional Heterogeneity in Live-Cell Membranes. *J. Am. Chem. Soc.* **139**:10944–10947. DOI:10.1021/jacs.7b03846
23. Zhanghao, K., Liu, W., Li, M. et al. (2020). High-dimensional super-resolution imaging reveals heterogeneity and dynamics of subcellular lipid membranes. *Nat. Commun.* **11**:5890. DOI:10.1038/s41467-020-19747-0
24. Zhanghao, K., Li, M., Chen, X. et al. (2025). Fast segmentation and multiplexing imaging of organelles in live cells. *Nat. Commun.* **16**:2769. DOI:10.1038/s41467-025-57877-5
25. Fu, Y., Hou, Y., Liang, Q. et al. (2025). Triangle-beam interference structured illumination microscopy. *Nat. Photonics* **19**:1122–1131. DOI:10.1038/s41566-025-01730-0
26. Zhong, S., Qiao, L., Ge, X. et al. (2024). Three-dimensional dipole orientation mapping with high temporal-spatial resolution using polarization modulation. *PhotoniX* **5**:12. DOI:10.1186/s43074-024-00127-6
27. Tan, Z., Hsiung, C.-H., Feng, J. et al. (2025). Time-resolved fluorescent proteins expand fluorescent microscopy in temporal and spectral domains. *Cell* **188**:6987–7005.e28. DOI:10.1016/j.cell.2025.08.035
28. Jose, J. and Burgess, K. (2006). Benzophenoxazine-based fluorescent dyes for labeling biomolecules. *Tetrahedron* **62**:11021–11037. DOI:10.1016/j.tet.2006.08.056
29. Lauritsen, L., Szomek, M., Hornum, M. et al. (2024). Ratiometric fluorescence nanoscopy and lifetime imaging of novel Nile red analogs for analysis of membrane packing in living cells. *Sci. Rep.* **14**:13748. DOI:10.1038/s41598-024-64180-8
30. Hornum, M., Mulberg, M.W., Szomek, M. et al. (2021). Substituted 9-diethylaminobenzo [a] phenoxazin-5-ones (Nile red Analogues): Synthesis and photophysical properties. *J. Org. Chem.* **86**:1471–1488. DOI:10.1021/acs.joc.0c02346
31. Prioli, S., Reinholdt, P., Hornum, M. et al. (2019). Rational design of Nile red analogs for sensing in membranes. *J. Phys. Chem. B* **123**:10424–10432. DOI:10.1021/acs.jpcb.9b09691
32. Long, J., Wang, Y.M., Matsuura, T. et al. (1999). Synthesis and fluorescence properties of novel benzo [a] phenoxazin-5-one derivatives. *J. Heterocycl. Chem.* **36**:895–899. DOI:10.1002/jhet.5570360411
33. Jose, J. and Burgess, K. (2006). Syntheses and properties of water-soluble Nile red derivatives. *J. Org. Chem.* **71**:7835–7839. DOI:10.1021/jo061369v
34. Sherman, D.B., Pitner, J.B., Ambroise, A. et al. (2006). Synthesis of thiol-reactive, long-wavelength fluorescent phenoxazine derivatives for biosensor applications. *Bioconjug. Chem.* **17**:387–392. DOI:10.1021/bc050309d

35. Dong, Y., Zou, Y., Jia, X. et al. (2023). An acidic medium-compatible deep-near-infrared dye for in vivo imaging. *Smart Mol.* **1**:e20230001. DOI:10.1002/smo.20230001
36. Liu, Z., Lu, T. and Chen, Q. (2020). An sp-hybridized all-carboatomic ring, cyclo [18] carbon: Electronic structure, electronic spectrum, and optical nonlinearity. *Carbon* **165**:461–467. DOI:10.1016/j.carbon.2020.05.023
37. Guo, Y., Li, D., Zhang, S. et al. (2018). Visualizing Intracellular Organelle and Cytoskeletal Interactions at Nanoscale Resolution on Millisecond Timescales. *Cell* **175**:1430–1442.e17. DOI:10.1016/j.cell.2018.09.057
38. Sot, J., Gartzia-Rivero, L., Bañuelos, J. et al. (2022). Liquid-crystalline, liquid-ordered, rippled and gel lipid bilayer phases as observed with nile red fluorescence. *J. Mol. Liq.* **363**:119874. DOI:10.1016/j.molliq.2022.119874
39. Zhanghao, K., Chen, X., Liu, W. et al. (2019). Super-resolution imaging of fluorescent dipoles via polarized structured illumination microscopy. *Nat. Commun.* **10**:4694. DOI:10.1038/s41467-019-12681-w
40. Igarashi, M. (2019). Molecular basis of the functions of the mammalian neuronal growth cone revealed using new methods. *Proc. Jpn. Acad. Ser. B Phys. Biol. Sci.* **95**:358–377. DOI:10.2183/pjab.95.026
41. Lowery, L.A. and Van Vactor, D. (2009). The trip of the tip: understanding the growth cone machinery. *Nat. Rev. Mol. Cell Biol.* **10**:332–343. DOI:10.1038/nrm2679
42. Ma, L., Li, Y., Peng, J. et al. (2015). Discovery of the migrasome, an organelle mediating release of cytoplasmic contents during cell migration. *Cell Res.* **25**:24–38. DOI:10.1038/cr.2014.135
43. Jiao, H., Jiang, D., Hu, X. et al. (2021). Mitocytosis, a migrasome-mediated mitochondrial quality-control process. *Cell* **184**:2896–2910.e13. DOI:10.1016/j.cell.2021.04.027
44. Wang, Y., Gao, S., Liu, Y. et al. (2022). Retractosomes: small extracellular vesicles generated from broken-off retraction fibers. *Cell Res.* **32**:953–956. DOI:10.1038/s41422-022-00666-2
45. Liang, H., Ma, X., Zhang, Y. et al. (2023). The formation of migrasomes is initiated by the assembly of sphingomyelin synthase 2 foci at the leading edge of migrating cells. *Nat. Cell Biol.* **25**:1173–1184. DOI:10.1038/s41556-023-01188-8
46. Huang, Y., Zucker, B., Zhang, S. et al. (2019). Migrasome formation is mediated by assembly of micron-scale tetraspanin macrodomains. *Nat. Cell Biol.* **21**:991–1002. DOI:10.1038/s41556-019-0367-5
47. Dharan, R., Huang, Y., Cheppali, S.K. et al. (2023). Tetraspanin 4 stabilizes membrane swellings and facilitates their maturation into migrasomes. *Nat. Commun.* **14**:1037. DOI:10.1038/s41467-023-36596-9
48. Wang, D., Jia, X., Zhang, Q. et al. (2024). Polymerization of Tspan7 into Helical Transmembrane Skeletons for Membrane Tether Stabilization. Preprint at *bioRxiv*. DOI:10.1101/2024.06.27.600806

The Innovation, Volume *7*

Supplemental Information

Far-red-emitting Nile red with boosted solvatochromism enables multi-dimensional imaging of membrane heterogeneity and dynamics

Mingqiao Liu, Yunzhe Fu, Xiaopeng Li, Haolin Zhang, Yumiao Ma, Meiqi Li, Chunyan Shan, Hongxi Zheng, Xiaoyi Lu, Yan Zhang, Li Yu, Peng Xi, and Zhixing Chen

Supporting Information

Table of Contents

Supplementary Figures……………………………………………………………….3

Experimental Details…………………………………………………………….15

Microscopy Setup and Imaging Protocol……………………………………….24

Computational Data…………………………………………………………….27

Reference………………………………………………………………………..34

NMR spectra……………………………………………………………………35

Supplementary Figures

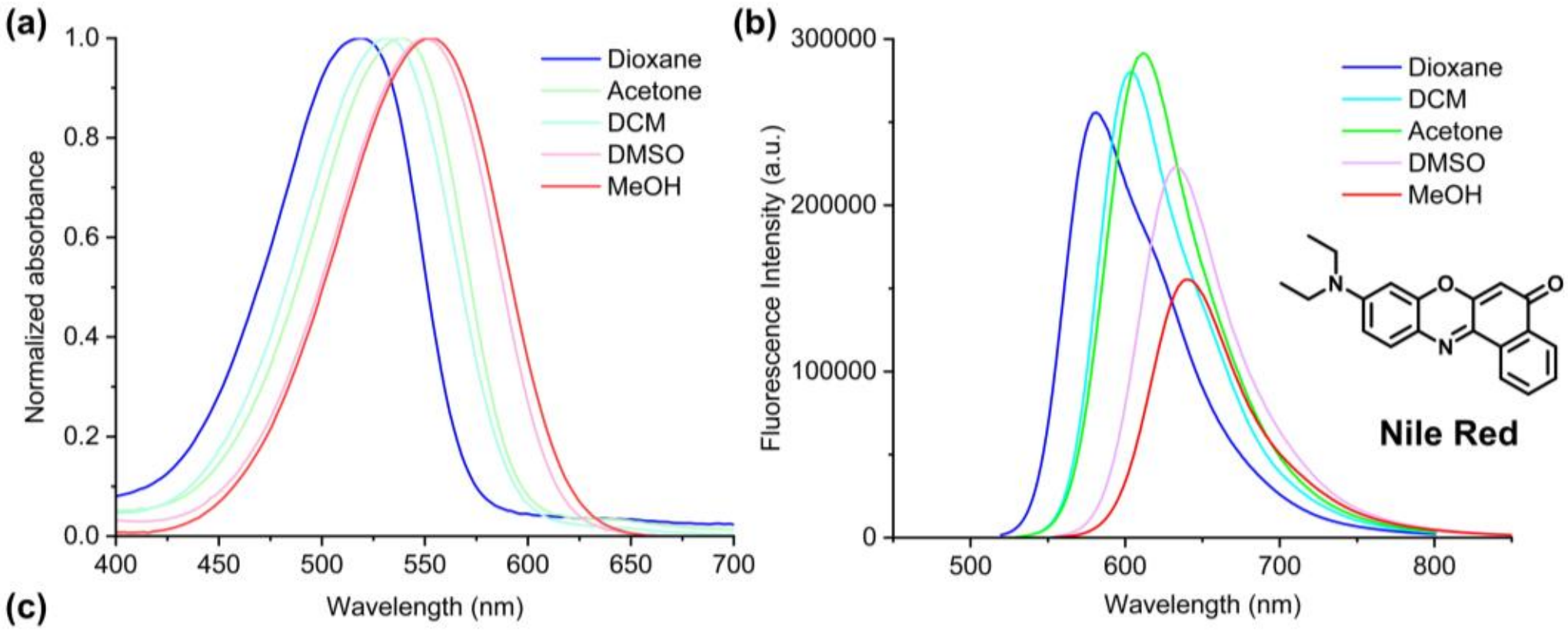

(c)

Solvent	λ^{abs}_{max} (nm)	λ^{em}_{max} (nm)	Solvatochromism (nm)	Stokes shift (nm)	Φ_f	ε [M^{-1} cm^{-1}]
Dioxane	520	581		61	0.82	29500
DCM	538	604		66	0.84	31000
Acetone	532	611	**59**	79	0.82	28500
DMSO	550	632		82	0.74	33000
MeOH	552	640		88	0.42	32600

Figure S1. Characterizations of Nile Red *in vitro*. (a). Normalized absorbance spectra of Nile Red (2 μM); (b). Emission spectra of Nile Red (2 μM); (c). Photophysical properties of Nile Red. Solvatochromism was observed by subtracting the maximum emission wavelength of Nile Red in methanol from that in dioxane.

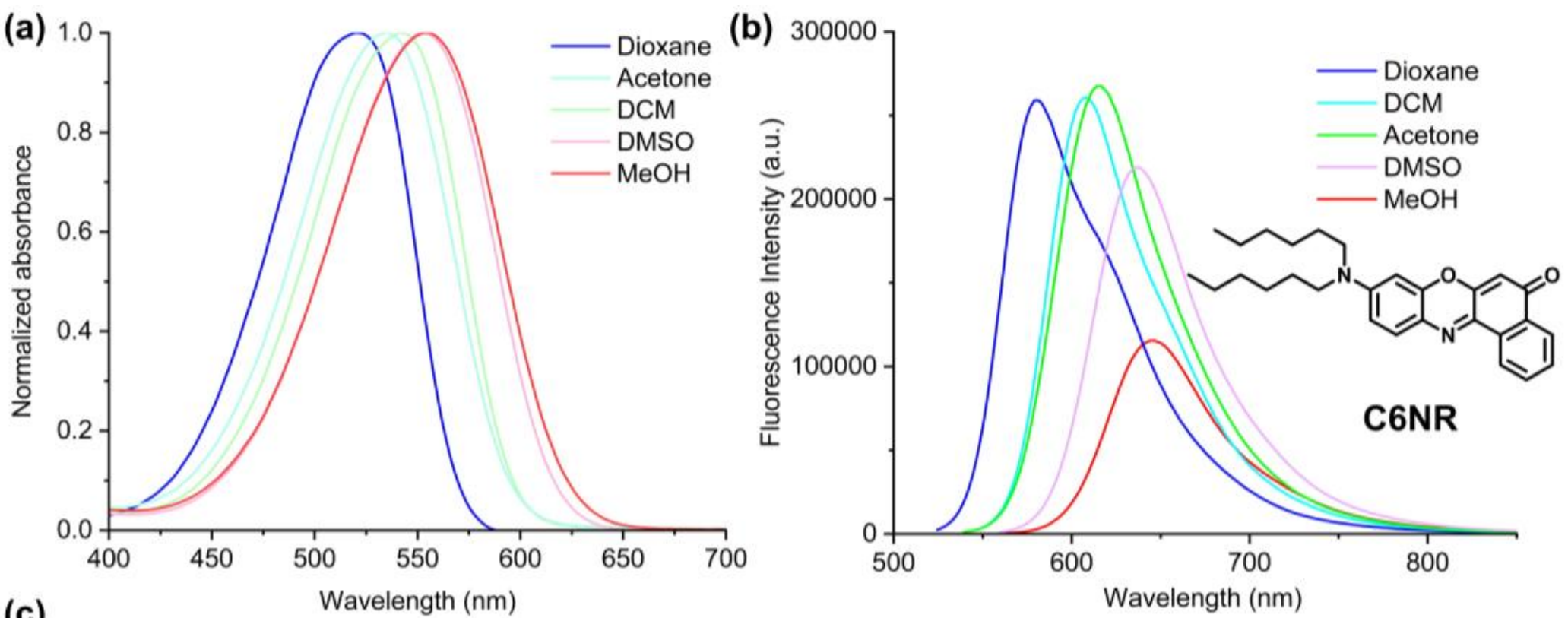

(c)

Solvent	λ^{abs}_{max} (nm)	λ^{em}_{max} (nm)	Solvatochromism (nm)	Stokes shift (nm)	Φ_f	ε [M^{-1} cm^{-1}]
Dioxane	520	580		60	0.81	30000
DCM	542	607		65	0.78	30800
Acetone	536	615	**64**	79	0.76	29000
DMSO	552	638		86	0.74	32000
MeOH	554	644		90	0.32	32800

Figure S2. Characterizations of C6NR *in vitro*. (a). Normalized absorbance spectra of C6NR (2 μM); (b). Emission spectra of C6NR (2 μM); (c). Photophysical properties of C6NR. Solvatochromism was observed by subtracting the maximum emission wavelength of C6NR in methanol from that in dioxane.

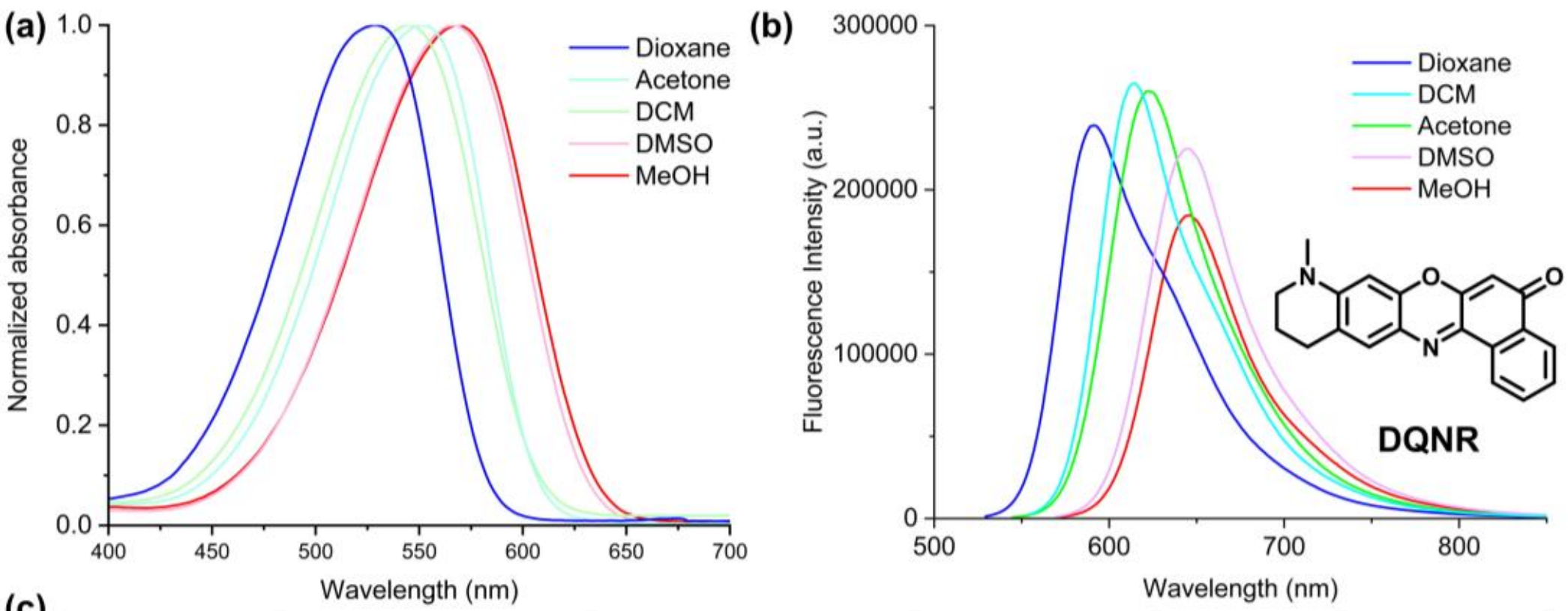

(c)

Solvent	λ^{abs}_{max} (nm)	λ^{em}_{max} (nm)	Solvatochromism (nm)	Stokes shift (nm)	Φ_f	ε [M^{-1} cm^{-1}]
Dioxane	528	591		63	0.74	28400
DCM	552	615		63	0.77	31400
Acetone	546	622	**55**	76	0.71	30500
DMSO	566	644		78	0.72	32000
MeOH	568	646		78	0.47	29000

Figure S3. Characterizations of DQNR *in vitro*. (a). Normalized absorbance spectra of DQNR (2 μM); (b). Emission spectra of DQNR (2 μM); (c). Photophysical properties of DQNR. Solvatochromism was observed by subtracting the maximum emission wavelength of DQNR in methanol from that in dioxane.

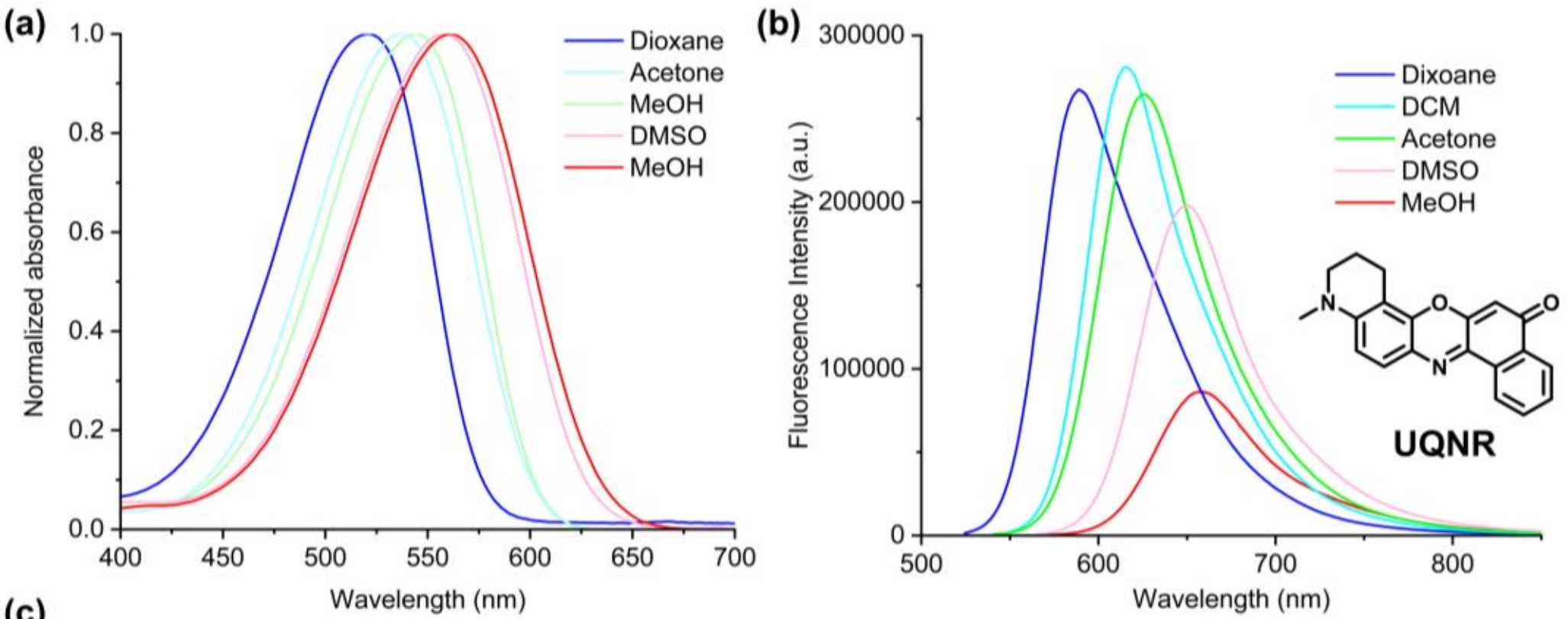

(c)

Solvent	λ^{abs}_{max} (nm)	λ^{em}_{max} (nm)	Solvatochromism (nm)	Stokes shift (nm)	Φ_f	ε [M^{-1} cm^{-1}]
Dioxane	522	589		67	0.82	29600
DCM	544	615		71	0.83	31000
Acetone	538	626	**65**	88	0.75	32400
DMSO	558	650		92	0.67	31700
MeOH	562	654		92	0.25	32300

Figure S4. Characterizations of UQNR *in vitro*. (a). Normalized absorbance spectra of UQNR (2 μM); (b). Emission spectra of UQNR (2 μM); (c). Photophysical properties of UQNR. Solvatochromism was observed by subtracting the maximum emission wavelength of UQNR in methanol from that in dioxane.

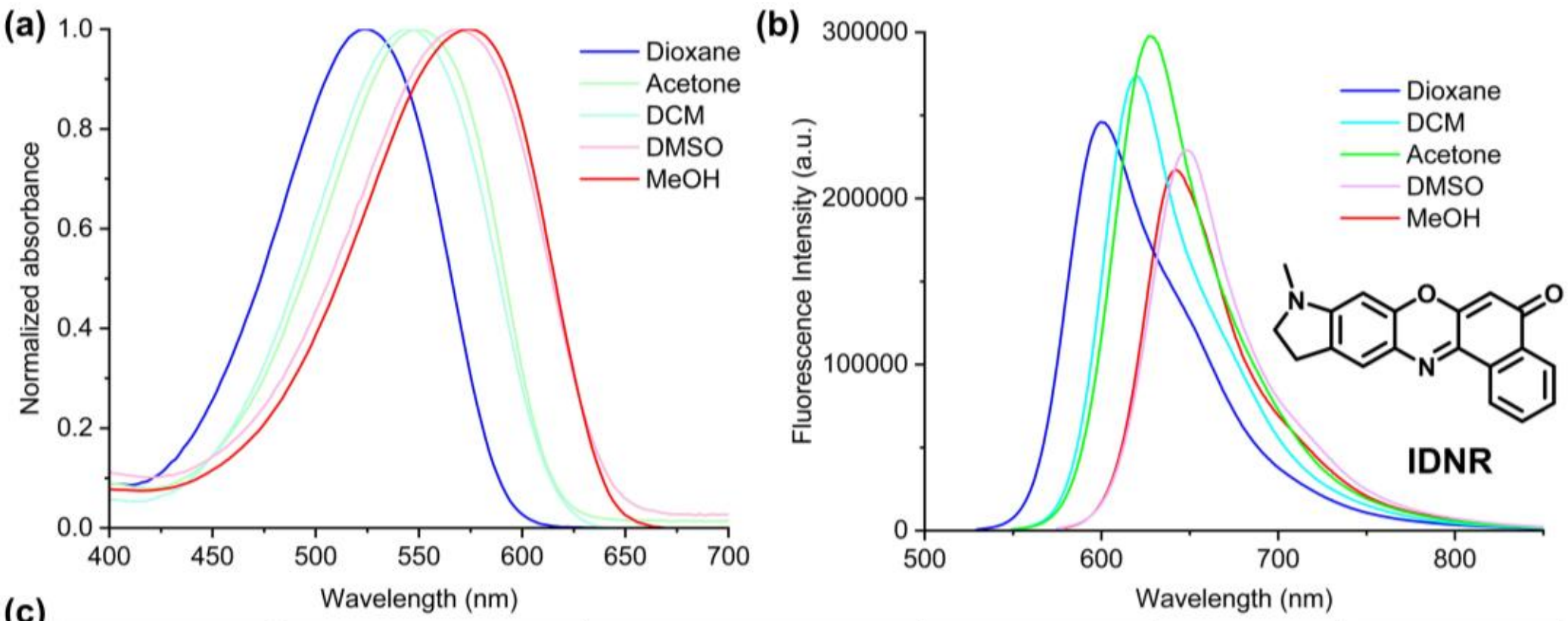

(c)

Solvent	λ^{abs}_{max} (nm)	λ^{em}_{max} (nm)	Solvatochromism (nm)	Stokes shift (nm)	Φ_f	ε [M^{-1} cm^{-1}]
Dioxane	524	600		76	0.76	28800
DCM	548	620		72	0.77	30000
Acetone	544	628	**42**	84	0.77	30800
DMSO	570	647		77	0.70	31000
MeOH	574	642		68	0.52	30600

Figure S5. Characterizations of IDNR *in vitro*. (a). Normalized absorbance of IDNR (2 μM); (b). Emission spectra of IDNR (2 μM); (c). Photophysical properties of IDNR. Solvatochromism was observed by subtracting the maximum emission wavelength of IDNR in methanol from that in dioxane.

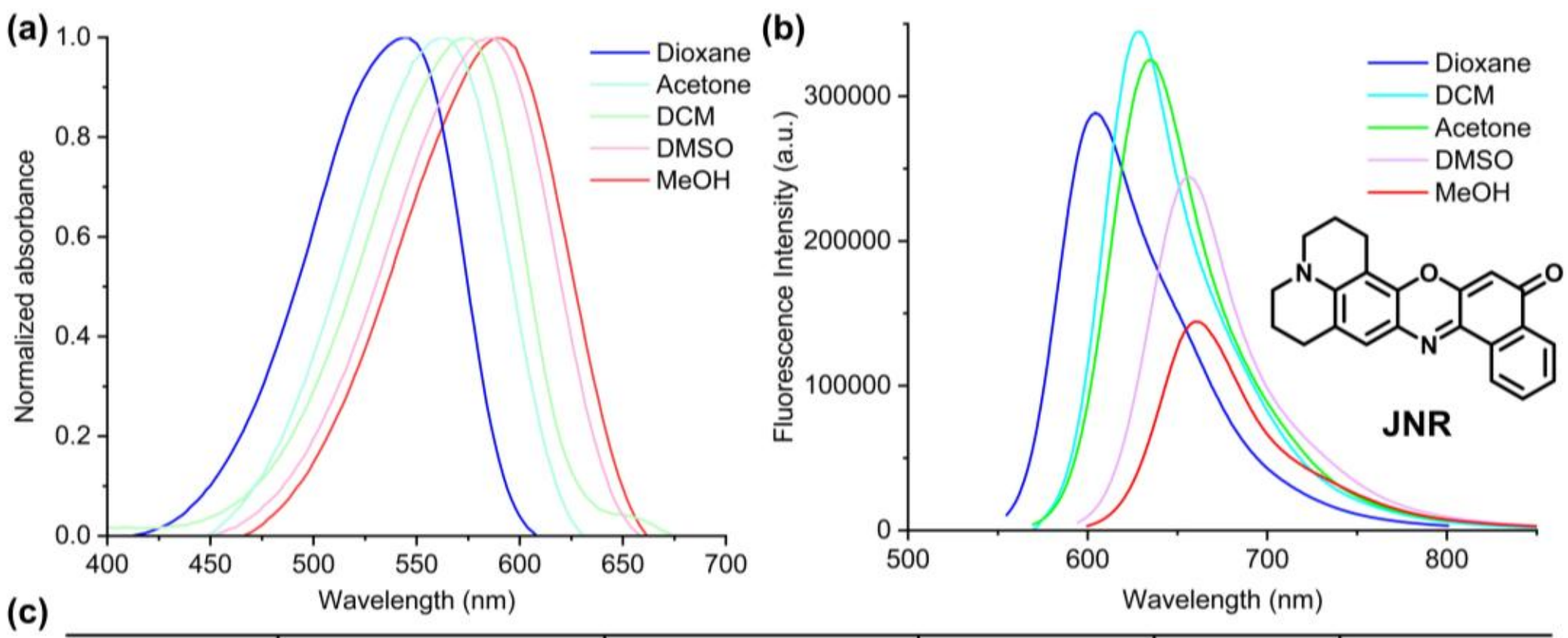

(c)

Solvent	λ^{abs}_{max} (nm)	λ^{em}_{max} (nm)	Solvatochromism (nm)	Stokes shift (nm)	Φ_f	ε [M^{-1} cm^{-1}]
Dioxane	544	604		60	0.85	28600
DCM	574	629		55	0.89	30000
Acetone	562	635	**57**	73	0.84	33000
DMSO	584	656		72	0.76	31800
MeOH	590	661		71	0.36	32400

Figure S6. Characterizations of JNR *in vitro.* (a). Normalized absorbance spectra of JNR (2 μM); (b). Emission spectra of JNR (2 μM); (c). Photophysical properties of JNR. Solvatochromism was observed by subtracting the maximum emission wavelength of JNR in methanol from that in dioxane.

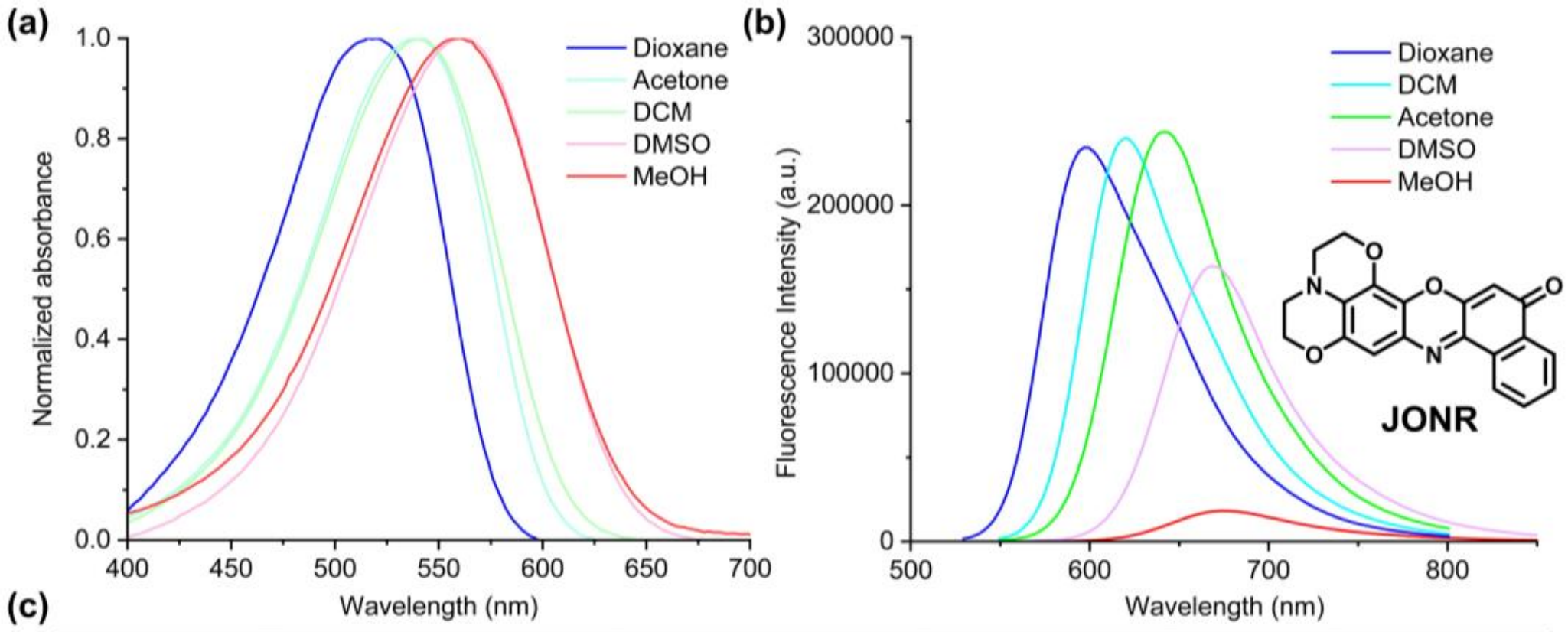

(c)

Solvent	λ^{abs}_{max} (nm)	λ^{em}_{max} (nm)	Solvatochromism (nm)	Stokes shift (nm)	Φ_f	ε [M^{-1} cm^{-1}]
Dioxane	516	599		83	0.75	32800
DCM	538	620		82	0.73	33000
Acetone	538	641	**75**	103	0.69	29600
DMSO	560	667		107	0.56	33200
MeOH	560	674		114	0.055	28400

Figure S7. Characterizations of JONR *in vitro*. (a). Normalized absorbance spectra of JONR (2 μM); (b). Emission spectra of JONR (2 μM); (c). Photophysical properties of JONR. Solvatochromism was observed by subtracting the maximum emission wavelength of JONR in methanol from that in dioxane.

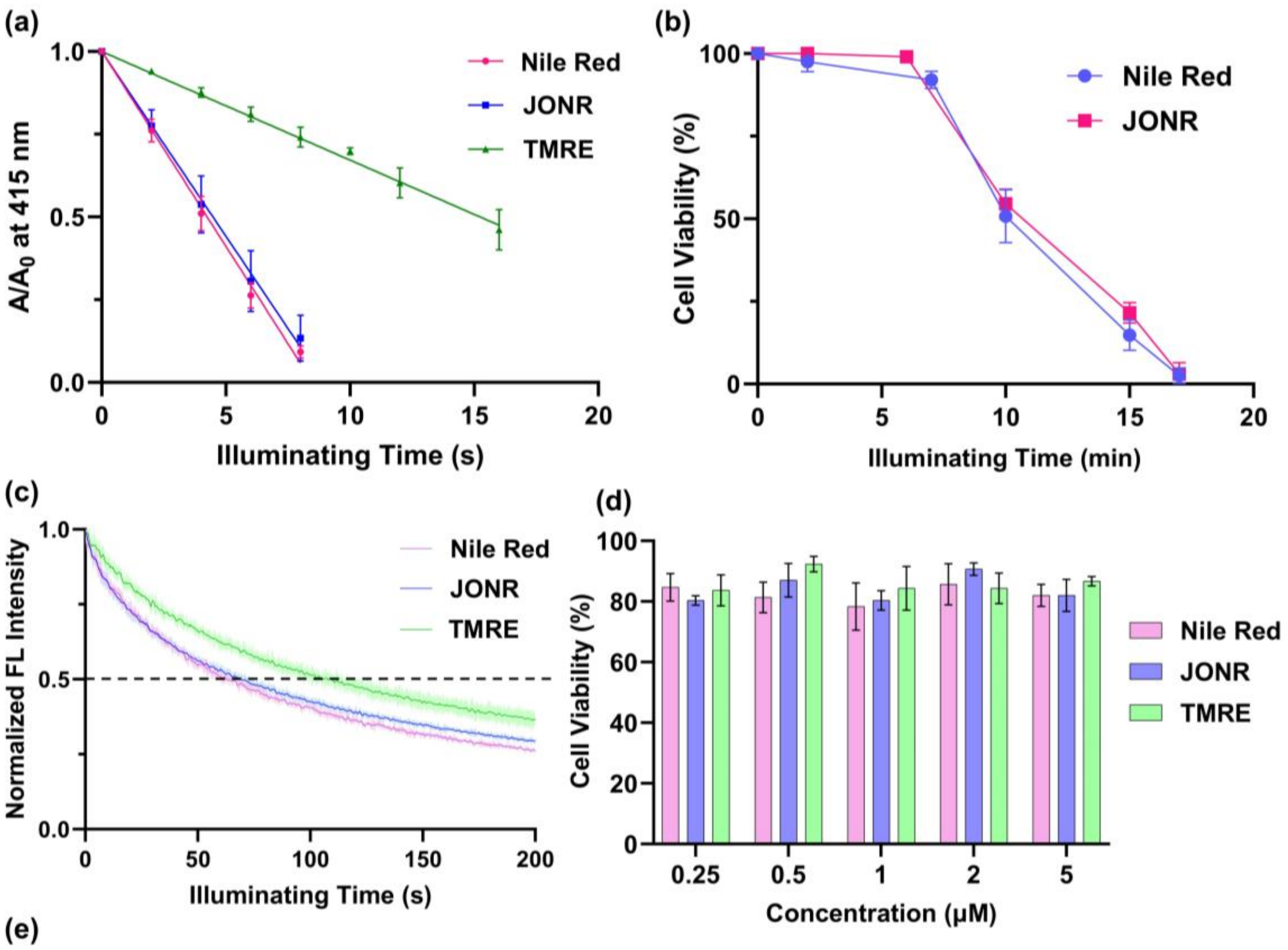

(e)

Compound	Absolute Singlet Oxygen Yield($\Phi_{\Delta}(^1O_2)$) Units: X 10^{-4} (Mean ± SD)	Half-lethal Light Dose (min)	Half-life of Photobleaching (s)
Nile Red	440 ± 6	~10	62
JONR	408 ± 10	~11	71
TMRE	120 ± 7		105

Figure S8. Cytotoxicity, phototoxicity and photostability of JONR. (a) *In vitro* singlet oxygen generation experiments of JONR and Nile Red. The maximum absorption of DPBF at 415 nm was measured under continuous irradiation with a 520-530 nm LED lamp in the presence of each dye (absorbance at 525 nm = 0.1; concentrations: JONR, 3.6 μM; Nile Red, 3.7 μM) in air-saturated MeOH. TMRE in MeOH (Φ_{Δ}= 0.012) in MeOH was selected as a standard. (b) Live-cell phototoxicity measurements of Nile Red and JONR in HeLa cells. Cell apoptosis of > 500 cells after 568 nm LED light illumination (2.6 W/cm^2) was examined at each time point of the three independent experiments. (c) Photobleaching curve of JONR, Nile Red and TMRE in polymethyl methacrylate (PMMA) film recorded under continuous 561 nm laser scanning of a confocal microscope. (d) Cytotoxicity assay of HeLa cells stained with JONR, Nile Red and TMRE. Cytotoxicity of HeLa cells treated with JONR, Nile Red and TMRE in different staining concentrations (0.25, 0.5, 1, 2, 5 μM) for 2 hours. (e) Table of singlet oxygen yield, phototoxicity and photostability of JONR, Nile Red and TMRE. Each experiment was independently repeated at least three times. Data are shown as mean ± SEM.

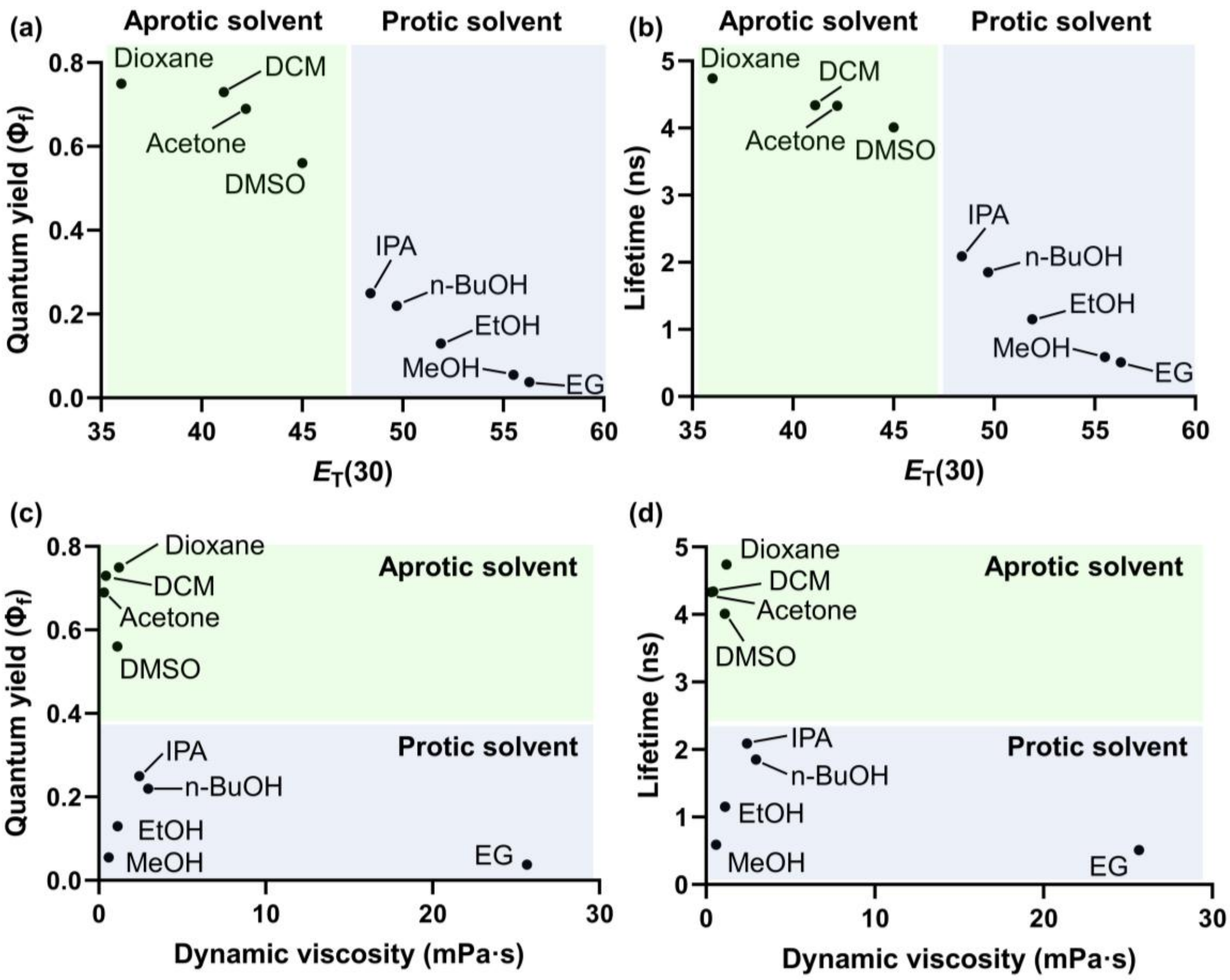

(e)

Solvent	$E_T(30)$	η (mPa·s, 25℃)	pKa (25℃)	λ^{em}_{max} (nm)	Φ_f	Lifetime (ns)
**Dioxane**	36.0	1.2	N.A.	599	0.75	4.74
**DCM**	41.1	0.43	N.A.	620	0.73	4.34
**Acetone**	42.2	0.316	N.A.	641	0.69	4.33
**DMSO**	45	1.1	N.A.	667	0.56	4.01
**IPA**	48.4	0.472	16.5	660	0.25	2.09
**n-BuOH**	49.7	0.594	16.0	663	0.22	1.85
**EtOH**	51.9	2.43	15.9	672	0.13	1.15
**MeOH**	55.5	2.95	15.5	674	0.055	0.59
**EG**	56.3	25.66	14.2	687	0.038	0.51

Figure S9. Relationship between the photophysical properties of JONR and solvent polarity and viscosity. (a) Relationship between the solvent polarity (measured by $E_T(30)$) and the quantum yield of JONR. (b) Relationship between the solvent polarity and the fluorescence lifetime of JONR. (c) Relationship between the solvent viscosity (using dynamic viscosity at 25 ℃) and the quantum yield of JONR. (d) Relationship between the solvent viscosity and the fluorescence lifetime of JONR. (e) Table of the polarity, viscosity, pKa of the solvents, and maximum emission wavelength, fluorescence quantum yields, and fluorescence lifetimes of JONR in different solvents.

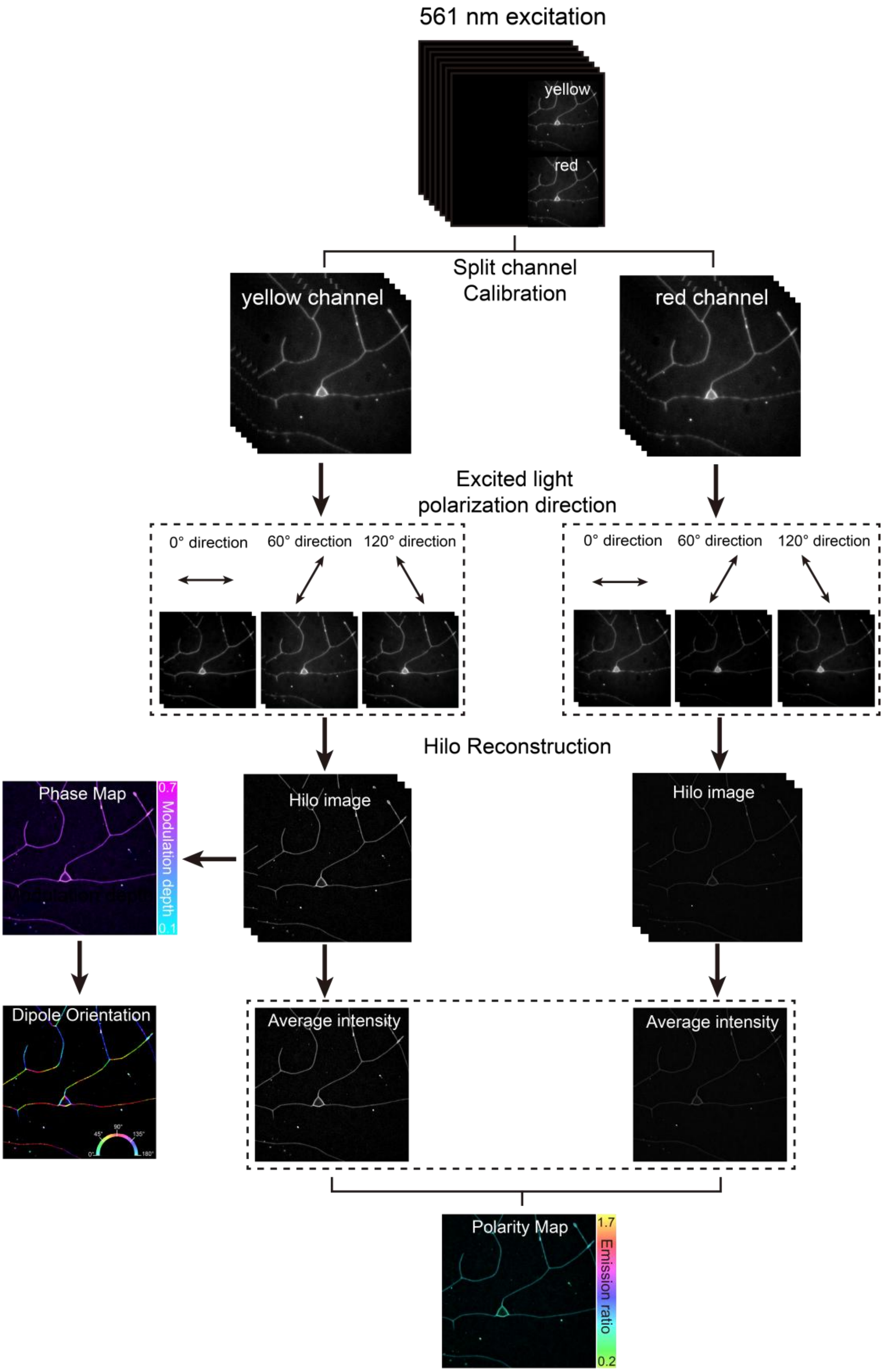

Figure S10. Principle of Spectrum and Polarization Optical Tomography (SPOT). The detailed reconstruction process of SPOT includes the measurement of emission ratio and modulation depth, as well as the mapping of dipole orientation.

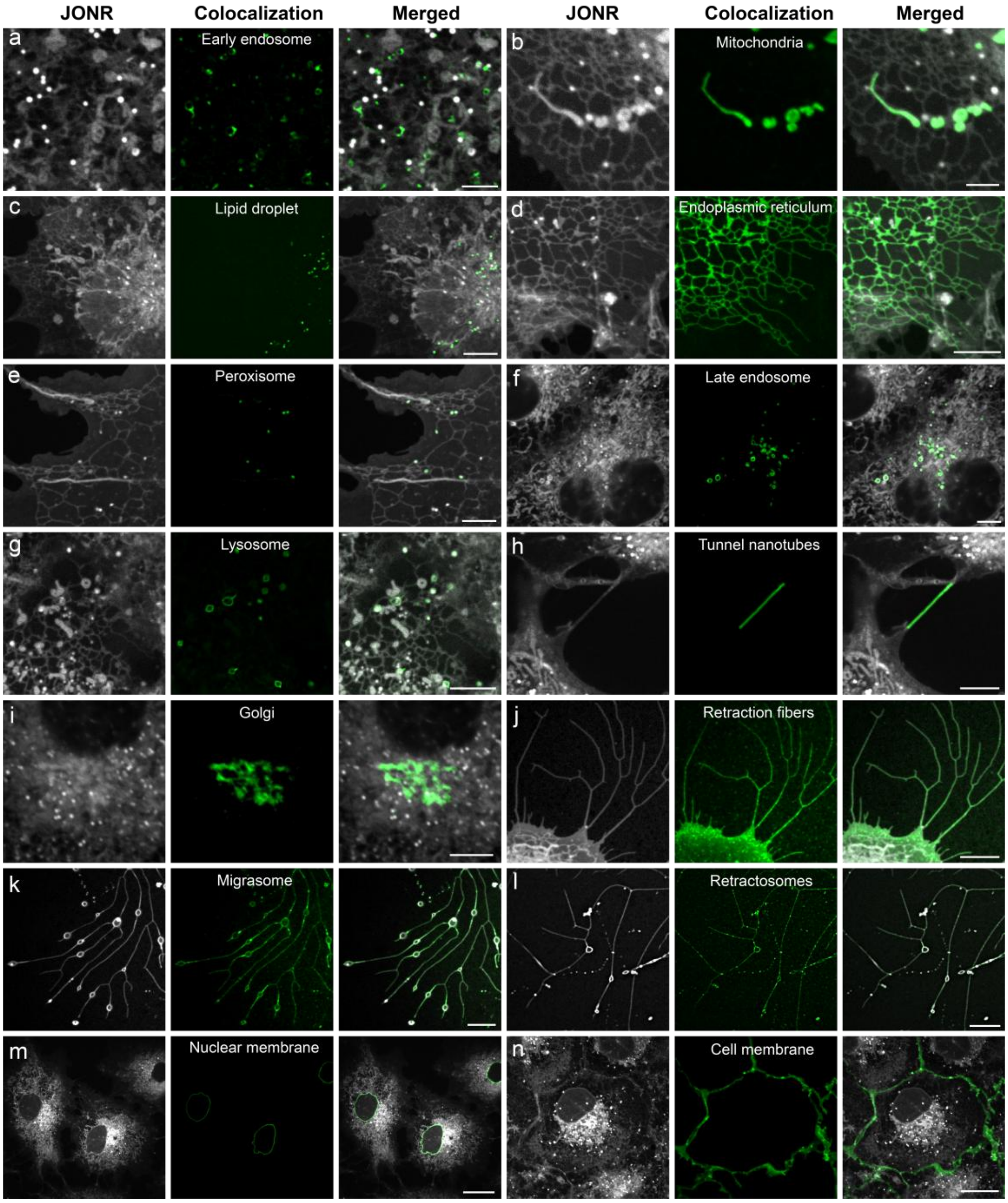

Figure S11. Colocalization of JONR with fluorescent proteins or dyes in live cells. The images in each left column are fluorescent images of JONR in the yellow channel (excitation: 561 nm, emission: 578-614 nm). The images in the middle are the fluorescent images in the green channel (excitation: 488 nm, emission: 505-545 nm). The images on the right are the colocalization of Nile Red signals in both green and yellow emission channels. All the experimental results were repeated at least three times with independently prepared samples. Scale bar: 3 μm (a, b); 5 μm(c-l); 15 μm (m, n).

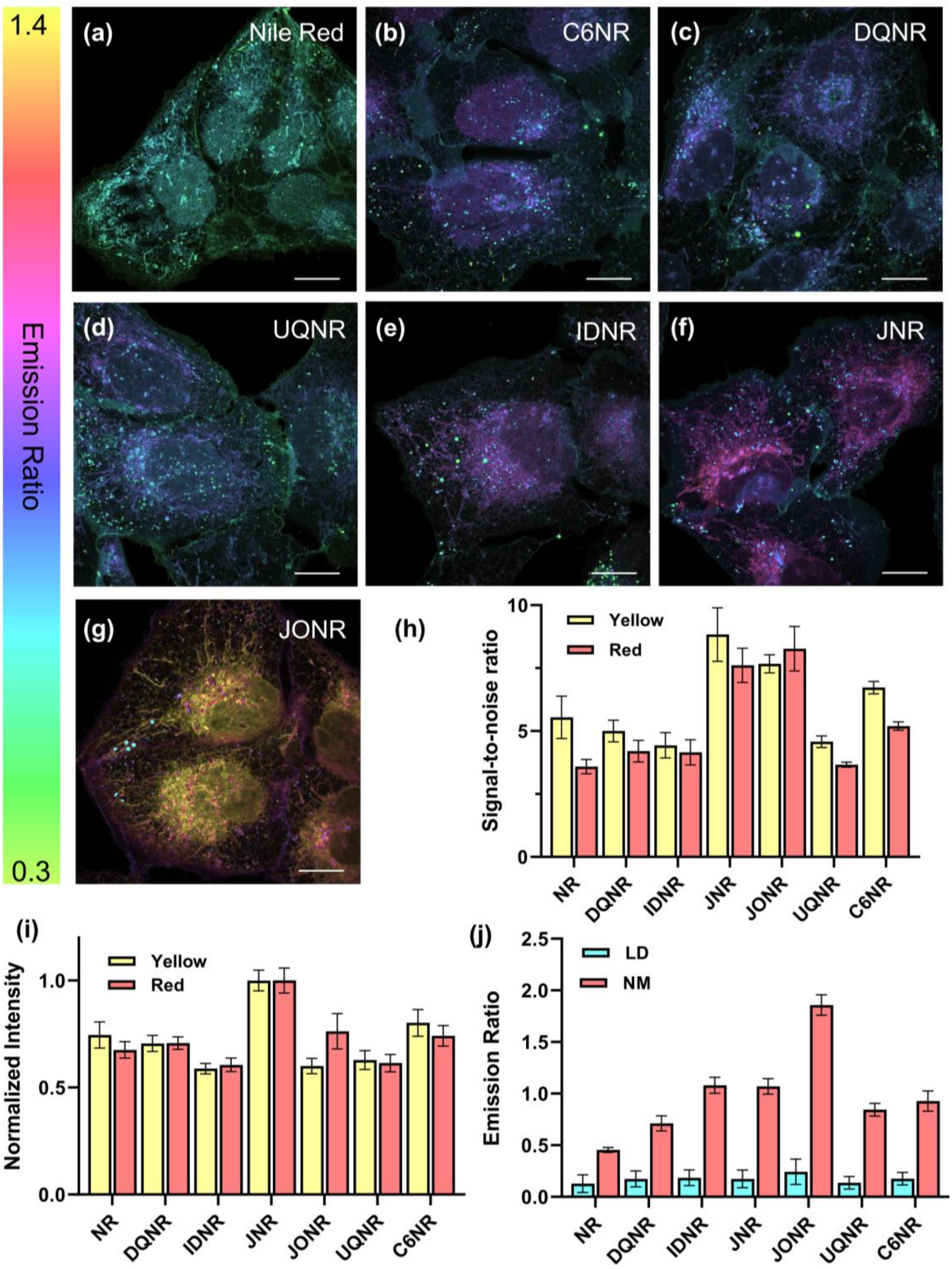

Figure S12. Comparison of imaging performance of Nile Red and derivatives in live cell ratiometric imaging. (a-g) Polarity map of COS-7 cells showing the spatial distribution of membrane polarity based on Nile Red (a), C6NR (b), DQNR (c), UQNR (d), IDNR (e), JNR (f) and JONR (g) staining. (h) Comparison of signal-to-noise ratios (SNR) in mitochondria in two channels stained with different probes. (i) Comparison of brightness in two channels stained with different probes. (j) Comparison of emission ratios in lipid droplets (LD) and nuclear membranes (NM) stained with different probes. Each experiment was independently repeated at least five times. Data are shown as mean ± SEM. Scale bars: 2 μm.

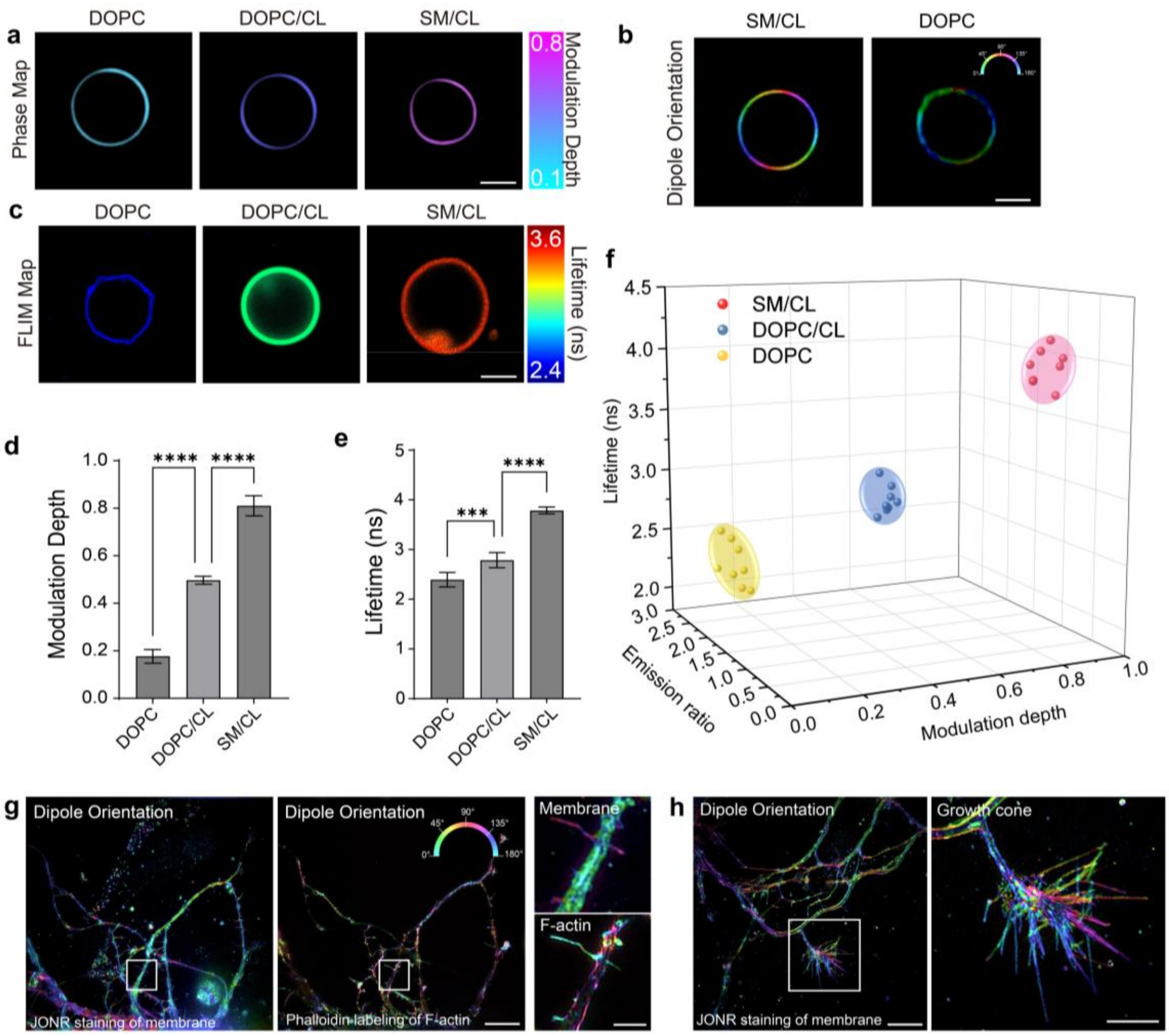

Figure S13. Multidimensional responsiveness of JONR to different lipid compositions. (a) Phase maps of GUVs with different lipid compositions obtained by SPOT imaging, where the color bar indicates the range of modulation depth values. (b) Orientation maps of GUVs composed of different lipid compositions. GUVs composed of SM/CL exhibit more accurately resolved dipole orientations, whereas those composed of pure DOPC show reduced dipole resolution. The hue of the color wheel reflects dipole orientation. (c) FLIM maps of GUVs with different lipid compositions, where the color bar represents the range of fluorescence lifetime values. (d) Quantification of modulation depth values for GUVs with different lipid compositions, corresponding to the phase maps shown in (b). (e) Quantification of fluorescence lifetime values for GUVs with different lipid compositions, corresponding to the FLIM maps shown in (c). (f) Multidimensional mapping of GUVs with different lipid compositions, plotted in three-dimensional coordinates. Solid spheres represent sampling points for different lipid compositions, and the transparent ellipsoids represent the coverage range of all data points. (g) Membrane structures labeled by JONR (left) and F-actin filaments labeled by Phalloidin-Alexa488 (right) in fixed mouse hippocampal neurons. The color coding of the orientation ring corresponds to the angles indicated above. A magnified view of a local region is shown on the right. (h) Dipole orientation mapping of membrane structures labeled by JONR in hippocampal neurons (left panel). A magnified view of the growth cone region is shown (right panel). Each experiment in GUVs was independently repeated more than five times. Data are shown as mean ± SEM. ***$p < 0.001$; ****$p < 0.0001$ (one-way ANOVA). Scale bars: 2 µm (g right), 3 µm (a, b, c),5 µm (h right) and 10 µm (g left, h left).

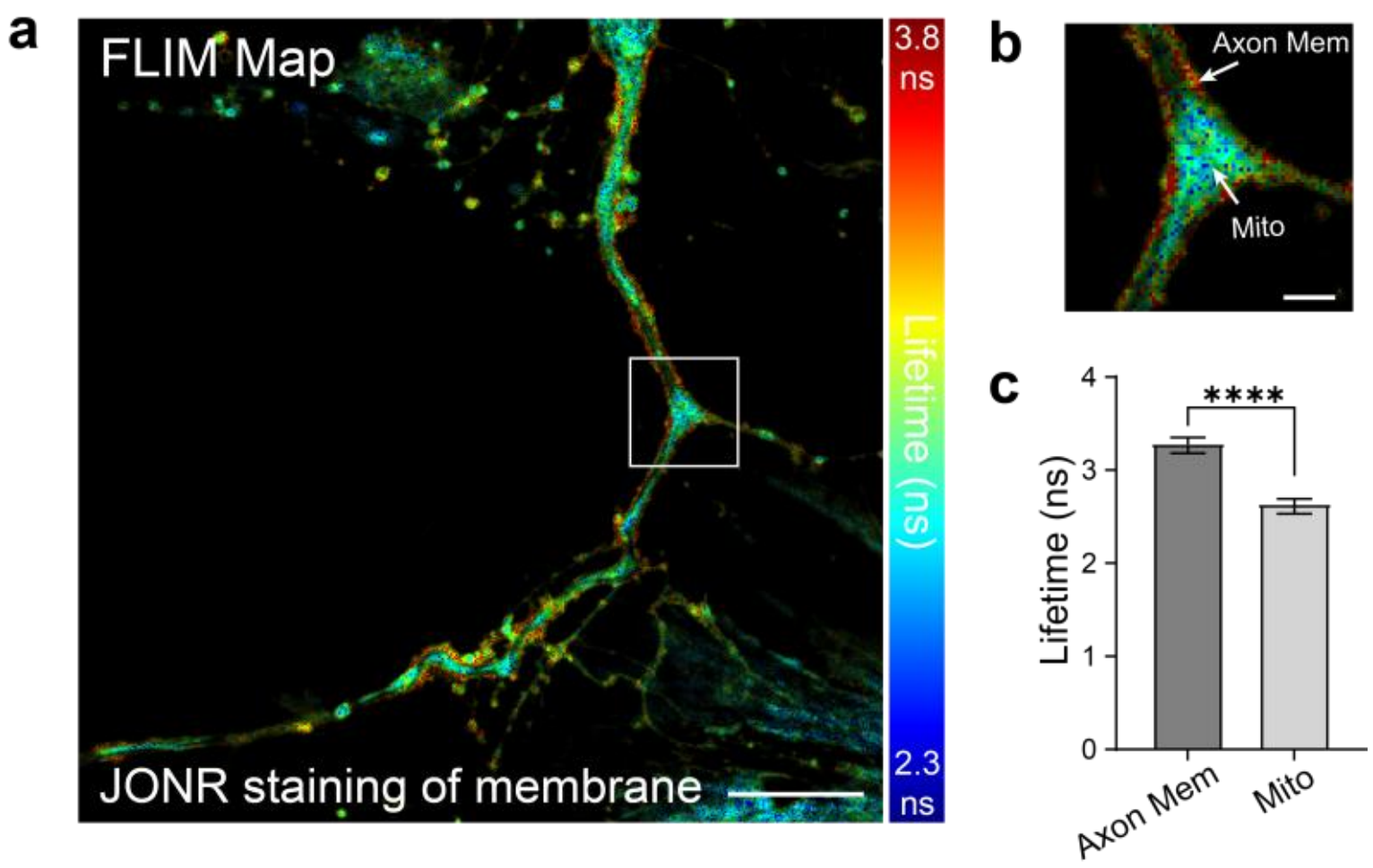

Figure S14. Fluorescence lifetime imaging of neurons labeled with JONR. (a) Representative FLIM image of a neuron membrane stained with JONR. (b) Enlarged views from (a) showing the fluorescence lifetime of the axonal membrane and intra-axonal mitochondria. (c) Statistical comparison reveals a significantly longer fluorescence lifetime in the axonal membrane compared to mitochondria (****P < 0.0001, t-test). Data are shown as mean ± SEM. Data are representative of three independent experiments. Scale bars: 10 μm (a), 2 μm (b).

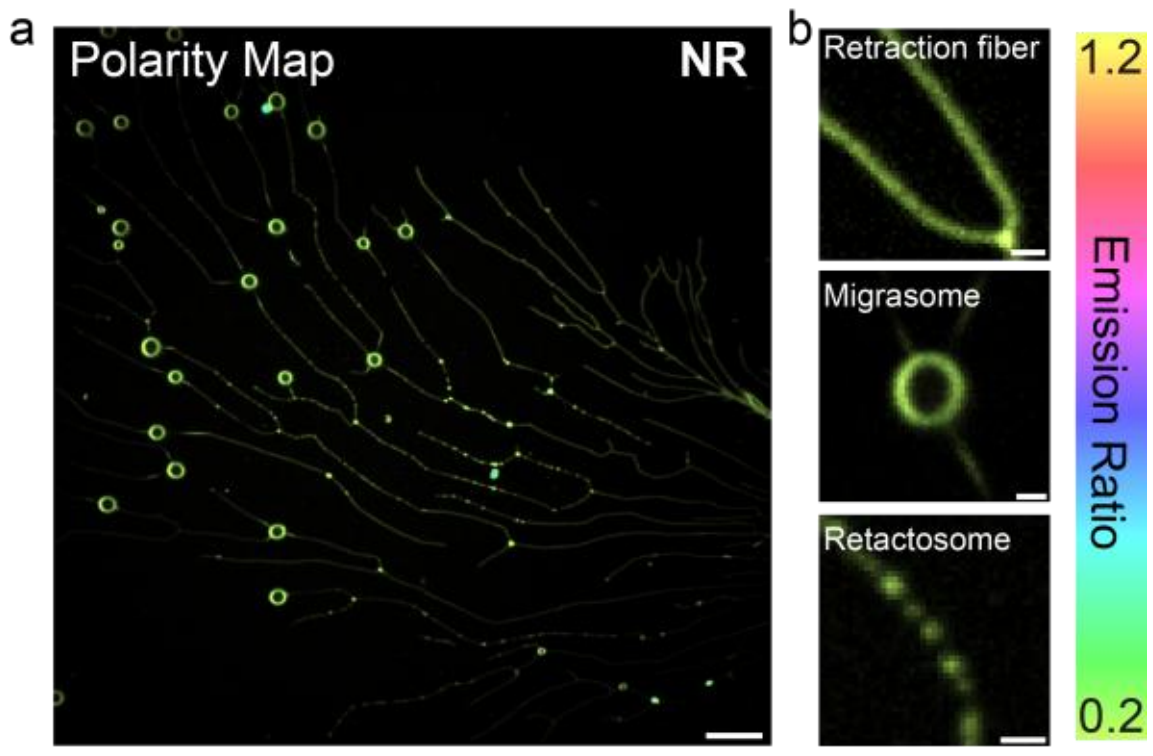

Figure S15. Ratiometric imaging of Nile Red-labeled L929 cells. (a) Overview of migration-related structures in L929 cells. (b) In specific migration-associated structures such as retraction fibers, migrasomes, and retractosomes, the ratio imaging based on Nile Red shows limited ability to distinguish subtle variations in lipid composition. Scale bars: 5 μm (a); 500 nm (b).

Experimental Details

Fluorescence lifetime measurement

JONR was dissolved in spectral grade solvents (dioxane, acetone, DCM, DMSO, MeOH, CD_3OD, EtOH, IPA, n-BuOH, EG) for fluorescence lifetime measurements. A 10 mM stock solution was diluted with different solvents to 2 μM. Fluorescent lifetime measurement was conducted using an FLS980 Spectrometer (Edinburgh Instruments) containing an integrating sphere at room temperature within a 1 cm square quartz cuvette. Fluorescence lifetime data were analyzed using the Fluoracle software (Edinburgh Instruments) by fitting a mono-exponential decay model (n-exponential reconvolution) to the decay. The instrument response function (IRF) used for deconvolution was measured under the same instrument settings. Measurements were carried out at their emission wavelength.

Computational methods

All the DFT calculations were performed by the Gaussian 16 package.[1] Geometry optimization of all structures was performed using the TPSSh functional and the TZVP basis set.[2,3] Frequency calculations were performed to obtain the Gibbs free energy at 298.15 K. The SMD implicit solvation model was used for all calculations, and each S1 state was optimized in the corresponding solvent.[4] The molecular geometry was plotted with CYLView.[5] The isosurface was plotted using VMD.[6] The electron-hole analysis was performed using Multiwfn.[7]

Fixed cell staining process

In general, neuron and L929 cells were fixed with 4% paraformaldehyde (I28800, Thermo Scientific) at room temperature for 10 minutes, followed by PBS buffer (pH =7.4) washes. To label actin filaments, cells were incubated with 1 μM phalloidin–Alexa Fluor 488 (A12379, Invitrogen) for 1 hour at room temperature. After PBS washes, cells were subsequently incubated with 1 μM JONR for 15 minutes to stain lipid membrane structures. Cells were then thoroughly washed with PBS before imaging.

Viability-based phototoxicity assay of HeLa cells

HeLa cells were grown to 80-90 % confluency in a 96-well plate (Costar 3599; Corning, NY, USA) before treatment. To achieve the same brightness after staining, HeLa cells were incubated with 1 μM JONR and 1 μM Nile Red respectively in DMEM (Gibco) for 10 min at 37 °C with 5% CO_2. Then, the cells were washed with PBS three times and maintained in fresh medium for subsequent imaging analysis. 96-well plates were analyzed using a high-content imaging system ImageXpress Micro XLS (Molecular Devices, San Jose, CA, USA), equipped with a 20×/0.4 NA air objective and live-cell imaging device. The average light intensity on cells was measured to be 2.6 W/cm^2 (568 nm). The cells in each well were irradiated with the maximum light intensity at different time points. Time points were selected as 2, 5, 6, 15 and 17 min (each time point with three parallel repeats).

After illumination, the cells were directly used for subsequent imaging analysis. The cells were washed with PBS once and a 100 μL cell viability assay solution containing 1 μM Calcein AM

(Beyotime, Shanghai, China) was added to each sample including controls (without dye incubation). Images of two channels (mitochondria: Cy3 channel, 568 nm; Calcein AM: FITC channel, 488 nm) were recorded on ImageXpress Micro XLS. The cell viability (%) was calculated according to the following equation: cell viability% = B/A *100%, where A = the number of total cells before illumination, and B = the number of Calcein AM-positive cells after illumination. More than 500 cells were counted at each time point.

Measurement of cytotoxicity using Cell Counting Kit-8 (CCK8) reagent

HeLa cells were grown to 80-90 % confluency in a 96-well plate (Costar 3599, Corning) the day before treatment. Cells were either stained with TMRE, JONR, and Nile Red with 5 different concentrations (0.25, 0.5, 1, 2, 5 μM in DMEM) for 12 hours at 37 °C with 5% CO_2. Cells were washed with PBS once after the staining procedure and incubated with cultured medium containing 10% Cell Counting Kit-8 reagent (CCK8; C0038; Beyotime) for 2 hours at 37 °C with 5% CO_2. The absorbance (450 nm) of each well was recorded using a TECAN Infinite M Nano+ microplate reader (TECAN, Männedorf, Switzerland).

Colocalization imaging of live-cell samples

To validate the effectiveness of JONR in labeling membrane-bound cellular structures, we performed spectral ratio imaging using JONR staining while simultaneously labeling individual membrane-bound organelles with fluorescent dyes or fluorescent proteins in live cells. In addition to spectral ratio imaging, a green detection channel (488 nm laser excitation, 525 ± 20 nm emission detection) was introduced to enable colocalization analysis. This approach allowed for the identification and characterization of over ten distinct membrane-bound subcellular structures.

For COS-7 cells, specific membrane-bound organelles were labeled using either plasmids or fluorescent dyes to achieve colocalization imaging. Peroxisomes were labeled with EGFP–SKL, late endosomes with mEGFP–Rab7a, early endosomes with GFP–Rab5, lysosomes with EGFP–LAMP1, Golgi apparatus with GFP–SiT, and the endoplasmic reticulum (ER) with mEmerald–Sec61b. Transfection was performed using Lipofectamine 3000 (Life Technologies) according to the manufacturer's protocol. Briefly, cells were transfected in Opti-MEM (Life Technologies) with Lipofectamine 3000 per well, incubated for 6 hours, and then replaced with standard culture medium. In addition to plasmid-based labeling, mitochondria were labeled with MitoTracker (A66468, Invitrogen) at a concentration of 250 nM for 20 minutes, followed by a medium exchange. Nuclei were labeled with NucBlue™ Live ReadyProbes™ Reagent (Hoechst, 33342) at 200 nM for 10 minutes. Migrasomes, plasma membranes, tunneling nanotubes, retraction fibers, and retractosomes were labeled with 100 nM Alexa Fluor 488-conjugated WGA (W11261, Invitrogen) for 10 minutes and manually annotated.

Data Availability Statement

Data presented in this study are provided in Supplementary Tables. Data are available from the corresponding authors upon request.

Ethics approval and consent to participate

Primary cell isolation procedures were approved by the Peking University Animal Use and Care Committee and complied with the standards of the Association for Assessment and Accreditation of

Laboratory Animal Care (code number IMM-ChenZX-1).

General procedures on synthetic chemistry

Unless otherwise stated, all commercially available materials were purchased at the highest commercial quality and used without further purification. Anhydrous DMF was purchased from MREDA (China), and anhydrous THF was purchased from Innochem (China). All reactions were carried out with dry solvents, unless otherwise mentioned.

Reactions were monitored by Thin Layer Chromatography on plates (GF254) (Yantai Chemicals) using UV light as visualizing agent or by LC/MS (4.6 mm × 150 mm 5 μm C18 column; 5 μL injection; 5-100% CH_3CN/H_2O, linear gradient, with constant 0.1 % v/v formic acid additive; 6 min run; 0.5 mL/min flow; ESI; positive or negative ion mode; detection at 550 nm). If not specially mentioned, flash column chromatography uses silica gel (200-300 mesh) supplied by Tsingtao Haiyang Chemicals (China).

NMR spectra were recorded on Brüker Advance 400 (^{1}H 400 MHz, ^{13}C 101 MHz) and are calibrated using residual undeuterated solvent ($CDCl_3$ at 7.26 ppm ^{1}H NMR, 77.16 ppm ^{13}C NMR; CD_3OD at 3.31 ppm ^{1}H NMR, 49.00 ppm ^{13}C NMR). In order to improve the solubility, several samples were dissolved in a mixture of $CDCl_3$ and CD_3OD and have been marked below. Data for ^{1}H NMR spectra are reported as follows: chemical shift (δ ppm), multiplicity (s = singlet, d = doublet, t = triplet, dd = doublet of doublets, m = multiplet, br = broad), coupling constant (Hz), integration. Data for ^{13}C NMR are reported by chemical shift (δ ppm).

Chemical synthesis

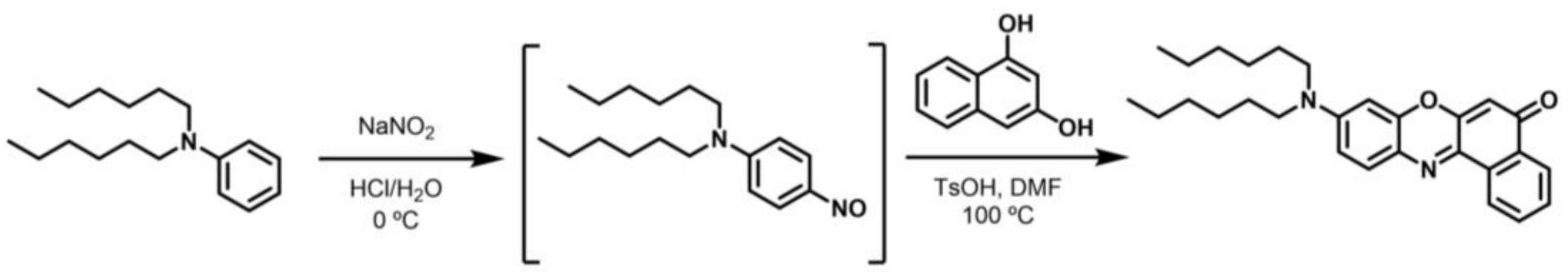

1

C6NR

C6NR: N,N-dihexylaniline (**1**, 1.00 g, 3.83 mmol) was dissolved in a mixture of concentrated HCl (2.00 mL, 23.1 mmol) and H_2O (1.20 mL), and the reaction mixture was cooled to 0 °C. A solution of $NaNO_2$ (317 mg, 4.60 mmol, 1.2 eq) in H_2O (3 mL) was added dropwise over 5 min, and the reaction mixture was stirred for another 8 min at 0 °C (ice bath). The reaction mixture was diluted with ice water, and a saturated solution of $NaHCO_3$ was slowly added to give a neutral solution. The product was extracted with DCM (3 × 10 mL), and the combined organic extracts were concentrated *in vacuo*. A solution of 1,6-naphthalenediol (674 mg, 4.21 mmol, 1.1 eq) and *p*-TsOH (65 mg, 0.380 mmol, 0.1 eq) in anhydrous DMF (10 mL) was added to the crude intermediate. The resulting reaction mixture was stirred at 100 °C for 10 h. After cooling to room temperature, the mixture was extracted with EtOAc (2 × 25 mL). The combined organic layer was washed with H_2O (20 mL), and then dried over anhydrous Na_2SO_4, and filtered. After concentration of the filtrate under reduced pressure, the crude product was purified by silica gel column chromatography (0-2% MeOH in DCM) to afford **C6NR** (1.45 g, 3.37 mmol, 88% yield).

^{1}H NMR (400 MHz, Methanol-*d*$_4$) δ 8.61 (d, *J* = 8.0 Hz, 1H), 8.21 (d, *J* = 7.8 Hz, 1H), 7.73 (t, *J* = 7.0 Hz, 1H), 7.67 – 7.62 (m, 1H), 7.60 – 7.54 (m, 1H), 6.69 (s, 1H), 6.43 (s, 1H), 6.36 – 6.31 (m, 1H), 3.38 (s, 4H), 1.66 (s, 4H), 1.37 (s, 12H), 0.96 – 0.88 (m, 6H).

^{13}C NMR (101 MHz, Methanol-*d*$_4$) δ 184.34, 152.69, 151.69, 146.75, 138.39, 132.05, 131.43, 131.17, 131.12, 129.62, 125.29, 125.20, 123.66, 110.67, 104.54, 96.05, 51.36, 31.52, 27.15, 26.53, 22.48, 13.52.
MS (ESI) calculated for $C_{28}H_{35}N_2O_2$ (MH^+) 431.3, observed 431.0.

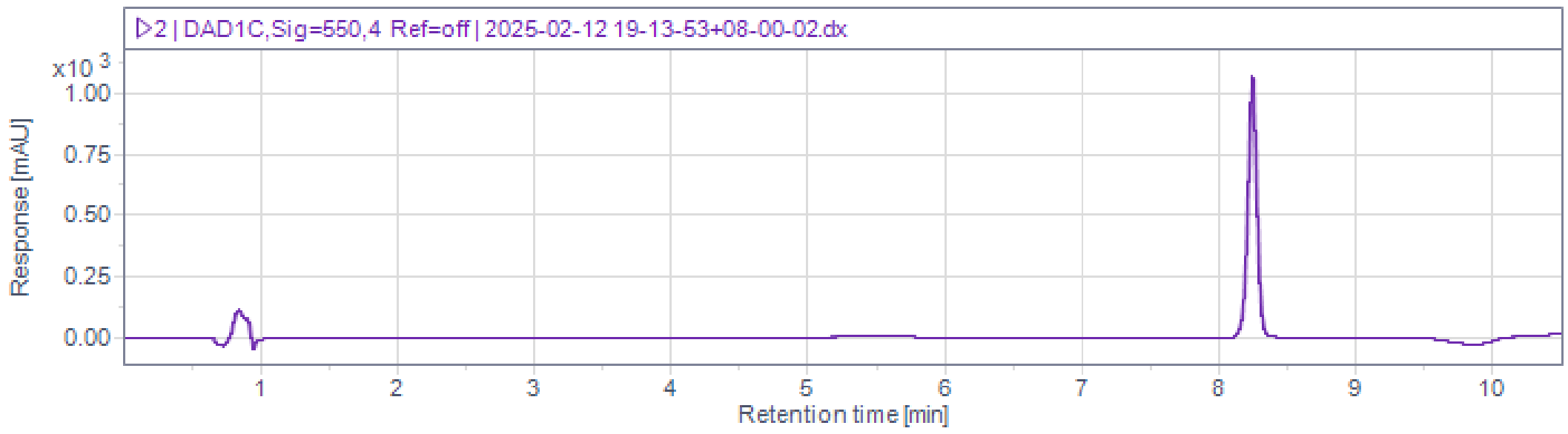

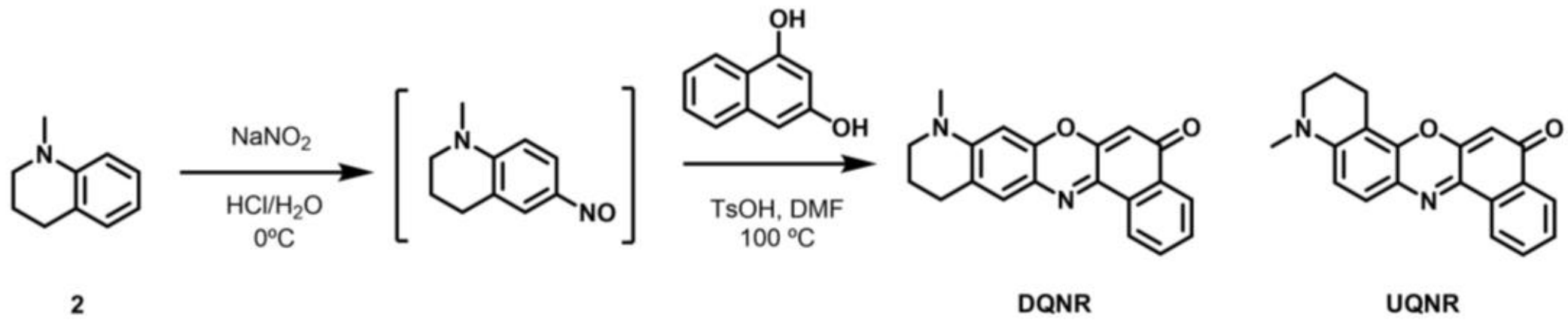

DQNR & UQNR: 1-methyl-1,2,3,4-tetrahydroquinoline (**2**, 1.00 g, 6.80 mmol) was dissolved in a mixture of concentrated HCl (2.00 mL, 23.1 mmol) and H_2O (1.20 mL), and the reaction mixture was cooled to 0 °C. A solution of $NaNO_2$ (563 mg, 8.16 mmol, 1.2 eq) in H_2O (3 mL) was added dropwise over 5 min, and the reaction mixture was stirred for another 8 min at 0 °C (ice bath). The reaction mixture was diluted with ice water, and a saturated solution of $NaHCO_3$ was slowly added to give a neutral solution. The product was extracted with DCM (3 × 10 mL), and the combined organic extracts were concentrated *in vacuo*. A solution of 1,6-naphthalenediol (1.20 g, 7.48 mmol, 1.1 eq) and *p*-TsOH (117 mg, 0.680 mmol, 0.1 eq) in anhydrous DMF (10 mL) was added to the crude intermediate and the resulting reaction mixture was stirred at 100 °C for 10 h. After cooling the reaction mixture to room temperature, the mixture was extracted with EtOAc (2 × 25 mL). The combined organic layer was washed with H_2O (20 mL), and then dried over anhydrous Na_2SO_4, and filtered. After concentration of the filtrate under reduced pressure, the crude product was purified by silica gel column chromatography (0-10% MeOH in DCM) to afford **DQNR** (860 mg, 2.72 mmol, 50% yield) and **UQNR** (709 mg, 2.24 mmol, 23% yield). **UQNR** was characterized by NMR using mixed solvents of $CDCl_3$ and CD_3OD (1:2).
^{1}H NMR (**DQNR**, 400 MHz, Chloroform-*d*) δ 8.64 (d, *J* = 7.1 Hz, 1H), 8.33 (d, *J* = 7.7 Hz, 1H), 7.75 – 7.70 (m, 1H), 7.68 – 7.63 (m, 1H), 7.31 (s, 1H), 6.38 (s, 1H), 6.32 (s, 1H), 3.44 – 3.38 (m, 2H), 3.00 (s, 3H), 2.80 (t, *J* = 6.2 Hz, 2H), 2.00 (p, *J* = 6.1 Hz, 2H).
^{13}C NMR (**DQNR**, 101 MHz, Chloroform-*d*) δ 183.46, 152.23, 149.60, 145.51, 139.39, 132.06, 131.71, 131.13, 129.70, 129.02, 125.62, 125.08, 123.68, 121.19, 105.23, 95.16, 51.06, 39.20, 27.27, 21.57.
MS (**DQNR,** ESI) calculated for $C_{20}H_{17}N_2O_2$ (MH^+) 317.1, observed 317.1.

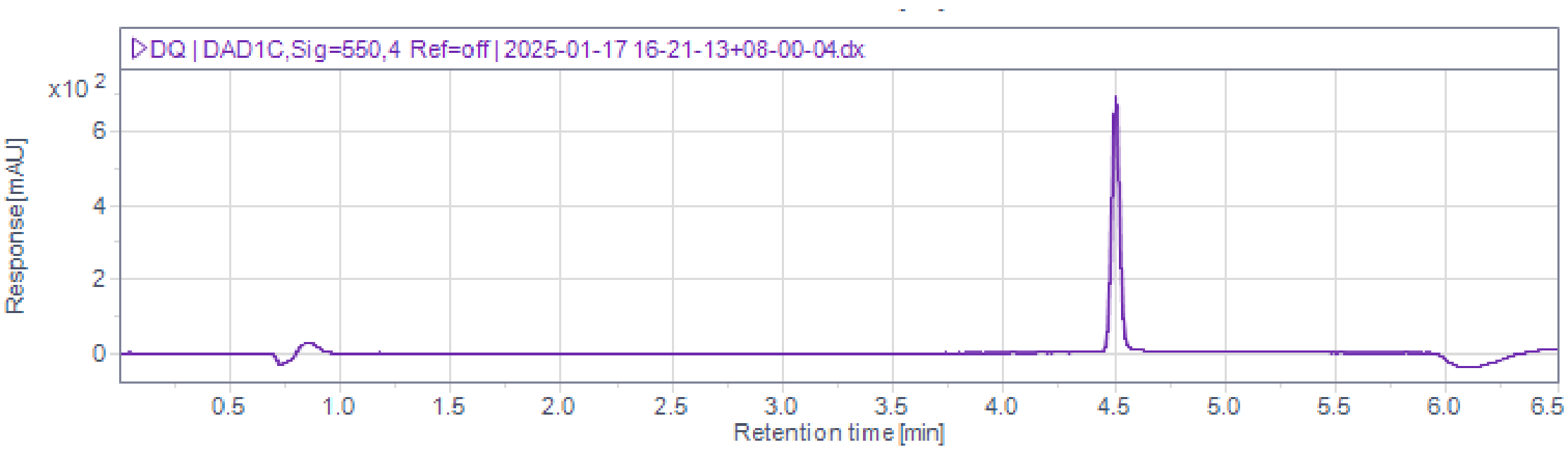

[1]**H NMR** (**UQNR**, 400 MHz, Methanol-d_4) δ 8.67 (d, J = 7.3 Hz, 1H), 8.25 (d, J = 7.9 Hz, 1H), 7.79 – 7.74 (m, 1H), 7.69 – 7.64 (m, 1H), 7.56 (d, J = 9.1 Hz, 1H), 6.74 (d, J = 9.1 Hz, 1H), 6.41 (s, 1H), 3.48 – 3.42 (m, 2H), 3.11 (s, 3H), 2.93 – 2.89 (m, 2H), 2.05 (dt, J = 11.8, 6.3 Hz, 2H).
[13]**C NMR** (**UQNR**, 101 MHz, Methanol-d_4) δ 183.53, 152.63, 150.37, 137.50, 132.07, 131.52, 130.87, 129.86, 129.61, 129.46, 126.43, 125.27, 123.69, 109.92, 106.89, 104.27, 50.99, 39.19, 20.42, 19.63.
MS (**UQNR,** ESI) calculated for $C_{20}H_{17}N_2O_2$ (MH^+) 317.1, observed 317.1.

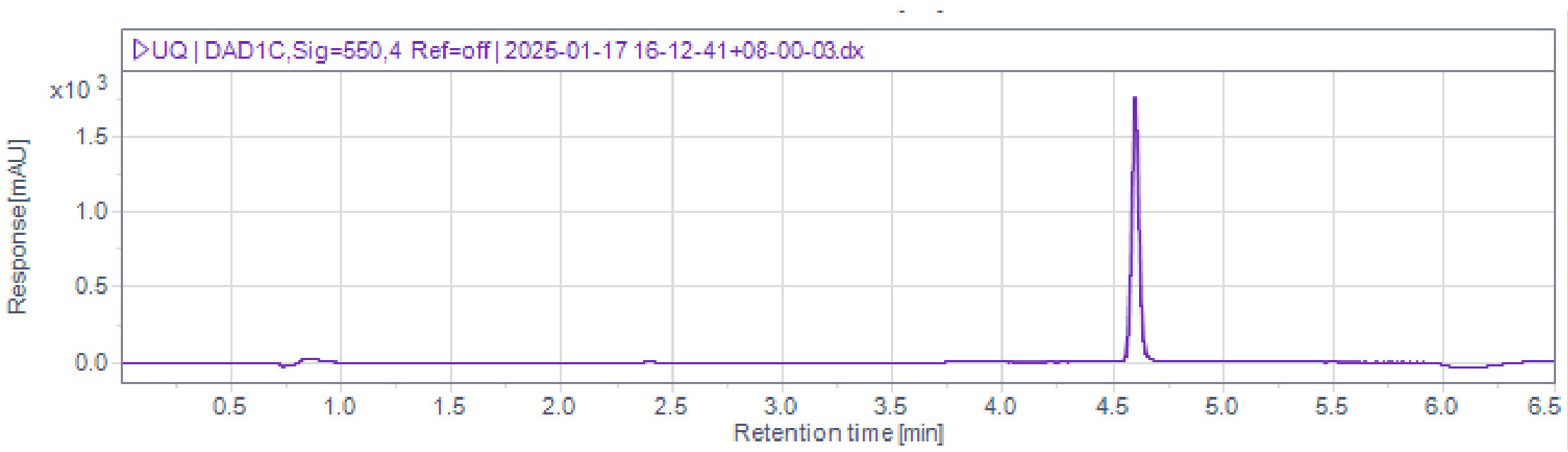

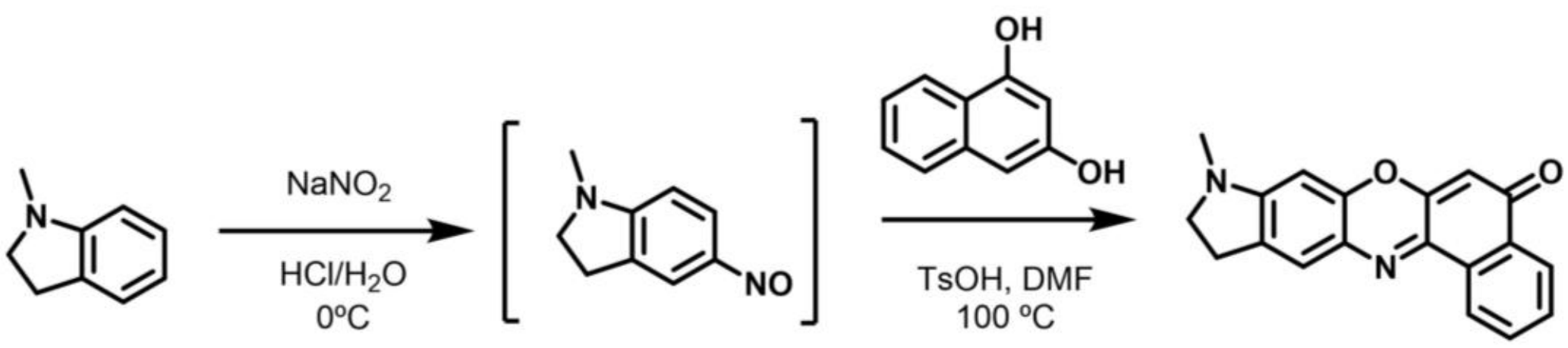

4 **IDNR**

IDNR: 1-methylindoline (**3**, 1.00 g, 7.52 mmol) was dissolved in a mixture of concentrated HCl (3.00 mL, 34.5 mmol) and H_2O (2.40 mL), and the reaction mixture was cooled to 0 °C. A solution of $NaNO_2$ (623 mg, 9.02 mmol, 1.2 eq) in H_2O (4 mL) was added dropwise over 5 min, and the reaction mixture was stirred for another 8 min at 0 °C (ice bath). The reaction mixture was diluted with ice water, and a saturated solution of $NaHCO_3$ was slowly added to give a neutral solution. The product was extracted with DCM (3 × 10 mL), and the combined organic extracts were concentrated *in vacuo*. To the crude intermediate, a solution of 1,6-naphthalenediol (1.32 g, 8.27 mmol, 1.1 eq) and *p*-TsOH (129 mg, 0.752 mmol, 0.1 eq) in anhydrous DMF (10 mL) was added and the resulting reaction mixture was stirred at 100 °C for 10 h. After cooling the reaction mixture to room temperature, the mixture was extracted with EtOAc (2 × 40 mL). The combined organic layer was washed with H_2O (40 mL), and then dried over anhydrous Na_2SO_4, and filtered. After concentration of the filtrate under reduced pressure, the crude product was purified by silica gel column

chromatography (0-10% MeOH in DCM) to afford **IDNR** (1.63 g, 5.41 mmol, 72% yield). **IDNR** was characterized by NMR using mixed solvents of $CDCl_3$ and CD_3OD (2:1).

^{1}H NMR (400 MHz, Methanol-*d*$_4$) δ 8.66 (d, *J* = 7.7 Hz, 1H), 8.26 (d, *J* = 8.0 Hz, 1H), 7.74 (t, *J* = 6.9 Hz, 1H), 7.65 (t, *J* = 8.1 Hz, 1H), 7.37 (s, 1H), 6.38 (s, 1H), 6.22 (s, 1H), 3.69 (t, *J* = 8.1 Hz, 2H), 3.12 (d, *J* = 7.7 Hz, 2H), 2.95 (s, 3H).

MS (ESI) calculated for $C_{19}H_{15}N_2O_2$ (MH^+) 303.1, observed 303.1.

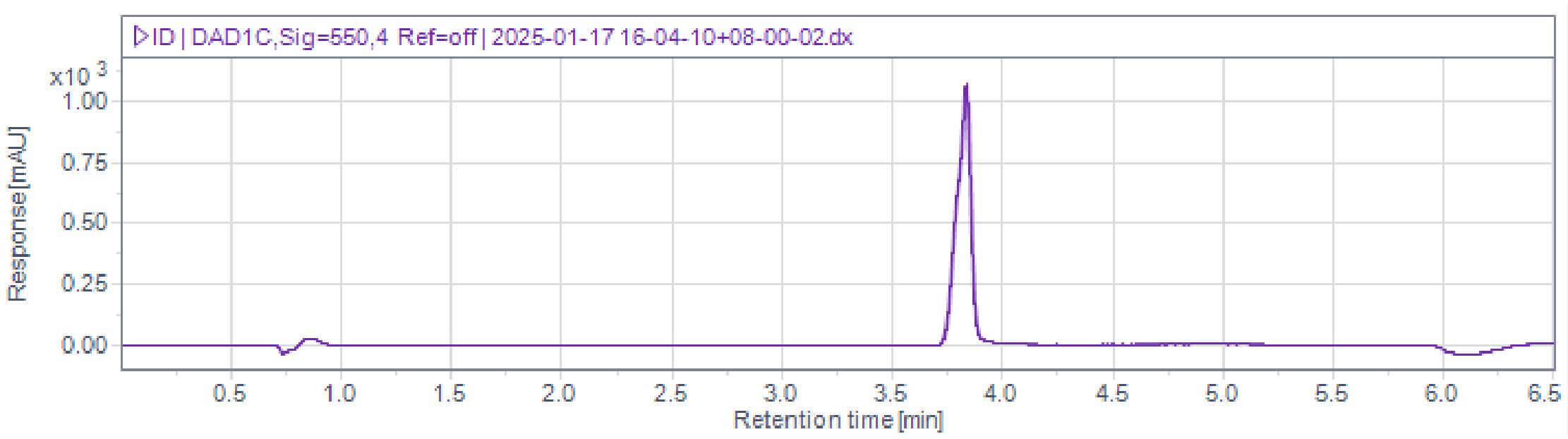

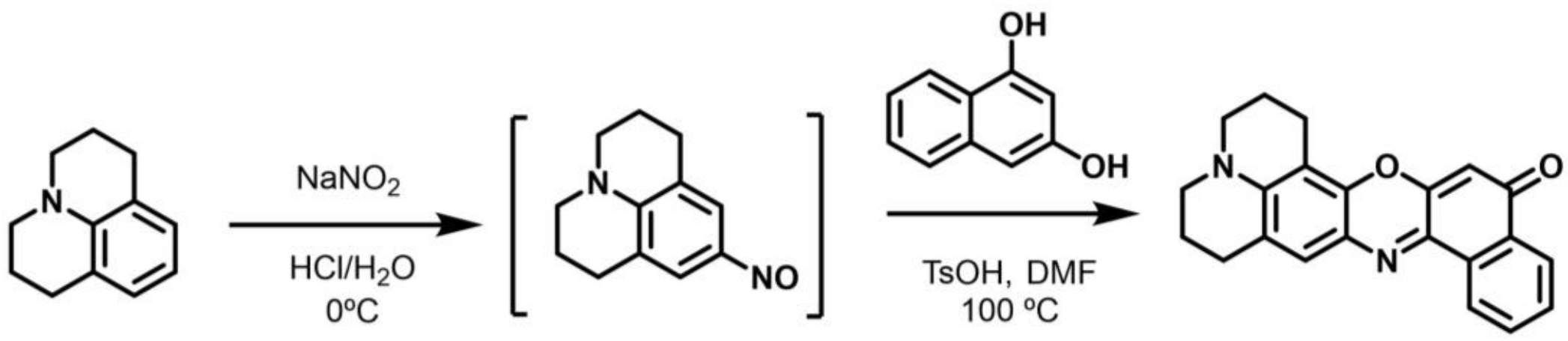

4 **JNR**

JNR: Julolidine (**4**, 1.00 g, 5.78 mmol) was dissolved in a mixture of concentrated HCl (3.00 mL, 34.5 mmol) and H_2O (2.40 mL), and the reaction mixture was cooled to 0 °C. A solution of $NaNO_2$ (479 mg, 6.94 mmol, 1.2 eq) in H_2O (4 mL) was added dropwise over 5 min, and the reaction mixture was stirred for another 6 min at 0 °C (ice bath). The reaction mixture was diluted with ice water, and a saturated solution of $NaHCO_3$ was slowly added to give a neutral solution. The product was extracted with DCM (3 × 10 mL), and the combined organic extracts were concentrated *in vacuo*. A solution of 1,6-naphthalenediol (1.00 g, 6.36 mmol, 1.1 eq) and *p*-TsOH (99 mg, 0.578 mmol, 0.1 eq) in anhydrous DMF (10 mL) was added to the crude intermediate and the resulting reaction mixture was stirred at 100 °C for 10 h. After cooling to room temperature, the reaction mixture was extracted with EtOAc (2 × 40 mL). The combined organic layer was washed with H_2O (40 mL), and then dried over anhydrous Na_2SO_4, and filtered. After concentration of the filtrate under reduced pressure, the crude product was purified by silica gel column chromatography (0-10% MeOH in DCM) to afford **JNR** (1.58 g, 4.62 mmol, 80% yield). **JNR** was characterized by NMR using mixed solvents of $CDCl_3$ and CD_3OD (1:3).

^{1}H NMR (400 MHz, Methanol-*d*$_4$) δ 8.62 – 8.57 (m, 1H), 8.24 – 8.20 (m, 1H), 7.75 – 7.69 (m, 1H), 7.63 (td, *J* = 7.6, 1.3 Hz, 1H), 7.14 (s, 1H), 6.33 (s, 1H), 3.35 (d, *J* = 5.9 Hz, 2H), 3.33 – 3.31 (m, 2H), 2.80 (t, *J* = 6.4 Hz, 2H), 2.77 – 2.73 (m, 2H), 2.00 (d, *J* = 6.1 Hz, 2H), 1.97 (d, *J* = 6.3 Hz, 2H).

^{13}C NMR (101 MHz, Methanol-*d*$_4$) δ 183.21, 152.63, 147.35, 142.06, 135.96, 132.08, 131.07, 130.83, 129.02, 127.62, 126.16, 125.05, 123.47, 121.43, 105.13, 103.41, 50.24, 49.75, 27.28, 20.85, 19.90, 19.47.

MS (ESI) calculated for $C_{22}H_{19}N_2O_2$ (MH^+) 343.1, observed 343.1.

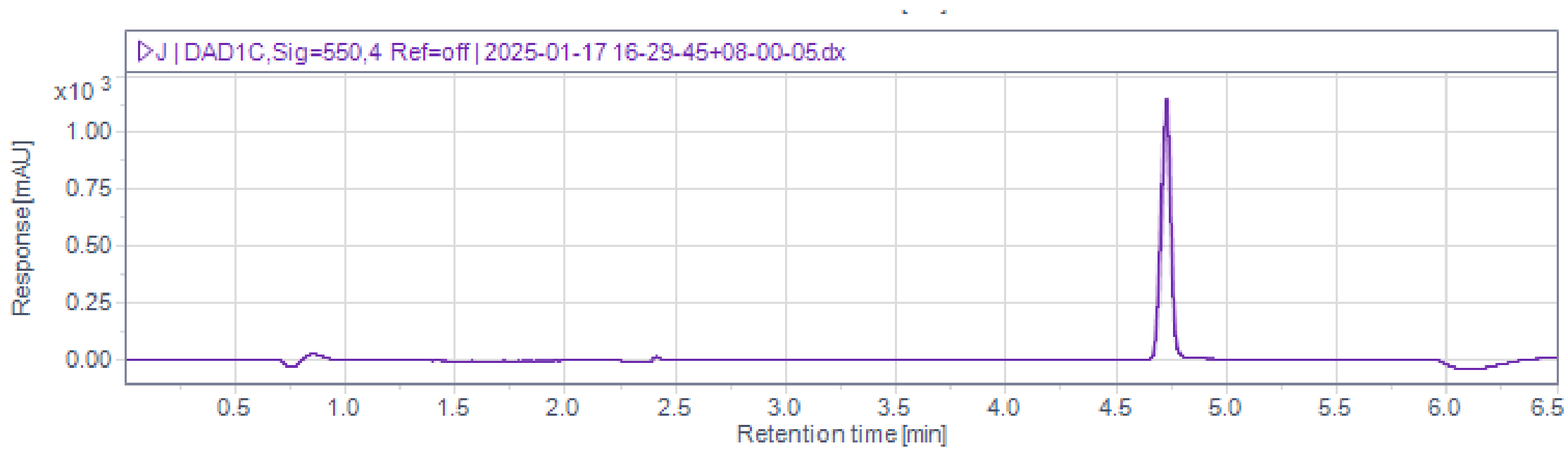

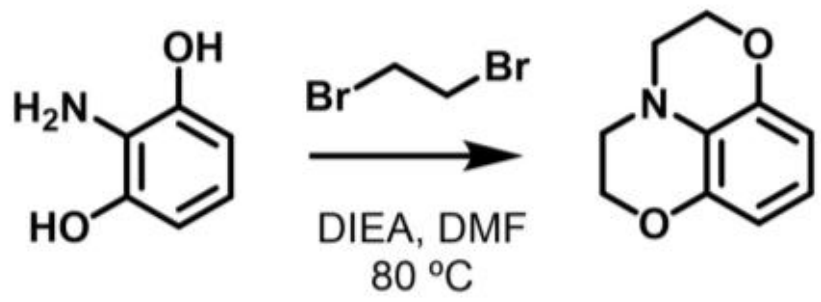

5

Compound **5**: 2-aminobenzene-1,3-diol (1.00 g, 8.00 mmol) was dissolved in a mixture of 1,2-dibromoethane (3.31 g, 17.6 mmol, 2.2 eq), Diisopropylethylamine (DIEA, 4.55 g, 35.2 mmol, 4.4 eq) and DMF (4 mL). The reaction mixture was stirred at 80 °C for 10 h. After cooling to room temperature, the reaction mixture was extracted with EtOAc (3 × 40 mL). The combined organic layer was washed with H_2O (80 mL), and then dried over anhydrous Na_2SO_4, and filtered. After concentration of the filtrate under reduced pressure, the crude product was purified by silica gel column chromatography (10% EtOAc in hexane) to afford compound **5** (1.28 g, 7.22 mmol, 90% yield).

^{1}H NMR (400 MHz, Chloroform-*d*) δ 6.65 – 6.58 (m, 1H), 6.44 (d, J = 8.1 Hz, 2H), 4.44 – 4.38 (m, 4H), 3.20 – 3.14 (m, 4H).

^{13}C NMR (101 MHz, Chloroform-*d*) δ 144.64, 121.96, 118.92, 108.38, 65.29, 46.86.

MS (ESI) calculated for $C_{10}H_{12}NO_2$ (MH^+) 178.1, observed 178.0.

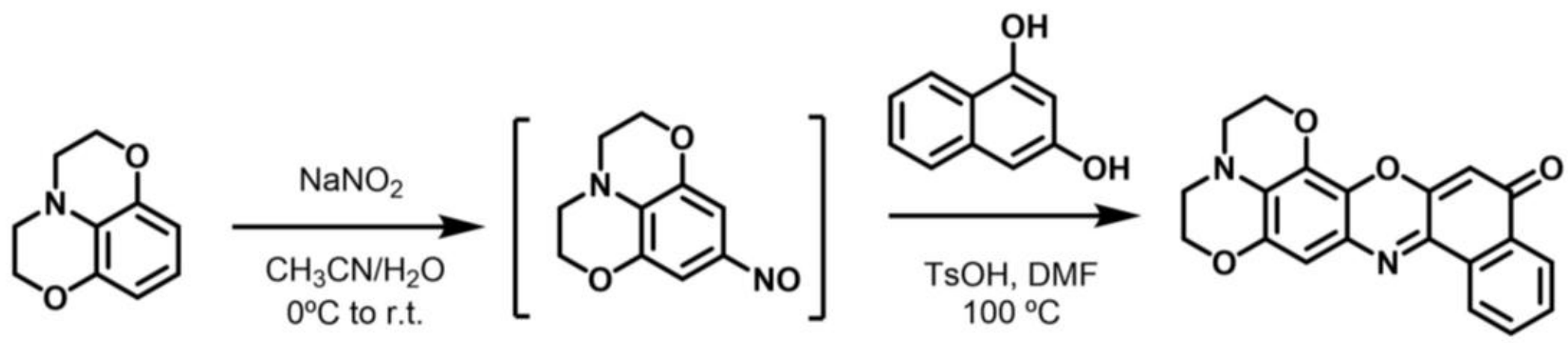

5 **JONR**

JONR: 2,3,4,5-tetrahydro-1,6-dioxa-3a-azaphenalene (**5**, 500 mg, 2.83 mmol) was dissolved in a mixture of CH_3CN (2.00 mL) and concentrated HCl (1.00 mL, 11.5 mmol), and the reaction mixture was cooled to 0 °C. A solution of $NaNO_2$ (234 mg, 3.39 mmol, 1.2 eq) in H_2O (2 mL) was added dropwise over 3 min, and the reaction mixture was stirred for another 7 min at 0 °C (ice bath). The reaction mixture was diluted with ice water, and a saturated solution of $NaHCO_3$ was slowly added to give a neutral solution. The product was extracted with DCM (2 × 20 mL), and the combined organic extracts were concentrated *in vacuo*. A solution of 1,6-naphthalenediol (500 mg, 31.1 mmol,

1.1 eq) and *p*-TsOH (48 mg, 0.28 mmol, 0.1 eq) in anhydrous DMF (10 mL) was added to the crude intermediate. The resulting reaction mixture was stirred at 100 °C for 10 h. After cooling to room temperature, the reaction mixture was extracted with EtOAc (3 × 30 mL). The combined organic layer was washed with H_2O (50 mL), and then dried over anhydrous Na_2SO_4, and filtered. After concentration of the filtrate under reduced pressure, the crude product was purified by silica gel column chromatography (0-5% MeOH in DCM) to afford **JONR** (663 mg, 1.92 mmol, 68% yield). **JONR** was characterized by NMR using mixed solvents of $CDCl_3$ and CD_3OD (1:1).

^{1}H NMR (400 MHz, Methanol-*d*4) δ 8.69 (d, *J* = 7.1 Hz, 1H), 8.26 (d, *J* = 7.2 Hz, 1H), 7.79 – 7.74 (m, 1H), 7.71 – 7.67 (m, 1H), 6.95 (s, 1H), 6.51 (s, 1H), 4.52 – 4.48 (m, 2H), 4.41 – 4.37 (m, 2H), 3.48 – 3.45 (m, 2H), 3.44 – 3.41 (m, 2H).

^{13}C NMR (101 MHz, Methanol-*d*4) δ 183.58, 151.76, 141.14, 140.93, 131.67, 131.25, 130.73, 130.41, 129.34, 129.02, 127.90, 126.33, 125.40, 124.10, 107.87, 104.96, 64.77, 64.31, 46.19, 29.58.

MS (ESI) calculated for $C_{20}H_{15}N_2O_4$ (MH^+) 347.1, observed 347.1.

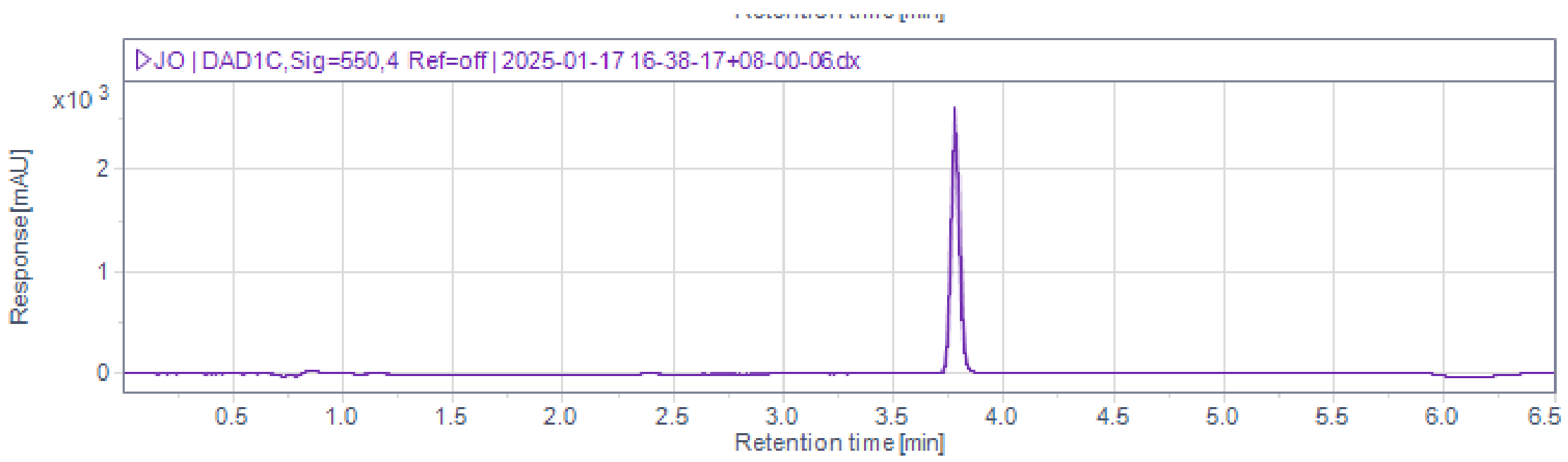

Microscopy Setup and Spectral Ratio Imaging

The SPOT system setup

Our Spectrum and Polarization Optical Tomography (SPOT) system is built upon a commercial inverted fluorescence microscope (Ti2-E, Nikon), equipped with a TIRF objective (100×, 1.49 NA, CFI SR HP Apochromat TIRF 100XC Oil, Nikon). The system employs four lasers (EC30841: MDL-III-405-200mW EF60004, MDL-III-488-180mW EF60009, MGL-FN-561-200mW DE52479, MDL-III-640-200mW EF60007), which are combined using a dichroic mirror set to generate a multicolor excitation source. Laser wavelength selection and intensity modulation are precisely controlled via an acousto-optic tunable filter (AOTF, AA Opto-Electronic, France). To ensure high beam quality, the laser beams are passed through a filtering system composed of focusing lens, collimating lens, and polarization-maintaining single-mode optical fiber (PMC405-PC-2, LBTEK). The collimated beams are then directed into a pure-phase grating module, consisting of polarizing beam splitter (PBS), half-wave plate (AVR1-VIS), and ferroelectric liquid crystal spatial light modulator (FC-SLM, QXGA-3DM, Fourth Dimension Display).

Following phase modulation, the beams are focused onto the pupil plane by an achromatic Fourier lens (AC254-300-A-ML, Thorlabs). To suppress unwanted zeroth-order light and stray diffraction artifacts caused by gaps in the SLM, we implemented a passive spatial filter (Mask) that selectively transmits only the desired ±1st-order diffraction beams. Furthermore, to maximize the contrast of the interference pattern at the sample plane, we designed a customized segmented half-wave plate (Pizza-HWP) that ensures all ±1st-order diffracted beams are modulated into s-polarization. The modulated diffraction beams are relayed to the objective's back focal plane via a relay lens pair, while a polarization-maintaining dichroic mirror (ZT405-415/488/561/640-phase fR-UF3) ensures a high polarization ratio at the objective's back focal plane. Upon passing through the objective, the diffracted beams interfere at the sample plane, generating structured illumination. The fluorescence emitted by the excited sample is transmitted through the dichroic mirror and emission filter (ZET405/488/561/640mv2) and then enters the multispectral imaging splitter (Multisplit V2, Cairn). This splitter spatially separates fluorescence signals of different wavelengths, directing them to distinct regions of the same camera sensor, thereby enabling simultaneous multicolor imaging. A high-quantum-efficiency sCMOS camera (Flash 4.0 V3, Hamamatsu, Japan) is used to capture the fluorescence signals with high sensitivity.

The LCSM and FLIM setup

Fluorescence lifetime imaging microscopy (FLIM) based on laser scanning confocal microscopy (LCSM) was performed using a gated STED microscope (Leica TCS SP8 STED 3X, Leica Microsystems), equipped with a 100×/1.40 NA oil-immersion objective (HCX PL APO, Leica). A white-light laser source provided 80 MHz pulsed excitation over a wavelength range of 470–670 nm. For fluorescence lifetime imaging, the excitation wavelength was set to 561 nm and the emission was collected across the broader range of 590–705 nm.

SPOT image acquisition and processing workflow

SPOT achieves polarization modulation of fluorescent dipoles by altering the polarization state of the excitation light. At the detection end, an integrated image splitter enables simultaneous

acquisition of emission images in the yellow (580-653 nm) and red (665-702 nm) channels, allowing quantification of spectral shift ranges.

The excitation light at 561 nm is used to illuminate the sample. To acquire raw images required for reconstruction, the spatial light modulator (SLM) sequentially loads three different structured illumination directions. For each direction, two phase-shifted patterns (with a phase difference of π) are applied, resulting in a total of six structured illumination images per acquisition cycle.

The raw dual-channel images obtained on the same camera sensor are separated into individual channels, followed by inter-channel registration and single-channel intensity correction.

To generate high-contrast interference fringes, the polarization direction of the excitation beam must be aligned with the fringe orientation on the sample. In SPOT, the polarization orientation of the structured illumination is varied across 0°, 60°, and 120° to match each fringe direction, thereby ensuring effective polarization modulation of fluorescent dipoles.

Each channel's raw image stack is reconstructed using the HiLo algorithm, which combines the high- and low-spatial frequency components of each pattern direction. This suppresses weakly modulated out-of-focus background signals, thereby enhancing the accuracy of lipid polarity and phase measurements.

By fitting the HiLo-reconstructed images acquired under three polarization directions of structured illumination, the modulation depth of fluorescent dipoles can be extracted to generate the Phase Map. The dipole orientation can also be retrieved through the same fitting process.

To calculate the **modulation depth** and determine the **dipole orientation** β, the emission intensity $I_{HiLo,Pi}$ of a fluorescent dipole under an incident polarization angle θ_i is expressed as:

$$I_{HiLo,Pi} = I_{DC} + I_{AC} \cdot \cos\left(2\theta_i - 2\beta\right), i = 1,2,3 \tag{1-1}$$

In matrix form, the equation can be rewritten as:

$$\begin{bmatrix} I_{HiLo,P1} \\ I_{HiLo,P2} \\ I_{HiLo,P3} \end{bmatrix} = \begin{bmatrix} 1 & \cos 2\theta_1 & \sin 2\theta_1 \\ 1 & \cos 2\theta_2 & \sin 2\theta_2 \\ 1 & \cos 2\theta_3 & \sin 2\theta_3 \end{bmatrix} \begin{bmatrix} I_{DC} \\ I_{AC} \times \cos 2\beta \\ I_{AC} \times \sin 2\beta \end{bmatrix} \tag{1-2}$$

Here, I_{DC} represents the direct current (DC) component, I_{AC} denotes the alternating current (AC) component, θ_i indicates the illumination polarization angle, and β is the orientation angle of the fluorescent dipole.

The values of I_{DC} and I_{AC} can be obtained by solving the inverse of the matrix equation.

The modulation depth is calculated as:

$$ModulationDepth = \frac{2I_{AC}}{I_{AC} + I_{DC}} \tag{1-3}$$

The emission intensities from the yellow and red channels of the HiLo images are averaged and then used to compute the emission ratio, which reflects the emission properties of different lipid compositions. The resulting values are visualized as the **Polarity Map**.

$$Emissionratio = \frac{I_{redchannel}}{I_{yellowchannel}} \tag{1-4}$$

Image registration and light intensity correction

Since spectral ratio imaging involves calculating the ratio between two images acquired at different wavelengths, it is particularly sensitive to chromatic shifts, optical distortions, and misalignments—issues that become more pronounced in super-resolution imaging compared to diffraction-limited multicolor 3D imaging. To ensure accurate quantitative ratio measurements, precise spatial alignment of fine structures at the super-resolution level is essential.

To achieve this, we first acquired calibration reference images using fixed samples to correct for channel misalignment. Specifically, Alexa Fluor™ 555 Phalloidin (A34055, Invitrogen™) and Alexa Fluor™ Plus 647 Phalloidin (A30107, Invitrogen™) were used to label the actin cytoskeleton, enabling the acquisition of images of the same subcellular structures at different emission wavelengths. Subsequently, live-cell imaging of subcellular organelle dynamics was performed using the acquisition approach described above. Finally, Chromagnon software was employed to measure and compute a channel misalignment correction matrix, which was then applied to the captured multichannel live-cell images to generate precisely aligned datasets. The aligned two-channel images were then used to compute ratio images, providing quantitative spectral ratio information.

Computational Data

Table S1. Computed S1 emission wavelength for NR in DMSO using various functionals.

Functional	Computed S1 emission wavelength (nm)
TPSSh	648
PBE0	623
B3LYP	636
cam-B3LYP	623
LC-wPBE	621
wB97xD	628

Cartesian coordinates

JONR_S1_DMSO
C 5.992702 -1.390927 -0.010398
C 5.615557 -0.054381 -0.008606
C 4.265863 0.315793 -0.010196
C 3.268587 -0.698074 -0.003735
C 3.669547 -2.050317 -0.008040
C 5.011348 -2.392292 -0.009573
C 1.862144 -0.352847 -0.015563
C 1.546216 1.046194 0.000271
C 2.490257 2.041824 -0.011303
C -0.730291 0.460154 -0.023618
C -0.382151 -0.902548 -0.012802
H 7.043683 -1.659430 -0.011433
H 6.361801 0.732339 -0.007361
H 2.906195 -2.819006 -0.006540
H 5.301940 -3.437387 -0.010887
H 2.177341 3.080192 -0.006966
N 0.938320 -1.323551 -0.013355
C 3.898903 1.749425 0.003157
O 4.766997 2.652248 0.016946
O 0.228306 1.442843 -0.008196
C -2.061113 0.870116 0.012238
C -1.408308 -1.867486 -0.011482
C -2.734549 -1.487312 0.006583
C -3.093045 -0.106425 -0.013037
H -1.144452 -2.917951 0.000829
N -4.398268 0.276814 -0.100631
C -4.699021 1.680075 -0.340559
H -5.704838 1.889494 0.028161
H -4.668552 1.895014 -1.417344
C -3.693737 2.526021 0.409550
H -3.781449 2.371832 1.488278
H -3.818059 3.580748 0.174524
C -5.411834 -0.743450 -0.323373
H -6.364106 -0.378806 0.066442
H -5.524296 -0.935224 -1.399143
C -5.003785 -2.002083 0.411575
H -5.685022 -2.819725 0.186626
H -4.976977 -1.829459 1.491396
O -2.338976 2.195563 0.008238
O -3.696743 -2.449931 -0.020142

TS_O
C -4.521783 -3.903141 0.170022
C -4.568556 -2.520652 0.086521
C -3.386950 -1.761256 0.049669
C -2.132949 -2.433097 0.086976
C -2.109918 -3.838121 0.175548
C -3.286554 -4.565000 0.214007
C -0.895354 -1.678151 0.061830
C -1.028977 -0.255876 -0.048103
C -2.236549 0.409636 -0.080926
C 1.305649 -0.112537 -0.020440
C 1.407749 -1.494950 0.056963
H -5.443957 -4.473280 0.199225
H -5.522234 -2.008070 0.050765
H -1.151204 -4.340986 0.208051
H -3.252102 -5.646974 0.279071
H -2.248566 1.491480 -0.145582
N 0.277606 -2.305684 0.114434
C -3.440270 -0.310434 -0.049527
O -4.613587 0.264382 -0.089449
O 0.105198 0.519841 -0.077906

C 2.454830 0.705970 -0.116455
C 2.699837 -2.073736 0.088235
C 3.836956 -1.298565 0.028650
C 3.749833 0.111621 -0.049129
H 2.789696 -3.151603 0.158628
N 4.853191 0.904484 -0.028084
C 4.673707 2.338327 0.165463
H 5.556786 2.857317 -0.210434
H 4.561288 2.562110 1.234066
C 3.465029 2.791919 -0.617126
H 3.602764 2.652647 -1.691277
H 3.209935 3.825462 -0.398407
C 6.146312 0.281159 0.216817
H 6.922070 0.900241 -0.238226
H 6.335953 0.216843 1.295943
C 6.149582 -1.100450 -0.408946
H 7.062925 -1.634223 -0.155259
H 6.047998 -1.034010 -1.495685
H -4.589001 1.561175 -0.177329
O -4.697134 2.929847 -0.300386
C -5.024480 3.202205 -1.654054
H -5.120315 4.285979 -1.800231
H -4.256479 2.838387 -2.353984
H -5.981408 2.742496 -1.941283
H -3.385931 3.762914 0.159630
O -2.583380 4.302057 0.448543
C -2.772776 4.670490 1.813848
H -1.914869 5.272480 2.123482
H -3.682962 5.268696 1.950473
H -2.832854 3.792017 2.469626
O 2.280099 2.022042 -0.239553
O 5.067639 -1.903779 0.115655

JONR_S1_DCM

C 5.992423 -1.389576 -0.012090
C 5.614984 -0.052939 -0.009543
C 4.264960 0.315463 -0.010491
C 3.268808 -0.698522 -0.004257
C 3.669497 -2.050451 -0.009415
C 5.011833 -2.391406 -0.011770
C 1.861483 -0.354166 -0.014548
C 1.545298 1.045489 0.001821
C 2.487659 2.041597 -0.009774
C -0.730324 0.458764 -0.020493
C -0.381852 -0.904333 -0.009815
H 7.043601 -1.657353 -0.013590
H 6.359661 0.735301 -0.007968
H 2.904505 -2.817383 -0.008210
H 5.303138 -3.436337 -0.014007
H 2.174593 3.079698 -0.005793
N 0.939596 -1.325687 -0.011197
C 3.896707 1.748298 0.002621
O 4.763919 2.651892 0.014132
O 0.226384 1.442512 -0.005746
C -2.061042 0.868354 0.015346
C -1.407527 -1.868307 -0.009770
C -2.733916 -1.485558 0.009948
C -3.091758 -0.106025 -0.006404
H -1.145466 -2.919080 0.000704
N -4.401865 0.278786 -0.083519
C -4.695443 1.680204 -0.342801
H -5.702946 1.899088 0.016446
H -4.655454 1.885253 -1.421471
C -3.689222 2.531704 0.402828
H -3.780980 2.388210 1.482972
H -3.810623 3.584558 0.157963
C -5.408350 -0.743224 -0.330553
H -6.368903 -0.384862 0.045126
H -5.501772 -0.932443 -1.408872
C -5.006754 -2.006141 0.402772
H -5.683700 -2.824283 0.166060
H -4.992184 -1.839865 1.483924
O -2.337309 2.196327 0.007537
O -3.697040 -2.449174 -0.017415

NR_S1_DMSO

C 6.019732 -1.389292 0.051107
C 5.638558 -0.053607 0.032815
C 4.288298 0.312942 0.007216
C 3.293054 -0.704287 0.009845
C 3.698579 -2.055622 0.025685
C 5.041295 -2.393524 0.047944
C 1.888035 -0.363595 -0.028381
C 1.566175 1.031414 -0.027850
C 2.506432 2.031579 -0.037744
C -0.723176 0.446456 -0.065924
C -0.351559 -0.914836 -0.051345
H 7.071402 -1.654443 0.068925
H 6.382863 0.735009 0.036731
H 2.937550 -2.826615 0.023615

H 5.334836 -3.437701 0.062436
H 2.189697 3.068784 -0.045431
N 0.962015 -1.337772 -0.037579
C 3.916677 1.744800 0.000280
O 4.780226 2.652080 0.019805
O 0.245868 1.424036 -0.059943
C -2.041231 0.878160 -0.055265
C -1.392089 -1.863637 -0.065475
C -2.724286 -1.503636 -0.081371
C -3.068647 -0.112125 -0.079467
H -1.118765 -2.913922 -0.058785
N -4.382341 0.270349 -0.087668
C -4.756057 1.674412 -0.254389
H -5.772102 1.784598 0.125983
H -4.769932 1.915927 -1.327461
C -3.785148 2.585945 0.477966
H -3.841251 2.383891 1.551804
H -4.080361 3.624702 0.315954
C -5.470046 -0.707382 -0.155818
H -6.337267 -0.252700 0.326496
H -5.728469 -0.877329 -1.211706
C -5.088000 -2.019569 0.508527
H -5.908130 -2.729456 0.381908
H -4.944314 -1.858609 1.581491
C -3.806399 -2.549268 -0.123806
H -4.007477 -2.821368 -1.168235
H -3.458762 -3.454590 0.379178
C -2.368029 2.348758 -0.032117
H -1.640651 2.877314 0.586980
H -2.268356 2.755030 -1.046645

NR_S1_MeOH

C 4.397519 -4.068234 -0.095980
C 4.481884 -2.682593 -0.090856
C 3.326731 -1.888400 -0.080131
C 2.051586 -2.521680 -0.085020
C 1.987674 -3.929334 -0.086141
C 3.143309 -4.692951 -0.094474
C 0.837613 -1.733914 -0.058745
C 0.999492 -0.312615 -0.076095
C 2.221140 0.318728 -0.067422
C -1.352364 -0.099851 -0.045393
C -1.456624 -1.504095 -0.041615
H 5.302828 -4.665608 -0.102040
H 5.449341 -2.193533 -0.092844
H 1.014762 -4.405428 -0.084060
H 3.075179 -5.775434 -0.098314
H 2.262664 1.402279 -0.073440
N -0.357939 -2.340560 -0.045977
C 3.435581 -0.425222 -0.087754
O 4.576779 0.145002 -0.102884
O -0.111868 0.497943 -0.070545
C -2.447484 0.750617 -0.022504
C -2.755562 -2.050337 -0.024940
C -3.890357 -1.266177 -0.022611
C -3.748774 0.160592 -0.021354
H -2.849362 -3.131555 -0.019588
N -4.855422 0.962764 -0.015753
C -4.741796 2.414851 -0.157977
H -5.649865 2.851118 0.259882
H -4.711549 2.666702 -1.228454
C -3.500809 2.939909 0.543000
H -3.589140 2.760348 1.618739
H -3.434890 4.018663 0.386916
C -6.206584 0.403779 -0.097683
H -6.879716 1.123594 0.371123
H -6.492398 0.323455 -1.157029
C -6.292723 -0.956059 0.574503
H -7.303230 -1.349141 0.443389
H -6.111447 -0.844831 1.648120
H 4.771357 1.819283 0.008498
O 4.975371 2.789645 0.112361
C 5.688773 2.962713 1.343907
H 5.919813 4.024454 1.444491
H 5.087656 2.646776 2.203849
H 6.625591 2.395497 1.335354
H 3.538485 3.769594 0.030940
O 2.732284 4.342111 -0.011233
C 2.816535 5.132104 -1.200574
H 1.928231 5.765952 -1.238365
H 3.704929 5.775305 -1.195844
H 2.838890 4.507176 -2.101588
C -5.260680 -1.891862 -0.044312
H -5.544365 -2.103281 -1.083578
H -5.231423 -2.851356 0.477612
C -2.261412 2.244278 -0.008476
H -1.378491 2.500612 0.580301
H -2.066841 2.590831 -1.031684

JONR_S1_MeOH

C 4.216395 -4.219900 -0.160662
C 4.348494 -2.839378 -0.114680
C 3.220933 -2.006824 -0.084602
C 1.925971 -2.595534 -0.110885
C 1.812943 -3.999209 -0.153164
C 2.941320 -4.801326 -0.181002
C 0.738398 -1.766257 -0.063477
C 0.955579 -0.348907 -0.038239
C 2.198260 0.237898 -0.010665
C -1.376599 -0.071725 -0.016574
C -1.550795 -1.459003 -0.036574
H 5.100524 -4.847970 -0.181408
H 5.331718 -2.383270 -0.099083
H 0.824057 -4.440941 -0.166647
H 2.836415 -5.880265 -0.216710
H 2.277552 1.318928 0.011252
N -0.471742 -2.331891 -0.065038
C 3.382931 -0.547993 -0.049142
O 4.546720 -0.023786 -0.047451
O -0.129333 0.498100 -0.015571
C -2.465919 0.800649 0.045532
C -2.864295 -1.979145 -0.013592
C -3.955724 -1.141261 0.029215
C -3.787083 0.272211 0.034981
H -3.007356 -3.053047 -0.024178
N -4.855483 1.112763 -0.017271
C -4.617047 2.531429 -0.248644
H -5.467709 3.096644 0.135891
H -4.523739 2.725386 -1.325429
C -3.362617 2.944254 0.486512
H -3.478538 2.829258 1.566776
H -3.083104 3.966951 0.245581
C -6.179748 0.542991 -0.223721
H -6.921449 1.226197 0.194646
H -6.375254 0.428247 -1.298166
C -6.252163 -0.794691 0.481960
H -7.191894 -1.296806 0.262335
H -6.139039 -0.672771 1.562798
H 4.819479 1.630694 0.084268
O 5.063588 2.590759 0.202928
C 5.376066 2.798637 1.586078
H 5.647296 3.848361 1.709509
H 4.517666 2.575396 2.229582
H 6.223361 2.176182 1.893067
H 3.778875 3.624867 -0.348559
O 3.052363 4.228039 -0.645248
C 3.639440 5.486256 -0.986648
H 2.837312 6.141401 -1.332737
H 4.124640 5.955792 -0.122031
H 4.377774 5.382891 -1.791131
O -2.230326 2.129215 0.072045
O -5.213819 -1.681985 0.002609

JONR_PT_N

C 5.865925 -0.070882 -0.454278
C 5.314714 -1.312573 -0.200507
C 3.923744 -1.490474 -0.120442
C 3.066107 -0.365904 -0.295946
C 3.651514 0.894070 -0.559242
C 5.023169 1.037237 -0.633615
C 1.643911 -0.551725 -0.223781
C 1.151485 -1.847484 0.033991
C 1.967201 -2.945567 0.202905
C -1.075050 -1.028773 -0.076362
C -0.610990 0.273569 -0.298256
H 6.942180 0.048462 -0.514674
H 5.950157 -2.179582 -0.060215
H 3.026132 1.767203 -0.700183
H 5.448229 2.014853 -0.834588
H 1.518409 -3.912210 0.399454
N 0.753725 0.478976 -0.367950
C 3.380182 -2.829736 0.157857
O 4.147470 -3.826658 0.339215
O -0.206031 -2.082648 0.112175
C -2.433803 -1.296489 0.018107
C -1.531590 1.310447 -0.483863
C -2.886629 1.044378 -0.398592
C -3.371824 -0.261351 -0.158095
H -1.188641 2.320965 -0.664905
N -4.729320 -0.522749 -0.084806
C -5.101732 -1.776491 0.559573
H -6.139340 -2.004385 0.307012
H -5.013299 -1.707802 1.652139
C -4.203907 -2.874917 0.030043
H -4.329139 -2.993989 -1.049482
H -4.401195 -3.820955 0.530758
C -5.590511 0.636302 0.116139
H -6.615098 0.357974 -0.139056
H -5.568710 0.976529 1.160250

C -5.124958 1.743475 -0.805497
H -5.700506 2.653883 -0.648018
H -5.201065 1.435241 -1.852087
H 1.071550 1.449529 -0.508335
O 1.167560 3.294514 -0.532723
C 1.809405 4.198132 -1.441810
H 1.358110 5.194205 -1.384485
H 2.882548 4.278697 -1.238504
H 1.666359 3.802410 -2.448175
H 1.304094 3.610427 0.389903
O 1.548510 3.954900 2.085184
C 1.220807 2.908374 2.901083
H 0.303542 2.400359 2.578621
H 2.033493 2.169025 2.755586
H 1.202866 3.177367 3.961781
O -3.751652 2.104669 -0.525472
O -2.811424 -2.581465 0.304124

NR_S1_DCM

C 6.020316 -1.387389 0.046999
C 5.638667 -0.051682 0.029533
C 4.287964 0.312916 0.006512
C 3.294384 -0.704701 0.010284
C 3.699683 -2.055607 0.025359
C 5.042983 -2.392384 0.045250
C 1.887935 -0.365088 -0.025880
C 1.565966 1.031192 -0.024265
C 2.504763 2.031633 -0.034755
C -0.722384 0.444704 -0.062558
C -0.351161 -0.915336 -0.048342
H 7.072276 -1.651597 0.062927
H 6.381247 0.738579 0.032297
H 2.937051 -2.824907 0.024175
H 5.337534 -3.436314 0.059026
H 2.188308 3.068739 -0.042017
N 0.964643 -1.339055 -0.034488
C 3.914963 1.744178 0.000053
O 4.778427 2.651801 0.016845
O 0.244362 1.423202 -0.054352
C -2.041879 0.876401 -0.053638
C -1.391004 -1.863560 -0.063306
C -2.724209 -1.503285 -0.080046
C -3.067642 -0.113099 -0.078167
H -1.116306 -2.913393 -0.056730
N -4.384452 0.271274 -0.085217
C -4.754958 1.674676 -0.259578
H -5.772543 1.788686 0.116537
H -4.765276 1.914393 -1.333483
C -3.785414 2.588491 0.473436
H -3.845544 2.389858 1.547666
H -4.079100 3.627240 0.307807
C -5.470425 -0.706674 -0.160460
H -6.341205 -0.253722 0.317878
H -5.725316 -0.877944 -1.217401
C -5.090778 -2.019578 0.505600
H -5.910703 -2.729613 0.377252
H -4.950343 -1.858333 1.578894
C -3.806559 -2.549918 -0.122303
H -4.004688 -2.827323 -1.165968
H -3.460711 -3.453358 0.385376
C -2.366127 2.348059 -0.030528
H -1.639740 2.873821 0.592263
H -2.259086 2.755934 -1.043719

JONR_PT_O

C 4.449439 -4.026122 -0.143400
C 4.525664 -2.650462 -0.105317
C 3.352335 -1.862459 -0.066180
C 2.079503 -2.505606 -0.076352
C 2.034644 -3.916471 -0.112375
C 3.193512 -4.663004 -0.147194
C 0.864326 -1.724721 -0.026214
C 1.014324 -0.330545 -0.006372
C 2.251674 0.316619 0.005698
C -1.328240 -0.121741 0.052381
C -1.437043 -1.530529 -0.002846
H 5.357245 -4.619244 -0.171205
H 5.489778 -2.156496 -0.103126
H 1.066301 -4.401784 -0.115202
H 3.138026 -5.746122 -0.176876
H 2.283463 1.398902 0.013473
N -0.343580 -2.343483 -0.032496
C 3.413725 -0.428804 -0.040068
O 4.642647 0.133966 -0.049565
O -0.091268 0.488564 0.037693
C -2.442200 0.703832 0.064865
C -2.734061 -2.083092 0.016897
C -3.847615 -1.268693 0.036567
C -3.733663 0.142941 0.073449
H -2.853802 -3.159408 -0.015604

N -4.855202 0.954056 0.106813
C -4.644273 2.350712 -0.252839
H -5.503196 2.932837 0.087611
H -4.544278 2.475164 -1.339593
C -3.393625 2.842602 0.445837
H -3.504417 2.776983 1.531778
H -3.160867 3.867031 0.160827
C -6.110182 0.324862 -0.284834
H -6.936674 0.950442 0.058695
H -6.182179 0.215966 -1.375526
C -6.192744 -1.030596 0.383727
H -7.088226 -1.567408 0.075249
H -6.182705 -0.926335 1.472623
H 4.595434 1.138982 -0.016285
O 4.765325 2.762861 0.036883
C 5.223842 3.272372 1.300620
H 5.462295 4.337122 1.221554
H 4.474430 3.123975 2.084631
H 6.128684 2.722521 1.560865
H 3.945617 3.248831 -0.219148
O 2.517213 4.144660 -0.662141
C 2.769112 5.481718 -0.801309
H 2.012292 5.992881 -1.406035
H 2.681314 5.890316 0.225159
H 3.791541 5.694260 -1.134099
O -2.240459 2.062726 0.048372
O -5.084623 -1.870314 -0.014795

TS_N

C -5.904947 -0.012805 -0.327706
C -5.388353 -1.292766 -0.198227
C -4.008352 -1.509680 -0.108959
C -3.124163 -0.393433 -0.148896
C -3.671005 0.902838 -0.283876
C -5.038065 1.087041 -0.377881
C -1.703766 -0.609366 -0.054688
C -1.245334 -1.958878 -0.006059
C -2.082042 -3.045815 0.030469
C 0.979368 -1.175910 -0.013992
C 0.550437 0.141101 -0.056412
H -6.977051 0.136585 -0.395113
H -6.047577 -2.152705 -0.169568
H -3.015256 1.764464 -0.322748
H -5.436965 2.089327 -0.488669
H -1.660257 -4.042626 0.086362
N -0.819834 0.412412 -0.046620
C -3.496205 -2.890029 -0.015776
O -4.283583 -3.879296 0.016654
O 0.108341 -2.223570 0.048392
C 2.351032 -1.484072 0.031898
C 1.499415 1.180741 -0.122582
C 2.849459 0.895472 -0.091862
C 3.312659 -0.439031 -0.039474
H 1.168008 2.209103 -0.191743
N 4.642034 -0.743820 -0.097257
C 5.012419 -2.128697 -0.360745
H 6.041821 -2.284798 -0.033726
H 4.947792 -2.345346 -1.435082
C 4.098955 -3.035655 0.429917
H 4.213768 -2.881963 1.505116
H 4.258349 -4.081511 0.179388
C 5.574742 0.332508 -0.401210
H 6.555660 0.066555 -0.001779
H 5.662499 0.467825 -1.487208
C 5.078832 1.606507 0.253703
H 5.699435 2.453420 -0.031290
H 5.066978 1.505307 1.342386
O 2.700316 -2.777264 0.102658
O 3.744269 1.931700 -0.201020
H -1.078808 1.489727 0.233704
O -1.143516 2.804749 0.689723
C -1.690542 2.917582 1.975771
H -1.245696 3.757029 2.528046
H -2.779513 3.096425 1.931674
H -1.543247 1.997973 2.562140
H -1.276291 4.198391 -0.203925
O -1.376181 5.034452 -0.743466
C -0.216327 5.838144 -0.525389
H -0.111942 6.124532 0.528886
H 0.700628 5.323866 -0.840037
H -0.324204 6.747330 -1.121353

Reference

1. Frisch, M. J.; Trucks, G. W.; Schlegel, H. B.; Scuseria, G. E.; Robb, M. A.; Cheeseman, J. R.; Scalmani, G.; Barone, V.; Petersson, G. A.; Nakatsuji, H.; Li, X.; Caricato, M.; Marenich, A. V.; Bloino, J.; Janesko, B. G.; Gomperts, R.; Mennucci, B.; Hratchian, H. P.; Ortiz, J. V.; Izmaylov, A. F.; Sonnenberg, J. L.; Williams; Ding, F.; Lipparini, F.; Egidi, F.; Goings, J.; Peng, B.; Petrone, A.; Henderson, T.; Ranasinghe, D.; Zakrzewski, V. G.; Gao, J.; Rega, N.; Zheng, G.; Liang, W.; Hada, M.; Ehara, M.; Toyota, K.; Fukuda, R.; Hasegawa, J.; Ishida, M.; Nakajima, T.; Honda, Y.; Kitao, O.; Nakai, H.; Vreven, T.; Throssell, K.; Montgomery Jr., J. A.; Peralta, J. E.; Ogliaro, F.; Bearpark, M. J.; Heyd, J. J.; Brothers, E. N.; Kudin, K. N.; Staroverov, V. N.; Keith, T. A.; Kobayashi, R.; Normand, J.; Raghavachari, K.; Rendell, A. P.; Burant, J. C.; Iyengar, S. S.; Tomasi, J.; Cossi, M.; Millam, J. M.; Klene, M.; Adamo, C.; Cammi, R.; Ochterski, J. W.; Martin, R. L.; Morokuma, K.; Farkas, O.; Foresman, J. B.; Fox, D. J. Gaussian 16 Rev. C.01, Wallingford, CT, 2016.
2. J. M. Tao, J. P. Perdew, V. N. Staroverov, and G. E. Scuseria, “Climbing the density functional ladder: Nonempirical meta-generalized gradient approximation designed for molecules and solids,” *Phys. Rev. Lett.*, 2003, 91, 146401.
3. A. Schaefer, C. Huber, and R. Ahlrichs, “Fully optimized contracted Gaussian-basis sets of triple zeta valence quality for atoms Li to Kr,” *J. Chem. Phys.*, 1994, 100, 5829-35.
4. Marenich, A. V.; Cramer, C. J.; Truhlar, D. G., Universal solvation model based on solute electron density and on a continuum model of the solvent defined by the bulk dielectric constant and atomic surface tensions. *J. Phys. Chem. B,* 2009, 113 (18), 6378-6396.
5. Legault, C., CYLview, 1.0 b, Université de Sherbrooke. Sherbrooke, QC: http://www. cylview. org 2009.
6. Humphrey, W., Dalke, A. and Schulten, K., "VMD - Visual Molecular Dynamics", *J. Molec. Graphics*, 1996, 14, 33-38.
7. Tian Lu, Feiwu Chen, Multiwfn: A Multifunctional Wavefunction Analyzer, *J. Comput. Chem*. 2012, 33, 580-592.

NMR Spectra

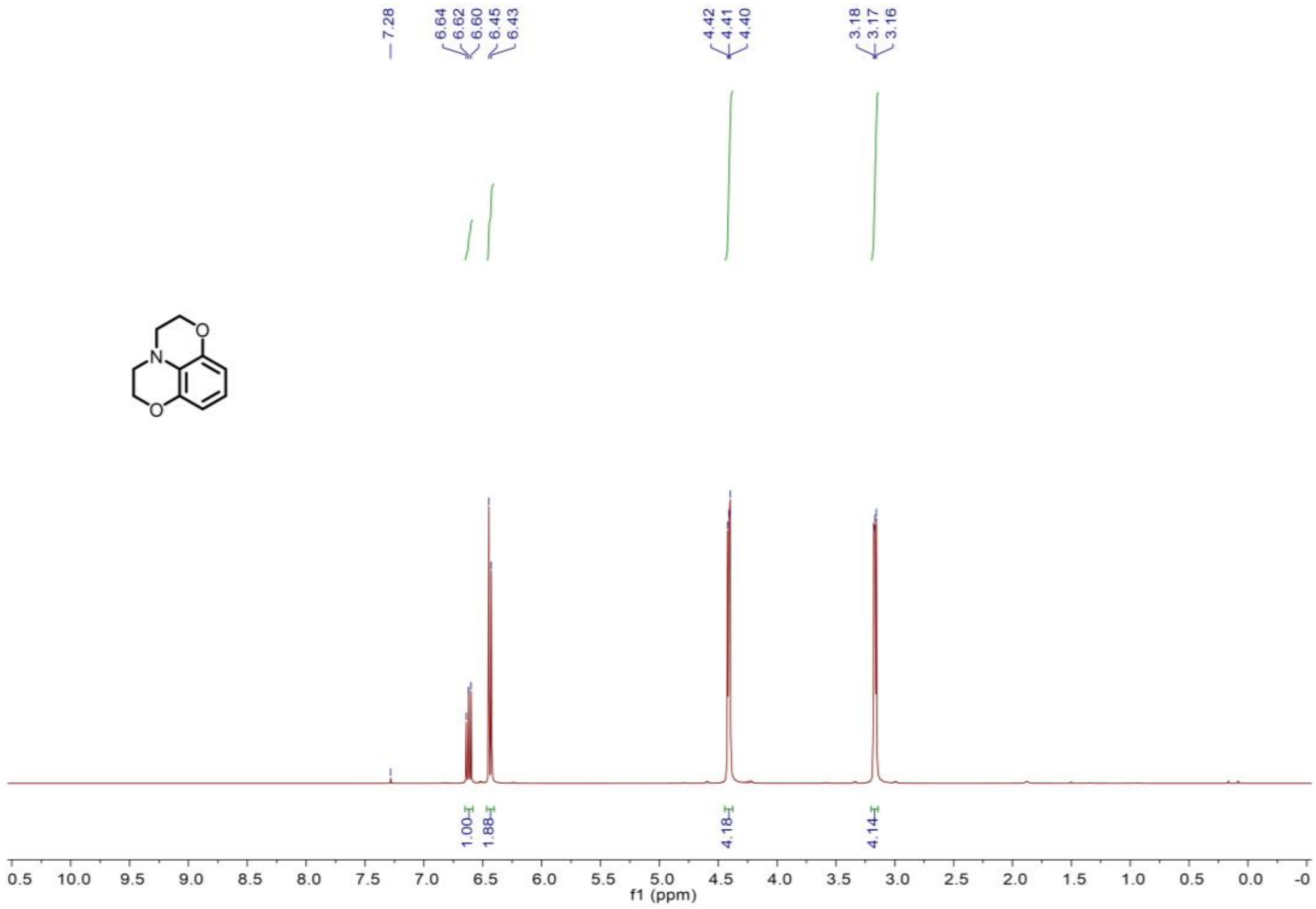

7.28
6.64
6.62
6.60
6.45
6.43
4.42
4.41
4.40
3.18
3.17
3.16
N
O
O
1.00
1.88
4.18
4.14
0.5
10.0
9.5
9.0
8.5
8.0
7.5
7.0
6.5
6.0
5.5
5.0
4.5
4.0
3.5
3.0
2.5
2.0
1.5
1.0
0.5
0.0
-0
f1 (ppm)

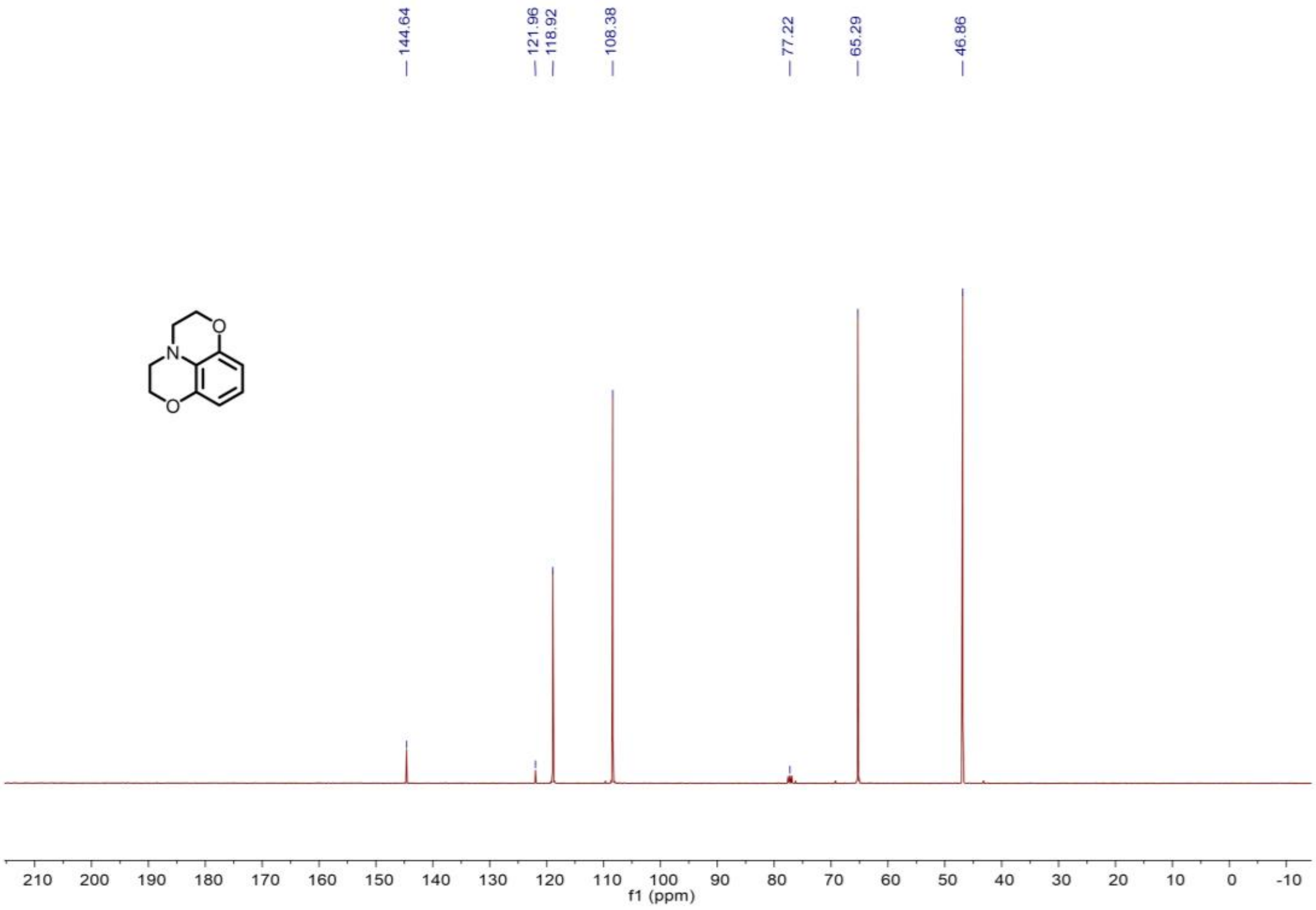

144.64
121.96
118.92
108.38
77.22
65.29
46.86
N
O
O
210
200
190
180
170
160
150
140
130
120
110
100
90
80
70
60
50
40
30
20
10
0
-10
f1 (ppm)

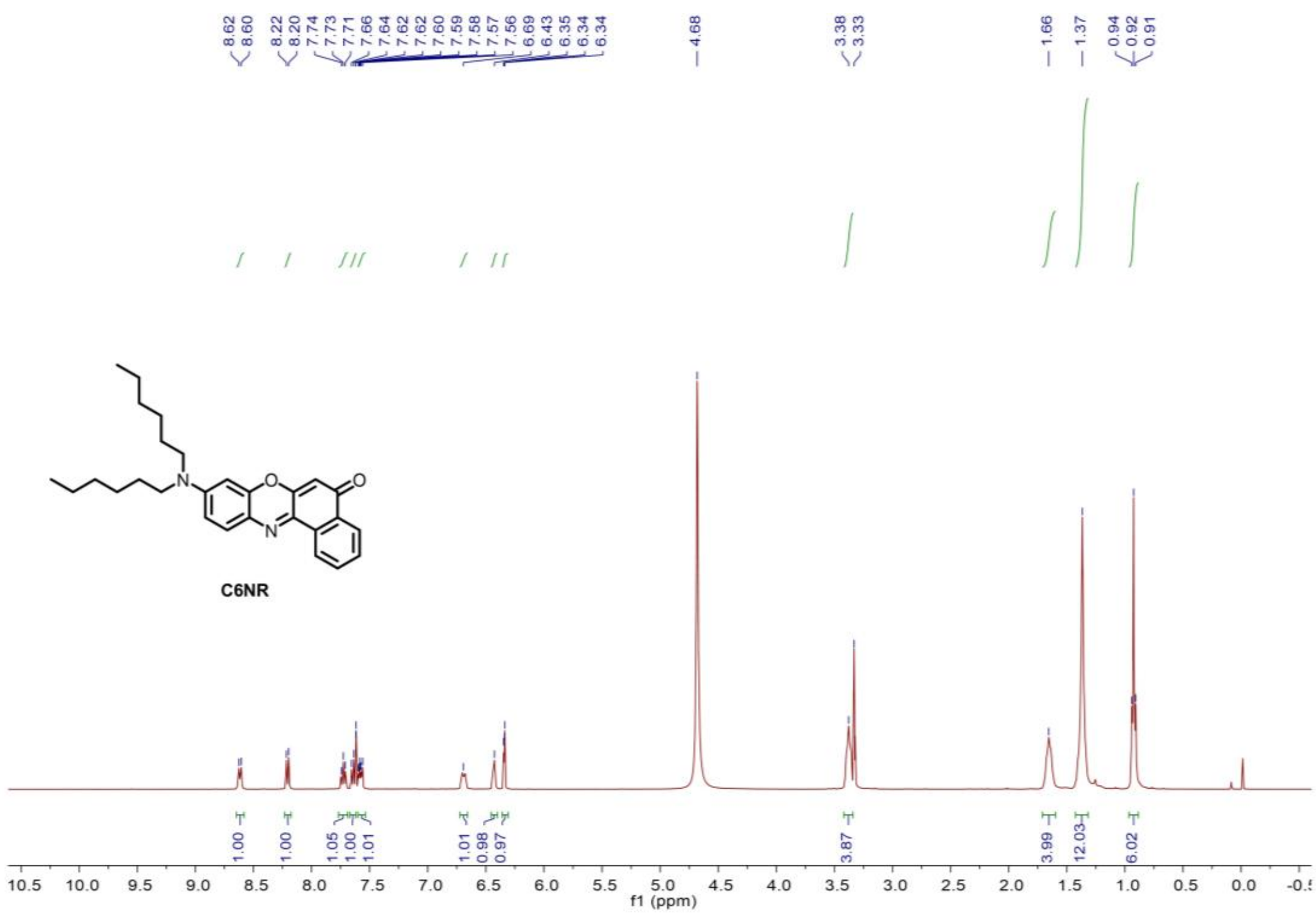
8.62
8.60
8.22
8.20
7.74
7.73
7.71
7.66
7.64
7.62
7.62
7.60
7.59
7.58
7.57
7.56
6.69
6.43
6.35
6.34
6.34
4.68
3.38
3.33
1.66
1.37
0.94
0.92
0.91
C6NR
1.00
1.00
1.05
1.00
1.01
1.01
0.98
0.97
3.87
3.99
12.03
6.02
f1 (ppm)

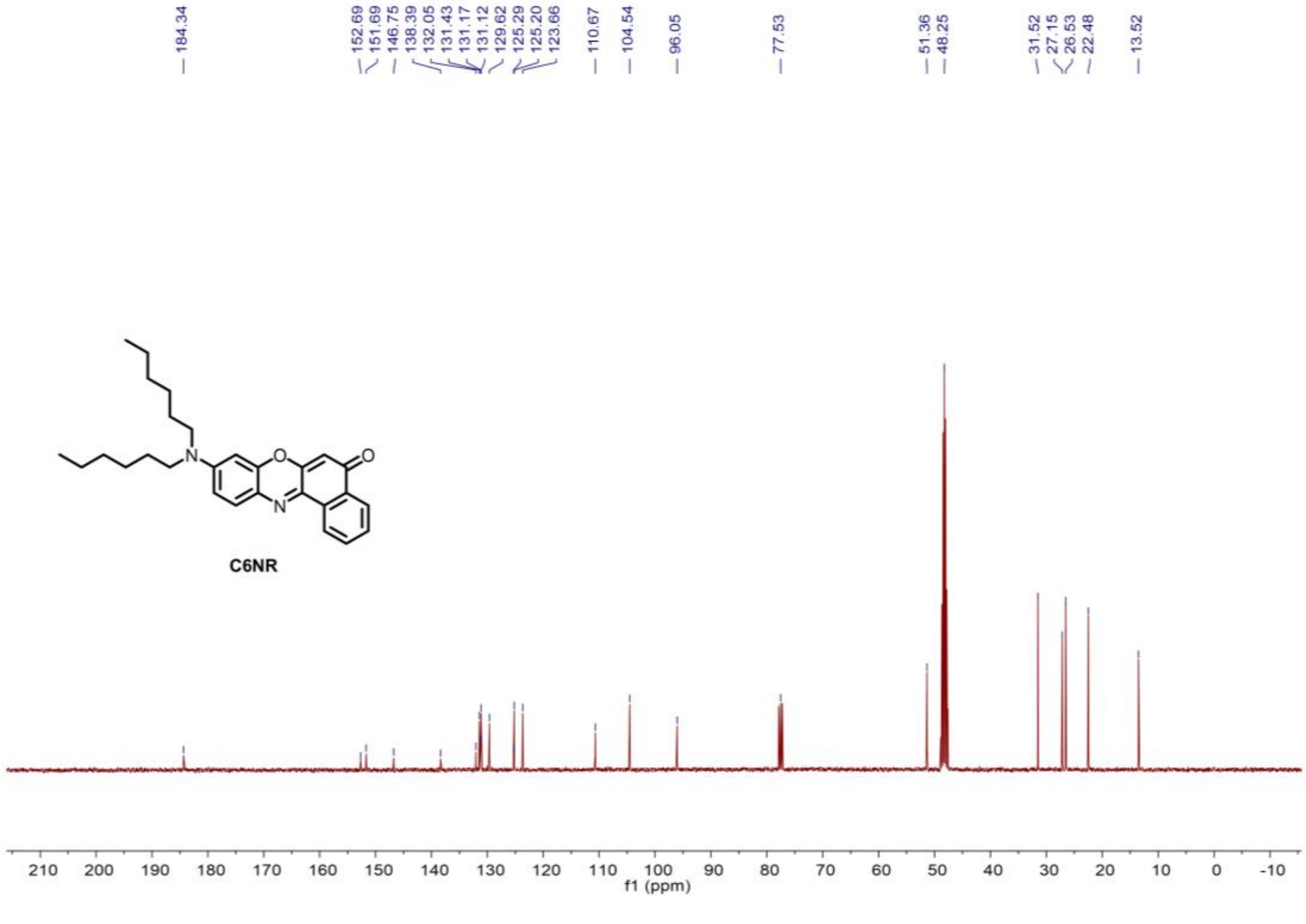
184.34
152.69
151.69
146.75
138.39
132.05
131.43
131.17
131.12
129.62
125.29
125.20
123.66
110.67
104.54
96.05
77.53
51.36
48.25
31.52
27.15
26.53
22.48
13.52
C6NR
f1 (ppm)

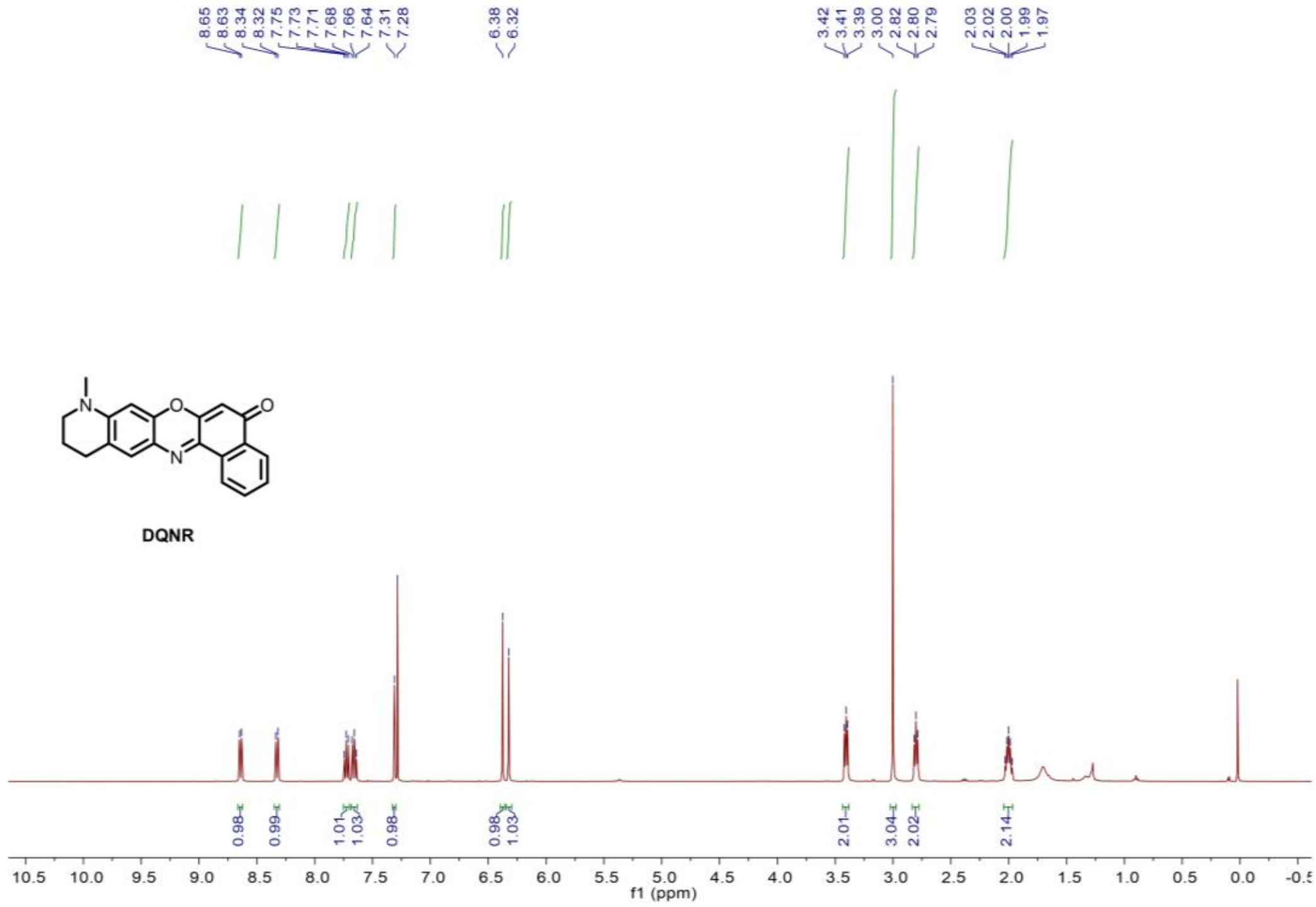
8.65
8.63
8.34
8.32
7.75
7.73
7.71
7.68
7.66
7.64
7.31
7.28
6.38
6.32
3.42
3.41
3.39
3.00
2.82
2.80
2.79
2.03
2.02
2.00
1.99
1.97
DQNR
0.98
0.99
1.01
1.03
0.98
0.98
1.03
2.01
3.04
2.02
2.14
f1 (ppm)

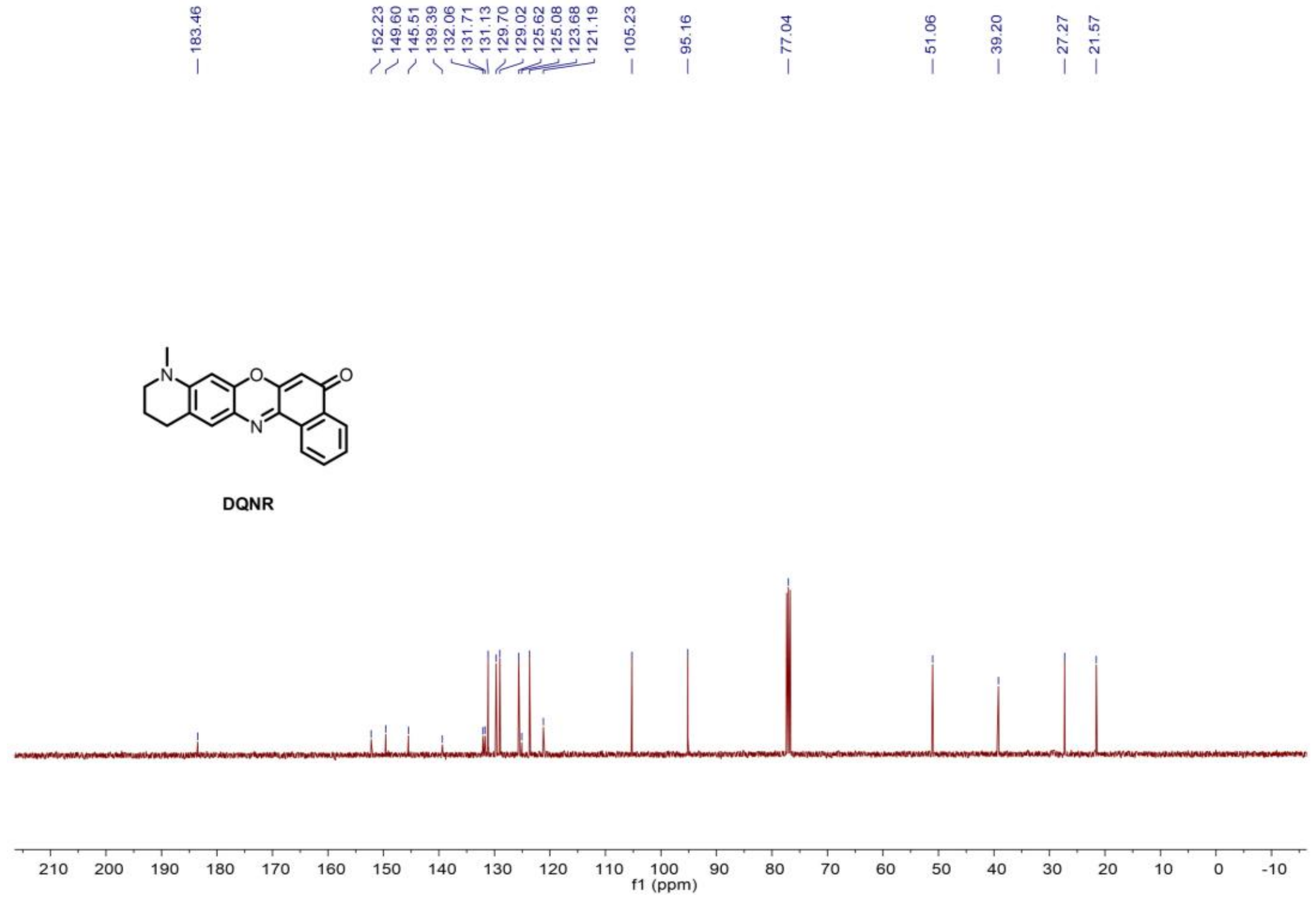
183.46
152.23
149.60
145.51
139.39
132.06
131.71
131.13
129.70
129.02
125.62
125.08
123.68
121.19
105.23
95.16
77.04
51.06
39.20
27.27
21.57
DQNR
f1 (ppm)

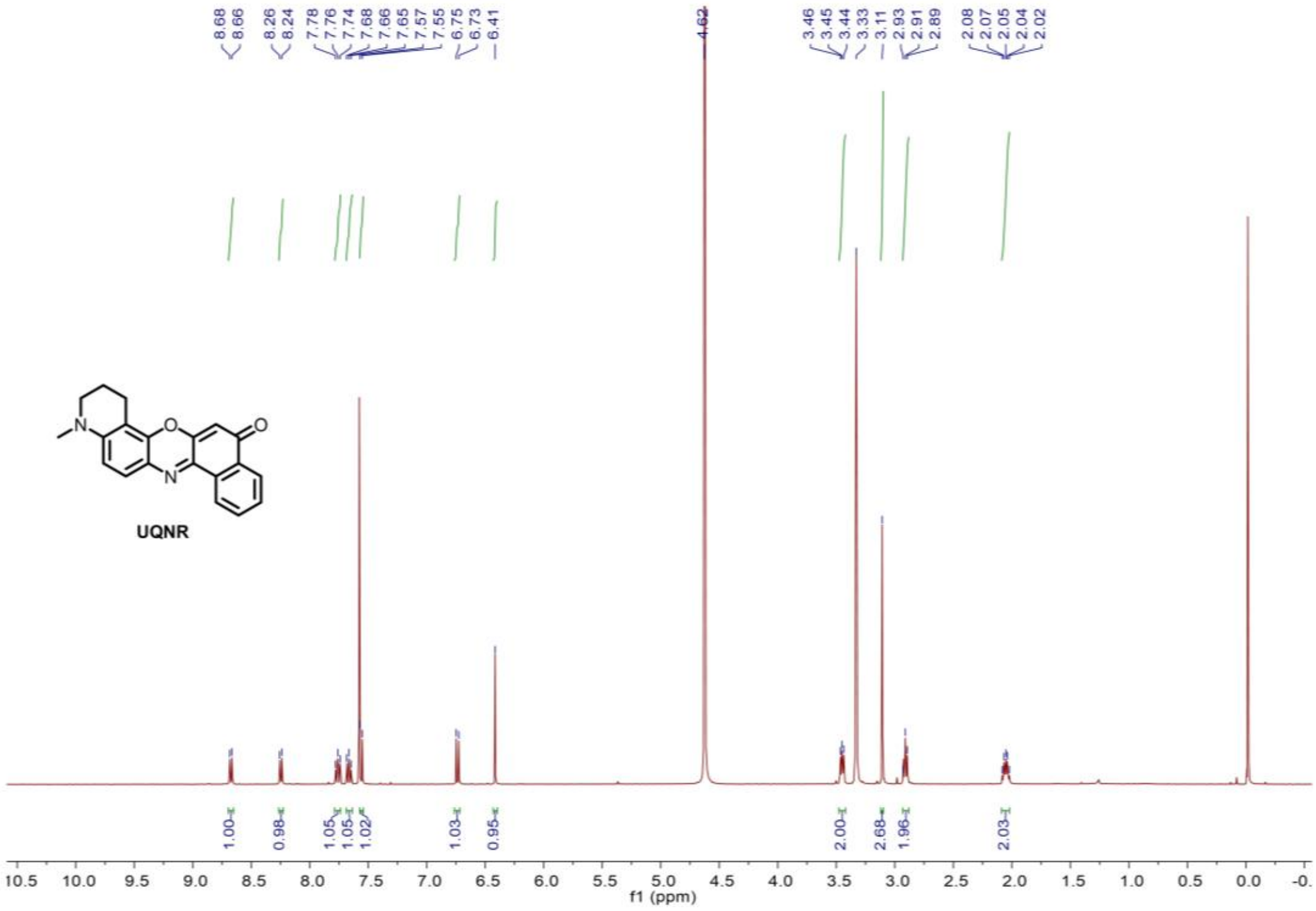
8.68
8.66
8.26
8.24
7.78
7.76
7.74
7.68
7.66
7.65
7.57
7.55
6.75
6.73
6.41
4.62
3.46
3.45
3.44
3.33
3.11
2.93
2.91
2.89
2.08
2.07
2.05
2.04
2.02
UQNR
1.00
0.98
1.05
1.05
1.02
1.03
0.95
2.00
2.68
1.96
2.03
f1 (ppm)

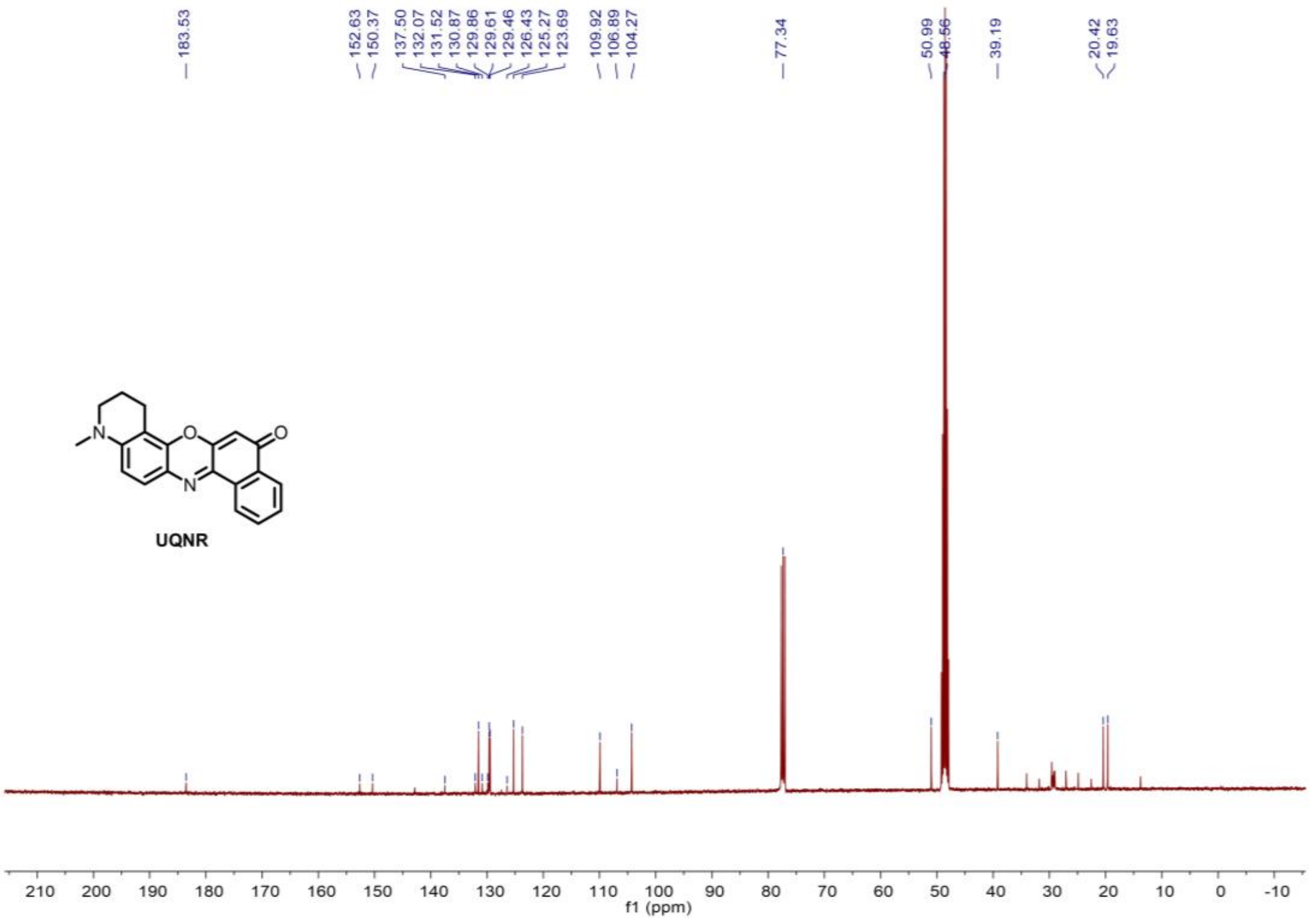
183.53
152.63
150.37
137.50
132.07
131.52
130.87
129.86
129.61
129.46
126.43
125.27
123.69
109.92
106.89
104.27
77.34
50.99
48.56
39.19
20.42
19.63
UQNR
f1 (ppm)

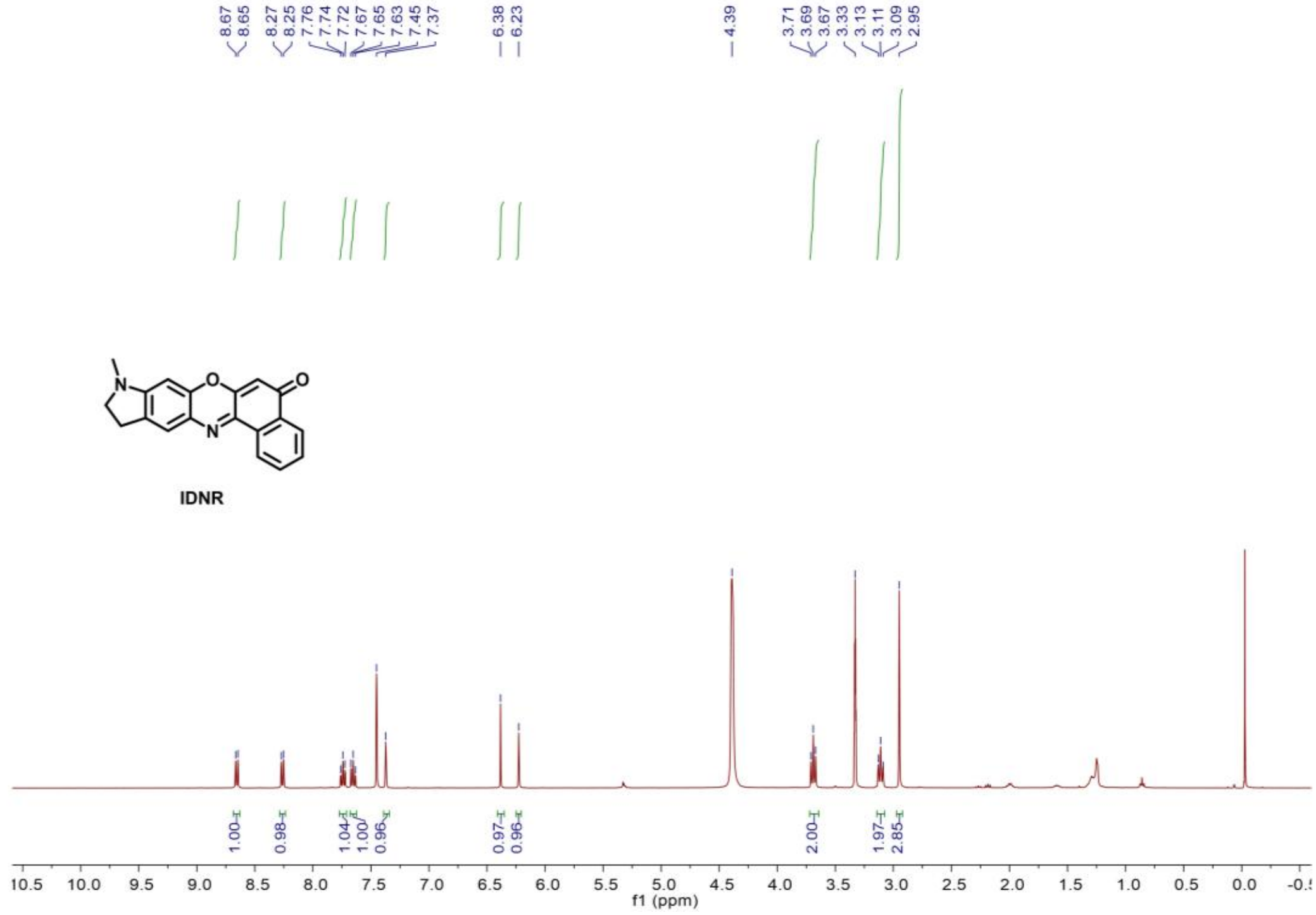
8.67
8.65
8.27
8.25
7.76
7.74
7.72
7.67
7.65
7.63
7.45
7.37
6.38
6.23
4.39
3.71
3.69
3.67
3.33
3.13
3.11
3.09
2.95
IDNR
1.00
0.98
1.04
1.00
0.96
0.97
0.96
2.00
1.97
2.85
f1 (ppm)

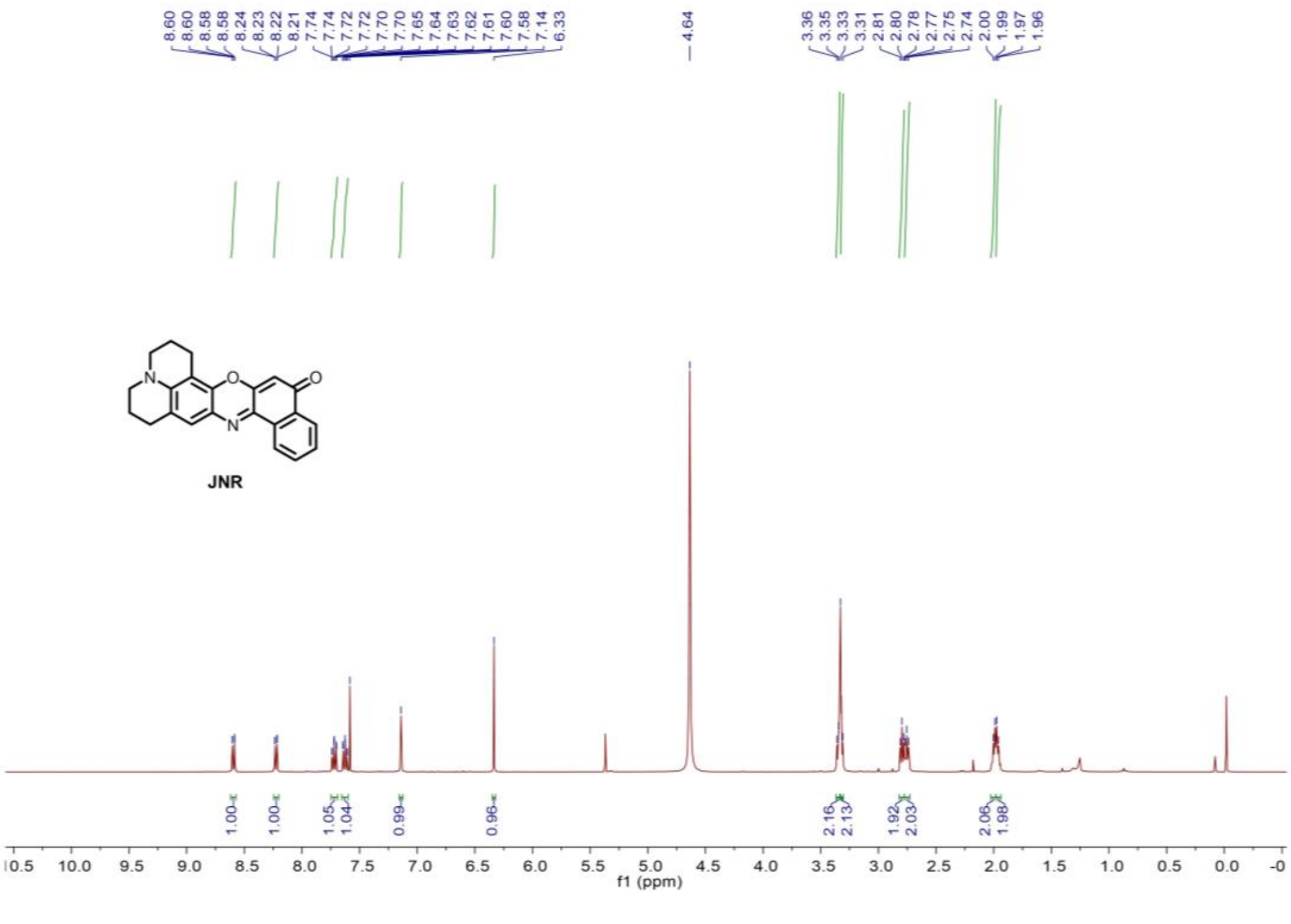
8.60
8.60
8.58
8.58
8.24
8.23
8.22
8.21
7.74
7.72
7.72
7.70
7.65
7.64
7.63
7.62
7.61
7.60
7.58
7.14
6.33
4.64
3.36
3.35
3.33
3.31
2.81
2.80
2.78
2.77
2.75
2.74
2.00
1.99
1.97
1.96
JNR
1.00
1.00
1.05
1.04
0.99
0.96
2.16
2.13
1.92
2.03
2.06
1.98
f1 (ppm)

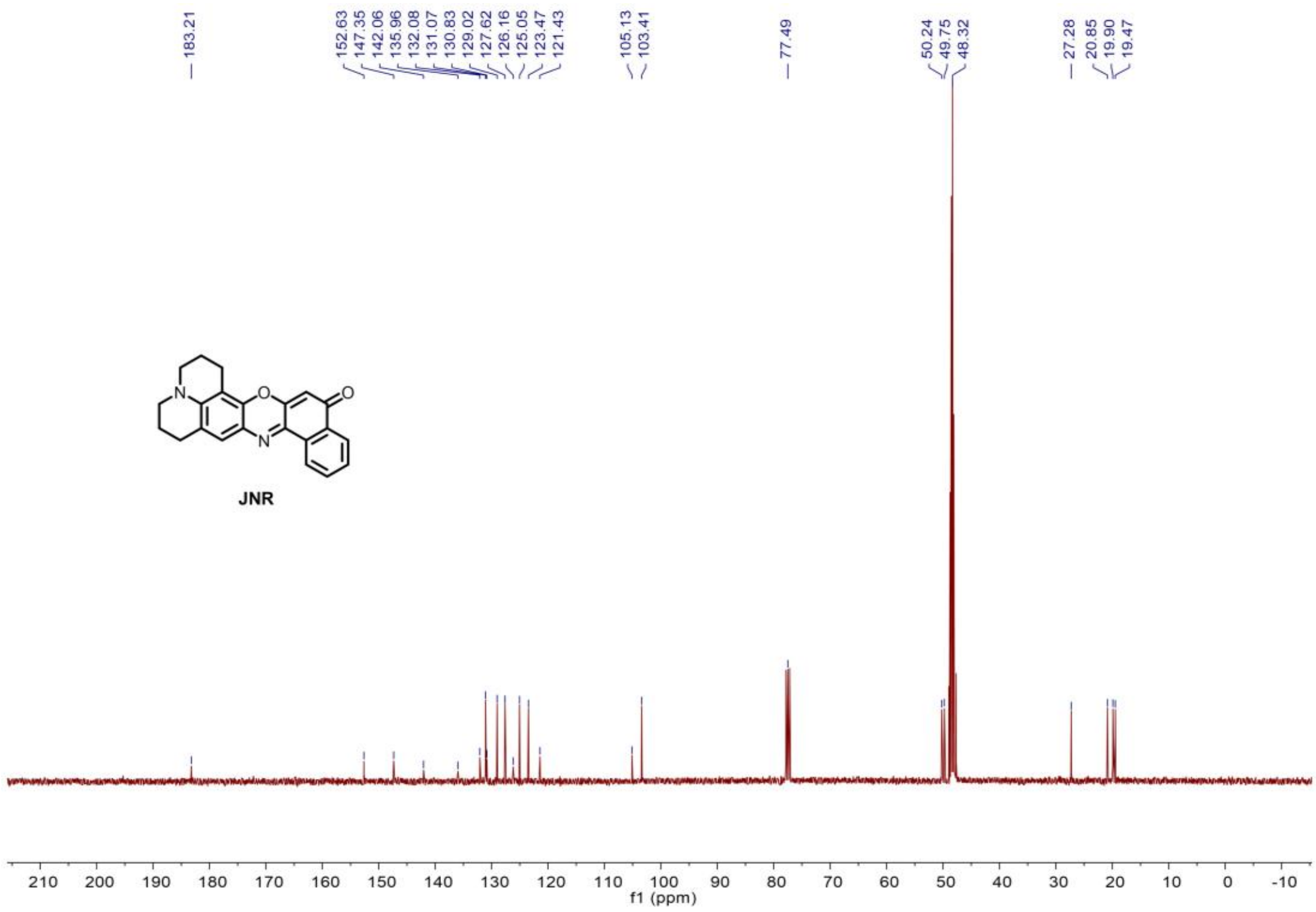
183.21
152.63
147.35
142.06
135.96
132.08
131.07
130.83
129.02
127.62
126.16
125.05
123.47
121.43
105.13
103.41
77.49
50.24
49.75
48.32
27.28
20.85
19.90
19.47
JNR
210 200 190 180 170 160 150 140 130 120 110 100 90 80 70 60 50 40 30 20 10 0 -10
f1 (ppm)

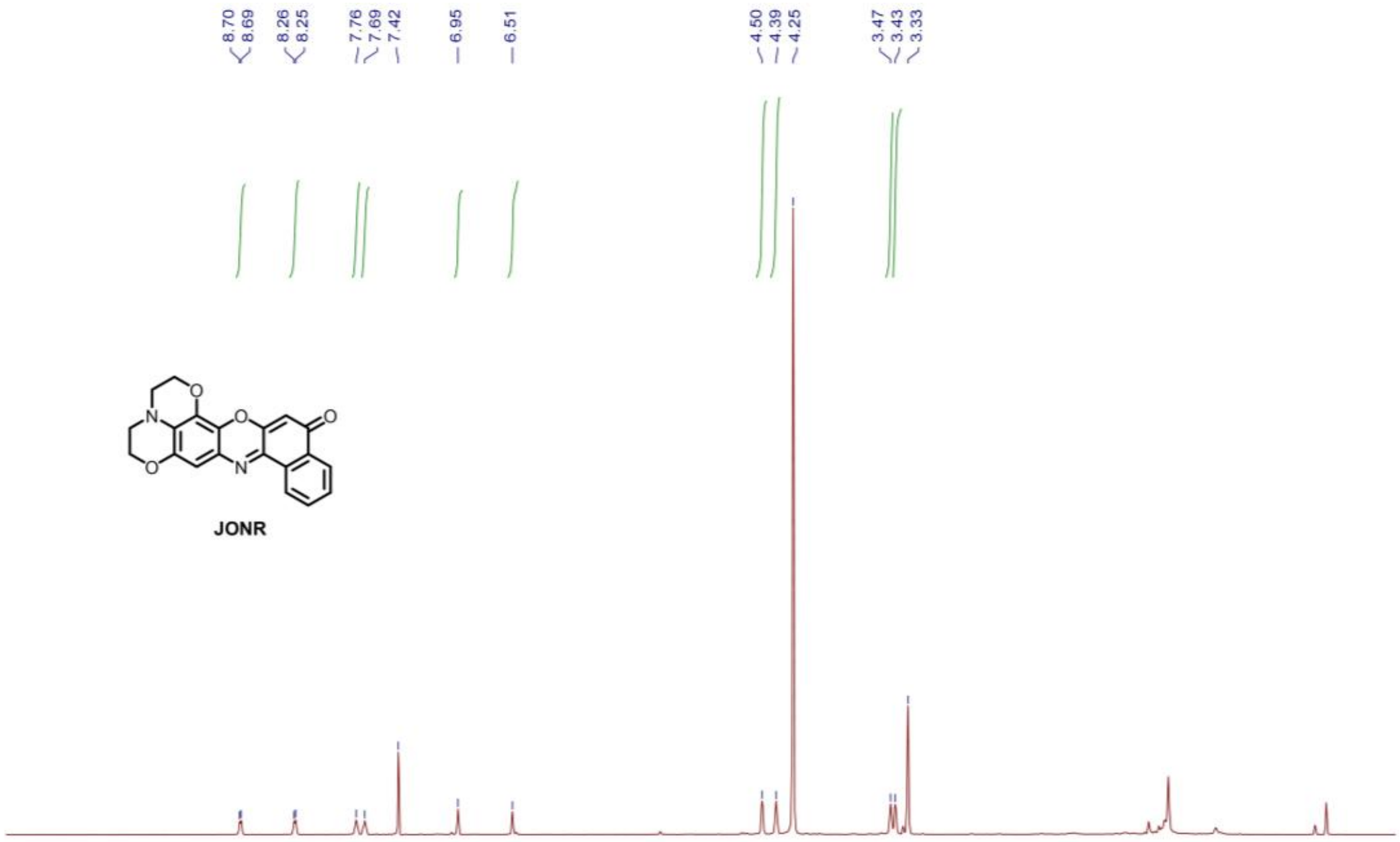
8.70
8.69
8.26
8.25
7.76
7.69
7.42
6.95
6.51
4.50
4.39
4.25
3.47
3.43
3.33
JONR
1.00
1.04
1.02
0.97
0.94
1.04
1.89
1.93
1.77
1.80
10.5 10.0 9.5 9.0 8.5 8.0 7.5 7.0 6.5 6.0 5.5 5.0 4.5 4.0 3.5 3.0 2.5 2.0 1.5 1.0 0.5 0.0
f1 (ppm)

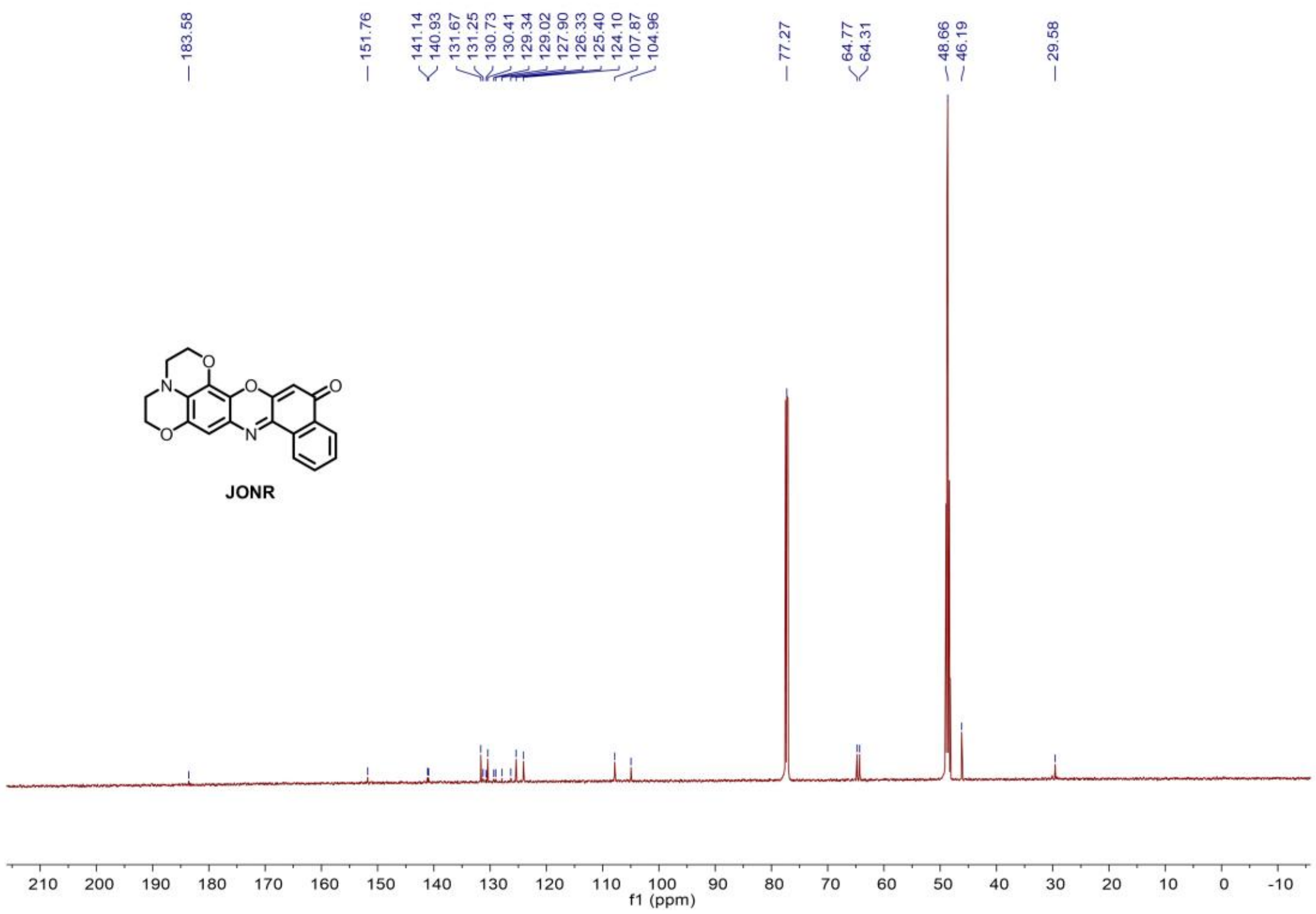
183.58
151.76
141.14
140.93
131.67
131.25
130.73
130.41
129.34
129.02
127.90
126.33
125.40
124.10
107.87
104.96
77.27
64.77
64.31
48.66
46.19
29.58
JONR
210 200 190 180 170 160 150 140 130 120 110 100 90 80 70 60 50 40 30 20 10 0 -10
f1 (ppm)
